# Expression of fatty acid and triacylglycerol synthesis genes in interspecific hybrids of oil palm

**DOI:** 10.1038/s41598-020-73170-5

**Published:** 2020-10-01

**Authors:** Ngoot-Chin Ting, Katrina Sherbina, Jia-Shiun Khoo, Katialisa Kamaruddin, Pek-Lan Chan, Kuang-Lim Chan, Mohd Amin Ab Halim, Kandha Sritharan, Zulkifli Yaakub, Sean Mayes, Festo Massawe, Peter L. Chang, Sergey V. Nuzhdin, Ravigadevi Sambanthamurthi, Rajinder Singh

**Affiliations:** 1 Advanced Biotechnology and Breeding Centre, Malaysian Palm Oil Board (MPOB), P.O. Box 10620, 50720 Kuala Lumpur, Malaysia; 2 Biotechnology Research Centre, School of Biosciences, University of Nottingham Malaysia Campus, Jalan Broga, 43500 Semenyih, Selangor Malaysia; 3 Quantitative and Computational Biology Section, University of Southern California, 1050 Childs Way, Los Angeles, CA USA; 4 Codon Genomics Sdn. Bhd. Malaysia, No. 26, Jalan Dutamas 7, Taman Dutamas, Balakong, 43200 Seri Kembangan, Selangor Malaysia; 5 United Plantations Bhd, Jendarata Estate, 36009 Teluk Intan, Perak Malaysia; 6 Plant and Crop Sciences, Sutton Bonington Campus, Sutton Bonington, University of Nottingham, Loughborough, LE12 5RD UK; 7 Molecular and Computational Biology Section, University of Southern California, 1050 Childs Way, Los Angeles, CA USA

**Keywords:** Gene expression, Transcriptomics, Quantitative trait, RNA sequencing

## Abstract

Evaluation of transcriptome data in combination with QTL information has been applied in many crops to study the expression of genes responsible for specific phenotypes. In oil palm, the mesocarp oil extracted from *E. oleifera* × *E. guineensis* interspecific hybrids is known to have lower palmitic acid (C16:0) content compared to pure African palms. The present study demonstrates the effectiveness of transcriptome data in revealing the expression profiles of genes in the fatty acid (FA) and triacylglycerol (TAG) biosynthesis processes in interspecific hybrids. The transcriptome assembly yielded 43,920 putative genes of which a large proportion were homologous to known genes in the public databases. Most of the genes encoding key enzymes involved in the FA and TAG synthesis pathways were identified. Of these, 27, including two candidate genes located within the QTL associated with C16:0 content, showed differential expression between developmental stages, populations and/or palms with contrasting C16:0 content. Further evaluation using quantitative real-time PCR revealed that differentially expressed patterns are generally consistent with those observed in the transcriptome data. Our results also suggest that different isoforms are likely to be responsible for some of the variation observed in FA composition of interspecific hybrids.

## Introduction

Currently, palm oil (with kernel oil) is the most important vegetable oil with ~ 75.2 million tons traded globally^1^. In comparison, other major oil crops supplied only 2.6–54 million tons^2^. The traded commodity provides an economic lifeline to many countries and people in Southeast Asia and Latin America. Oil palm is the subject of numerous research and development initiatives to further improve oil yield as well as modify its fatty acid composition (FAC). Studies were initiated as early as in the 1970s to decipher the genetic basis of important economic traits of oil palm^3–15^. The biosynthesis of fatty acids (FAs) and regulation of triacylglycerol (TAG) production are of particular interest because of the potential to produce specific feedstocks for a whole range of industries.

The development of next generation sequencing (NGS) technologies provides the opportunity for a more in-depth analysis of genes expressed, especially to understand the genome-wide regulatory mechanisms of oil biosynthesis in oil palm. Large-scale exploitation of NGS data for studying regulation and transcription of genes that govern FA and TAG synthesis has been reported for oil palm. Tranbarger et al*.*^16^ reconstructed the palm oil biosynthesis pathway in the mesocarp tissue. They revealed that the plastidial FA synthesis and the endoplasmic reticulum (ER)-based TAG synthesis are regulated by two distinct transcriptional programmes. The authors confirmed earlier findings by Ramli et al.^17^ that FA synthesis exerts a major control on the synthesis of storage oil. They showed that FA synthesis is most likely regulated by *WRINKLED* (*WRI1*), which encodes an *Apetala 2* (*AP2*) ethylene response element binding protein family transcription factor (TF). The regulatory role played by *WRI1* was further supported by other studies^18–21^. A bi-genic interaction via *WRI1* co-expressed with *palmitoyl-acyl carrier protein (ACP) thioesterase* (*FATB/PATE*) resulted in an increase in total oil produced^19^. The interaction network was further expanded by examining the co-expression of 23 genes from the oil palm FA synthesis pathway with other biological processes i.e. glycolysis, starch metabolism, plastid biogenesis and auxin transportation^20^.

Examining differential expression patterns of quantitative trait loci (QTLs) associated candidate genes and transcripts is an exciting new area for candidate gene validation in plants. This has provided interesting insights into the regulatory mechanisms of genes influencing quantitative traits. In chickpea (*Cicer arietinum* L.) for example, several SNP-containing candidate genes associated with height were shown to be differentially expressed between tall and dwarf/semi-dwarf parental lines^22^. Similarly, in potato (*Solanum tuberosum* L.), a number of genes that were differentially expressed between susceptible and resistant genotypes were also found in the genomic region associated with resistance to *Phytophthora infestans* (late blight)^23^. Additionally, in sorghum (S*orghum bicolor* L. Moench), candidate genes regulating nitrogen use efficiency (NUE) were revealed by the combined use of conventional QTL analysis and differential expression of genes (DEGs) in samples having contrasting NUE^24^. This strategy, to the best of our knowledge, has not been reported for oil palm although QTL information is available for many important traits including FAC^15,25^.

To date, almost all publications on transcriptome analysis in oil palm are based on *E. guineensis*, the commercially cultivated species in most of the oil palm growing countries. A second species of South American origin, *E. oleifera*, has lower yield but interesting characteristics, such as higher unsaturated oil content, resistance to some diseases, and reduced height^26,27^. As such, interspecific hybrid breeding has often carried out to introgress the desirable traits from *E. oleifera* into *E. guineensis* through F_1_ (OxG) and repeated backcrossing (BC)^13,28,29^. However, transcriptome analysis has not been reported for the interspecific OxG hybrids and, so far, is only available for interspecific backcross-one (BC_1_) palms^20^. Data for subsequent recurrent backcrossing, such as BC_2_ and BC_3_, are also lacking.

Therefore, in the present study, the global expression profiles of FA and TAG related genes (including their putative isoforms) in OxG and BC_2_ palms were evaluated. Differentially expressed genes were also identified in different genetic backgrounds across two developmental stages as well as between palms with contrasting palmitic acid (C16:0) content. FA-associated candidate genes, including *FATB* (/*PATE*), that were within the confidence intervals of the QTL previously identified for C16:0 and iodine value (IV, a measure of the degree of unsaturation) of mesocarp oil^15^ were further evaluated using quantitative real-time PCR (qRT-PCR). In OxG, these major QTLs which account for up to 69.0% of the phenotypic variation explained (PVE), were mapped to CHR03 (scaffolds p5_sc00001 and p5_sc00104) of the current oil palm genome build. Interestingly, the same QTL region was also identified independently in two interspecific BC_2_ populations^15^. In addition, we also discuss the fact that present findings in many aspects either support or have supporting evidence from the QTL and transcriptome analyses previously carried out for interspecific BC_1_ populations^20,25^.

## Materials and methods

### Palm materials

An interspecific OxG (Colombian *E. oleifera*, UP1026 x Nigerian *E. guineensis*, T128) and two separate BC_2_ (2.6–1 and 2.6–5) populations from a breeding program at United Plantations Berhad, Perak, Malaysia were sampled for this study (Supplementary Table S1). The common BC_1_ male parent of both the BC_2_ populations was obtained by crossing the La Mé *E. guineensis* × Colombian *E. oleifera* hybrid to a T128 palm. Subsequently, the 2.6–1 and 2.6–5 populations were created by backcrossing the BC_1_ male parent to an *E. guineensis* palm obtained from the cross pollination between T128 and a Serdang *pisifera* and from the self-pollination of palm T128, respectively. The FAC data for the OxG and the two BC_2_ populations was obtained as previously described^15^. For each population, two biological replicates with low C16:0 content (22.2–28.9%) and another two with high C16:0 content (33.1–40.6%) were selected. The characterization of low- and high-C16:0 content for the hybrid palms was determined based on the distribution of the C16:0 content in the respective populations. Fruit bunches were harvested for the four palms in each of the populations at two stages, namely early (up to 17/18 weeks after anthesis, WAA) and late (after 17/18 WAA) stages of mesocarp development. Individual intact fruitlets collected from each bunch were mixed and randomly sampled to obtain mesocarp tissue. The mesocarp was separated from shell and exocarp and immediately frozen in liquid nitrogen and stored at −80 °C prior to RNA extraction.

### Oil palm mesocarp RNA extraction

Total RNA was extracted from the mesocarp tissue using the cetyltrimethylammonium bromide method in combination with the silica column of RNeasy Plant Mini Kit (QIAGEN, Germany), as previously reported for *Jatropha curcas* L^30^. The method was optimized and modified for oil palm fruit tissues by Ong et al.^31^. The total RNA was purified using the RNeasy Mini kit and RNase-free DNase I following the manufacturer’s instructions (QIAGEN, Germany). The purified total RNA was eluted in 50 ul RNase-free water (QIAGEN, Germany) and the RNA purity and concentration were measured using a NanoDrop ND-1000 UV–Vis spectrophotometer (Thermo Fisher Scientific, USA). Total RNA with OD 260/280 and OD 260/230 ratios of ≥ 1.8 and 28S/18S ratios of ≥ 2.0 was acceptable and subjected to further quality check using the 2100 Bioanalyzer and the RNA 6000 LabChip kit following the manufacturer’s instructions (Agilent Technologies, USA). An RNA integrity number ≥ 7.0 suggests that the sample is suitable for RNA-seq.

### Library preparation and transcriptome sequencing

The ERCC spike-in RNA control (Thermo Fisher Scientific, USA) was added to the purified RNA (2–4 µg) at a concentration around 200 ng/µl to construct 24 libraries. A standard poly-A enrichment protocol was used for preparing the non-stranded RNA-seq libraries. The method captures the polyadenylated mRNA by hybridization to poly-T oligos bound to magnetic beads. The purified mRNA was fragmented and randomly primed to synthesize the double-stranded cDNAs. Subsequently, the cDNAs were end-repaired and ligated to paired-end adaptors prior to sequencing. Paired-end RNA-seq was performed using the Illumina HiSeq 4000 sequencing platform (Illumina, USA).

### RNA-seq data analysis

The RNA-seq raw sequence data were examined for sequence quality, sequence length distribution, sequence (A, C, G and T) content ratios, ambiguous base (N) content, sequence duplication, presence of adaptors and the percentage of GC content using FastQC v0.10.1. (https://www.bioinformatics.babraham.ac.uk/projects/fastqc/). Consistency and reproducibility of reads was also examined by comparing these parameters among the samples. Low quality read filtration was performed using the FastX toolkit v0.0.13.2 (www.hannonlab.cshl.edu/fastx_toolkit). Reads with Phred score < 20, length < 30 bp, and ambiguous bases and artefacts were discarded from further analysis.

Only good quality paired-reads in each library were mapped to the oil palm EG5.1 genome build^32^ using TopHat (https://ccb.jhu.edu/software/tophat/index.shtml) with mostly default parameters except for minimum intron length (30) and maximum intron length (50,000). The resulting BAM files of the aligned reads including splicing information were used as input into Cufflinks (https://github.com/cole-trapnell-lab/cufflinks) to assemble all possible transcripts in each library. The resulting transcript assemblies of the individual samples were then clustered using Cuffmerge (https://cole-trapnell-lab.github.io/cufflinks/cuffmerge/) to generate a set of non-redundant transcripts.

Homology-based functional annotation of the non-redundant transcripts was first carried out by performing sequence similarity search (BLASTX) against the Swiss-Prot/UniProtKB (https://www.ebi.ac.uk/uniprot) and Reference Sequence database (RefSeq, taxonomy: Magnoliophyta, https://www.ncbi.nlm.nih.gov/refseq/) using a maximum e-value cut-off at 1.0e^−5^. Using Blast2GO (https://www.blast2go.com/), the non-redundant transcripts were further mapped and annotated with Gene Ontology terms (GO, https://geneontology.org/page/download-ontology) and pathways from Kyoto Encyclopedia of Genes and Genomes (KEGG, https://www.genome.jp/) databases^33–35^. For the FA related genes, homology search also included the NCBI BioSystems database (https://www.ncbi.nlm.nih.gov/biosystems/) and the transcript and protein sequences previously published for FA genes^16,18–20,36^. However, this targeted gene search did not report additional genes/transcripts other than those already identified from the annotation pipeline. The expression profiles were normalized and represented as fragments per kilobase of transcript per million mapped fragments (FPKM) using Cuffdiff (https://cole-trapnell-lab.github.io/cufflinks/cuffdiff/).

### Differential expression analysis

FPKM values output by Cuffdiff were converted to read counts using the formula $\frac{{FPKM_{g} \times fl_{g} \times \mathop \sum \nolimits_{g \in G} r_{g} }}{{10^{9} }}$, where $fl_{g}$ is the length of gene, $r_{g}$ is the number of reads mapped to a gene, and *G* is the total set of genes to which reads were mapped. Using the R package DESeq2 1.18.1^37^, the read counts were normalized by library size and transformed using a regularized logarithm (rlog) taking into account the experimental design in estimating the dispersion. The rlog transformed data was used to compute Pearson correlation between all 24 RNA-seq libraries.

Significant changes in expression were determined using DESeq2 for genes with at least one read count in each of the 24 samples. Briefly, two different generalized linear models (GLM) with a logarithm link were fitted for each gene. The design matrix used to fit the model consisted of three factors: (i) population (OxG, 2.6–1 and 2.6–5); (ii) mesocarp developmental stages, namely early and late development and, (iii) C16:0 content (low- and high-C16:0). The log_2_ fold-change (FC) coefficients were estimated using the Wald test and contrasts of the coefficients were set up to test whether the difference between groups (*i.e*. levels of a factor) was zero. The Benjamini and Hochberg procedure^38^ was used to control the false discovery rate at *p* < 0.01. To focus on genes that not only have a significant change in expression but effect size, we performed additional tests with the GLM aforementioned for the null hypothesis that the FC are less than or equal to |1|.

### Quantitative real-time PCR (qRT-PCR)

The same stock of the extracted mesocarp RNA (used for RNA-seq) was also used in the qRT-PCR experiment. Template without reverse transcriptase (NRT) and no-template control (NTC) were also included to determine the possibility of genomic DNA contamination, presence of primer-dimers or any spurious amplification. The qRT-PCR was performed using Mastercycler ep realplex (Eppendorf, Germany) and the BioMark HD system (FLUIDIGM, USA). For the Mastercycler ep realplex system, conversion of total RNA to single-stranded cDNAs was carried out using the high-capacity cDNA reverse-transcription kit following the manufacturer’s instructions (Applied Biosystems, USA). Preparation of qRT-PCR master reaction mixtures and the PCR programme used were as described by Chan et al.^39^.

For both the reference and candidate genes, the qRT-PCR data was analysed using geNorm 3.4^40^. Eight reference genes (*Ubiquitin*, *pOP-EA01332*, *Actin*, *GAPDH*, *NAD5*, *Tubulin*, *GRAS* and *Cyp2*)^39,41^ were tested and the results showed that the *GAPDH*, *ACTIN* and *Cyp2* combination gave the most satisfactory expression stability (M < 1.5) (Supplementary Fig. S3). The primer information is available in Supplementary Table S3. The selected reference gene set was subsequently used for normalizing the expression of candidate genes. Mean Ct across three replicates in a sample was calculated and transformed into the relative expression quantities using formula Q = E(minCt—sampleCt) where, E = Ex + 1 and minCt is the smallest Ct observed among the tested samples. For a candidate gene, the term representing relative expression quantities was Q_GOI_ whereas, Q_refij_ refers to the expression quantities for the selected reference genes. Both the terms were subsequently used for estimation of normalized expression for a candidate gene, GOI_norm_ = Q_GOI_/Q_refij._ In the case where three reference genes were selected, Q_refij_ = (Q_ref1_ × Q_ref2_ × Q_ref3_)1/3. Calculation for standard deviations (SD) for each GOI_norm_ was also carried out as described in the geNorm manual^40^.

For the BioMark HD system, cDNA stock was prepared using the FLUIDIGM reverse transcription master mix. Preparation of qRT-PCR master reaction mixtures, preamplification, Delta gene assay and qRT-PCR were carried out by following the manufacturer’s instructions (FLUIDIGM, USA). Default parameters were used for collecting and analysing the data where expression values (delta delta Ct, ddCt) were normalized against the same set of reference genes consisting of *GAPDH*, *ACTIN* and *Cyp2* (determined when qRT-PCT was performed in the Mastercycler ep realplex system) and the calibrator (which was the pooled samples). Fold change of ddCt (ddCtFC) = 2^−ddCt^ was also calculated representing the relative changes of gene expression^42^. Significance (*p* < 0.05) of differential expression between low- and high-C16:0 content groups was determined using t-test (SPSS 16.0).

## Results

### Gene functional annotation

Total RNA extracted from mesocarp tissue of the 12 interspecific palms harvested at two stages of fruit development were sequenced using the Illumina HiSeq 4000 sequencing platform (Illumina, USA). For each sample, the abundance of ERCC transcripts was measured and normalized to FPKM using the TopHat-Cufflinks pipeline as described in the RNA-seq data analysis section. The dose response of ERCC spike-in RNA transcripts showed strong correlation between the sequencing read counts and RNA inputs with an average R^2^ of 0.9115. An average of 118 million quality paired-reads were generated after filtration. Approximately 79.0–88.0% of the paired-reads generated were successfully mapped to the EG5.1 genome. A vast majority of these reads (97.7%) was mapped uniquely and only 2.3% of the reads mapped at multiple locations on EG5.1 using the mapping criteria applied in this study.

The combined assembly of all 24 samples yielded 43,920 potential genes (with nomenclature XLOC_) consisting of 141,513 putative isoforms (nomenclature TCONS_). The putative isoforms were searched for plant sequence homology in several protein databases including RefSeq, Swiss-Prot and GO. The results showed that 88.7 and 63.7% of the assembled transcripts were homologous to known proteins in RefSeq and Swiss-Prot, respectively (Table 1). The majority of the significant similarities (97,319 transcripts) from the RefSeq BLAST search was to genes from *E. guineensis* and 10,878 aligned to date palm (*Phoenix dactylifera*) genes. The annotation to GO terms resulted in 160 genes (consisting of 588 putative isoforms) and 38 genes (202 putative isoforms) assigned to FA (GO:0006633) and TAG (GO:0019432) biosynthetic processes, respectively. In fact, a larger number of GO terms was found to be associated with various FA and lipid related biological processes and are listed in Supplementary Table S2.

**Table 1:** Functional annotation of 43,920 potential genes (141,513 putative isoforms) against four public databases.

Public database	Annotated genes	Annotated isoforms	Unigene annotation rate (%)
RefSeq	30,657	125,548	88.72
Swiss-Prot	21,380	96,066	67.88
GO*	20,069	90,117	63.68
KEGG**	4059	18,062	12.76

*At least in one of the three main categories of GO terms (biological process, molecular function and cellular component);

**Metabolic pathways.

In order to understand the possible metabolic and biological functions of these putative genes, 918 were successfully mapped to 147 metabolic pathways in the KEGG database. A total of 72 genes consisting 312 putative isoforms from the present study were mapped to the FA biosynthesis pathway. Transcripts were also assigned to other related pathways involving metabolic processes of pyruvate, α-linolenic acid (C18:3), linoleic acid (C18:2), biosynthesis of unsaturated FAs, FA elongation and FA degradation. A number of genes were also mapped to lipid related pathways such as 168 genes (887 putative isoforms) that were mapped to the glycerolipid metabolic pathway, which includes the TAG formation process. Other than FA and TAG, genes were also assigned to the glycerolphospholipid, sphingolipid and other lipid related pathways (Supplementary Fig. S1).

### Expression of genes related to FA and TAG biosynthetic processes

The transcripts that mapped to the EG5.1 genome build were converted to FPKM. Transcripts with FPKM = 0 refers to no expression while FPKM > 0 refers to some expression. However, low FPKM may also be due to technical artefacts and, therefore FPKM expression values > 0.1 were accepted as being a more reliable threshold. The number of expressed transcripts with FPKM > 0.1 were consistent across all samples in the validation panel, ranging from 74,476–81,999 (52.6–57.9%) transcripts in OxG, 73,366–80,919 (51.8–57.2%) in BC_2_ (2.6–1) and 76,575–82,303 (54.1–58.2%) in BC_2_ (2.6–5) (Supplementary Fig. S2). It was also consistently observed that the number of expressed transcripts was higher in the earlier mesocarp developmental stage compared to late stage. In the earlier stage, the average expressed transcripts observed in OxG, BC_2_ (2.6–1) and BC_2_ (2.6–5) were 81,620, 78,027 and 80,651, respectively. Whereas, a slightly lower number of transcripts 77,986, 77,387 and 78,457 were observed in the later stage of the three respective crosses.

Compilation of the top-50 highly expressed genes in each sample resulted in a total of 340 genes. Of these, 110 matched to 535 GO terms, including biosynthesis of FA (GO:0006633) unsaturated FA (GO:0006636), TAG (GO:0019432), acyl-carrier-protein (GO:0042967), lipid oxidation (GO:0034440) and transportation (GO:0006869). This suggests that the FA and lipid related genes were actively expressed during the two ripening stages when the fruits were sampled.

**Figure 1:**
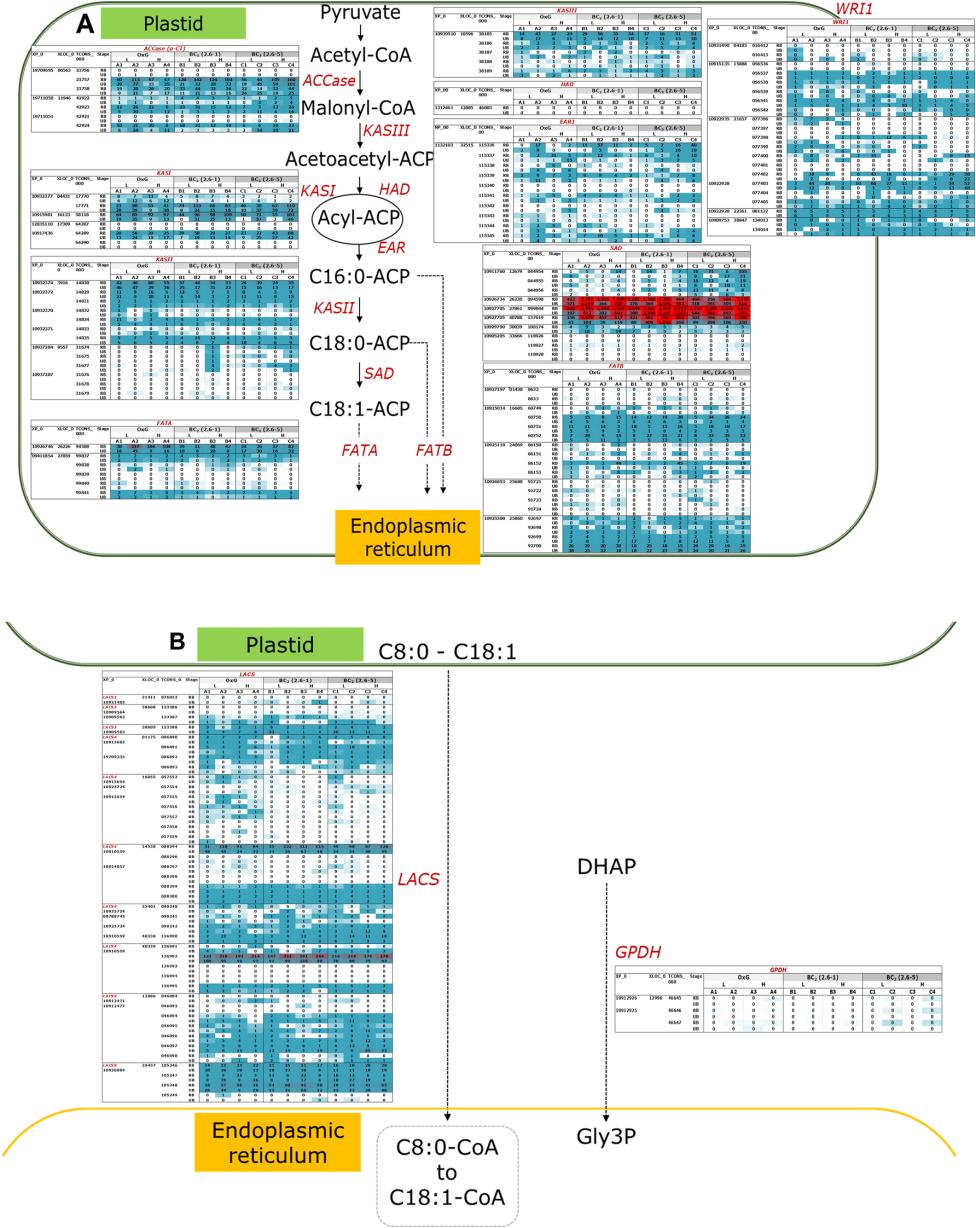

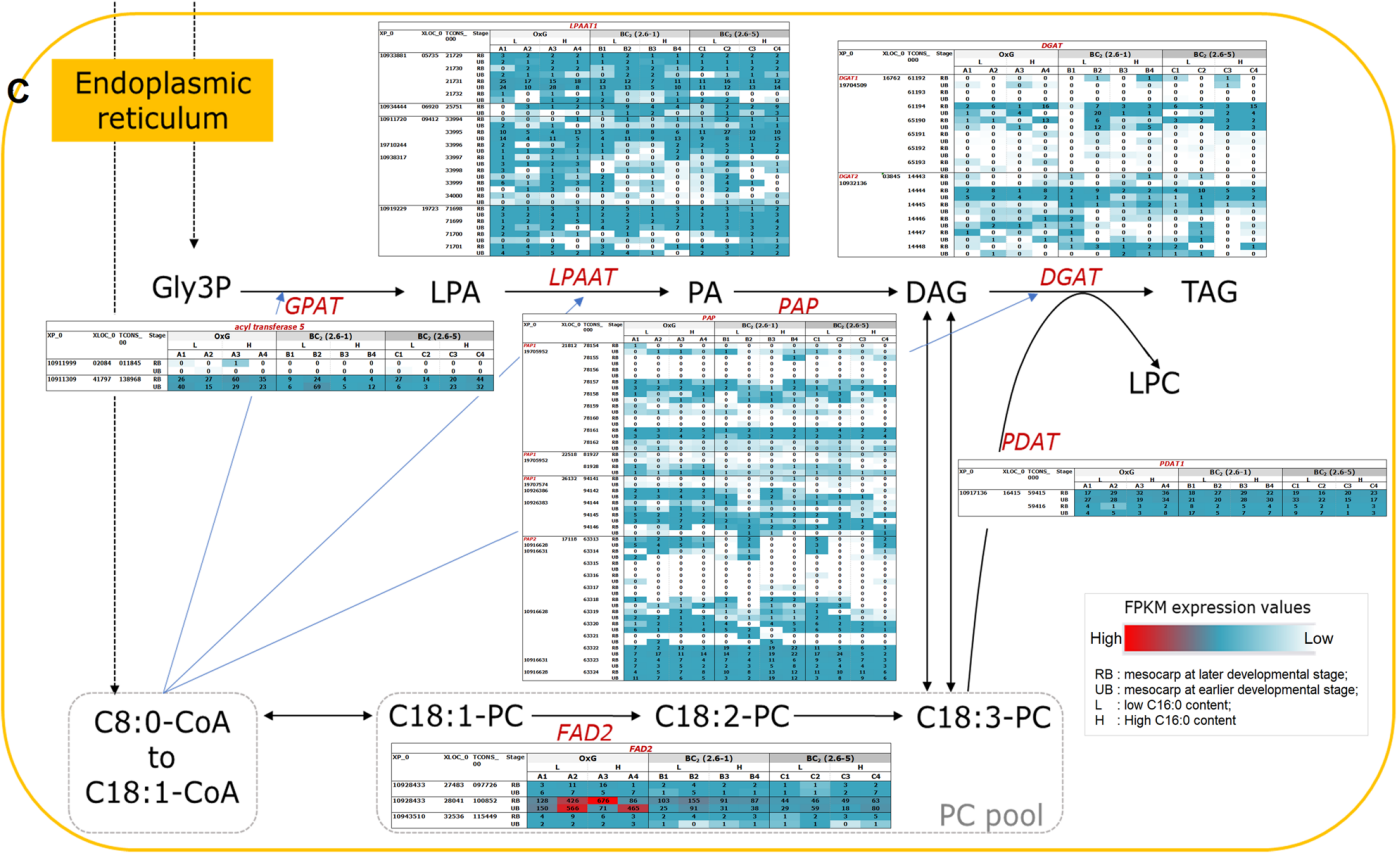
Transcriptomic expression levels for the putative isoforms (TCONS_) of genes (XLOC_) involved in (A.) biosynthesis of fatty acids in plastids, (B.) channeling of non-esterified fatty acids from plastid to endoplasmic reticulum and (C.) biosynthesis of triacylglycerol in the endoplasmic reticulum. Only enzymes (in red font) encoded by genes identified from the present study are shown in the diagram. Identified genes: acetyl-CoA carboxylase carboxyl transferase subunit alpha {ACCase (α-CT)}; β-ketoacyl-ACP synthases (KAS I, II and III); hydroxyacyl-ACP dehydrase (HAD); enoyl-ACP reductase (EAR/ENR); Δ9-stearoyl-ACP desaturase (SAD); chloroplastic omega-3 fatty acid desaturase (FAD7/8); acyl-ACP thioesterases (FATA and B); AP2 ethylene-responsive transcription factor (WRI1); long-chain acyl-CoA synthetases (LACS1, 2, 4 and 9); glycerol-3-phosphate dehydrogenase (GPDH); glycerol-3-phosphate acyltransferase (suspected to be GPAT5); lysophosphatidic acid acyltransferase (LPAAT1); phosphatidic acid phosphatases (PAP1 and 2); diacylglycerol:acyl-CoA acyltransferases (DGAT1 and 2); delta-12 fatty acid desaturase (FAD2) and phosphatidylcholine:diacylglycerol acyltransferase (PDAT).

In relation to the biosynthesis of FAs, major genes encoding enzymes such as acetyl-CoA carboxylase (ACCase), beta-ketoacyl-ACP synthases (KAS I, II and III), hydroxyacyl-ACP dehydrase (HAD), enoyl-ACP reductase (EAR/ENR); Δ9-stearoyl-ACP desaturase (SAD), ACP thioesterases mainly FATA and FATB, long-chain acyl-CoA synthetase (LACS) and the *WRI1* transcription factor were identified from GO:0006633, GO:0006636 and GO:0008610. One to five putative paralogous genes (XLOC_) were found for each of these, which in most cases were located on different chromosomes. A majority of the genes was also found to have multiple putative isoforms such as XLOC_021657 of *WRI1* and XLOC_032515 of *EAR1* consisting of up to ten putative isoforms. The isoforms were expressed at varying levels in the two mesocarp developmental stages analysed (Fig. 1A and B). For XLOC_021657 of *WRI1*, TCONS_00077403 and _00077402 were the two most actively expressed putative isoforms, detected in most of the later stages of mesocarp development with FPKM values up to 65.67 and 43.31, respectively. In contrast, very low expression was detected for TCONS_00077397 and _00077401 of the same TF in both stages of fruit development across all the three genetic backgrounds. Although, TCONS_00077400 had considerably higher expression of up to 15.15 FPKM, it was only expressed in about 50.0% of the samples in the validation panel. Other putative isoforms of *WRI1* mostly demonstrated either no expression or had low FPKM compared to TCONS_00077400. Variability in expression of these putative isoforms could contribute to the multiple roles that *WRI1* plays during oil palm fruit development.

**Figure 2:**
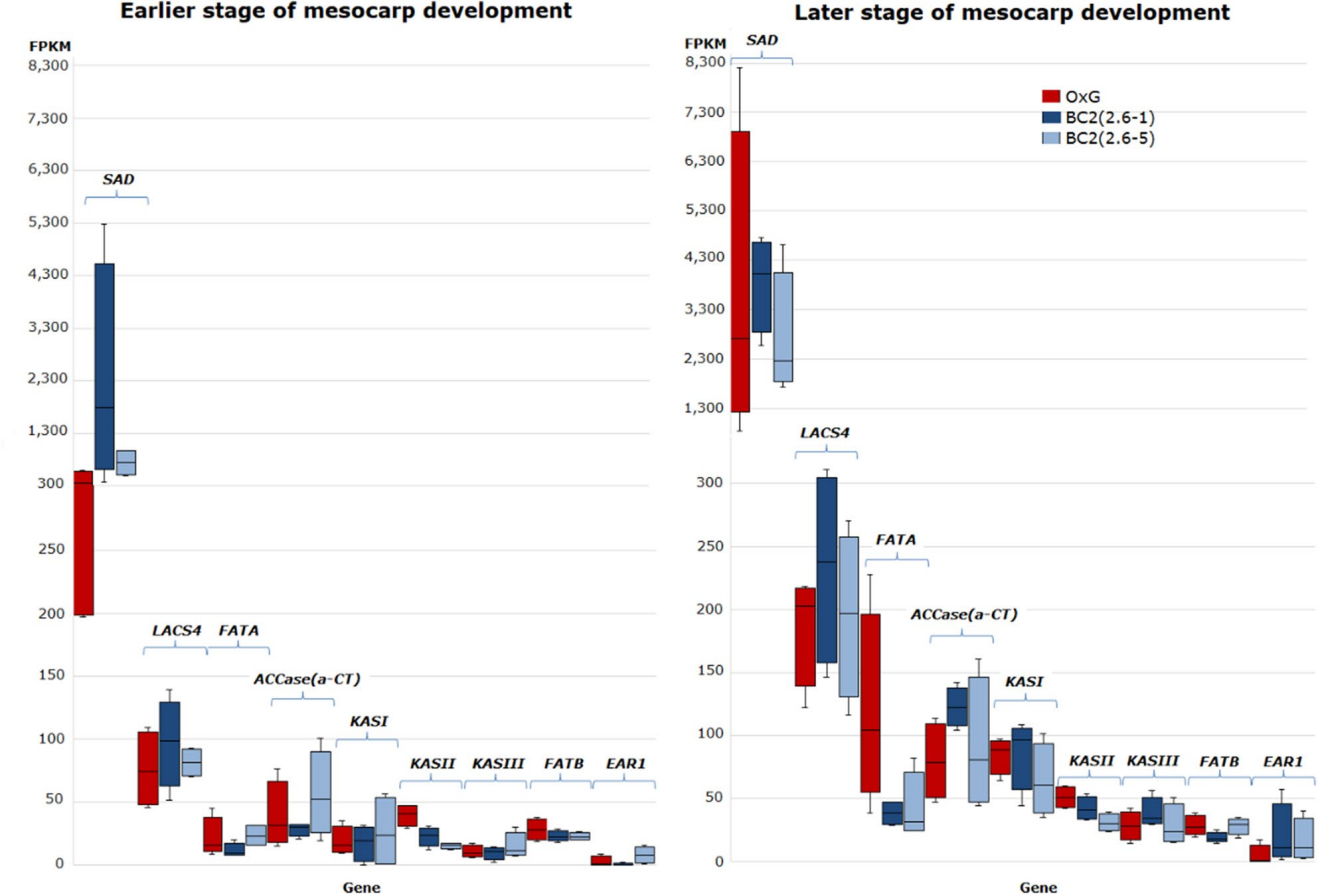
FPKM expression levels for various genes mapped to the fatty acid biosynthesis pathway as observed in the early (left panel) and late (right panel) stages of mesocarp development.

The three putative genes (XLOC_027861, _026230 and _040788) identified as *SAD* (XP_010926734 and _010927705), which is involved in conversion of C18:0- to C18:1-ACPs, were the most highly expressed genes among the identified FA synthesis genes throughout the biosynthesis pathway (Figs. 1A and 2). High FPKM values were observed for XLOC_027861 (TCONS_00099844) ranging from 196.76–5271.14 and 829.13–8192.63 in the earlier and later stages of mesocarp development, respectively. For another two *SAD* gene variants, the observed FPKM values in the earlier and later stages were in the range of 263.73–1575.63 and 256.16–2382.01 for XLOC_026230 and, 39.32–1103.72 and 156.46–1574.87 for XLOC_040788.

The next most highly expressed FA genes were *FATA* and *LACS4*. High expression levels in the later stage of fruit development, ranging from 24.28–227.23 were observed for the putative gene XLOC_026226 of *FATA*. For *LACS4*, six transcript variants were identified of which XLOC_040339 (TCONS_00136992) and XLOC_024538 (TCONS_00088294) were found to be abundantly expressed in the later stages of mesocarp development in all three families with FPKM values in the range of 116.24–311.02 and 48.92–126.30, respectively. In comparison, a lower expression level was observed at the earlier stage of mesocarp development, ranging from 45.88–138.90 (XLOC_040339, TCONS_00136992) and 22.83–61.81 (XLOC_024538, TCONS_00088294).

For the TAG biosynthetic pathway, putative genes and isoforms encoding enzymes glycerol-3-phosphate dehydrogenase (GPDH), glycerol-3-phosphate acyltransferase (GPAT), lysophosphatidic acid acyltransferase (LPAAT), phosphatidic acid phosphatase (PAP), diacylglycerol:acyl-CoA acyltransferase (DGAT), phosphatidylcholine:diacylglycerol acyltransferase (PDAT) and delta-12 fatty acid desaturase (FAD2) were also identified and mapped to various steps of the TAG synthesis and FA modification pathways in ER (Fig. 1C). Among these, *FAD2* (XLOC_028041) was the most actively expressed gene with FPKM values ranging from 18.39–566.19 and 44.10–676.13 measured in the earlier and later stages of mesocarp development, respectively. This ER located desaturase introduces the second double bond to the PC-bound C18:1 thereby creating C18:2-PC. However, *omega-3 fatty acid desaturase* (*FAD3*) which is involved in subsequent desaturation to form C18:3-PC, was not detected in this GO term. This is not surprising since the mesocarp has about 10.0% C18:2 and only negligible levels of C18:3. However, a chloroplastic *omega-3 fatty acid desaturase (FAD7/8*, XP_010920844) was identified which had high expression levels similar to *FAD2*. Chloroplastic omega-3 fatty acid desaturase introduces the third double-bond in the biosynthesis of C16:3 and C18:3 FAs, which are important membrane constituents. It is also interesting to note that the *FAD2* expression levels were higher than the those of *GPAT* (< 70 FPKM), *LPAAT* (< 30 FPKM), *PAP* (< 25 FPKM) and *DGAT* (< 21 FPKM) genes that are involved in the Kennedy pathway. The results concur with evidence presented by Ramli et al.^17^ that FA synthesis exerts a more important effect than the Kennedy pathway in the regulation of oil synthesis in oil palm. The present study indicates this to be so even in interspecific hybrids and the backcrosses.

### DEGs in different genetic backgrounds across early and late developmental stages

**Figure 3:**
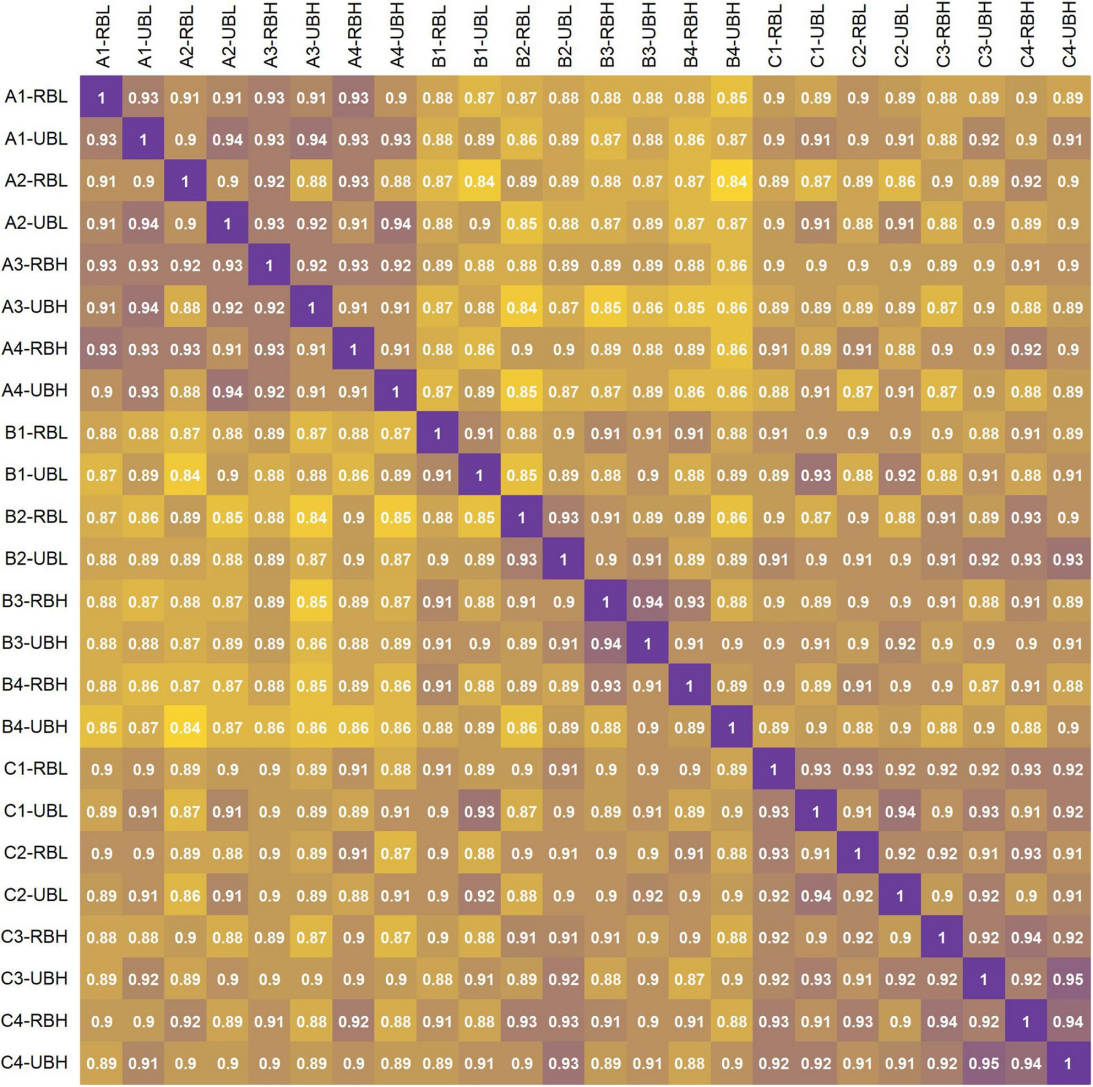
Pearson correlation between all 24 samples using regularized logarithm transformed RNA-seq data plotted using R version 4.0.0 (r-project.org). Mesocarp samples are categorised into early (up to 17/18 WAA) and late (after 17/18 WAA) developmental stages denoted by the plot’s row/column labels containing -U and -R, respectively. Samples labelled as A1–4; B1–4 and C1–4 were from OxG, BC2 (2.6–1) and BC2 (2.6–5) populations, respectively. For C16:0 content, “low” (-U/RBL) ranged from 22.2–28.9% while “high” (-U/RBH) ranged from 33.1–40.6% with respect to the level of C16:0 in mesocarp.

For each of the 12 palms, the Pearson correlation in the normalized read counts for 43,920 genes was high, ranging from 0.89 to 0.94 (Fig. 3). Additionally, the OxG samples regardless of developmental stage or C16:0 content, had a higher range of pairwise correlation to each other than to samples from either of the BC_2_ populations. Looking at samples from within the same population, some pairs of samples from the same developmental stage had a generally higher correlation than to samples from another stage.

DESeq2 was used to investigate population, developmental stage, and C16:0 content specific differences in expression of genes with at least one read count per sample, which reduced the number of potential genes under consideration to 24,496. Differential gene expression amongst pairs of the three populations, mesocarp developmental stages and C16.0 content was determined using the Wald test. The largest number of DEGs was identified between developmental stages followed by between pairs of populations and then between high and low C16:0 content (Table 2). By comparing the two mesocarp developmental stages, a total of 6,094 DEGs was identified of which 217 were related to GO terms for biosynthetic, metabolic, oxidation and transportation processes of FAs, TAGs, lipids, glycerol-3-phosphate and acyl-carrier-protein. Of these, it is worth noting that 21 DEGs were among the key genes (Fig. 1) involved directly in biosynthesis of FAs (GO:0006633) and TAGs (GO:0019432). As expected, 19 of these DEGs showed a significantly higher expression ranging from 0.4–2.36 FC at the later stage compared to early stage of fruit development.

**Table 2:** The number of genes with a significant change in expression among the OxG, BC_2_ (2.6–1) and (2.6–5) populations, mesocarp developmental stages (late and early) and C16.0 content (high and low). Significant DEGs at adjusted *p* value < 0.01 are presented for up-regulation (FC > 0 and > 1) and down-regulation (FC < 0 and < −1).

Contrast	H_0_: log_2_ fold change (FC) = 0		H_0_: |log_2_ fold change (FC)|< = 1	
	FC > 0	FC < 0	FC > 1	FC < −1
BC_2_ (2.6–1) vs. OxG	1919 (7.80%)	1852 (7.60%)	69 (0.28%)	118 (0.48%)
BC_2_ (2.6–5) vs. OxG	637 (2.60%)	774 (3.20%)	20 (0.08%)	81 (0.33%)
BC_2_ (2.6–5) vs. BC_2_ (2.6–1)	352 (1.40%)	369 (1.50%)	2 (0.008%)	5 (0.02%)
Late vs. early stage	3120 (13.00%)	2974 (12.00%)	162 (0.66%)	154 (0.63%)
High- vs. low-C16.0	14 (0.06%)	10 (0.04%)	0	0

Among the population contrasts, the highest number of genes with significant expression changes were observed between OxG and BC_2_ (2.6–1) (3771 DEGs) followed by 1411 DEGs in OxG vs. BC_2_ (2.6–5) and 721 DEGs when comparing the two BC_2_ populations (Table 2). The number of DEGs involved in the FA and TAG biosynthesis pathways was also found correspondingly reduced from eight to five and one across the three respective contrasts (Table 3). There were only 24 DEGs identified between high- vs. low-C16:0 content palms. Unfortunately, none were found to be involved in FA or TAG biosynthetic activities.

**Table 3:** Differentially expressed genes (DEGs) related to synthesis of FA (GO:0006633, GO:0006636 and GO:0008610) and TAG (GO:0019432) identified by comparing the two mesocarp developmental stages (late and early) and amongst the OxG, BC_2_ (2.6–1) and (2.6–5) populations. Significant in expression at adjusted *p* value (padj) < 0.01 determined with H_0_: log_2_ fold change (FC) = 0.

Gene	DEG (XLOC_)	Corresponding gene position in EG5.1 build		Late vs. early stage		BC2 (2.6–1) vs. OxG		BC_2_ (2.6–5) vs. OxG		BC2 (2.6–5) vs. BC2 (2.6–1)	
		CHR/Sc	Position (bp)	FC	padj	FC	padj	FC	padj	FC	padj
*ACCase_1*	006563	CHR12	12,184,042–12,203,276	1.14	0.00018469	–	–	–	–	–	–
*KASIII_1*	010596	CHR15	19,057,442–19,067,706	1.24	0.00004816	–	–	–	–	–	–
*KASI_1*	004431	CHR10	23,056,853–23,066,783	1.17	0.00162919	–	–	–	–	–	–
*KASI_2*	016133	CHR03	15,058,145–15,063,433	1.95	0.00175106	–	–	–	–	–	–
*KASI_3*	017309	CHR03	38,723,675–38,767,004	1.12	0.00054201	–	–	–	–	–	–
*KASII_1*	003916	CHR10	22,940,770–22,949,844	0.40	0.00636450	−0.52	0.00609184	−0.76	0.00006136	–	–
*SAD_3*	027861	CHR08	8,306,193–8,310,109	1.99	0.00001669	–	–	–	–	–	–
*SAD_4*	040788	Sc08824	1899–2580	1.76	0.00023363	–	–	–	–	–	–
*FATA_1*	026226	CHR07	23,339,761–23,350,100	1.69	0.00000485	−1.36	0.00537388	–	–	–	–
*FATA_2*	027859	CHR08	8,152,030–8,156,099	0.89	0.00036109	–	–	–	–	–	–
*FATB_1*	016685	CHR03	1,846,010–1,852,083	1.98	0.00000000	–	–	–	–	–	–
*WRI1_3*	021657	CHR05	39,782,472–39,805,223	2.31	0.00344690	–	–	–	–	–	–
*LACS2_2*	038609	Sc01482	169,573–173,778	–1.95	0.00009942	–	–	–	–	–	–
*LACS4_1*	016055	CHR03	11,141,682–11,177,588	–	–	−3.36	0.00000000	−1.60	0.00064250	1.76	0.00038627
*LACS4_2*	001175	CHR01	8,875,904–8,916,992	1.88	0.00000117	–	–	–	–	–	–
*LACS4_3*	024538	CHR06	40,749,497–40,760,176	1.08	0.00000079	–	–	–	–	–	–
*LACS4_5*	040338	Sc06230	1531–7626	1.46	0.00263216	–	–	–	–	–	–
*LACS4_6*	040339	Sc06230	1531–7626	1.26	0.00000001	–	–	–	–	–	–
*LACS9_1*	012886	CHR02	24,690,189–24,711,942	−1.57	0.00008391	–	–	–	–	–	–
*LPAAT1_1*	005735	CHR11	19,068,230–19,102,990	–	–	−0.63	0.00799942	–	–	–	–
*LPAAT1_2*	006920	CHR12	2,630,995–2,662,553	2.36	0.00003887	–	–	–	–	–	–
*PAP_1*	021812	CHR05	47,506,332–47,513,305	–	–	−0.58	0.00972337	–	–	–	–
*PAP_2*	022518	CHR05	47,506,332–47,513,305	–	–	−1.11	0.00529959	–	–	–	–
*DGAT2_1*	003845	CHR10	19,704,781–19,710,080	1.12	0.00018840		–	–	–	–	–
*FAD2_1*	027483	CHR08	27,389,878–27,394,517	–	–	–	–	−1.70	0.00995809	–	–
*FAD2_2*	028041	CHR08	27,389,878–27,394,517	–	–	−2.17	0.00004566	−2.72	0.00000032	–	–
*FAD2_3*	032536	Sc00131	551,194–554,932	1.80	0.00000002	−1.34	0.00154280	−1.30	0.00468157	–	–

### Evaluation of the QTL-linked DEGs using qRT-PCR

Of the 27 DEGs related to FA (GO:0006633) and TAG (GO:0019432) biosynthetic processes, *FATB_1* (XLOC_016685) and *LACS4_1* (XLOC_016055) were also previously located within the QTL confidence regions (ranging from 1.5–11 Mbp on CHR03) associated with myristic acid (C14:0), C16:0, stearic acid (C18:0), oleic acid (C18:1) and IV^15^. These genes and their putative isoforms were quantified in the present study using the OxG and BC_2_ palms with contrasting C16:0 content.

**Figure 4:**
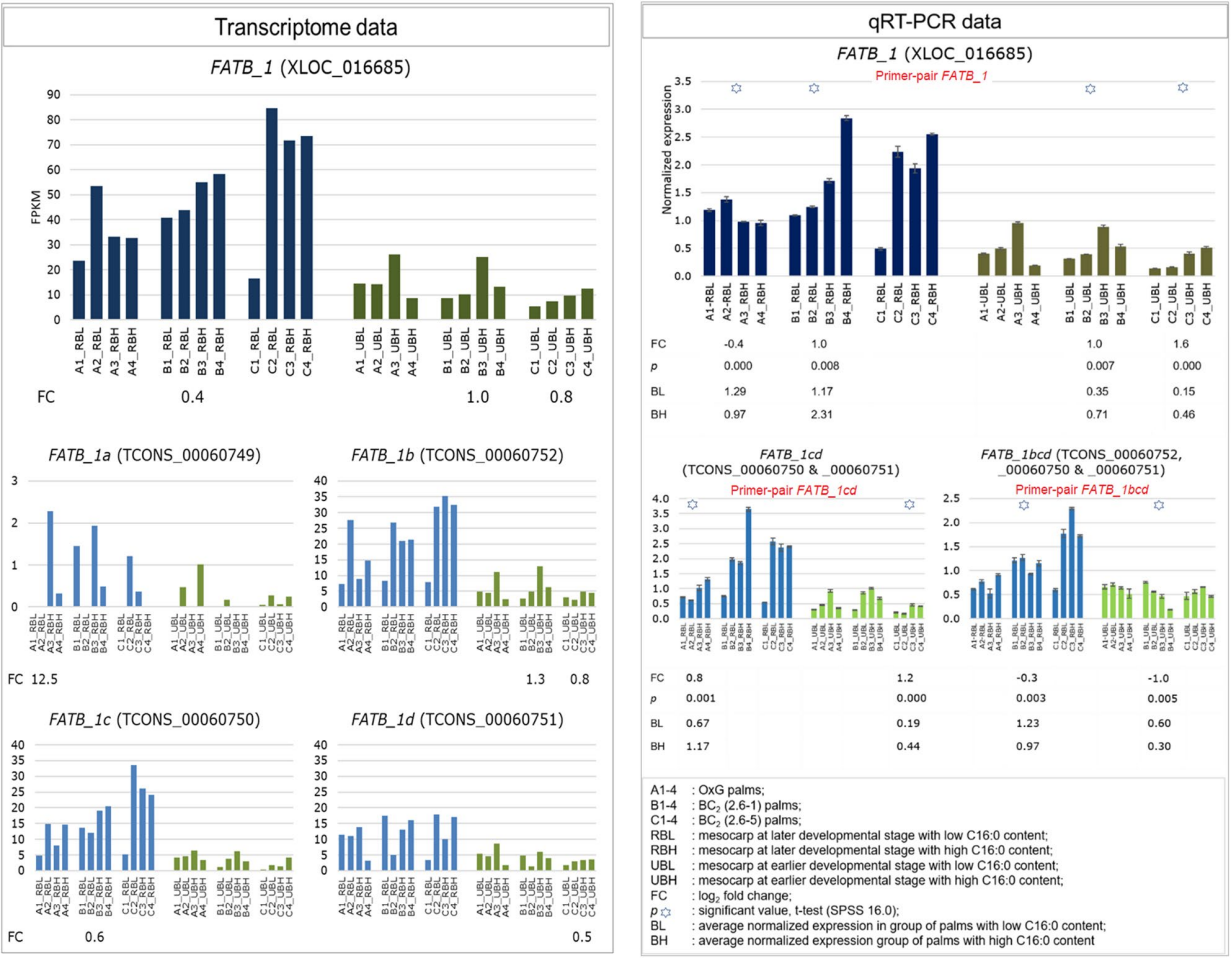
Normalized expression profiles of *FATB_1* (XLOC_016685) and the isoforms (TCONS_) quantified by qRT-PCR (right panel) in comparison with the transcriptomic expression profiles (left panel). The primers for qRT-PCR were designed to amplify the common exonic regions between isoforms denoted by two/three letters.

Expression profiles observed in the qRT-PCR experiment were compared to those observed for the corresponding genes and isoforms in the transcriptome data. A similar expression pattern was observed for *FATB_1* in which, an obvious increase in expression was observed at the later stages of mesocarp development compared to the earlier stages (Table 3, Fig. 4). More specifically, at the later stages of mesocarp development, significant differential expression (*p* < 0.05) of *FATB_1* was noticeable in specific comparison groups *i.e.* A1&2_RBL vs. A3&4_RBH in OxG and B1&2_RBL vs. B3&4_RBH in BC_2_ (2.6–1). However, at the earlier fruit developmental stages, only contrasting palms in BC_2_ (B1&2_UBL vs. B3&4_UBH and C1&2_UBL vs. C3&4_UBH) demonstrated significant differential expression. The *FATB_1* gene (XLOC_016685) demonstrated a higher level of expression in the BC_2_ palms with high C16:0 content in comparison with palms containing low C16:0 content. In OxG, however, palms with low C16:0 content (A1&2_RBL) showed a relatively higher level of *FATB_1* expression (1.29) than the high C16:0 palms (average expression level in A3&4_RBH was 0.97)*.* The qRT-PCR data was generally consistent with that observed for the transcriptome data except for the differential patterns in A1&2_RBL vs. B3&4_RBH which were only observed in the qRT-PCR data (Fig. 4).

The present study also evaluated expression of the candidate isoforms for *FATB_1*. Four putative isoforms were identified and labelled as *FATB_1a* (TCONS_00060749), *_1b* (TCONS_00060752), _*1c* (TCONS_00060750) and *_1d* (TCONS_00060751) (Supplementary Fig. S4). By evaluating the expression profiles of individual putative isoforms (in the transcriptome data), the possibility that the observed combined differential expression of *FATB_1* gene was contributed by the independent isoforms *FATB_1b* (B1&2_UBL vs. B3&4_UBH and C1&2_UBL vs. C3&4_UBH), *FATB_1c* (B1&2_RBL vs. B3&4_RBH) and *FATB_1d* (C1&2_UBL vs. C3&4_UBH) was evaluated. Changes in expression profiles were observed when two (*FATB_1cd*) to three (*FATB_1bcd*) of these isoforms were co-amplified with a common primer-pair (in qRT-PCR experiment). Distinct primer-pairs that could distinguish all isoforms were not possible based on the sequence information available. In *FATB_1cd*, differential expression was observed in C1&2_UBL vs. C3&4_UBH and the profile was similar to that of *FATB_1b* and *FATB_1d* alone*.* Interestingly, significant differential expression was observed for *FATB_1cd* in the A1&2_RBL vs. A3&4_RBH contrast where neither *FATB_1c* nor *FATB_1d* independently had a similar expression profile. The combined expression of *FATB_1cd* was higher in the A3&4_RBH palms relative to A1&2_RBL in OxG and this profile was in contrast to that observed for *FATB_1* at the gene level. Similarly, the profile observed for *FATB_1bcd* in B1&2_UBL vs. B3&4_UBH and B1&2_RBL vs. B3&4_RBH was not similar to that observed for *FATB_1cd* and *FATB_1* (Fig. 4).

**Figure 5:**
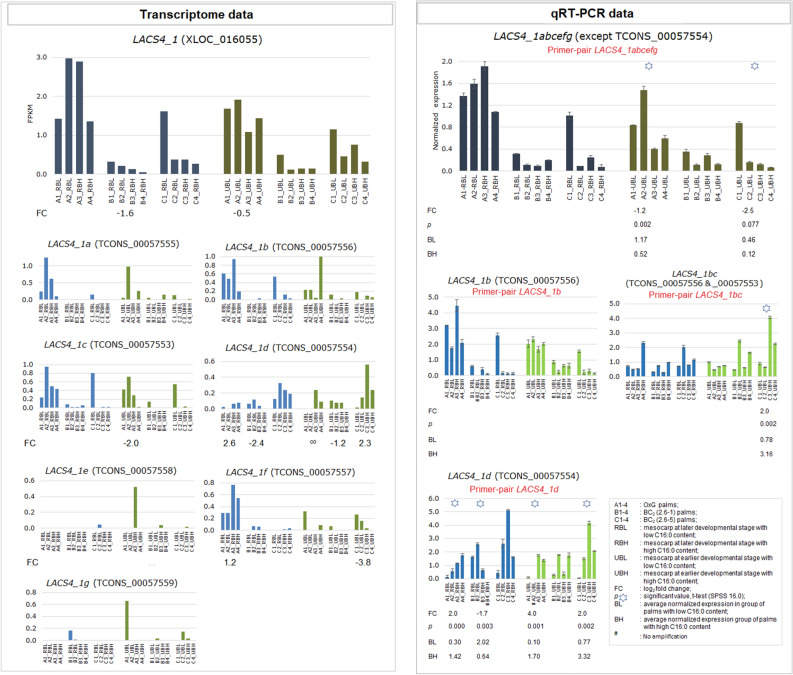
Normalized expression profiles of *LACS4_1* (XLOC_016055) and the isoforms (TCONS_) quantified by qRT-PCR (right panel) in comparison with the transcriptomic expression profiles (left panel). The primer for qRT-PCR denoted by two letters (*i.e*. _bc) was designed to amplify the common exonic region between isoforms TCONS_00057556 and_00057553.

The putative gene *LACS4_1* (XLOC_016055) was identified at position 11,141,682–11,177,588 bp on CHR03. Unlike the other two putative genes of *LACS4*, XLOC_040339 (located in p5_Sc06230) and XLOC_024538 (located in CHR06) which were measured in tens to over hundreds of FPKM, XLOC_016055 (*LACS4_1*) was expressed at a much lower level with FPKM values of no higher than 3.00 (Fig. 5). For *LACS4_1*, a primer-pair labelled as *LACS4_1abcefg* was designed to amplify a common exonic region shared among all the putative isoforms except for *LACS4_1d* (TCONS_00057559). This was because the entire length of the *LACS4_1d* sequence falls outside the range shared by other isoforms (Supplementary Fig. S4). The qRT-PCR result showed significant differential expression between the high- and low-C16:0 palms in OxG at the earlier stages of mesocarp development (A1&2_UBL vs. A3&2_UBH) and the profile was similar to that demonstrated by *LACS4_1* in the transcriptome data. The transcriptomic profile of *LACS4_1* also revealed differential expression between high- and low-C16:0 palms in BC_2_ (2.6–1) (B1&2_RBL vs. B3&4_RBH) but the same profile was not observed in *LACS4_1abcefg*. This discrepancy could be due to exclusion of *LACS4_1d* which may be contributing to the differential expression in B1&2_RBL vs. B3&4_RBH. This was confirmed when the *LACS4_1d* isoform was evaluated separately using qRT-PCR (Fig. 5).

For *LACS4_1* (XLOC_016055), the expression profile of another individual isoform *LACS4_1b* (TCONS_00057556) was also reproducible when evaluated by qRT-PCR although no significant differential expression was identified. However, a significant differential expression profile was detected between high- and low-C16:0 palms in BC_2_ (2.6–5) (C3&4_UBH vs. C1&2_UBL) when this isoform is co-expressed with isoform *LACS4_1c* (TCONS_00057553). The average combined expression level of *LACS4_1bc* in C1&2_UBL was 0.78 whereas it was 3.16 in C3&4_UBH, which corresponds to a FC of 2.0 (Fig. 5).

The predicted protein sequence of all four isoforms of *FATB_1* contained both a functional N-terminal domain of acyl-ATP thioesterase (Acyl-thio_N, PF12590.8) and the acyl-ACP thioesterase domain (Acyl-ACP TE, PF01643.17) (Supplementary Fig. S5). One of the isoforms, *FATB_1a*, had 38 amino acids deleted from Acyl-ACP TE, which could affect the protein function and may explain the low expression levels observed. With respect to *LACS4_1*, truncations were observed at the 5′ site of the AMP-binding domain (PF00501.28) among all the identified isoforms with the exception of *LACS4_1b*. However, other regions in the AMP-binding domain were highly conserved, with *LACS4_1d* being the only isoform where changes in a number of amino acids were observed (Supplementary Fig. S6). This likely explains the different genomic location of *LAC4_1d* from the shared region of the other isoforms (Supplementary Fig. S4). The results suggest that isoforms may be partially functional or may even be non-functional, providing initial evidence that isoform abundance may be a valid area for further investigation. Post-translational modification, protein longevity, localisation and turnover rates may also influence oil synthesis and accumulation.

## Discussion

In many oil producing plants, oil content and the proportion of specific FAs have been associated with the expression of regulatory genes and TFs^43,44^. In oil palm, high level expression of a number of key genes in FA and TAG biosynthesis pathways was also found to be positively correlated with higher oil content in the mesocarp tissue^19–21^. These findings lay a good foundation and suggest that the expression profile of FA and TAG synthesis genes has a strong effect on the observed phenotype, which is the FAC. As such, the present study was aimed at evaluating the expression of these genes, including their putative isoforms and identifying differential expression patterns between palms with high- and low-C16:0 content, the main saturated FA in palm oil.

In this study, there was an acceptable reads-mapping rate up to 88.0%. Furthermore, approximately 98.0% of the mapped reads were unique transcripts although the reference genome used was an *E. guineensis* assembly (EG5.1). A high percentage of the ~ 44 K potential genes was also successfully annotated via RefSeq, Swiss-Prot, GO and the KEGG databases, which gives further confidence in the quality of generated reads and allows for subsequent transcriptome profiling. Most of the genes encoding key enzymes involved in both the FA and TAG synthesis pathways were successfully identified from the main GO terms (GO:0006633, GO:0006636, GO:0008610 and GO:0019432). Therefore, this study has generated a good collection of FA and lipid related genes as well as the putative isoforms for a more in-depth study on specific genes of interest. Only a few genes went undetected such as *pyruvate dehydrogenase* (*PDH*), *FAD3* and *acyl-CoA:lyso-phosphatidylcholine acyltransferase* (*LPCAT*), possibly categorized under other GO terms which were not examined in the present study.

Similar to other plant species^45^, many of the FA genes in oil palm have been reported to be transcriptionally regulated by *WRI1*^18–21^. *WRI1* was one of the key candidate genes present in the QTL region associated with a number of FAs identified in OxG and BC_2_^15^. The more than twofold higher expression of a *WRI1* paralog (XLOC_021657) at the later stage of mesocarp development in conjunction with the increased expression of most of the FA and TAG DEGs listed in Table 3, supports the regulatory mechanism as mentioned above.

Among the FA and TAG genes evaluated, *SAD*, *LACS4*, *FATA* and *FAD2* were found to exhibit extra-high expression levels. Wong et al*.*^46^ made a similar observation reporting that these four genes were among the top six highly expressed genes in the mature mesocarp tissue of *E. guineensis*. Interestingly some of these genes namely *SAD_2* (XLOC_026230/XP_010926734) and *FATA_1* (XLOC_026226/XP_010926746) located on CHR07, as well as *SAD_3* (XLOC_027861/XP_010927705) and *SAD_4* (XLOC_040788/XP_010927705) on CHR08, were also present in previously identified QTL regions associated with various FAC in a BC_1_ mapping population^25^. The CHR07 chromosomal region harbours QTLs associated with C16:1, C18:0, C18:1, C20:0 and IV. The PVE explained by each of the QTLs ranged from 18.3 to 50.2% indicating that the genomic region had a moderate to high effect on the traits concerned. The PVE for C18:0 content was particularly high suggesting that *SAD* and *FATA* play a critical role in regulating FA in the interspecific palms. On CHR08, the QTL was associated with C14:0 where PVE was 10.4%, which indicates that the genomic region has some effect on FAC.

In this study, 12 paralogous genes of *SAD*, *LACS4*, *FATA* and *FAD2* appeared to be significantly differentially expressed both between the two developmental stages as well as when comparing BC_2_ to the OxG genetic background (Table 2). Eight of these paralogs including two for *SAD*, four for *LACS4*, one for each of *FATA* and *FAD2*, showed higher expression levels at the later stages of mesocarp development with FC ranging from 1.08–1.99. For *SAD*, this is in agreement with previous reports on the high desaturase activity of SAD, which facilitates C18:1-ACP accumulation and plays a crucial role in determining the ratio of saturated to unsaturated C18 FAs in membranes and storage lipids in the mesocarp^47,48^. This higher gene expression coincides with the higher rate of FA synthesis and oil accumulation as the fruits mature and ripen after 17/18 WAA.

In addition, comparison of the BC_2_ and OxG genetic backgrounds revealed that all the nine FA (GO:0006633) and TAG (GO:0019432) synthesis DEGs (including *LACS4*, *FATA* and *FAD2*) showed higher expression levels in OxG compared to BC_2_. Considering that 50% of the OxG genome comes from *E. oleifera*, which has higher unsaturated C18 and lower C16:0 content than *E. guineensis*, this suggests a co-regulation system that promotes unsaturated C18 synthesis in the OxG hybrids compared to BC_2_ which has more *E. guineensis* genetic background. *FATA* encodes a thioesterase that terminates de novo FA synthesis by catalyzing the hydrolysis of particularly C18:1-ACP. The higher expression of *FATA_1* at later stage of development (during the oil deposition period) as well as higher expression in OxG hybrids compared to BC_2_ suggest oil palm mesocarp FATA acyl-ACP thioesterase is important not only for oil deposition in the mesocarp but also for the final composition of FAs that are exported from the plastid and enter the storage lipid pool.

FAD2, localized in the ER, is responsible for the synthesis of C18:2 and drives the desaturation of C18:1 in the mesocarp. The concordant higher expression of both *SAD* and *FAD2* in OxG hybrids compared to BC_2_ would facilitate higher accumulation of C18:2 in OxG hybrids. Free FAs released by FATA and FATB are activated by LACS, which forms an important link between FA synthesis in the plastid and TAG assembly in the ER. Dussert et al*.*^19^ reported that *LACS9* and *LACS4-1* were both highly expressed in the oil palm mesocarp but, based on co-expression studies, Guerin et al*.*^20^ suggested that *LACS9* plays the predominant role in the oil palm mesocarp. The current study, however indicates that *LACS4* is more highly expressed, which was also observed in the oil-accumulating avocado mesocarp^49^. It is also interesting that in the current study the paralogs of *LACS4* were more highly expressed at the later stage of development coinciding with higher FA and TAG synthesis while *LACS9* showed higher transcription at the earlier stage of development coinciding with higher membrane lipid synthesis. In *Arabidopsis*, it was shown that LACS4 and LACS9 have overlapping functions and LACS9 is involved in importation of FA into plastid for the biosynthesis of glycolipids^50^. It would be interesting to learn if LACS9 plays a similar role in lipid trafficking from the ER into the plastid in the oil palm mesocarp for membrane lipid synthesis given its higher expression at the earlier stage of development. The significantly higher expression of *LACS4* in the OxG hybrids compared to BC_2_ suggests a more prominent role of this gene in *E. oleifera* compared to *E. guineensis*. The effect of specific genetic backgrounds on gene expression has also been reported previously where many genes encoding PC-related enzymes in the FA and TAG synthesis pathways were found to be more abundantly expressed in the *E. guineensis* intraspecific hybrids (*dura* x *pisifera*) compared to the *dura* mother palm^21^.

Interestingly, *FATB_1* (XLOC_016685) and *LACS4_1* (XLOC_016055) detected in multiple comparisons via late vs. early, BC_2_(2.6–1) vs. OxG, BC_2_(2.6–5) vs. OxG and BC_2_(2.6–5) vs. BC_2_(2.6–1), were also located in a genomic region associated with a major QTL for FAC^15^. Consequently, the expression of the two genes was further evaluated via qRT-PCR. As expected, overall expression profiles revealed by qRT-PCR for the two genes was similar with those observed in the transcriptomic expression data. Unfortunately, significant changes in expression for *FATB_1* and *LACS4_1* were inconsistent across the two different stages of mesocarp development and genetic backgrounds evaluated. For both the BC_2_ crosses, it was observed that *FATB_1* was more abundantly expressed in early stages in palms with high C16:0 content, in comparison with palms containing low C16:0 content. However, at later stages of mesocarp development, the pattern remained consistent in only one of the BC_2_ populations. Furthermore, the expression pattern observed in the OxG background was not consistent with that in the BC_2_ population, where palms with low C16:0 content demonstrated a higher level of expression of *FATB_1* compared to high C16:0-palms. This again suggests the differential expression patterns observed are dependent on the genetic background being investigated. In interspecific hybrid breeding specifically, the expression profile is also likely influenced by the number of backcrossing cycles carried out for the population.

Another possibility is that the different isoforms of a gene influence the expression profile observed and even the activity or functionality of the enzyme produced. For this reason, a number of the putative isoforms for *FATB_1* and *LACS4_1* was evaluated with qRT-PCR. As expected, there was variation in expression profiles demonstrated by the individual isoforms, which clearly suggests their potential contribution towards the final measured expression of a gene. In some cases, the expression profiles of two or more isoforms were investigated using single primer-pair encompassing these isoforms. Even in this case, clear differential expression associated with the various isoforms was evident across the different genetic backgrounds, with high and low levels of C16:0 content. This suggests that different isoforms are expressed at different levels during fruit maturation across different genetic backgrounds.

The results from the present study also suggest that differences in expression of the candidate genes *i.e. FATB_1* and *LACS4_1*, including their isoforms can be generally correlated with the presence of the QTL interval, as determined using conventional QTL mapping of a segregating bi-parental family. Further in-depth studies with a large sample size will surely unravel the role of different isoforms in the FA and oil synthesis regulatory processes and the genes involved. This study demonstrates that evaluating the expression patterns of candidate genes (especially their isoforms) within QTL regions revealed by DNA markers can help to better understand the genetic basis of complex traits in plants. Expression profiles of the specific isoforms of candidate genes like *SAD*, *FATB* and *LACS4* provide another layer of support for the association of the markers defining the QTL regions to FAC in oil palm interspecific hybrids and their backcrosses. However, as pointed out by Azodi et al*.*^51^, genomic regions defined by genetic markers may not capture the entire variation associated with complex traits. Transcriptome analysis in combination with DNA markers using appropriate statistical models is probably required to better decipher the molecular mechanism of complex traits^51^, which is an interesting area for future research in oil palm.

## Supplementary information

Supplementary file1

## Data Availability

The data have been deposited to BioProject accession number PRJNA606124 in the NCBI BioProject database.
