# Supplementary material for: Expression of fatty acid and triacylglycerol synthesis genes in interspecific hybrids of oil palm
Source: Sci Rep. 2020 Oct 1;10:16296. doi: 10.1038/s41598-020-73170-5 (PMC7529811; doi:10.1038/s41598-020-73170-5)
Supplement: Supplementary file 1 — Supplementary file1 [file 41598_2020_73170_MOESM1_ESM.pdf]

Expression of fatty acid and triacylglycerol synthesis genes in interspecific hybrids of oil palm

Ngoot-Chin Ting[1,2,8], Katrina Sherbina[3,8], Jia-Shiun Khoo[4], Katialisa Kamaruddin[1], Pek-Lan Chan[1], Kuang-Lim Chan[1], Mohd Amin Ab Halim[1], Kandha Sritharan[5], Zulkifli Yaakub[1], Sean Mayes[6], Festo Massawe[2], Peter L. Chang[7], Sergey V. Nuzhdin[7], Ravigadevi Sambanthamurthi[1], Rajinder Singh[1*]

[1]Advanced Biotechnology and Breeding Centre, Malaysian Palm Oil Board (MPOB), P.O. Box 10620, 50720 Kuala Lumpur, Malaysia. [2]Biotechnology Research Centre, School of Biosciences, University of Nottingham Malaysia Campus, Jalan Broga, 43500 Semenyih, Selangor, Malaysia. [3]Quantitative and Computational Biology Section, University of Southern California, 1050 Childs Way, Los Angeles, CA, USA. [4]Codon Genomics Sdn. Bhd. Malaysia, No. 26, Jalan Dutamas 7, Taman Dutamas, Balakong, 43200, Seri Kembangan, Selangor, Malaysia. [5]United Plantations Bhd., Jendarata Estate, 36009 Teluk Intan, Perak, Malaysia. [6]Plant and Crop Sciences, Sutton Bonington Campus, University of Nottingham, Sutton Bonington, Loughborough LE12 5RD, UK. [7]Molecular and Computational Biology Section, University of Southern California, 1050 Childs Way, Los Angeles, CA, USA. [8] These authors contributed equally: Ngoot-Chin Ting, Katrina Sherbina.
[*]Corresponding author email: rajinder@mpob.gov.my

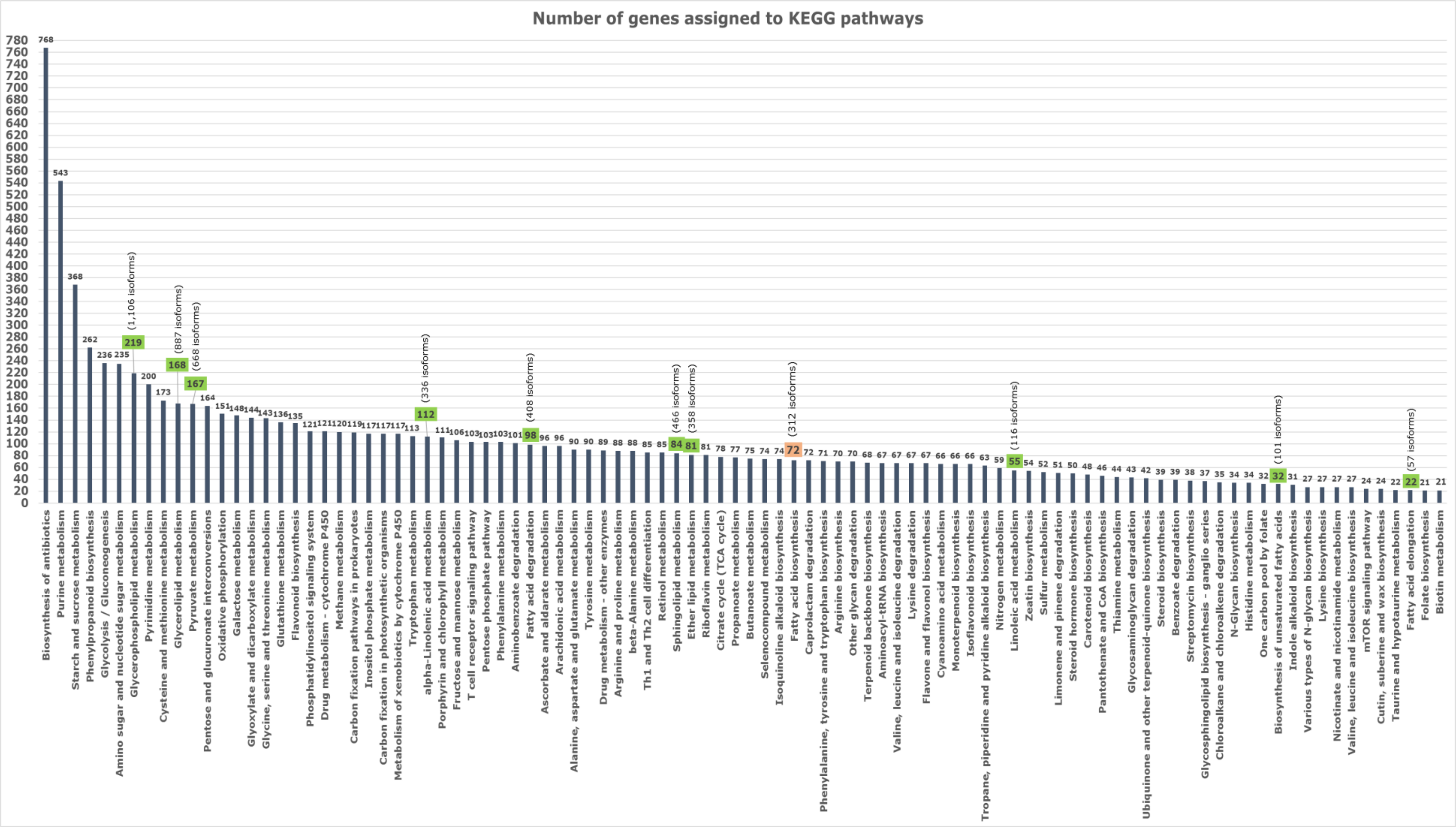

Supplementary Figure S1. Number of putative genes and isoforms mapped to the KEGG pathways. Only pathways with gene numbers > 20 are shown.

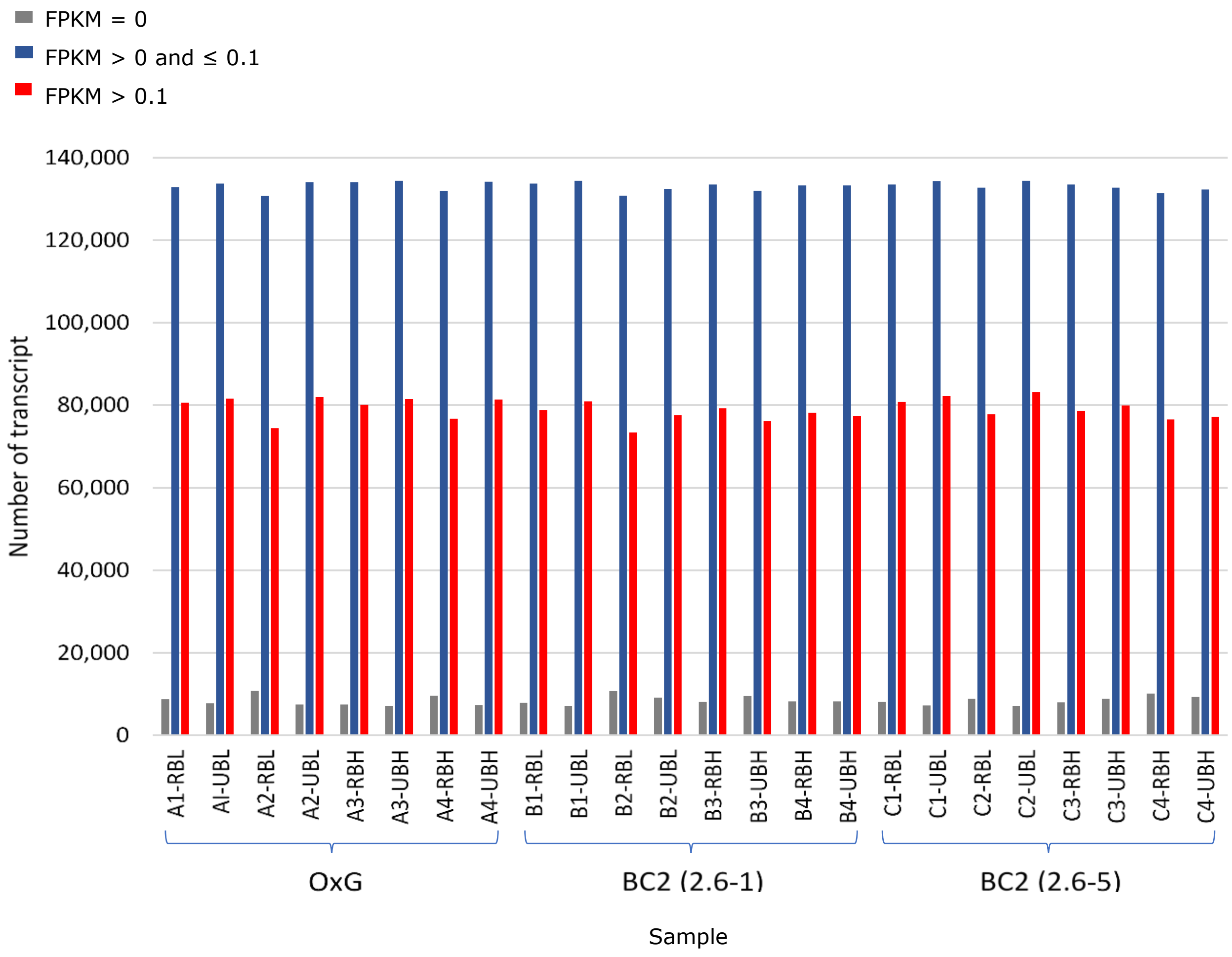

Supplementary Figure S2. Expression of transcripts across all samples from three genetic backgrounds.

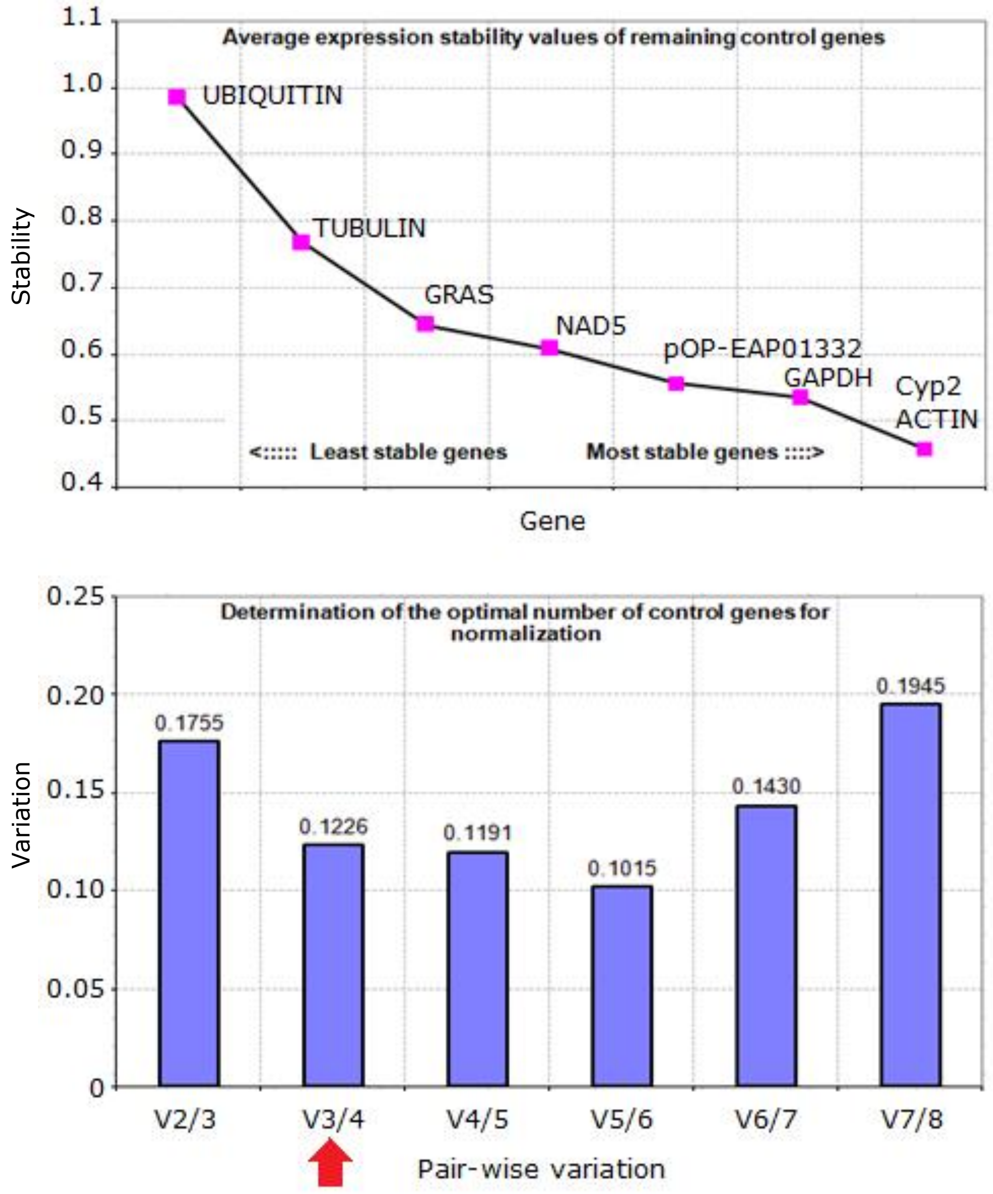

Supplementary Figure S3. Screening and selection of an optimal set of the most stably expressed reference genes.

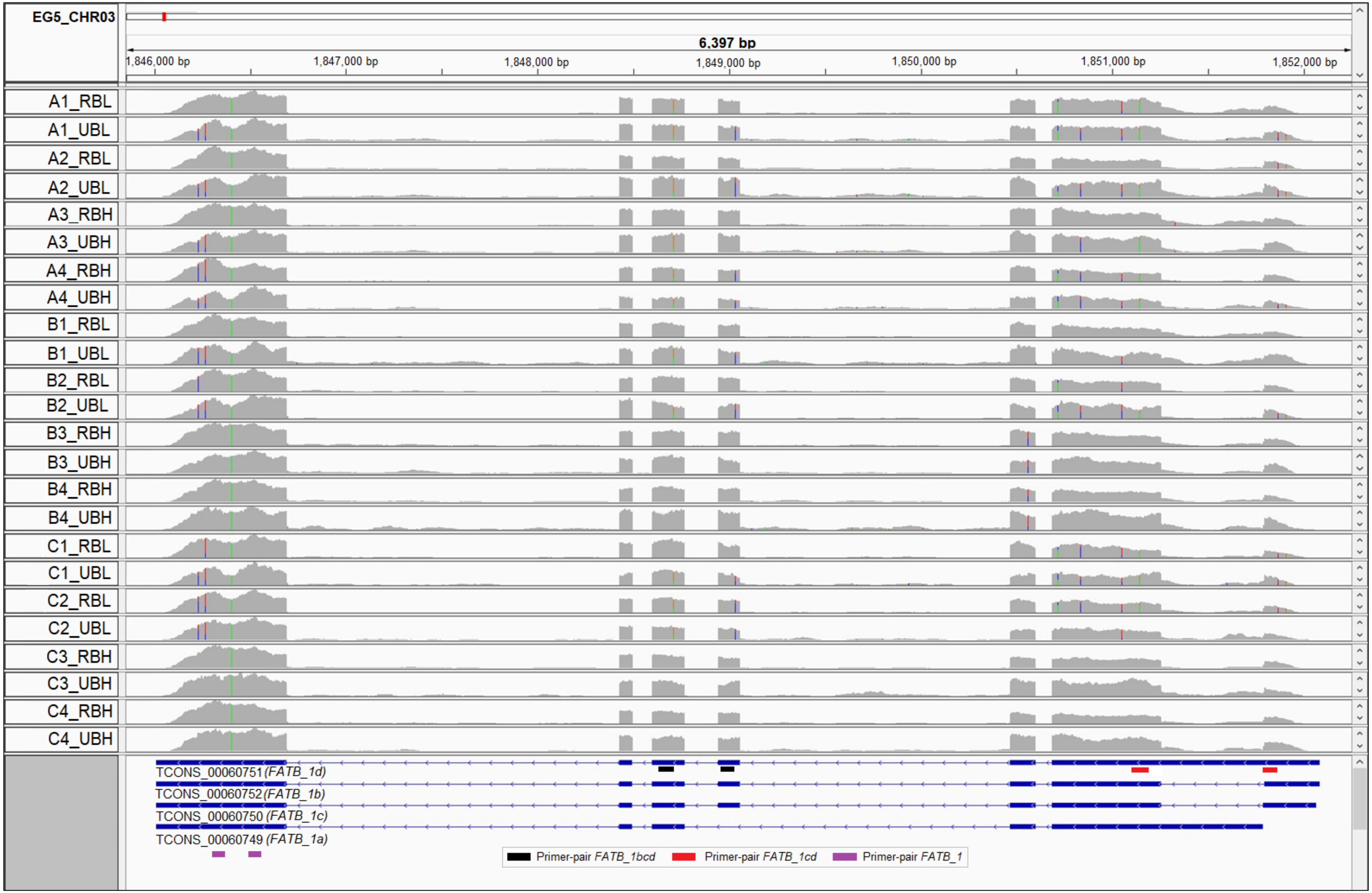
EG5_CHR03
6,397 bp
1,846,000 bp
1,847,000 bp
1,848,000 bp
1,849,000 bp
1,850,000 bp
1,851,000 bp
1,852,000 bp
A1_RBL
A1_UBL
A2_RBL
A2_UBL
A3_RBH
A3_UBH
A4_RBH
A4_UBH
B1_RBL
B1_UBL
B2_RBL
B2_UBL
B3_RBH
B3_UBH
B4_RBH
B4_UBH
C1_RBL
C1_UBL
C2_RBL
C2_UBL
C3_RBH
C3_UBH
C4_RBH
C4_UBH
TCONS_00060751 (FATB_1d)
TCONS_00060752 (FATB_1b)
TCONS_00060750 (FATB_1c)
TCONS_00060749 (FATB_1a)
Primer-pair FATB_1bcd
Primer-pair FATB_1cd
Primer-pair FATB_1

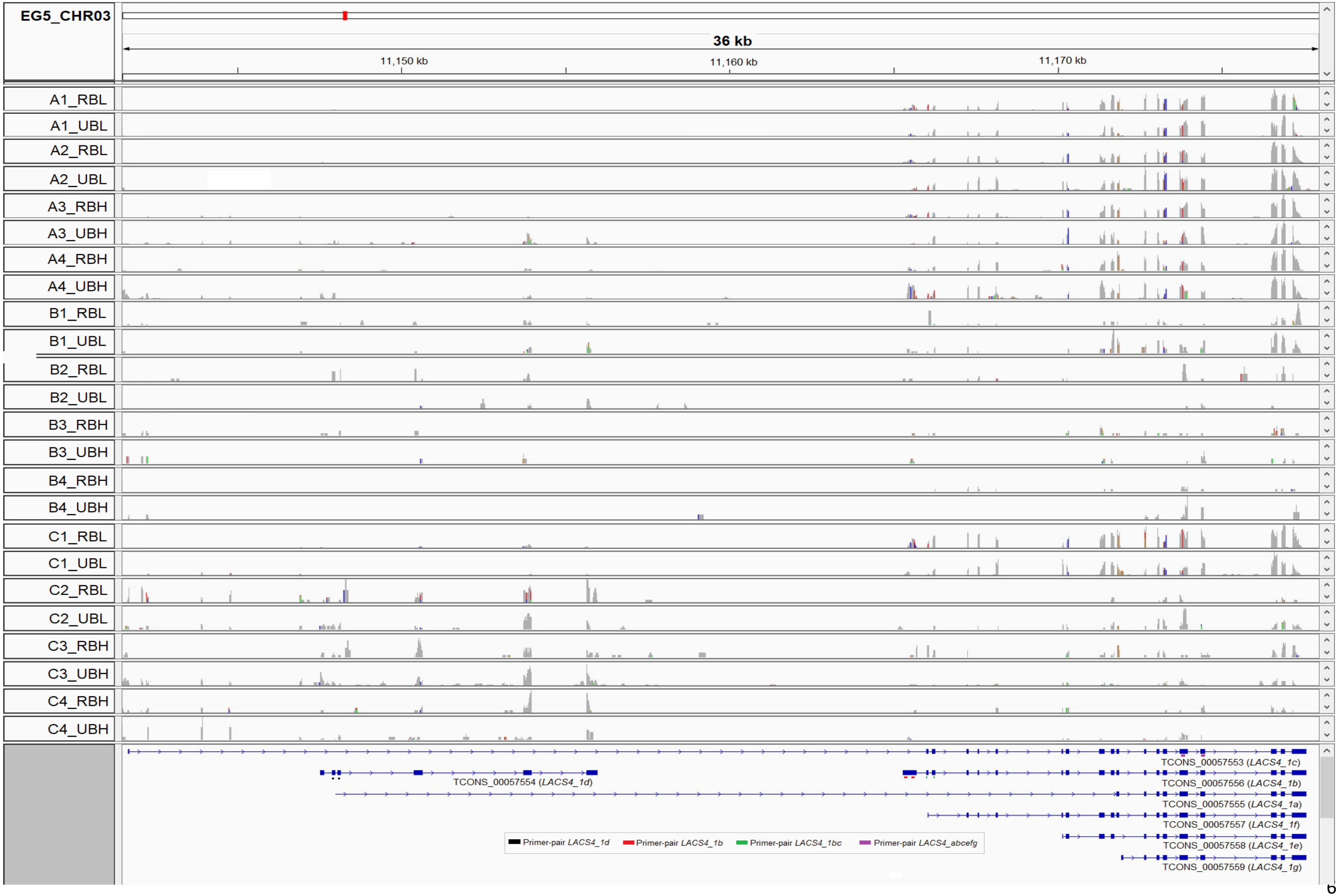
EG5_CHR03
36 kb
11,150 kb
11,160 kb
11,170 kb
A1_RBL
A1_UBL
A2_RBL
A2_UBL
A3_RBH
A3_UBH
A4_RBH
A4_UBH
B1_RBL
B1_UBL
B2_RBL
B2_UBL
B3_RBH
B3_UBH
B4_RBH
B4_UBH
C1_RBL
C1_UBL
C2_RBL
C2_UBL
C3_RBH
C3_UBH
C4_RBH
C4_UBH
TCONS_00057553 (LACS4_1c)
TCONS_00057554 (LACS4_1d)
TCONS_00057556 (LACS4_1b)
TCONS_00057555 (LACS4_1a)
TCONS_00057557 (LACS4_1f)
TCONS_00057558 (LACS4_1e)
TCONS_00057559 (LACS4_1g)
Primer-pair LACS4_1d
Primer-pair LACS4_1b
Primer-pair LACS4_1bc
Primer-pair LACS4_abcefg

Supplementary Figure S4. Mapping of putative isoforms of *FATB_1* (XLOC_016685) (top panel) and *LACS4_1* (XLOC_016055) (bottom panel) to the EG5 reference genome using the Integrative genomics viewer (IGV 2.4.2)[52]. Four putative isoforms of *FATB_1* are labelled as TCONS_00060749 (*FATB_1a*), TCONS_00060752 (*FATB_1b*), TCONS_00060750 (*FATB_1c*) and TCONS_00060751 (*FATB_1d*). For *LACS4_1*, seven identified putative isoforms were TCONS_00057555 (*LACS4_1a*), TCONS_00057556 (*LACS4_1b*), TCONS_00057553 (*LACS4_1c*), TCONS_00057554 (*LACS4_1d*), TCONS_00057558 (*LACS4_1e*), TCONS_00057557 (*LACS4_1f*) and TCONS_00057559 (*LACS4_1g*).

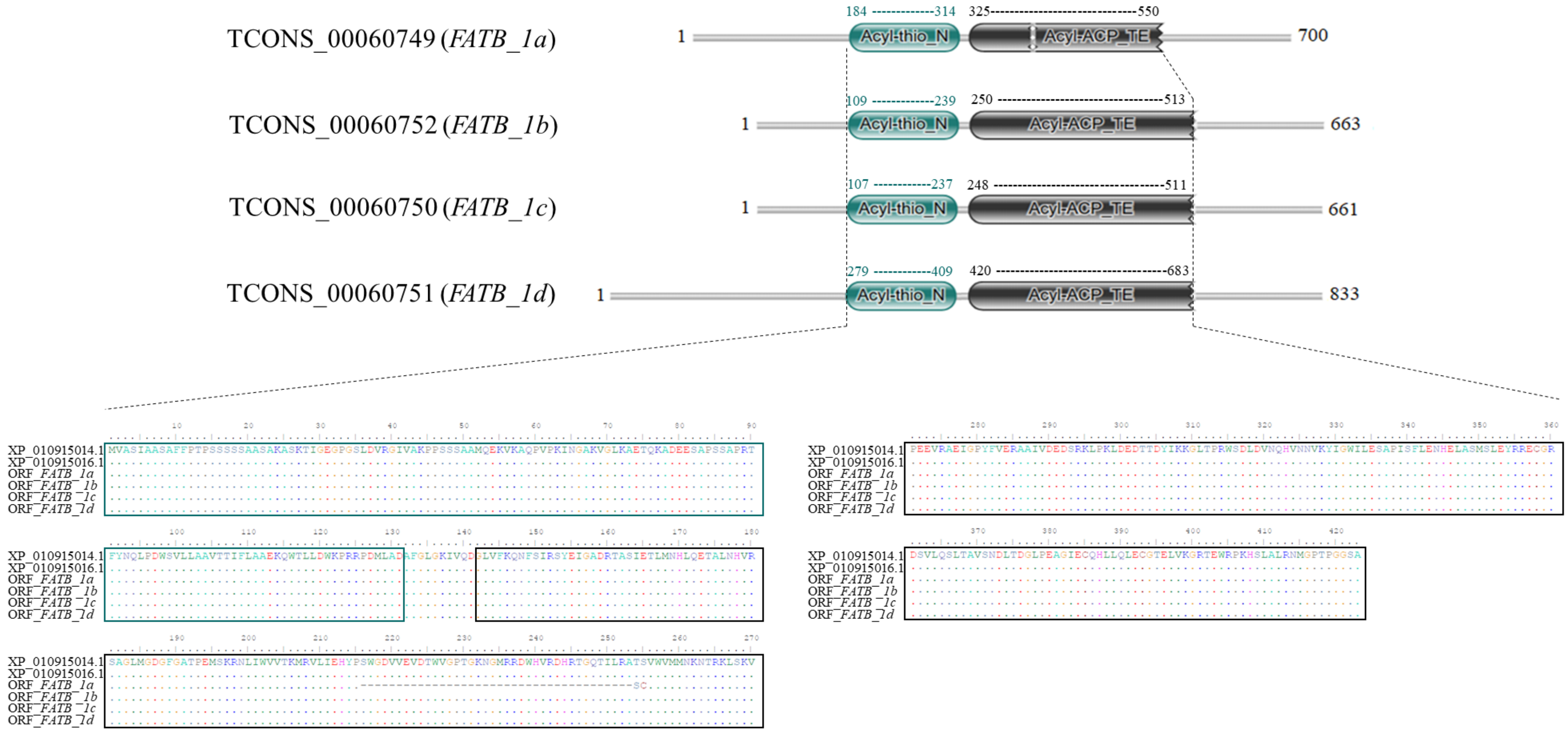

Supplementary Figure S5. Alignment of functional domains among the *FATB_1* isoforms using the HMMER webserver (http://www.ebi.ac.uk/Tools/hmmer)[53]. Protein sequences of two *E. guineensis FATB* isoforms X1 (XP_010915014.1) and X2 (XP_010915016.1) obtained from NCBI database were also included for comparison.

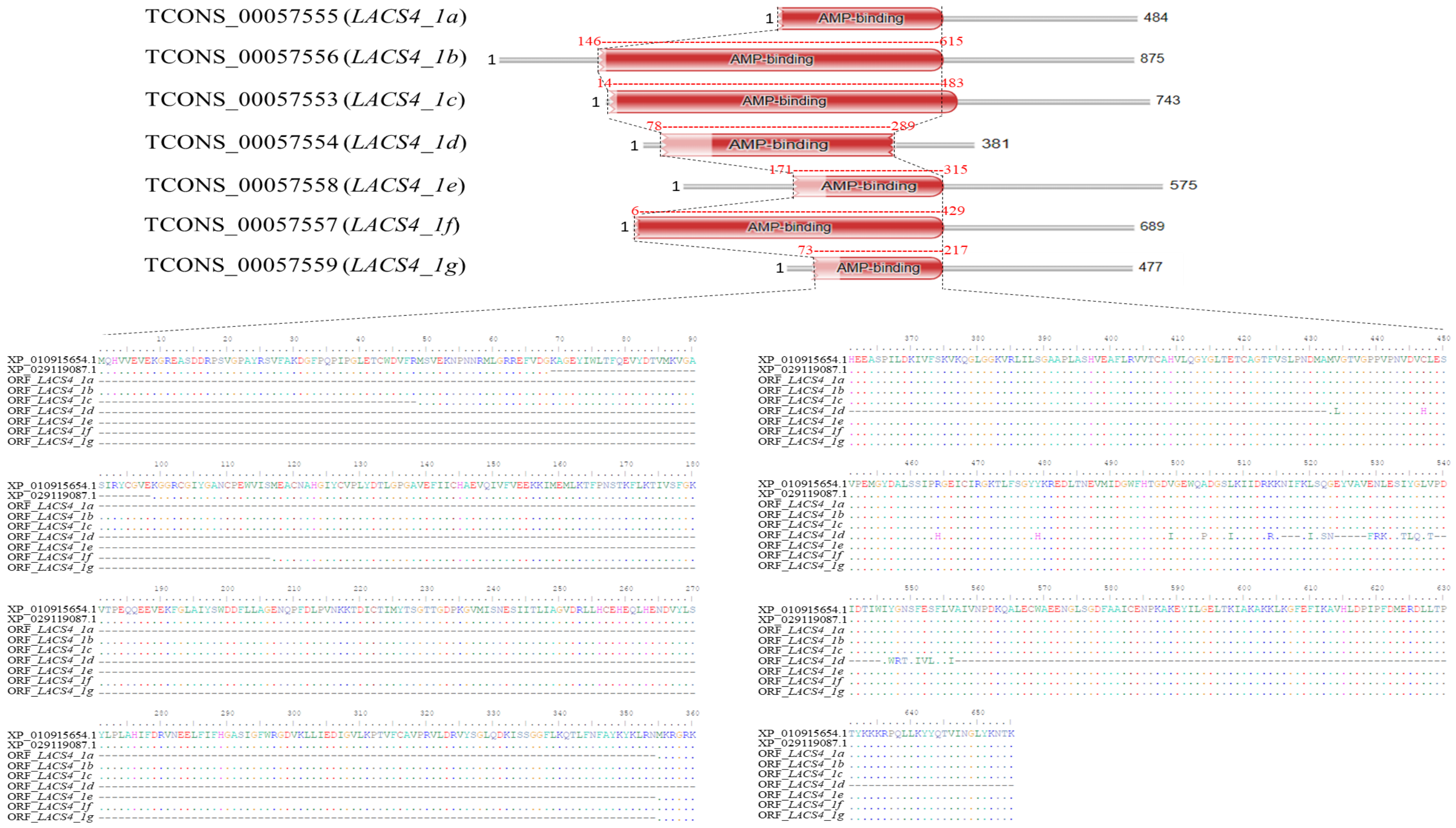

Supplementary Figure S6. Alignment of functional domains among the *LACS4_1* isoforms using the HMMER webserver (http://www.ebi.ac.uk/Tools/hmmer)[53]. Protein sequences of two *E. guineensis LACS4* isoforms X1 (XP_010915654.1) and X2 (XP_029119087.1) obtained from NCBI database were also included for comparison.

No.	Palm	Palmitic acid (C16:0 %)	Oleic acid (C18:1 %)	Iodine value (IV)	Cross	Earlier stage of mesocarp development		Later stage of mesocarp development	
						Labelled as	Estimated WAA	Labelled as	Estimated WAA
1	A1	Low (22.2)	High (61.5)	High (77.3)	OxG	A1_UBL	16–17	A1_RBL	20
2	A2	Low (26.0)	High (59.5)	High (70.2)	OxG	A2_UBL	16–17	A2_RBL	21–22
3	A3	High (34.3)	Low (49.0)	Low (67.4)	OxG	A3_UBH	15	A3_RBH	20–21
4	A4	High (34.1)	Low (49.0)	Low (67.2)	OxG	A4_UBH	15–16	A4_RBH	19–20
5	B1	Low (24.7)	High (57.2)	High (69.2)	BC_2 (2.6-1)	B1_UBL	11 – 12	B1_RBL	20
6	B2	Low (27.2)	High (53.7)	High (69.2)	BC_2 (2.6-1)	B2_UBL	15 – 16	B2_RBL	20
7	B3	High (33.4)	Low (45.4)	Low (61.9)	BC_2 (2.6-1)	B3_UBH	16 – 17	B3_RBH	20
8	B4	High (33.1)	Low (45.0)	Low (61.4)	BC_2 (2.6-1)	B4_UBH	15 – 16	B4_RBH	20
9	C1	Low (28.9)	High (53.3)	High (69.6)	BC_2 (2.6-5)	C1_UBL	12 – 13	C1_RBL	18–19
10	C2	Low (26.9)	High (53.7)	High (69.6)	BC_2 (2.6-5)	C2_UBL	11	C2_RBL	19–20
11	C3	High (40.6)	Low (37.6)	Low (60.3)	BC_2 (2.6-5)	C3_UBH	15 – 16	C3_RBH	20–21
12	C4	High (38.9)	Low (45.5)	Low (60.5)	BC_2 (2.6-5)	C4_UBH	16	C4_RBH	21

Supplementary Table S1. Phenotypic data for the 12 palms from three genetic backgrounds selected for RNA sequencing.

No.	GO term (A)	Description	No. of gene	No. of isoforms
1	**GO:0006633**	**fatty acid biosynthetic process**	**160**	**588**
2	**GO:0006631**	**fatty acid metabolic process**	**60**	**220**
3	**GO:0009062**	**fatty acid catabolic process**	**8**	**37**
4	GO:0042967	acyl-carrier-protein biosynthetic process	375	1,965
5	GO:0006090	pyruvate metabolic process	76	332
6	GO:0042761	very long-chain fatty acid biosynthetic process	78	270
7	GO:0006635	fatty acid beta-oxidation	39	234
8	GO:0036109	alpha-linolenic acid metabolic process	17	131
9	GO:0006637	acyl-CoA metabolic process	24	122
10	GO:0032000	positive regulation of fatty acid beta-oxidation	11	112
11	GO:0006636	unsaturated fatty acid biosynthetic process	41	107
12	GO:0015908	fatty acid transport	22	100
13	GO:0006107	oxaloacetate metabolic process	23	98
14	GO:0035338	long-chain fatty-acyl-CoA biosynthetic process	14	93
15	GO:0042759	long-chain fatty acid biosynthetic process	25	87
16	GO:0033539	fatty acid beta-oxidation using acyl-CoA dehydrogenase	11	83
17	GO:0033540	fatty acid beta-oxidation using acyl-CoA oxidase	9	79
18	GO:0010747	positive regulation of plasma membrane long-chain fatty acid transport	11	76
19	GO:0045723	positive regulation of fatty acid biosynthetic process	12	75
20	GO:0044539	long-chain fatty acid import	11	75
21	GO:0071398	cellular response to fatty acid	15	70
22	GO:0035336	long-chain fatty-acyl-CoA metabolic process	22	69
23	GO:0000038	very long-chain fatty acid metabolic process	27	68
24	GO:2001295	malonyl-CoA biosynthetic process	11	68
25	GO:0055089	fatty acid homeostasis	6	65
26	GO:0015916	fatty-acyl-CoA transport	3	55
27	GO:0030497	fatty acid elongation	19	55
28	GO:0015937	coenzyme A biosynthetic process	11	54
29	GO:0015910	peroxisomal long-chain fatty acid import	3	52
30	GO:0042758	long-chain fatty acid catabolic process	3	52
31	GO:0042760	very long-chain fatty acid catabolic process	3	52
32	GO:0006084	acetyl-CoA metabolic process	6	40
33	GO:0031998	regulation of fatty acid beta-oxidation	9	39
34	GO:0015936	coenzyme A metabolic process	8	37
35	GO:0051792	medium-chain fatty acid biosynthetic process	9	36
36	GO:0051790	short-chain fatty acid biosynthetic process	7	34
37	GO:0001561	fatty acid alpha-oxidation	11	33
38	GO:0019395	fatty acid oxidation	15	33
39	GO:0001676	long-chain fatty acid metabolic process	8	31
40	GO:0019217	regulation of fatty acid metabolic process	3	31
41	GO:0045717	negative regulation of fatty acid biosynthetic process	7	31
42	GO:0070542	response to fatty acid	4	27
43	GO:0006083	acetate metabolic process	17	24
44	GO:0019626	short-chain fatty acid catabolic process	2	24
45	GO:0006086	acetyl-CoA biosynthetic process from pyruvate	8	23
46	GO:0051791	medium-chain fatty acid metabolic process	4	22
47	GO:0006085	acetyl-CoA biosynthetic process	6	20
48	GO:0042866	pyruvate biosynthetic process	3	20
49	GO:0033559	unsaturated fatty acid metabolic process	7	17
50	GO:0046167	glycerol-3-phosphate biosynthetic process	5	17
51	GO:0010510	regulation of acetyl-CoA biosynthetic process from pyruvate	6	16
52	GO:0046166	glyceraldehyde-3-phosphate biosynthetic process	5	16
53	GO:0042304	regulation of fatty acid biosynthetic process	5	15
54	GO:0019413	acetate biosynthetic process	3	14
55	GO:0051156	glucose 6-phosphate metabolic process	3	11
56	GO:0046459	short-chain fatty acid metabolic process	3	10

57	GO:2000191	regulation of fatty acid transport	1	10
58	GO:0031999	negative regulation of fatty acid beta-oxidation	1	9
59	GO:0019427	acetyl-CoA biosynthetic process from acetate	2	8
60	GO:0010430	fatty acid omega-oxidation	3	8
61	GO:0009108	coenzyme biosynthetic process	1	6
62	GO:0046949	fatty-acyl-CoA biosynthetic process	3	5
63	GO:0080020	regulation of coenzyme A biosynthetic process	1	5
64	GO:0019367	fatty acid elongation, saturated fatty acid	4	4
65	GO:0046322	negative regulation of fatty acid oxidation	2	4
66	GO:0036111	very long-chain fatty-acyl-CoA metabolic process	1	3
67	GO:0036112	medium-chain fatty-acyl-CoA metabolic process	1	3
68	GO:0015909	long-chain fatty acid transport	1	3
69	GO:0015911	plasma membrane long-chain fatty acid transport	1	3
70	GO:0033542	fatty acid beta-oxidation, unsaturated, even number	1	3
71	GO:0034625	fatty acid elongation, monounsaturated fatty acid	3	3
72	GO:0034626	fatty acid elongation, polyunsaturated fatty acid	3	3
73	GO:0046320	regulation of fatty acid oxidation	1	3
74	GO:0051793	medium-chain fatty acid catabolic process	1	3
75	GO:0046356	acetyl-CoA catabolic process	1	3

No.	GO term (B)	Description	No. of gene	No. of isoforms
1	**GO:0019432**	**triglyceride biosynthetic process**	**38**	**202**
2	**GO:0006641**	**triglyceride metabolic process**	**4**	**32**
3	**GO:0019433**	**triglyceride catabolic process**	**17**	**42**
4	GO:0046486	glycerolipid metabolic process	186	903
5	GO:0006687	glycosphingolipid metabolic process	83	531
6	GO:0006629	lipid metabolic process	92	417
7	GO:0010025	wax biosynthetic process	63	280
8	GO:0044255	cellular lipid metabolic process	31	221
9	GO:0034389	lipid particle organization	32	183
10	GO:0008654	phospholipid biosynthetic process	37	169
11	GO:0010867	positive regulation of triglyceride biosynthetic process	15	141
12	GO:0008610	lipid biosynthetic process	36	129
13	GO:0019375	galactolipid biosynthetic process	17	117
14	GO:0030148	sphingolipid biosynthetic process	23	112
15	GO:0009245	lipid A biosynthetic process	12	90
16	GO:0016024	CDP-diacylglycerol biosynthetic process	12	89
17	GO:2001289	lipid X metabolic process	6	85
18	GO:0006650	glycerophospholipid metabolic process	9	75
19	GO:0006665	sphingolipid metabolic process	13	75
20	GO:0046890	regulation of lipid biosynthetic process	8	71
21	GO:0034197	triglyceride transport	9	70
22	GO:0009247	glycolipid biosynthetic process	15	62
23	GO:0046474	glycerophospholipid biosynthetic process	11	52
24	GO:0006642	triglyceride mobilization	11	50
25	GO:0046339	diacylglycerol metabolic process	6	41
26	GO:0006655	phosphatidylglycerol biosynthetic process	7	39
27	GO:0046464	acylglycerol catabolic process	10	30
28	GO:0046889	positive regulation of lipid biosynthetic process	2	29
29	GO:0071071	regulation of phospholipid biosynthetic process	7	29
30	GO:0006643	membrane lipid metabolic process	4	28
31	GO:0019216	regulation of lipid metabolic process	10	26
32	GO:0036104	Kdo2-lipid A biosynthetic process	1	25
33	GO:0034014	response to triglyceride	3	24
34	GO:0090157	negative regulation of sphingolipid biosynthesis involved in cellular sphingolipid homeostasis	2	21
35	GO:0030259	lipid glycosylation	2	18
36	GO:0006644	phospholipid metabolic process	5	16
37	GO:0019374	galactolipid metabolic process	8	16
38	GO:0030258	lipid modification	5	16

39	GO:0090207	regulation of triglyceride metabolic process	2	15
40	GO:0044241	lipid digestion	2	13
41	GO:0046466	membrane lipid catabolic process	3	13
42	GO:0010883	regulation of lipid storage	2	12
43	GO:0006651	diacylglycerol biosynthetic process	4	11
44	GO:0030149	sphingolipid catabolic process	1	11
45	GO:0042158	lipoprotein biosynthetic process	3	10
46	GO:0051055	negative regulation of lipid biosynthetic process	2	10
47	GO:0010884	positive regulation of lipid storage	1	9
48	GO:0045017	glycerolipid biosynthetic process	1	8
49	GO:0010888	negative regulation of lipid storage	2	8
50	GO:0046475	glycerophospholipid catabolic process	3	7
51	GO:0010868	negative regulation of triglyceride biosynthetic process	1	6
52	GO:0009106	lipoate metabolic process	2	3
53	GO:0071073	positive regulation of phospholipid biosynthetic process	1	3
54	GO:0060588	negative regulation of lipoprotein lipid oxidation	2	3
55	GO:2001140	positive regulation of phospholipid transport	3	3
56	GO:0090155	negative regulation of sphingolipid biosynthetic process	1	2
57	GO:0048017	inositol lipid-mediated signaling	1	2
58	GO:0045834	positive regulation of lipid metabolic process	1	1
59	GO:0046834	lipid phosphorylation	1	1
60	GO:0030730	sequestering of triglyceride	1	1
61	GO:0070328	triglyceride homeostasis	1	1

**No.**	**GO term (C)**	**Description**	**No. of gene**	**No. of isoforms**
1	GO:0009651	response to salt stress	1,350	4,766
2	GO:0009069	serine family amino acid metabolic process	1,238	5,628
3	GO:0009737	response to abscisic acid stimulus	1,166	4,219
4	GO:0006355	regulation of transcription, DNA-dependent	1,061	4,381
5	GO:0009414	response to water deprivation	1,021	3,475
6	GO:0055114	oxidation-reduction process	1,007	3,784
7	GO:0090304	nucleic acid metabolic process	981	3,965
8	GO:0046686	response to cadmium ion	933	3,405
9	GO:0009793	embryo development ending in seed dormancy	928	3,764
10	GO:0042742	defense response to bacterium	888	3,397
11	GO:0046777	protein autophosphorylation	848	3,754
12	GO:0045893	positive regulation of transcription, DNA-dependent	827	3,053
13	GO:0009611	response to wounding	820	2,864
14	GO:0050832	defense response to fungus	806	2,672
15	GO:0009409	response to cold	724	2,428
16	GO:0044260	cellular macromolecule metabolic process	657	2,736
17	GO:0009733	response to auxin stimulus	648	2,210
18	GO:0009555	pollen development	629	2,407
19	GO:0045892	negative regulation of transcription, DNA-dependent	613	2,352
20	GO:0044238	primary metabolic process	609	2,188
21	GO:0009751	response to salicylic acid stimulus	560	1,613
22	GO:0006259	DNA metabolic process	548	2,348
23	GO:0010200	response to chitin	508	1,274
24	GO:0007165	signal transduction	499	1,958
25	GO:0006952	defense response	496	1,381
26	GO:0006979	response to oxidative stress	475	1,890
27	GO:0010150	leaf senescence	470	1,885
28	GO:0009735	response to cytokinin stimulus	468	1,554
29	GO:0005985	sucrose metabolic process	462	1,915
30	GO:0006468	protein phosphorylation	450	1,676
31	GO:0043170	macromolecule metabolic process	432	1,482
32	GO:0048364	root development	424	1,810
33	GO:0051301	cell division	424	1,664
34	GO:0030154	cell differentiation	419	1,579
35	GO:0009753	response to jasmonic acid stimulus	418	1,479

36	GO:0005982	starch metabolic process	412	1,685
37	GO:0080167	response to karrikin	403	1,125
38	GO:0016567	protein ubiquitination	392	1,582
39	GO:0009723	response to ethylene stimulus	388	1,314
40	GO:0009408	response to heat	378	1,404
41	GO:0007178	transmembrane receptor protein serine/threonine kinase signaling pathway	377	2,065
42	GO:0006118	electron transport	369	1,579
43	GO:0048366	leaf development	368	1,476
44	GO:0006351	transcription, DNA-dependent	367	1,651
45	GO:0006810	transport	363	1,424
46	GO:0009826	unidimensional cell growth	356	1,224
47	GO:0009908	flower development	356	1,497
48	GO:0080156	mitochondrial mRNA modification	354	1,058
49	GO:0009624	response to nematode	351	1,524
50	GO:0009987	cellular process	349	1,283
51	GO:0009860	pollen tube growth	343	1,603
52	GO:0009617	response to bacterium	342	965
53	GO:0016310	phosphorylation	336	1,352
54	GO:0009620	response to fungus	335	1,363
55	GO:0009416	response to light stimulus	334	1,171
56	GO:0006144	purine nucleobase metabolic process	332	1,401
57	GO:0050794	regulation of cellular process	330	1,315
58	GO:0007275	multicellular organismal development	315	1,239
59	GO:0009739	response to gibberellin stimulus	309	940
60	GO:0007623	circadian rhythm	308	1,537
61	GO:0010224	response to UV-B	307	844
62	GO:0000184	nuclear-transcribed mRNA catabolic process, nonsense-mediated decay	303	894
63	GO:0006950	response to stress	300	1,225
64	GO:0048527	lateral root development	300	908
65	GO:1900865	chloroplast RNA modification	298	860
66	GO:0009845	seed germination	297	1,215
67	GO:0042254	ribosome biogenesis	291	1,146
68	GO:0009626	plant-type hypersensitive response	290	929
69	GO:0045944	positive regulation of transcription from RNA polymerase II promoter	287	1,487
70	GO:0006412	translation	285	1,012
71	GO:0006364	rRNA processing	284	890
72	GO:0009738	abscisic acid mediated signaling pathway	284	1,231
73	GO:0042542	response to hydrogen peroxide	280	1,041
74	GO:0010228	vegetative to reproductive phase transition of meristem	277	1,086
75	GO:0006470	protein dephosphorylation	275	1,352
76	GO:0006094	gluconeogenesis	264	958
77	GO:0006508	proteolysis	263	1,241
78	GO:0000122	negative regulation of transcription from RNA polymerase II promoter	262	1,406
79	GO:0006413	translational initiation	255	597
80	GO:0031425	chloroplast RNA processing	255	758
81	GO:0009791	post-embryonic development	254	1,016
82	GO:0006357	regulation of transcription from RNA polymerase II promoter	250	1,179
83	GO:0010305	leaf vascular tissue pattern formation	250	950
84	GO:0015031	protein transport	250	1,184
85	GO:0006614	SRP-dependent cotranslational protein targeting to membrane	247	556
86	GO:0009867	jasmonic acid mediated signaling pathway	245	889
87	GO:0042538	hyperosmotic salinity response	245	759
88	GO:0009809	lignin biosynthetic process	244	658
89	GO:0019083	viral transcription	244	554
90	GO:0006457	protein folding	241	915

91	GO:0016554	cytidine to uridine editing	238	657
92	GO:0010051	xylem and phloem pattern formation	237	970
93	GO:0007169	transmembrane receptor protein tyrosine kinase signaling pathway	232	876
94	GO:0009742	brassinosteroid mediated signaling pathway	232	874
95	GO:0009734	auxin mediated signaling pathway	231	915
96	GO:0010029	regulation of seed germination	230	753
97	GO:0035556	intracellular signal transduction	230	1,081
98	GO:0008283	cell proliferation	229	938
99	GO:0006397	mRNA processing	226	1,201
100	GO:0009658	chloroplast organization	226	1,086
101	GO:0001666	response to hypoxia	223	1,089
102	GO:0016043	cellular component organization	223	711
103	GO:0009628	response to abiotic stimulus	221	739
104	GO:0009813	flavonoid biosynthetic process	219	460
105	GO:0006096	glycolysis	217	767
106	GO:0002229	defense response to oomycetes	216	754
107	GO:0048767	root hair elongation	215	947
108	GO:0019760	glucosinolate metabolic process	214	532
109	GO:0048653	anther development	212	561
110	GO:0009965	leaf morphogenesis	211	817
111	GO:0009873	ethylene mediated signaling pathway	208	690
112	GO:0006886	intracellular protein transport	207	946
113	GO:0009816	defense response to bacterium, incompatible interaction	206	994
114	GO:0010119	regulation of stomatal movement	205	918
115	GO:0040007	growth	205	787
116	GO:0009792	embryo development ending in birth or egg hatching	202	759
117	GO:0010087	phloem or xylem histogenesis	202	800
118	GO:0010229	inflorescence development	202	715
119	GO:0050896	response to stimulus	198	684
120	GO:0010468	regulation of gene expression	196	679
121	GO:0009834	secondary cell wall biogenesis	195	599
122	GO:0048316	seed development	193	746
123	GO:0002181	cytoplasmic translation	191	497
124	GO:0009909	regulation of flower development	190	797
125	GO:0010218	response to far red light	190	830
126	GO:0010311	lateral root formation	190	816
127	GO:0055085	transmembrane transport	190	843
128	GO:0008284	positive regulation of cell proliferation	188	786
129	GO:0009395	phospholipid catabolic process	187	824
130	GO:0016070	RNA metabolic process	187	754
131	GO:0043161	proteasomal ubiquitin-dependent protein catabolic process	186	866
132	GO:0048367	shoot development	186	814
133	GO:0001944	vasculature development	185	500
134	GO:0048510	regulation of timing of transition from vegetative to reproductive phase	185	744
135	GO:0009644	response to high light intensity	184	470
136	GO:0016036	cellular response to phosphate starvation	183	799
137	GO:0009846	pollen germination	182	931
138	GO:0010588	cotyledon vascular tissue pattern formation	182	596
139	GO:0051865	protein autoubiquitination	181	675
140	GO:0009911	positive regulation of flower development	179	685
141	GO:0071555	cell wall organization	178	598
142	GO:0043066	negative regulation of apoptotic process	176	831
143	GO:0010223	secondary shoot formation	174	486
144	GO:0044237	cellular metabolic process	174	649
145	GO:0009744	response to sucrose stimulus	172	701
146	GO:0009805	coumarin biosynthetic process	171	494
147	GO:0000209	protein polyubiquitination	170	600
148	GO:0009640	photomorphogenesis	170	650

149	GO:0000398	nuclear mRNA splicing, via spliceosome	169	802
150	GO:0009725	response to hormone stimulus	168	613
151	GO:0010183	pollen tube guidance	167	724
152	GO:0009653	anatomical structure morphogenesis	166	727
153	GO:0016032	viral reproduction	165	675
154	GO:0071456	cellular response to hypoxia	165	486
155	GO:0090305	nucleic acid phosphodiester bond hydrolysis	165	699
156	GO:0006281	DNA repair	163	834
157	GO:0009741	response to brassinosteroid stimulus	163	464
158	GO:0010182	sugar mediated signaling pathway	163	695
159	GO:0009958	positive gravitropism	162	718
160	GO:0042787	protein ubiquitination involved in ubiquitin-dependent protein catabolic process	162	704
161	GO:0010449	root meristem growth	161	475
162	GO:0048481	ovule development	161	815
163	GO:0042493	response to drug	160	724
164	GO:0006206	pyrimidine nucleobase metabolic process	159	794
165	GO:0009817	defense response to fungus, incompatible interaction	159	611
166	GO:0006511	ubiquitin-dependent protein catabolic process	157	695
167	GO:0010075	regulation of meristem growth	157	481
168	GO:0045087	innate immune response	156	511
169	GO:0000162	tryptophan biosynthetic process	155	481
170	GO:0009788	negative regulation of abscisic acid mediated signaling pathway	155	755
171	GO:0048229	gametophyte development	155	399
172	GO:0071215	cellular response to abscisic acid stimulus	155	568
173	GO:0002119	nematode larval development	154	561
174	GO:0009553	embryo sac development	154	748
175	GO:0000165	MAPK cascade	152	640
176	GO:0009451	RNA modification	152	559
177	GO:0006396	RNA processing	151	745
178	GO:0010043	response to zinc ion	151	605
179	GO:0008285	negative regulation of cell proliferation	150	763
180	GO:0002213	defense response to insect	149	330
181	GO:0015992	proton transport	149	640
182	GO:0005975	carbohydrate metabolic process	148	586
183	GO:0010584	pollen exine formation	148	447
184	GO:0015979	photosynthesis	148	633
185	GO:0009627	systemic acquired resistance	147	443
186	GO:0009863	salicylic acid mediated signaling pathway	147	604
187	GO:0010467	gene expression	147	705
188	GO:0034654	nucleobase-containing compound biosynthetic process	147	686
189	GO:0051716	cellular response to stimulus	147	539
190	GO:0006812	cation transport	146	758
191	GO:0018105	peptidyl-serine phosphorylation	145	781
192	GO:0009740	gibberellic acid mediated signaling pathway	144	433
193	GO:0032259	methylation	144	680
194	GO:0042335	cuticle development	143	637
195	GO:0048513	organ development	142	694
196	GO:0051707	response to other organism	142	390
197	GO:0006974	response to DNA damage stimulus	141	834
198	GO:0007067	mitosis	141	742
199	GO:0007166	cell surface receptor signaling pathway	139	452
200	GO:0048518	positive regulation of biological process	139	620
201	GO:0051252	regulation of RNA metabolic process	139	675
202	GO:0009811	stilbene biosynthetic process	138	404
203	GO:0001101	response to acid	137	431
204	GO:0007049	cell cycle	137	665
205	GO:0010286	heat acclimation	137	470
206	GO:0010114	response to red light	136	472

207	GO:0034605	cellular response to heat	135	574
208	GO:0042127	regulation of cell proliferation	135	599
209	GO:0071704	organic substance metabolic process	135	461
210	GO:0010089	xylem development	134	402
211	GO:0010027	thylakoid membrane organization	133	441
212	GO:0048523	negative regulation of cellular process	133	541
213	GO:0050789	regulation of biological process	133	467
214	GO:0006855	drug transmembrane transport	132	706
215	GO:0008152	metabolic process	132	453
216	GO:0016192	vesicle-mediated transport	132	634
217	GO:0042221	response to chemical stimulus	132	500
218	GO:0006804	peroxidase reaction	131	458
219	GO:0009615	response to virus	130	505
220	GO:0010628	positive regulation of gene expression	130	553
221	GO:0009637	response to blue light	129	605
222	GO:0010016	shoot morphogenesis	128	247
223	GO:0048825	cotyledon development	127	394
224	GO:0065008	regulation of biological quality	127	647
225	GO:0071472	cellular response to salt stress	127	486
226	GO:0030244	cellulose biosynthetic process	126	517
227	GO:0090090	negative regulation of canonical Wnt receptor signaling pathway	126	476
228	GO:0006898	receptor-mediated endocytosis	125	382
229	GO:0022008	neurogenesis	125	558
230	GO:0048731	system development	125	523
231	GO:0052544	defense response by callose deposition in cell wall	125	585
232	GO:0010082	regulation of root meristem growth	124	328
233	GO:0010102	lateral root morphogenesis	124	324
234	GO:1900459	positive regulation of brassinosteroid mediated signaling pathway	124	601
235	GO:0006807	nitrogen compound metabolic process	123	478
236	GO:0009910	negative regulation of flower development	123	573
237	GO:0031348	negative regulation of defense response	123	598
238	GO:0038095	Fc-epsilon receptor signaling pathway	123	419
239	GO:0009556	microsporogenesis	122	573
240	GO:0009901	anther dehiscence	122	306
241	GO:0048522	positive regulation of cellular process	122	641
242	GO:0043488	regulation of mRNA stability	121	435
243	GO:0006012	galactose metabolic process	120	504
244	GO:0009825	multidimensional cell growth	120	416
245	GO:0034645	cellular macromolecule biosynthetic process	120	597
246	GO:0050852	T cell receptor signaling pathway	120	416
247	GO:0009664	plant-type cell wall organization	119	286
248	GO:0051091	positive regulation of sequence-specific DNA binding transcription factor activity	119	441
249	GO:0006915	apoptotic process	118	556
250	GO:0010380	regulation of chlorophyll biosynthetic process	118	341
251	GO:0007283	spermatogenesis	117	600
252	GO:0050821	protein stabilization	117	666
253	GO:0002223	stimulatory C-type lectin receptor signaling pathway	116	427
254	GO:0006897	endocytosis	116	609
255	GO:0008361	regulation of cell size	116	448
256	GO:0010501	RNA secondary structure unwinding	116	611
257	GO:0040008	regulation of growth	116	405
258	GO:0006814	sodium ion transport	115	493
259	GO:0009631	cold acclimation	115	411
260	GO:0010030	positive regulation of seed germination	115	434
261	GO:0010193	response to ozone	115	485
262	GO:0070301	cellular response to hydrogen peroxide	115	513
263	GO:0009117	nucleotide metabolic process	113	494

264	GO:0009630	gravitropism	113	447
265	GO:0048856	anatomical structure development	113	492
266	GO:0051726	regulation of cell cycle	113	483
267	GO:0006888	ER to Golgi vesicle-mediated transport	111	500
268	GO:0006970	response to osmotic stress	111	356
269	GO:0010090	trichome morphogenesis	111	577
270	GO:0043068	positive regulation of programmed cell death	111	332
271	GO:0045454	cell redox homeostasis	111	379
272	GO:2000026	regulation of multicellular organismal development	111	268
273	GO:0007264	small GTPase mediated signal transduction	110	389
274	GO:0051704	multi-organism process	110	295
275	GO:0000003	reproduction	109	374
276	GO:0010033	response to organic substance	109	385
277	GO:0031145	anaphase-promoting complex-dependent proteasomal ubiquitin-dependent protein catabolic process	109	351
278	GO:0009789	positive regulation of abscisic acid mediated signaling pathway	108	461
279	GO:0010091	trichome branching	108	411
280	GO:0015976	carbon utilization	108	386
281	GO:0002237	response to molecule of bacterial origin	107	400
282	GO:0010431	seed maturation	107	363
283	GO:0042546	cell wall biogenesis	107	259
284	GO:0071281	cellular response to iron ion	107	440
285	GO:0006119	oxidative phosphorylation	106	410
286	GO:0010359	regulation of anion channel activity	106	213
287	GO:0080022	primary root development	106	364
288	GO:0008380	RNA splicing	105	505
289	GO:0016071	mRNA metabolic process	105	391
290	GO:0009682	induced systemic resistance	104	242
291	GO:0010017	red or far-red light signaling pathway	104	528
292	GO:0048573	photoperiodism, flowering	103	395
293	GO:0065007	biological regulation	103	408
294	GO:0000302	response to reactive oxygen species	102	404
295	GO:0009736	cytokinin mediated signaling pathway	102	455
296	GO:0044249	cellular biosynthetic process	102	372
297	GO:0051607	defense response to virus	102	404
298	GO:0070417	cellular response to cold	102	374
299	GO:0006013	mannose metabolic process	101	366
300	GO:0006544	glycine metabolic process	101	526
301	GO:0006560	proline metabolic process	101	496
302	GO:0007568	aging	101	408
303	GO:0010103	stomatal complex morphogenesis	101	294
304	GO:0016045	detection of bacterium	101	208
305	GO:0051260	protein homooligomerization	101	449
306	GO:0051437	positive regulation of ubiquitin-protein ligase activity involved in mitotic cell cycle	101	305
307	GO:0006446	regulation of translational initiation	100	330
308	GO:0006749	glutathione metabolic process	100	333
309	GO:1900425	negative regulation of defense response to bacterium	100	224
310	GO:2000377	regulation of reactive oxygen species metabolic process	100	521
311	GO:0007420	brain development	99	488
312	GO:0010540	basipetal auxin transport	99	522
313	GO:0034599	cellular response to oxidative stress	99	449
314	GO:0046487	glyoxylate metabolic process	99	319
315	GO:0048564	photosystem I assembly	99	307
316	GO:0006417	regulation of translation	98	547
317	GO:0006890	retrograde vesicle-mediated transport, Golgi to ER	98	426
318	GO:0006996	organelle organization	98	490
319	GO:0009944	polarity specification of adaxial/abaxial axis	98	408
320	GO:0016049	cell growth	98	427

321	GO:0006525	arginine metabolic process	97	498
322	GO:0009749	response to glucose stimulus	97	441
323	GO:0010207	photosystem II assembly	97	294
324	GO:0031347	regulation of defense response	97	294
325	GO:0009411	response to UV	96	434
326	GO:0010118	stomatal movement	96	313
327	GO:0043065	positive regulation of apoptotic process	96	530
328	GO:0048765	root hair cell differentiation	96	376
329	GO:0051436	negative regulation of ubiquitin-protein ligase activity involved in mitotic cell cycle	96	306
330	GO:0080090	regulation of primary metabolic process	96	403
331	GO:0006563	L-serine metabolic process	95	442
332	GO:0006566	threonine metabolic process	95	466
333	GO:0033554	cellular response to stress	94	359
334	GO:0090263	positive regulation of canonical Wnt receptor signaling pathway	94	294
335	GO:1900057	positive regulation of leaf senescence	94	227
336	GO:0000272	polysaccharide catabolic process	93	227
337	GO:0006000	fructose metabolic process	93	338
338	GO:0009926	auxin polar transport	93	461
339	GO:0012501	programmed cell death	93	335
340	GO:0048519	negative regulation of biological process	93	345
341	GO:0048608	reproductive structure development	93	367
342	GO:0002221	pattern recognition receptor signaling pathway	92	484
343	GO:0006366	transcription from RNA polymerase II promoter	92	479
344	GO:0006571	tyrosine biosynthetic process	92	378
345	GO:0006744	ubiquinone biosynthetic process	92	440
346	GO:0008340	determination of adult lifespan	92	406
347	GO:0009094	L-phenylalanine biosynthetic process	92	378
348	GO:0009755	hormone-mediated signaling pathway	92	368
349	GO:0010204	defense response signaling pathway, resistance gene-independent	92	224
350	GO:0030433	ER-associated protein catabolic process	92	428
351	GO:0000086	G2/M transition of mitotic cell cycle	91	424
352	GO:0000963	mitochondrial RNA processing	91	260
353	GO:0006406	mRNA export from nucleus	91	481
354	GO:0006568	tryptophan metabolic process	91	398
355	GO:0009750	response to fructose stimulus	91	350
356	GO:0010227	floral organ abscission	91	236
357	GO:0008219	cell death	90	393
358	GO:0006955	immune response	88	207
359	GO:0009059	macromolecule biosynthetic process	88	377
360	GO:0010154	fruit development	88	403
361	GO:0051302	regulation of cell division	88	330
362	GO:0052033	pathogen-associated molecular pattern dependent induction by symbiont of host innate immune response	88	323
363	GO:0009610	response to symbiotic fungus	87	311
364	GO:0009934	regulation of meristem structural organization	87	373
365	GO:0048658	tapetal layer development	87	173
366	GO:2000022	regulation of jasmonic acid mediated signaling pathway	87	347
367	GO:0001558	regulation of cell growth	86	343
368	GO:0009827	plant-type cell wall modification	86	348
369	GO:0010268	brassinosteroid homeostasis	86	258
370	GO:0060071	Wnt receptor signaling pathway, planar cell polarity pathway	86	291
371	GO:0080092	regulation of pollen tube growth	86	394
372	GO:0009636	response to toxin	85	293
373	GO:0031323	regulation of cellular metabolic process	85	315
374	GO:0048354	mucilage biosynthetic process involved in seed coat development	85	233
375	GO:0048869	cellular developmental process	85	207

376	GO:0006623	protein targeting to vacuole	84	316
377	GO:0009864	induced systemic resistance, jasmonic acid mediated signaling pathway	84	235
378	GO:0009960	endosperm development	84	319
379	GO:0010252	auxin homeostasis	84	342
380	GO:0045490	pectin catabolic process	84	185
381	GO:0048829	root cap development	84	253
382	GO:0006338	chromatin remodeling	83	429
383	GO:0007018	microtubule-based movement	83	406
384	GO:0009718	anthocyanin biosynthetic process	83	165
385	GO:0010068	protoderm histogenesis	83	406
386	GO:0042147	retrograde transport, endosome to Golgi	83	368
387	GO:0070936	protein K48-linked ubiquitination	83	340
388	GO:0006283	transcription-coupled nucleotide-excision repair	82	535
389	GO:0010152	pollen maturation	82	346
390	GO:0061077	chaperone-mediated protein folding	82	359
391	GO:0006570	tyrosine metabolic process	81	259
392	GO:0007154	cell communication	81	286
393	GO:0010192	mucilage biosynthetic process	81	267
394	GO:0038061	NIK/NF-kappaB cascade	81	239
395	GO:0043087	regulation of GTPase activity	81	338
396	GO:0044267	cellular protein metabolic process	81	255
397	GO:0090307	spindle assembly involved in mitosis	81	289
398	GO:0034641	cellular nitrogen compound metabolic process	80	446
399	GO:0042631	cellular response to water deprivation	80	261
400	GO:0009605	response to external stimulus	79	329
401	GO:0010214	seed coat development	79	303
402	GO:0019886	antigen processing and presentation of exogenous peptide antigen via MHC class II	79	395
403	GO:0048437	floral organ development	79	219
404	GO:0071897	DNA biosynthetic process	79	367
405	GO:0007030	Golgi organization	78	342
406	GO:0007399	nervous system development	78	373
407	GO:0009625	response to insect	78	268
408	GO:0016042	lipid catabolic process	78	286
409	GO:0030308	negative regulation of cell growth	78	287
410	GO:0000959	mitochondrial RNA metabolic process	77	414
411	GO:0009294	DNA mediated transformation	77	383
412	GO:0010015	root morphogenesis	77	178
413	GO:0016579	protein deubiquitination	77	387
414	GO:0044271	cellular nitrogen compound biosynthetic process	77	336
415	GO:0080147	root hair cell development	77	245
416	GO:1900150	regulation of defense response to fungus	77	341
417	GO:0000724	double-strand break repair via homologous recombination	76	493
418	GO:0000911	cytokinesis by cell plate formation	76	307
419	GO:0006369	termination of RNA polymerase II transcription	76	423
420	GO:0019722	calcium-mediated signaling	76	396
421	GO:0032436	positive regulation of proteasomal ubiquitin-dependent protein catabolic process	76	368
422	GO:0043622	cortical microtubule organization	76	443
423	GO:0048580	regulation of post-embryonic development	76	140
424	GO:0070534	protein K63-linked ubiquitination	76	298
425	GO:0003006	developmental process involved in reproduction	75	298
426	GO:0006694	steroid biosynthetic process	75	289
427	GO:0018298	protein-chromophore linkage	75	430
428	GO:0018874	benzoate metabolic process	75	208
429	GO:0042938	dipeptide transport	75	263
430	GO:0071369	cellular response to ethylene stimulus	75	216
431	GO:0006120	mitochondrial electron transport, NADH to ubiquinone	74	300

432	GO:0009585	red, far-red light phototransduction	74	336
433	GO:0031047	gene silencing by RNA	74	301
434	GO:0048583	regulation of response to stimulus	74	265
435	GO:0000462	maturation of SSU-rRNA from tricistronic rRNA transcript (SSU-rRNA, 5.8S rRNA, LSU-rRNA)	73	205
436	GO:0006098	pentose-phosphate shunt	73	292
437	GO:0009267	cellular response to starvation	73	291
438	GO:0009932	cell tip growth	73	439
439	GO:0010199	organ boundary specification between lateral organs and the meristem	73	214
440	GO:0022900	electron transport chain	73	246
441	GO:0051179	localization	73	215
442	GO:0080168	abscisic acid transport	73	252
443	GO:0006303	double-strand break repair via nonhomologous end joining	72	411
444	GO:0006977	DNA damage response, signal transduction by p53 class mediator resulting in cell cycle arrest	72	391
445	GO:0016556	mRNA modification	72	270
446	GO:0043069	negative regulation of programmed cell death	72	296
447	GO:0048467	gynoecium development	72	315
448	GO:0070370	cellular heat acclimation	72	218
449	GO:0007005	mitochondrion organization	71	390
450	GO:0008356	asymmetric cell division	71	290
451	GO:0009992	cellular water homeostasis	71	174
452	GO:0042939	tripeptide transport	71	258
453	GO:0048509	regulation of meristem development	71	213
454	GO:0051510	regulation of unidimensional cell growth	71	307
455	GO:0071365	cellular response to auxin stimulus	71	219
456	GO:0009759	indole glucosinolate biosynthetic process	70	137
457	GO:0010035	response to inorganic substance	70	178
458	GO:0010105	negative regulation of ethylene mediated signaling pathway	70	315
459	GO:0010345	suberin biosynthetic process	70	210
460	GO:0048359	mucilage metabolic process involved seed coat development	70	282
461	GO:0050826	response to freezing	70	163
462	GO:0002764	immune response-regulating signaling pathway	69	127
463	GO:0005983	starch catabolic process	69	244
464	GO:0019538	protein metabolic process	69	330
465	GO:0090400	stress-induced premature senescence	69	155
466	GO:1900034	regulation of cellular response to heat	69	329
467	GO:2000012	regulation of auxin polar transport	69	223
468	GO:0006368	transcription elongation from RNA polymerase II promoter	68	444
469	GO:0006513	protein monoubiquitination	68	295
470	GO:0006521	regulation of cellular amino acid metabolic process	68	218
471	GO:0006531	aspartate metabolic process	68	301
472	GO:0006554	lysine catabolic process	68	276
473	GO:0006986	response to unfolded protein	68	303
474	GO:0016477	cell migration	68	275
475	GO:0043547	positive regulation of GTPase activity	68	321
476	GO:0045492	xylan biosynthetic process	68	255
477	GO:0048827	phyllome development	68	134
478	GO:0070887	cellular response to chemical stimulus	68	290
479	GO:0001649	osteoblast differentiation	67	206
480	GO:0006260	DNA replication	67	368
481	GO:0010187	negative regulation of seed germination	67	262
482	GO:0042594	response to starvation	67	295
483	GO:0051321	meiotic cell cycle	67	229
484	GO:0019233	sensory perception of pain	66	390
485	GO:0019643	reductive tricarboxylic acid cycle	66	207

486	GO:0006310	DNA recombination	65	374
487	GO:0006771	riboflavin metabolic process	65	269
488	GO:0009695	jasmonic acid biosynthetic process	65	179
489	GO:0009785	blue light signaling pathway	65	298
490	GO:0009875	pollen-pistil interaction	65	325
491	GO:0046039	GTP metabolic process	65	194
492	GO:0070588	calcium ion transmembrane transport	65	349
493	GO:0009862	systemic acquired resistance, salicylic acid mediated signaling pathway	64	294
494	GO:0010438	cellular response to sulfur starvation	64	151
495	GO:0018107	peptidyl-threonine phosphorylation	64	347
496	GO:0030307	positive regulation of cell growth	64	222
497	GO:0032869	cellular response to insulin stimulus	64	357
498	GO:0045815	positive regulation of gene expression, epigenetic	64	277
499	GO:0045995	regulation of embryonic development	64	247
500	GO:0046688	response to copper ion	64	148
501	GO:0051128	regulation of cellular component organization	64	272
502	GO:0060548	negative regulation of cell death	64	192
503	GO:1900056	negative regulation of leaf senescence	64	196
504	GO:0002230	positive regulation of defense response to virus by host	63	231
505	GO:0005986	sucrose biosynthetic process	63	241
506	GO:0009693	ethylene biosynthetic process	63	211
507	GO:0009800	cinnamic acid biosynthetic process	63	118
508	GO:0009835	fruit ripening	63	142
509	GO:0010148	transpiration	63	200
510	GO:0010208	pollen wall assembly	63	257
511	GO:0010315	auxin efflux	63	248
512	GO:0040011	locomotion	63	241
513	GO:0042744	hydrogen peroxide catabolic process	63	212
514	GO:0046685	response to arsenic-containing substance	63	250
515	GO:0000226	microtubule cytoskeleton organization	62	280
516	GO:0006278	RNA-dependent DNA replication	62	228
517	GO:0006284	base-excision repair	62	398
518	GO:0006342	chromatin silencing	62	295
519	GO:0006522	alanine metabolic process	62	286
520	GO:0006552	leucine catabolic process	62	344
521	GO:0006874	cellular calcium ion homeostasis	62	266
522	GO:0007584	response to nutrient	62	345
523	GO:0009853	photorespiration	62	248
524	GO:0034220	ion transmembrane transport	62	249
525	GO:0040029	regulation of gene expression, epigenetic	62	338
526	GO:0009306	protein secretion	61	260
527	GO:0016099	monoterpenoid biosynthetic process	61	169
528	GO:0016925	protein sumoylation	61	385
529	GO:0031648	protein destabilization	61	240
530	GO:0032508	DNA duplex unwinding	61	392
531	GO:0045471	response to ethanol	61	208
532	GO:0048507	meristem development	61	195
533	GO:0048657	tapetal cell differentiation	61	156
534	GO:0000160	two-component signal transduction system (phosphorelay)	60	284
535	GO:0006334	nucleosome assembly	60	227
536	GO:0006550	isoleucine catabolic process	60	309
537	GO:0006574	valine catabolic process	60	309
538	GO:0009814	defense response, incompatible interaction	60	242
539	GO:0010072	primary shoot apical meristem specification	60	274
540	GO:0010073	meristem maintenance	60	286
541	GO:0010197	polar nucleus fusion	60	241
542	GO:0017148	negative regulation of translation	60	254
543	GO:0019684	photosynthesis, light reaction	60	298

544	GO:0033209	tumor necrosis factor-mediated signaling pathway	60	169
545	GO:0048766	root hair initiation	60	291
546	GO:0071310	cellular response to organic substance	60	238
547	GO:0080060	integument development	60	122
548	GO:0000103	sulfate assimilation	59	213
549	GO:0002479	antigen processing and presentation of exogenous peptide antigen via MHC class I, TAP-dependent	59	168
550	GO:0006182	cGMP biosynthetic process	59	163
551	GO:0007062	sister chromatid cohesion	59	276
552	GO:0009821	alkaloid biosynthetic process	59	127
553	GO:0010480	microsporocyte differentiation	59	171
554	GO:0019497	hexachlorocyclohexane metabolic process	59	225
555	GO:0019761	glucosinolate biosynthetic process	59	113
556	GO:0030010	establishment of cell polarity	59	216
557	GO:0032501	multicellular organismal process	59	282
558	GO:0006308	DNA catabolic process	58	399
559	GO:0006879	cellular iron ion homeostasis	58	285
560	GO:0009832	plant-type cell wall biogenesis	58	303
561	GO:0009933	meristem structural organization	58	259
562	GO:0030155	regulation of cell adhesion	58	204
563	GO:0006099	tricarboxylic acid cycle	57	247
564	GO:0009888	tissue development	57	202
565	GO:0009942	longitudinal axis specification	57	198
566	GO:0015986	ATP synthesis coupled proton transport	57	226
567	GO:0016125	sterol metabolic process	57	173
568	GO:0034613	cellular protein localization	57	286
569	GO:0048586	regulation of long-day photoperiodism, flowering	57	208
570	GO:0048589	developmental growth	57	271
571	GO:0071470	cellular response to osmotic stress	57	202
572	GO:0006813	potassium ion transport	56	263
573	GO:0006869	lipid transport	56	206
574	GO:0006887	exocytosis	56	227
575	GO:0009877	nodulation	56	247
576	GO:0010976	positive regulation of neuron projection development	56	333
577	GO:0031647	regulation of protein stability	56	291
578	GO:0034059	response to anoxia	56	145
579	GO:0048441	petal development	56	153
580	GO:0006914	autophagy	55	287
581	GO:0006968	cellular defense response	55	171
582	GO:0007059	chromosome segregation	55	283
583	GO:0010037	response to carbon dioxide	55	238
584	GO:0010417	glucuronoxylan biosynthetic process	55	172
585	GO:0015706	nitrate transport	55	193
586	GO:0016311	dephosphorylation	55	302
587	GO:0019252	starch biosynthetic process	55	290
588	GO:0032355	response to estradiol stimulus	55	181
589	GO:0048468	cell development	55	311
590	GO:0000028	ribosomal small subunit assembly	54	110
591	GO:0002238	response to molecule of fungal origin	54	115
592	GO:0009686	gibberellin biosynthetic process	54	249
593	GO:0010629	negative regulation of gene expression	54	188
594	GO:0032922	circadian regulation of gene expression	54	260
595	GO:0045727	positive regulation of translation	54	228
596	GO:0048443	stamen development	54	232
597	GO:0048477	oogenesis	54	334
598	GO:0051603	proteolysis involved in cellular protein catabolic process	54	144
599	GO:0051782	negative regulation of cell division	54	218
600	GO:0060255	regulation of macromolecule metabolic process	54	201
601	GO:0090333	regulation of stomatal closure	54	167
602	GO:1900140	regulation of seedling development	54	157

603	GO:1900426	positive regulation of defense response to bacterium	54	286
604	GO:0000027	ribosomal large subunit assembly	53	193
605	GO:0000380	alternative nuclear mRNA splicing, via spliceosome	53	209
606	GO:0009060	aerobic respiration	53	178
607	GO:0009405	pathogenesis	53	287
608	GO:0009850	auxin metabolic process	53	293
609	GO:0010942	positive regulation of cell death	53	251
610	GO:0016131	brassinosteroid metabolic process	53	119
611	GO:0019482	beta-alanine metabolic process	53	224
612	GO:0043123	positive regulation of I-kappaB kinase/NF-kappaB cascade	53	203
613	GO:0048768	root hair cell tip growth	53	272
614	GO:0051028	mRNA transport	53	230
615	GO:0071333	cellular response to glucose stimulus	53	162
616	GO:0006796	phosphate-containing compound metabolic process	52	229
617	GO:0006817	phosphate ion transport	52	170
618	GO:0009269	response to desiccation	52	114
619	GO:0009861	jasmonic acid and ethylene-dependent systemic resistance	52	270
620	GO:0010411	xyloglucan metabolic process	52	170
621	GO:0022607	cellular component assembly	52	245
622	GO:0048317	seed morphogenesis	52	203
623	GO:0050790	regulation of catalytic activity	52	197
624	GO:0000082	G1/S transition of mitotic cell cycle	51	210
625	GO:0000413	protein peptidyl-prolyl isomerization	51	228
626	GO:0001889	liver development	51	272
627	GO:0006464	cellular protein modification process	51	211
628	GO:0010026	trichome differentiation	51	175
629	GO:0016055	Wnt receptor signaling pathway	51	237
630	GO:0031540	regulation of anthocyanin biosynthetic process	51	98
631	GO:0042753	positive regulation of circadian rhythm	51	202
632	GO:0043086	negative regulation of catalytic activity	51	221
633	GO:0046251	limonene catabolic process	51	132
634	GO:0048646	anatomical structure formation involved in morphogenesis	51	179
635	GO:0051276	chromosome organization	51	300
636	GO:0070979	protein K11-linked ubiquitination	51	223
637	GO:0071229	cellular response to acid	51	165
638	GO:0071732	cellular response to nitric oxide	51	225
639	GO:2000031	regulation of salicylic acid mediated signaling pathway	51	219
640	GO:2000034	regulation of seed maturation	51	189
641	GO:0001701	in utero embryonic development	50	213
642	GO:0006302	double-strand break repair	50	356
643	GO:0006378	mRNA polyadenylation	50	293
644	GO:0006606	protein import into nucleus	50	278
645	GO:0006803	glutathione conjugation reaction	50	112
646	GO:0007080	mitotic metaphase plate congression	50	155
647	GO:0008360	regulation of cell shape	50	342
648	GO:0009311	oligosaccharide metabolic process	50	155
649	GO:0010233	phloem transport	50	76
650	GO:0015991	ATP hydrolysis coupled proton transport	50	190
651	GO:0015995	chlorophyll biosynthetic process	50	251
652	GO:0022603	regulation of anatomical structure morphogenesis	50	144
653	GO:0030036	actin cytoskeleton organization	50	225
654	GO:0043484	regulation of RNA splicing	50	364
655	GO:0044550	secondary metabolite biosynthetic process	50	132
656	GO:0050793	regulation of developmental process	50	180
657	GO:0051092	positive regulation of NF-kappaB transcription factor activity	50	165
658	GO:0080001	mucilage extrusion from seed coat	50	200
659	GO:1901002	positive regulation of response to salt stress	50	177

660	GO:0006461	protein complex assembly	49	144
661	GO:0006486	protein glycosylation	49	327
662	GO:0006909	phagocytosis	49	244
663	GO:0007033	vacuole organization	49	175
664	GO:0008033	tRNA processing	49	285
665	GO:0032496	response to lipopolysaccharide	49	190
666	GO:0040018	positive regulation of multicellular organism growth	49	252
667	GO:0042059	negative regulation of epidermal growth factor receptor signaling pathway	49	213
668	GO:0071805	potassium ion transmembrane transport	49	329
669	GO:2000070	regulation of response to water deprivation	49	236
670	GO:0003002	regionalization	48	153
671	GO:0007389	pattern specification process	48	190
672	GO:0031426	polycistronic mRNA processing	48	144
673	GO:0040035	hermaphrodite genitalia development	48	206
674	GO:0043525	positive regulation of neuron apoptotic process	48	207
675	GO:0071497	cellular response to freezing	48	78
676	GO:0002376	immune system process	47	256
677	GO:0006725	cellular aromatic compound metabolic process	47	121
678	GO:0006839	mitochondrial transport	47	214
679	GO:0007507	heart development	47	229
680	GO:0009729	detection of brassinosteroid stimulus	47	113
681	GO:0010023	proanthocyanidin biosynthetic process	47	118
682	GO:0010336	gibberellic acid homeostasis	47	139
683	GO:0014070	response to organic cyclic compound	47	187
684	GO:0016114	terpenoid biosynthetic process	47	243
685	GO:0030001	metal ion transport	47	205
686	GO:0031175	neuron projection development	47	151
687	GO:0045489	pectin biosynthetic process	47	175
688	GO:0046034	ATP metabolic process	47	258
689	GO:0048579	negative regulation of long-day photoperiodism, flowering	47	279
690	GO:0080086	stamen filament development	47	127
691	GO:0090332	stomatal closure	47	224
692	GO:0006011	UDP-glucose metabolic process	46	172
693	GO:0006811	ion transport	46	255
694	GO:0009903	chloroplast avoidance movement	46	189
695	GO:0010053	root epidermal cell differentiation	46	221
696	GO:0015940	pantothenate biosynthetic process	46	222
697	GO:0016573	histone acetylation	46	319
698	GO:0021766	hippocampus development	46	218
699	GO:0022402	cell cycle process	46	214
700	GO:0043085	positive regulation of catalytic activity	46	206
701	GO:0043966	histone H3 acetylation	46	280
702	GO:0043967	histone H4 acetylation	46	328
703	GO:0051289	protein homotetramerization	46	182
704	GO:0072593	reactive oxygen species metabolic process	46	224
705	GO:0072659	protein localization in plasma membrane	46	181
706	GO:2000028	regulation of photoperiodism, flowering	46	177
707	GO:0000919	cell plate assembly	45	186
708	GO:0002098	tRNA wobble uridine modification	45	199
709	GO:0002215	defense response to nematode	45	108
710	GO:0007034	vacuolar transport	45	142
711	GO:0007095	mitotic cell cycle G2/M transition DNA damage checkpoint	45	266
712	GO:0007281	germ cell development	45	201
713	GO:0008104	protein localization	45	263
714	GO:0008643	carbohydrate transport	45	201
715	GO:0009554	megasporogenesis	45	131
716	GO:0009833	primary cell wall biogenesis	45	224

717	GO:0010120	camalexin biosynthetic process	45	136
718	GO:0042023	DNA endoreduplication	45	203
719	GO:0042752	regulation of circadian rhythm	45	242
720	GO:0048868	pollen tube development	45	199
721	GO:0080188	RNA-directed DNA methylation	45	322
722	GO:0006367	transcription initiation from RNA polymerase II promoter	44	238
723	GO:0007029	endoplasmic reticulum organization	44	186
724	GO:0007155	cell adhesion	44	226
725	GO:0009097	isoleucine biosynthetic process	44	197
726	GO:0009938	negative regulation of gibberellic acid mediated signaling pathway	44	112
727	GO:0009970	cellular response to sulfate starvation	44	147
728	GO:0016226	iron-sulfur cluster assembly	44	183
729	GO:0032465	regulation of cytokinesis	44	180
730	GO:0042981	regulation of apoptotic process	44	223
731	GO:0043162	ubiquitin-dependent protein catabolic process via the multivesicular body sorting pathway	44	139
732	GO:0044281	small molecule metabolic process	44	144
733	GO:0048444	floral organ morphogenesis	44	233
734	GO:0048574	long-day photoperiodism, flowering	44	131
735	GO:0051259	protein oligomerization	44	171
736	GO:0055046	microgametogenesis	44	211
737	GO:0071356	cellular response to tumor necrosis factor	44	207
738	GO:0080110	sporopollenin biosynthetic process	44	168
739	GO:0001578	microtubule bundle formation	43	276
740	GO:0006730	one-carbon metabolic process	43	165
741	GO:0006878	cellular copper ion homeostasis	43	175
742	GO:0006995	cellular response to nitrogen starvation	43	184
743	GO:0007015	actin filament organization	43	291
744	GO:0007411	axon guidance	43	217
745	GO:0009098	leucine biosynthetic process	43	192
746	GO:0009099	valine biosynthetic process	43	192
747	GO:0009887	organ morphogenesis	43	172
748	GO:0009966	regulation of signal transduction	43	189
749	GO:0010067	procambium histogenesis	43	84
750	GO:0018193	peptidyl-amino acid modification	43	243
751	GO:0043481	anthocyanin accumulation in tissues in response to UV light	43	214
752	GO:0045732	positive regulation of protein catabolic process	43	253
753	GO:0046907	intracellular transport	43	223
754	GO:0048235	pollen sperm cell differentiation	43	135
755	GO:0048584	positive regulation of response to stimulus	43	230
756	GO:0048629	trichome patterning	43	157
757	GO:0051453	regulation of intracellular pH	43	162
758	GO:0070911	global genome nucleotide-excision repair	43	249
759	GO:0000723	telomere maintenance	42	332
760	GO:0006071	glycerol metabolic process	42	148
761	GO:0006139	nucleobase-containing compound metabolic process	42	185
762	GO:0007140	male meiosis	42	200
763	GO:0007596	blood coagulation	42	148
764	GO:0009266	response to temperature stimulus	42	207
765	GO:0009790	embryo development	42	195
766	GO:0009808	lignin metabolic process	42	123
767	GO:0009962	regulation of flavonoid biosynthetic process	42	67
768	GO:0010325	raffinose family oligosaccharide biosynthetic process	42	118
769	GO:0016117	carotenoid biosynthetic process	42	216
770	GO:0018996	molting cycle, collagen and cuticulin-based cuticle	42	110
771	GO:0035690	cellular response to drug	42	296
772	GO:0043524	negative regulation of neuron apoptotic process	42	216
773	GO:0048208	COPII vesicle coating	42	143

774	GO:0051262	protein tetramerization	42	203
775	GO:0051298	centrosome duplication	42	137
776	GO:0090058	metaxylem development	42	138
777	GO:2000114	regulation of establishment of cell polarity	42	176
778	GO:2000280	regulation of root development	42	82
779	GO:0001525	angiogenesis	41	231
780	GO:0006561	proline biosynthetic process	41	114
781	GO:0006896	Golgi to vacuole transport	41	156
782	GO:0007346	regulation of mitotic cell cycle	41	148
783	GO:0008406	gonad development	41	131
784	GO:0009567	double fertilization forming a zygote and endosperm	41	105
785	GO:0009638	phototropism	41	150
786	GO:0009639	response to red or far red light	41	152
787	GO:0009641	shade avoidance	41	128
788	GO:0009694	jasmonic acid metabolic process	41	106
789	GO:0010093	specification of floral organ identity	41	183
790	GO:0010439	regulation of glucosinolate biosynthetic process	41	124
791	GO:0030968	endoplasmic reticulum unfolded protein response	41	197
792	GO:0032091	negative regulation of protein binding	41	185
793	GO:0033993	response to lipid	41	148
794	GO:0042769	DNA damage response, detection of DNA damage	41	189
795	GO:0043009	chordate embryonic development	41	113
796	GO:0045491	xylan metabolic process	41	153
797	GO:0045597	positive regulation of cell differentiation	41	193
798	GO:0048759	vessel member cell differentiation	41	112
799	GO:0055062	phosphate ion homeostasis	41	173
800	GO:0080027	response to herbivore	41	191
801	GO:0000281	cytokinesis after mitosis	40	213
802	GO:0000381	regulation of alternative nuclear mRNA splicing, via spliceosome	40	296
803	GO:0006027	glycosaminoglycan catabolic process	40	166
804	GO:0006325	chromatin organization	40	236
805	GO:0006448	regulation of translational elongation	40	199
806	GO:0006906	vesicle fusion	40	161
807	GO:0007050	cell cycle arrest	40	257
808	GO:0009961	response to 1-aminocyclopropane-1-carboxylic acid	40	145
809	GO:0010038	response to metal ion	40	225
810	GO:0010039	response to iron ion	40	157
811	GO:0010363	regulation of plant-type hypersensitive response	40	171
812	GO:0010492	maintenance of shoot apical meristem identity	40	211
813	GO:0015691	cadmium ion transport	40	194
814	GO:0016126	sterol biosynthetic process	40	159
815	GO:0019827	stem cell maintenance	40	198
816	GO:0030097	hemopoiesis	40	183
817	GO:0031062	positive regulation of histone methylation	40	179
818	GO:0050829	defense response to Gram-negative bacterium	40	101
819	GO:0051555	flavonol biosynthetic process	40	83
820	GO:0052696	flavonoid glucuronidation	40	90
821	GO:0060918	auxin transport	40	102
822	GO:0000722	telomere maintenance via recombination	39	245
823	GO:0006109	regulation of carbohydrate metabolic process	39	170
824	GO:0006298	mismatch repair	39	290
825	GO:0006891	intra-Golgi vesicle-mediated transport	39	185
826	GO:0007126	meiosis	39	212
827	GO:0007286	spermatid development	39	264
828	GO:0009407	toxin catabolic process	39	79
829	GO:0009704	de-etiolation	39	98
830	GO:0010074	maintenance of meristem identity	39	104
831	GO:0010234	tapetal cell fate specification	39	80
832	GO:0016569	covalent chromatin modification	39	225

833	GO:0018279	protein N-linked glycosylation via asparagine	39	183
834	GO:0019852	L-ascorbic acid metabolic process	39	165
835	GO:0032543	mitochondrial translation	39	211
836	GO:0042545	cell wall modification	39	81
837	GO:0042991	transcription factor import into nucleus	39	66
838	GO:0043433	negative regulation of sequence-specific DNA binding transcription factor activity	39	185
839	GO:0052386	cell wall thickening	39	115
840	GO:2000038	regulation of stomatal complex development	39	124
841	GO:0001934	positive regulation of protein phosphorylation	38	172
842	GO:0007010	cytoskeleton organization	38	158
843	GO:0007519	skeletal muscle tissue development	38	130
844	GO:0009612	response to mechanical stimulus	38	102
845	GO:0009646	response to absence of light	38	186
846	GO:0009812	flavonoid metabolic process	38	50
847	GO:0010077	maintenance of inflorescence meristem identity	38	184
848	GO:0016998	cell wall macromolecule catabolic process	38	69
849	GO:0018108	peptidyl-tyrosine phosphorylation	38	212
850	GO:0019872	streptomycin biosynthetic process	38	157
851	GO:0031124	mRNA 3'-end processing	38	203
852	GO:0031325	positive regulation of cellular metabolic process	38	227
853	GO:0042026	protein refolding	38	88
854	GO:0043154	negative regulation of cysteine-type endopeptidase activity involved in apoptotic process	38	184
855	GO:0048833	specification of floral organ number	38	82
856	GO:0071702	organic substance transport	38	291
857	GO:0001731	formation of translation preinitiation complex	37	98
858	GO:0006296	nucleotide-excision repair, DNA incision, 5'-to lesion	37	271
859	GO:0006826	iron ion transport	37	97
860	GO:0007032	endosome organization	37	215
861	GO:0008202	steroid metabolic process	37	174
862	GO:0009609	response to symbiotic bacterium	37	130
863	GO:0009880	embryonic pattern specification	37	79
864	GO:0010031	circumnutation	37	201
865	GO:0010452	histone H3-K36 methylation	37	163
866	GO:0014823	response to activity	37	150
867	GO:0019384	caprolactam catabolic process	37	106
868	GO:0030163	protein catabolic process	37	159
869	GO:0031053	primary miRNA processing	37	112
870	GO:0034644	cellular response to UV	37	190
871	GO:0042391	regulation of membrane potential	37	205
872	GO:0045859	regulation of protein kinase activity	37	208
873	GO:0046244	salicylic acid catabolic process	37	119
874	GO:0048830	adventitious root development	37	149
875	GO:0052543	callose deposition in cell wall	37	134
876	GO:0071577	zinc ion transmembrane transport	37	144
877	GO:0000060	protein import into nucleus, translocation	36	112
878	GO:0000183	chromatin silencing at rDNA	36	149
879	GO:0000187	activation of MAPK activity	36	140
880	GO:0006352	DNA-dependent transcription, initiation	36	179
881	GO:0006558	L-phenylalanine metabolic process	36	140
882	GO:0006825	copper ion transport	36	101
883	GO:0007267	cell-cell signaling	36	124
884	GO:0007476	imaginal disc-derived wing morphogenesis	36	208
885	GO:0007605	sensory perception of sound	36	194
886	GO:0009561	megagametogenesis	36	205
887	GO:0009870	defense response signaling pathway, resistance gene-dependent	36	217
888	GO:0010112	regulation of systemic acquired resistance	36	154
889	GO:0010582	floral meristem determinacy	36	90

890	GO:0019853	L-ascorbic acid biosynthetic process	36	146
891	GO:0036258	multivesicular body assembly	36	113
892	GO:0042276	error-prone translesion synthesis	36	167
893	GO:0045595	regulation of cell differentiation	36	285
894	GO:0051553	flavone biosynthetic process	36	132
895	GO:0051571	positive regulation of histone H3-K4 methylation	36	177
896	GO:0051924	regulation of calcium ion transport	36	89
897	GO:0070987	error-free translesion synthesis	36	139
898	GO:0001708	cell fate specification	35	90
899	GO:0006904	vesicle docking involved in exocytosis	35	154
900	GO:0006954	inflammatory response	35	119
901	GO:0010167	response to nitrate	35	137
902	GO:0015770	sucrose transport	35	126
903	GO:0031397	negative regulation of protein ubiquitination	35	186
904	GO:0031408	oxylipin biosynthetic process	35	140
905	GO:0043405	regulation of MAP kinase activity	35	156
906	GO:0043473	pigmentation	35	92
907	GO:0043623	cellular protein complex assembly	35	125
908	GO:0044419	interspecies interaction between organisms	35	161
909	GO:0045088	regulation of innate immune response	35	206
910	GO:0045229	external encapsulating structure organization	35	90
911	GO:0048545	response to steroid hormone stimulus	35	206
912	GO:0051171	regulation of nitrogen compound metabolic process	35	202
913	GO:0061418	regulation of transcription from RNA polymerase II promoter in response to hypoxia	35	160
914	GO:0071219	cellular response to molecule of bacterial origin	35	190
915	GO:0071840	cellular component organization or biogenesis	35	127
916	GO:0080144	amino acid homeostasis	35	111
917	GO:0097275	cellular ammonia homeostasis	35	88
918	GO:2000904	regulation of starch metabolic process	35	202
919	GO:0000902	cell morphogenesis	34	242
920	GO:0001503	ossification	34	153
921	GO:0003333	amino acid transmembrane transport	34	173
922	GO:0005992	trehalose biosynthetic process	34	169
923	GO:0006868	glutamine transport	34	133
924	GO:0007131	reciprocal meiotic recombination	34	301
925	GO:0010604	positive regulation of macromolecule metabolic process	34	226
926	GO:0015807	L-amino acid transport	34	114
927	GO:0015994	chlorophyll metabolic process	34	207
928	GO:0019953	sexual reproduction	34	119
929	GO:0031146	SCF-dependent proteasomal ubiquitin-dependent protein catabolic process	34	153
930	GO:0040020	regulation of meiosis	34	163
931	GO:0042273	ribosomal large subunit biogenesis	34	130
932	GO:0042344	indole glucosinolate catabolic process	34	243
933	GO:0043201	response to leucine	34	138
934	GO:0045037	protein import into chloroplast stroma	34	209
935	GO:0045040	protein import into mitochondrial outer membrane	34	76
936	GO:0046323	glucose import	34	133
937	GO:0048281	inflorescence morphogenesis	34	82
938	GO:0048438	floral whorl development	34	59
939	GO:0048813	dendrite morphogenesis	34	158
940	GO:0071629	cytoplasm-associated proteasomal ubiquitin-dependent protein catabolic process	34	124
941	GO:0080055	low affinity nitrate transport	34	117
942	GO:2000112	regulation of cellular macromolecule biosynthetic process	34	170
943	GO:0000289	nuclear-transcribed mRNA poly(A) tail shortening	33	162
944	GO:0001933	negative regulation of protein phosphorylation	33	214
945	GO:0006335	DNA replication-dependent nucleosome assembly	33	69

946	GO:0006534	cysteine metabolic process	33	150
947	GO:0006541	glutamine metabolic process	33	207
948	GO:0006661	phosphatidylinositol biosynthetic process	33	225
949	GO:0006699	bile acid biosynthetic process	33	100
950	GO:0006857	oligopeptide transport	33	151
951	GO:0007276	gamete generation	33	154
952	GO:0010044	response to aluminum ion	33	153
953	GO:0010104	regulation of ethylene mediated signaling pathway	33	139
954	GO:0010371	regulation of gibberellin biosynthetic process	33	53
955	GO:0016102	diterpenoid biosynthetic process	33	114
956	GO:0032989	cellular component morphogenesis	33	108
957	GO:0035666	TRIF-dependent toll-like receptor signaling pathway	33	126
958	GO:0043933	macromolecular complex subunit organization	33	181
959	GO:0045604	regulation of epidermal cell differentiation	33	143
960	GO:0048227	plasma membrane to endosome transport	33	119
961	GO:0055072	iron ion homeostasis	33	99
962	GO:0061025	membrane fusion	33	154
963	GO:0072583	clathrin-mediated endocytosis	33	135
964	GO:1901001	negative regulation of response to salt stress	33	211
965	GO:0000245	spliceosomal complex assembly	32	88
966	GO:0001501	skeletal system development	32	110
967	GO:0006487	protein N-linked glycosylation	32	161
968	GO:0006536	glutamate metabolic process	32	125
969	GO:0006885	regulation of pH	32	158
970	GO:0009959	negative gravitropism	32	214
971	GO:0010052	guard cell differentiation	32	90
972	GO:0010161	red light signaling pathway	32	91
973	GO:0010483	pollen tube reception	32	137
974	GO:0010623	developmental programmed cell death	32	62
975	GO:0015711	organic anion transport	32	99
976	GO:0016458	gene silencing	32	184
977	GO:0016485	protein processing	32	178
978	GO:0030447	filamentous growth	32	152
979	GO:0032879	regulation of localization	32	142
980	GO:0032968	positive regulation of transcription elongation from RNA polymerase II promoter	32	202
981	GO:0033572	transferrin transport	32	140
982	GO:0036180	filamentous growth of a population of unicellular organisms in response to biotic stimulus	32	222
983	GO:0045176	apical protein localization	32	160
984	GO:0080052	response to histidine	32	93
985	GO:0080053	response to phenylalanine	32	93
986	GO:0080113	regulation of seed growth	32	110
987	GO:0000266	mitochondrial fission	31	186
988	GO:0000290	deadenylation-dependent decapping of nuclear-transcribed mRNA	31	80
989	GO:0000447	endonucleolytic cleavage in ITS1 to separate SSU-rRNA from 5.8S rRNA and LSU-rRNA from tricistronic rRNA transcript (SSU-rRNA, 5.8S rRNA, LSU-rRNA)	31	106
990	GO:0000463	maturation of LSU-rRNA from tricistronic rRNA transcript (SSU-rRNA, 5.8S rRNA, LSU-rRNA)	31	100
991	GO:0000715	nucleotide-excision repair, DNA damage recognition	31	157
992	GO:0001932	regulation of protein phosphorylation	31	118
993	GO:0005978	glycogen biosynthetic process	31	140
994	GO:0006401	RNA catabolic process	31	109
995	GO:0006479	protein methylation	31	158
996	GO:0006662	glycerol ether metabolic process	31	100
997	GO:0006882	cellular zinc ion homeostasis	31	129
998	GO:0007409	axonogenesis	31	165
999	GO:0008543	fibroblast growth factor receptor signaling pathway	31	148

1000	GO:0009299	mRNA transcription	31	120
1001	GO:0009828	plant-type cell wall loosening	31	60
1002	GO:0009913	epidermal cell differentiation	31	114
1003	GO:0009969	xyloglucan biosynthetic process	31	125
1004	GO:0010212	response to ionizing radiation	31	222
1005	GO:0010941	regulation of cell death	31	131
1006	GO:0010981	regulation of cell wall macromolecule metabolic process	31	79
1007	GO:0016197	endosomal transport	31	162
1008	GO:0019253	reductive pentose-phosphate cycle	31	116
1009	GO:0021762	substantia nigra development	31	113
1010	GO:0023014	signal transduction by phosphorylation	31	225
1011	GO:0032197	transposition, RNA-mediated	31	118
1012	GO:0033036	macromolecule localization	31	213
1013	GO:0035067	negative regulation of histone acetylation	31	149
1014	GO:0035196	production of miRNAs involved in gene silencing by miRNA	31	168
1015	GO:0042432	indole biosynthetic process	31	99
1016	GO:0042771	DNA damage response, signal transduction by p53 class mediator resulting in induction of apoptosis	31	165
1017	GO:0045070	positive regulation of viral genome replication	31	150
1018	GO:0048236	plant-type spore development	31	62
1019	GO:0048262	determination of dorsal/ventral asymmetry	31	73
1020	GO:0048440	carpel development	31	97
1021	GO:2000785	regulation of autophagic vacuole assembly	31	142
1022	GO:0005987	sucrose catabolic process	30	108
1023	GO:0006032	chitin catabolic process	30	58
1024	GO:0006097	glyoxylate cycle	30	77
1025	GO:0006103	2-oxoglutarate metabolic process	30	109
1026	GO:0006289	nucleotide-excision repair	30	184
1027	GO:0006294	nucleotide-excision repair, preincision complex assembly	30	226
1028	GO:0007017	microtubule-based process	30	161
1029	GO:0009051	pentose-phosphate shunt, oxidative branch	30	135
1030	GO:0009687	abscisic acid metabolic process	30	107
1031	GO:0009954	proximal/distal pattern formation	30	52
1032	GO:0010076	maintenance of floral meristem identity	30	145
1033	GO:0010541	acropetal auxin transport	30	160
1034	GO:0010605	negative regulation of macromolecule metabolic process	30	136
1035	GO:0019288	isopentenyl diphosphate biosynthetic process, mevalonate-independent pathway	30	97
1036	GO:0031324	negative regulation of cellular metabolic process	30	137
1037	GO:0031334	positive regulation of protein complex assembly	30	129
1038	GO:0032880	regulation of protein localization	30	168
1039	GO:0044030	regulation of DNA methylation	30	158
1040	GO:0046148	pigment biosynthetic process	30	34
1041	GO:0051240	positive regulation of multicellular organismal process	30	197
1042	GO:0051592	response to calcium ion	30	167
1043	GO:2000024	regulation of leaf development	30	238
1044	GO:0000304	response to singlet oxygen	29	149
1045	GO:0002679	respiratory burst involved in defense response	29	80
1046	GO:0006297	nucleotide-excision repair, DNA gap filling	29	191
1047	GO:0006349	regulation of gene expression by genetic imprinting	29	139
1048	GO:0006465	signal peptide processing	29	140
1049	GO:0006605	protein targeting	29	151
1050	GO:0006833	water transport	29	71
1051	GO:0007219	Notch signaling pathway	29	102
1052	GO:0008272	sulfate transport	29	129
1053	GO:0009647	skotomorphogenesis	29	89
1054	GO:0009904	chloroplast accumulation movement	29	124
1055	GO:0009937	regulation of gibberellic acid mediated signaling pathway	29	147

1056	GO:0010205	photoinhibition	29	123
1057	GO:0010267	production of ta-siRNAs involved in RNA interference	29	177
1058	GO:0010601	positive regulation of auxin biosynthetic process	29	147
1059	GO:0010906	regulation of glucose metabolic process	29	131
1060	GO:0010971	positive regulation of G2/M transition of mitotic cell cycle	29	94
1061	GO:0015757	galactose transport	29	100
1062	GO:0015793	glycerol transport	29	93
1063	GO:0016236	macroautophagy	29	130
1064	GO:0016246	RNA interference	29	94
1065	GO:0016973	poly(A)+ mRNA export from nucleus	29	188
1066	GO:0019287	isopentenyl diphosphate biosynthetic process, mevalonate pathway	29	76
1067	GO:0019438	aromatic compound biosynthetic process	29	83
1068	GO:0019593	mannitol biosynthetic process	29	101
1069	GO:0030100	regulation of endocytosis	29	90
1070	GO:0030512	negative regulation of transforming growth factor beta receptor signaling pathway	29	96
1071	GO:0042795	snRNA transcription from RNA polymerase II promoter	29	131
1072	GO:0045824	negative regulation of innate immune response	29	127
1073	GO:0045948	positive regulation of translational initiation	29	99
1074	GO:0048528	post-embryonic root development	29	130
1075	GO:0051225	spindle assembly	29	268
1076	GO:0051290	protein heterotetramerization	29	60
1077	GO:0060271	cilium morphogenesis	29	158
1078	GO:0071222	cellular response to lipopolysaccharide	29	230
1079	GO:0071902	positive regulation of protein serine/threonine kinase activity	29	224
1080	GO:0090408	phloem nitrate loading	29	82
1081	GO:2000021	regulation of ion homeostasis	29	156
1082	GO:0000077	DNA damage checkpoint	28	147
1083	GO:0006020	inositol metabolic process	28	89
1084	GO:0006510	ATP-dependent proteolysis	28	164
1085	GO:0006555	methionine metabolic process	28	113
1086	GO:0006626	protein targeting to mitochondrion	28	141
1087	GO:0006892	post-Golgi vesicle-mediated transport	28	185
1088	GO:0006997	nucleus organization	28	109
1089	GO:0009786	regulation of asymmetric cell division	28	93
1090	GO:0009956	radial pattern formation	28	119
1091	GO:0010018	far-red light signaling pathway	28	104
1092	GO:0010078	maintenance of root meristem identity	28	105
1093	GO:0010143	cutin biosynthetic process	28	101
1094	GO:0010216	maintenance of DNA methylation	28	194
1095	GO:0010289	homogalacturonan biosynthetic process	28	122
1096	GO:0010592	positive regulation of lamellipodium assembly	28	79
1097	GO:0015783	GDP-fucose transport	28	190
1098	GO:0015786	UDP-glucose transport	28	191
1099	GO:0019068	virion assembly	28	81
1100	GO:0019915	lipid storage	28	79
1101	GO:0030334	regulation of cell migration	28	183
1102	GO:0030422	production of siRNA involved in RNA interference	28	226
1103	GO:0032467	positive regulation of cytokinesis	28	152
1104	GO:0033365	protein localization to organelle	28	106
1105	GO:0035019	somatic stem cell maintenance	28	143
1106	GO:0042593	glucose homeostasis	28	160
1107	GO:0043486	histone exchange	28	157
1108	GO:0043620	regulation of DNA-dependent transcription in response to stress	28	78
1109	GO:0048812	neuron projection morphogenesis	28	136
1110	GO:0048831	regulation of shoot development	28	99
1111	GO:0051017	actin filament bundle assembly	28	186

1112	GO:0051568	histone H3-K4 methylation	28	154
1113	GO:0071347	cellular response to interleukin-1	28	194
1114	GO:0080141	regulation of jasmonic acid biosynthetic process	28	63
1115	GO:0090351	seedling development	28	101
1116	GO:2000037	regulation of stomatal complex patterning	28	110
1117	GO:0006006	glucose metabolic process	27	84
1118	GO:0006458	'de novo' protein folding	27	110
1119	GO:0006816	calcium ion transport	27	122
1120	GO:0006865	amino acid transport	27	98
1121	GO:0010098	suspensor development	27	184
1122	GO:0010107	potassium ion import	27	105
1123	GO:0010244	response to low fluence blue light stimulus by blue low-fluence system	27	127
1124	GO:0015700	arsenite transport	27	150
1125	GO:0015824	proline transport	27	128
1126	GO:0016233	telomere capping	27	74
1127	GO:0030150	protein import into mitochondrial matrix	27	110
1128	GO:0031328	positive regulation of cellular biosynthetic process	27	117
1129	GO:0032268	regulation of cellular protein metabolic process	27	128
1130	GO:0032527	protein exit from endoplasmic reticulum	27	109
1131	GO:0034976	response to endoplasmic reticulum stress	27	81
1132	GO:0035195	gene silencing by miRNA	27	169
1133	GO:0035266	meristem growth	27	52
1134	GO:0038128	ERBB2 signaling pathway	27	100
1135	GO:0042632	cholesterol homeostasis	27	152
1136	GO:0046854	phosphatidylinositol phosphorylation	27	169
1137	GO:0046856	phosphatidylinositol dephosphorylation	27	221
1138	GO:0048511	rhythmic process	27	166
1139	GO:0048864	stem cell development	27	171
1140	GO:0051641	cellular localization	27	224
1141	GO:0060968	regulation of gene silencing	27	104
1142	GO:0072334	UDP-galactose transmembrane transport	27	193
1143	GO:0080178	5-carbamoylmethyluridine metabolic process	27	107
1144	GO:0090150	establishment of protein localization in membrane	27	57
1145	GO:1900111	positive regulation of histone H3-K9 dimethylation	27	59
1146	GO:1900864	mitochondrial RNA modification	27	92
1147	GO:2000122	negative regulation of stomatal complex development	27	78
1148	GO:2000605	positive regulation of secondary growth	27	82
1149	GO:0000373	Group II intron splicing	26	141
1150	GO:0001764	neuron migration	26	111
1151	GO:0001822	kidney development	26	106
1152	GO:0006261	DNA-dependent DNA replication	26	187
1153	GO:0006346	methylation-dependent chromatin silencing	26	141
1154	GO:0006370	7-methylguanosine mRNA capping	26	163
1155	GO:0007601	visual perception	26	182
1156	GO:0007612	learning	26	151
1157	GO:0009719	response to endogenous stimulus	26	102
1158	GO:0009768	photosynthesis, light harvesting in photosystem I	26	125
1159	GO:0009968	negative regulation of signal transduction	26	119
1160	GO:0010116	positive regulation of abscisic acid biosynthetic process	26	104
1161	GO:0010337	regulation of salicylic acid metabolic process	26	169
1162	GO:0010726	positive regulation of hydrogen peroxide metabolic process	26	89
1163	GO:0015692	lead ion transport	26	136
1164	GO:0015914	phospholipid transport	26	114
1165	GO:0030855	epithelial cell differentiation	26	110
1166	GO:0031297	replication fork processing	26	170
1167	GO:0034219	carbohydrate transmembrane transport	26	126
1168	GO:0040014	regulation of multicellular organism growth	26	165
1169	GO:0042178	xenobiotic catabolic process	26	82

1170	GO:0043407	negative regulation of MAP kinase activity	26	160
1171	GO:0043457	regulation of cellular respiration	26	64
1172	GO:0043627	response to estrogen stimulus	26	88
1173	GO:0045036	protein targeting to chloroplast	26	76
1174	GO:0045793	positive regulation of cell size	26	129
1175	GO:0048278	vesicle docking	26	99
1176	GO:0048661	positive regulation of smooth muscle cell proliferation	26	119
1177	GO:0050766	positive regulation of phagocytosis	26	69
1178	GO:0051026	chiasma assembly	26	182
1179	GO:0051130	positive regulation of cellular component organization	26	135
1180	GO:0055117	regulation of cardiac muscle contraction	26	111
1181	GO:0060919	auxin influx	26	157
1182	GO:0071392	cellular response to estradiol stimulus	26	89
1183	GO:0072661	protein targeting to plasma membrane	26	78
1184	GO:0090084	negative regulation of inclusion body assembly	26	61
1185	GO:1901000	regulation of response to salt stress	26	130
1186	GO:0000717	nucleotide-excision repair, DNA duplex unwinding	25	194
1187	GO:0005980	glycogen catabolic process	25	82
1188	GO:0006348	chromatin silencing at telomere	25	187
1189	GO:0007231	osmosensory signaling pathway	25	151
1190	GO:0007369	gastrulation	25	86
1191	GO:0007517	muscle organ development	25	165
1192	GO:0007626	locomotory behavior	25	89
1193	GO:0008333	endosome to lysosome transport	25	103
1194	GO:0009061	anaerobic respiration	25	127
1195	GO:0009268	response to pH	25	87
1196	GO:0009590	detection of gravity	25	148
1197	GO:0009696	salicylic acid metabolic process	25	74
1198	GO:0009955	adaxial/abaxial pattern specification	25	86
1199	GO:0010222	stem vascular tissue pattern formation	25	99
1200	GO:0010243	response to organic nitrogen	25	93
1201	GO:0010338	leaf formation	25	111
1202	GO:0010444	guard mother cell differentiation	25	73
1203	GO:0010634	positive regulation of epithelial cell migration	25	100
1204	GO:0015867	ATP transport	25	100
1205	GO:0016132	brassinosteroid biosynthetic process	25	98
1206	GO:0016575	histone deacetylation	25	143
1207	GO:0030182	neuron differentiation	25	95
1208	GO:0031326	regulation of cellular biosynthetic process	25	127
1209	GO:0032402	melanosome transport	25	66
1210	GO:0032481	positive regulation of type I interferon production	25	167
1211	GO:0033574	response to testosterone stimulus	25	99
1212	GO:0034620	cellular response to unfolded protein	25	82
1213	GO:0043928	exonucleolytic nuclear-transcribed mRNA catabolic process involved in deadenylation-dependent decay	25	90
1214	GO:0045010	actin nucleation	25	158
1215	GO:0045926	negative regulation of growth	25	82
1216	GO:0046835	carbohydrate phosphorylation	25	108
1217	GO:0048577	negative regulation of short-day photoperiodism, flowering	25	90
1218	GO:0050727	regulation of inflammatory response	25	169
1219	GO:0051365	cellular response to potassium ion starvation	25	96
1220	GO:0060213	positive regulation of nuclear-transcribed mRNA poly(A) tail shortening	25	122
1221	GO:0060297	regulation of sarcomere organization	25	118
1222	GO:0070374	positive regulation of ERK1 and ERK2 cascade	25	124
1223	GO:0071217	cellular response to external biotic stimulus	25	201
1224	GO:0003407	neural retina development	24	125
1225	GO:0006353	DNA-dependent transcription, termination	24	116
1226	GO:0006363	termination of RNA polymerase I transcription	24	177

1227	GO:0006520	cellular amino acid metabolic process	24	93
1228	GO:0006547	histidine metabolic process	24	126
1229	GO:0006734	NADH metabolic process	24	88
1230	GO:0006919	activation of cysteine-type endopeptidase activity involved in apoptotic process	24	52
1231	GO:0007129	synapsis	24	178
1232	GO:0007254	JNK cascade	24	81
1233	GO:0009645	response to low light intensity stimulus	24	101
1234	GO:0009650	UV protection	24	136
1235	GO:0009799	specification of symmetry	24	34
1236	GO:0010100	negative regulation of photomorphogenesis	24	152
1237	GO:0010272	response to silver ion	24	175
1238	GO:0010400	rhamnogalacturonan I side chain metabolic process	24	88
1239	GO:0010506	regulation of autophagy	24	188
1240	GO:0015074	DNA integration	24	139
1241	GO:0015886	heme transport	24	94
1242	GO:0015949	nucleobase-containing small molecule interconversion	24	187
1243	GO:0022400	regulation of rhodopsin mediated signaling pathway	24	101
1244	GO:0030198	extracellular matrix organization	24	140
1245	GO:0030900	forebrain development	24	139
1246	GO:0031349	positive regulation of defense response	24	66
1247	GO:0031396	regulation of protein ubiquitination	24	130
1248	GO:0032497	detection of lipopolysaccharide	24	90
1249	GO:0035264	multicellular organism growth	24	84
1250	GO:0038096	Fc-gamma receptor signaling pathway involved in phagocytosis	24	149
1251	GO:0043067	regulation of programmed cell death	24	76
1252	GO:0043434	response to peptide hormone stimulus	24	99
1253	GO:0045814	negative regulation of gene expression, epigenetic	24	157
1254	GO:0045862	positive regulation of proteolysis	24	158
1255	GO:0046513	ceramide biosynthetic process	24	133
1256	GO:0046677	response to antibiotic	24	50
1257	GO:0048575	short-day photoperiodism, flowering	24	128
1258	GO:0048585	negative regulation of response to stimulus	24	137
1259	GO:0050690	regulation of defense response to virus by virus	24	147
1260	GO:0050999	regulation of nitric-oxide synthase activity	24	43
1261	GO:0051131	chaperone-mediated protein complex assembly	24	115
1262	GO:0051574	positive regulation of histone H3-K9 methylation	24	104
1263	GO:0051649	establishment of localization in cell	24	123
1264	GO:0060627	regulation of vesicle-mediated transport	24	62
1265	GO:0070413	trehalose metabolism in response to stress	24	153
1266	GO:0072594	establishment of protein localization to organelle	24	49
1267	GO:0080024	indolebutyric acid metabolic process	24	41
1268	GO:0080148	negative regulation of response to water deprivation	24	124
1269	GO:0000051	urea cycle intermediate metabolic process	23	105
1270	GO:0000729	DNA double-strand break processing	23	155
1271	GO:0000904	cell morphogenesis involved in differentiation	23	81
1272	GO:0006108	malate metabolic process	23	118
1273	GO:0006610	ribosomal protein import into nucleus	23	89
1274	GO:0006754	ATP biosynthetic process	23	100
1275	GO:0006949	syncytium formation	23	52
1276	GO:0007035	vacuolar acidification	23	118
1277	GO:0007112	male meiosis cytokinesis	23	131
1278	GO:0007141	male meiosis I	23	65
1279	GO:0007338	single fertilization	23	141
1280	GO:0009089	lysine biosynthetic process via diaminopimelate	23	126
1281	GO:0009767	photosynthetic electron transport chain	23	92
1282	GO:0010047	fruit dehiscence	23	74
1283	GO:0010115	regulation of abscisic acid biosynthetic process	23	72
1284	GO:0010424	DNA methylation on cytosine within a CG sequence	23	111

1285	GO:0010458	exit from mitosis	23	179
1286	GO:0010646	regulation of cell communication	23	92
1287	GO:0015693	magnesium ion transport	23	151
1288	GO:0015825	L-serine transport	23	83
1289	GO:0015970	guanosine tetraphosphate biosynthetic process	23	72
1290	GO:0030178	negative regulation of Wnt receptor signaling pathway	23	72
1291	GO:0030970	retrograde protein transport, ER to cytosol	23	98
1292	GO:0032092	positive regulation of protein binding	23	131
1293	GO:0032781	positive regulation of ATPase activity	23	87
1294	GO:0033609	oxalate metabolic process	23	35
1295	GO:0034052	positive regulation of plant-type hypersensitive response	23	160
1296	GO:0034504	protein localization to nucleus	23	131
1297	GO:0034975	protein folding in endoplasmic reticulum	23	62
1298	GO:0042128	nitrate assimilation	23	69
1299	GO:0042754	negative regulation of circadian rhythm	23	124
1300	GO:0043254	regulation of protein complex assembly	23	31
1301	GO:0043278	response to morphine	23	144
1302	GO:0045624	positive regulation of T-helper cell differentiation	23	95
1303	GO:0046898	response to cycloheximide	23	171
1304	GO:0047484	regulation of response to osmotic stress	23	92
1305	GO:0048013	ephrin receptor signaling pathway	23	138
1306	GO:0048193	Golgi vesicle transport	23	110
1307	GO:0048480	stigma development	23	51
1308	GO:0048544	recognition of pollen	23	102
1309	GO:0050665	hydrogen peroxide biosynthetic process	23	163
1310	GO:0051591	response to cAMP	23	124
1311	GO:0052546	cell wall pectin metabolic process	23	114
1312	GO:0060070	canonical Wnt receptor signaling pathway	23	99
1313	GO:0071249	cellular response to nitrate	23	87
1314	GO:0071345	cellular response to cytokine stimulus	23	106
1315	GO:0071363	cellular response to growth factor stimulus	23	67
1316	GO:0000083	regulation of transcription involved in G1/S phase of mitotic cell cycle	22	111
1317	GO:0001756	somitogenesis	22	118
1318	GO:0006171	cAMP biosynthetic process	22	84
1319	GO:0006337	nucleosome disassembly	22	113
1320	GO:0006449	regulation of translational termination	22	176
1321	GO:0006471	protein ADP-ribosylation	22	108
1322	GO:0006515	misfolded or incompletely synthesized protein catabolic process	22	124
1323	GO:0006611	protein export from nucleus	22	71
1324	GO:0008286	insulin receptor signaling pathway	22	112
1325	GO:0008646	high-affinity hexose transport	22	66
1326	GO:0009657	plastid organization	22	77
1327	GO:0009684	indoleacetic acid biosynthetic process	22	68
1328	GO:0009831	plant-type cell wall modification involved in multidimensional cell growth	22	51
1329	GO:0009851	auxin biosynthetic process	22	55
1330	GO:0009939	positive regulation of gibberellic acid mediated signaling pathway	22	86
1331	GO:0010022	meristem determinacy	22	65
1332	GO:0010256	endomembrane system organization	22	133
1333	GO:0010332	response to gamma radiation	22	126
1334	GO:0015761	mannose transport	22	66
1335	GO:0015866	ADP transport	22	79
1336	GO:0016441	posttranscriptional gene silencing	22	117
1337	GO:0018130	heterocycle biosynthetic process	22	90
1338	GO:0019882	antigen processing and presentation	22	77
1339	GO:0030162	regulation of proteolysis	22	141
1340	GO:0030206	chondroitin sulfate biosynthetic process	22	76

1341	GO:0032088	negative regulation of NF-kappaB transcription factor activity	22	107
1342	GO:0032270	positive regulation of cellular protein metabolic process	22	88
1343	GO:0032868	response to insulin stimulus	22	80
1344	GO:0033591	response to L-ascorbic acid	22	52
1345	GO:0033962	cytoplasmic mRNA processing body assembly	22	75
1346	GO:0034080	CenH3-containing nucleosome assembly at centromere	22	89
1347	GO:0034214	protein hexamerization	22	129
1348	GO:0034765	regulation of ion transmembrane transport	22	82
1349	GO:0044403	symbiosis, encompassing mutualism through parasitism	22	53
1350	GO:0045444	fat cell differentiation	22	100
1351	GO:0045773	positive regulation of axon extension	22	87
1352	GO:0045787	positive regulation of cell cycle	22	86
1353	GO:0048838	release of seed from dormancy	22	99
1354	GO:0051016	barbed-end actin filament capping	22	122
1355	GO:0060027	convergent extension involved in gastrulation	22	65
1356	GO:0070423	nucleotide-binding oligomerization domain containing signaling pathway	22	54
1357	GO:0071260	cellular response to mechanical stimulus	22	125
1358	GO:0071285	cellular response to lithium ion	22	76
1359	GO:0071549	cellular response to dexamethasone stimulus	22	72
1360	GO:0071554	cell wall organization or biogenesis	22	97
1361	GO:0071712	ER-associated misfolded protein catabolic process	22	111
1362	GO:0080187	floral organ senescence	22	57
1363	GO:0090059	protoxylem development	22	68
1364	GO:2000032	regulation of secondary shoot formation	22	53
1365	GO:2000652	regulation of secondary cell wall biogenesis	22	90
1366	GO:2001243	negative regulation of intrinsic apoptotic signaling pathway	22	151
1367	GO:0000045	autophagic vacuole assembly	21	105
1368	GO:0000079	regulation of cyclin-dependent protein kinase activity	21	140
1369	GO:0002027	regulation of heart rate	21	113
1370	GO:0006207	'de novo' pyrimidine nucleobase biosynthetic process	21	154
1371	GO:0006400	tRNA modification	21	202
1372	GO:0006607	NLS-bearing substrate import into nucleus	21	96
1373	GO:0006805	xenobiotic metabolic process	21	50
1374	GO:0006972	hyperosmotic response	21	82
1375	GO:0007040	lysosome organization	21	162
1376	GO:0007263	nitric oxide mediated signal transduction	21	61
1377	GO:0009699	phenylpropanoid biosynthetic process	21	30
1378	GO:0009967	positive regulation of signal transduction	21	126
1379	GO:0010020	chloroplast fission	21	101
1380	GO:0010800	positive regulation of peptidyl-threonine phosphorylation	21	69
1381	GO:0015947	methane metabolic process	21	88
1382	GO:0019430	removal of superoxide radicals	21	76
1383	GO:0019521	D-gluconate metabolic process	21	84
1384	GO:0019748	secondary metabolic process	21	55
1385	GO:0030026	cellular manganese ion homeostasis	21	106
1386	GO:0030953	astral microtubule organization	21	45
1387	GO:0033138	positive regulation of peptidyl-serine phosphorylation	21	115
1388	GO:0033169	histone H3-K9 demethylation	21	134
1389	GO:0033500	carbohydrate homeostasis	21	144
1390	GO:0035307	positive regulation of protein dephosphorylation	21	57
1391	GO:0042592	homeostatic process	21	117
1392	GO:0043335	protein unfolding	21	40
1393	GO:0043392	negative regulation of DNA binding	21	84
1394	GO:0045429	positive regulation of nitric oxide biosynthetic process	21	101
1395	GO:0048598	embryonic morphogenesis	21	94
1396	GO:0048702	embryonic neurocranium morphogenesis	21	95
1397	GO:0050776	regulation of immune response	21	49

1398	GO:0051646	mitochondrion localization	21	179
1399	GO:0055075	potassium ion homeostasis	21	96
1400	GO:0061092	positive regulation of phospholipid translocation	21	114
1401	GO:0065009	regulation of molecular function	21	56
1402	GO:0070483	detection of hypoxia	21	79
1403	GO:0072358	cardiovascular system development	21	97
1404	GO:0080036	regulation of cytokinin mediated signaling pathway	21	79
1405	GO:0090057	root radial pattern formation	21	25
1406	GO:1900994	(-)-secologanin biosynthetic process	21	45
1407	GO:1901141	regulation of lignin biosynthetic process	21	91
1408	GO:2000035	regulation of stem cell division	21	135
1409	GO:2000067	regulation of root morphogenesis	21	55
1410	GO:2000069	regulation of post-embryonic root development	21	135
1411	GO:0000070	mitotic sister chromatid segregation	20	171
1412	GO:0000154	rRNA modification	20	78
1413	GO:0000387	spliceosomal snRNP assembly	20	89
1414	GO:0000920	cytokinetic cell separation	20	84
1415	GO:0001881	receptor recycling	20	82
1416	GO:0006268	DNA unwinding involved in replication	20	86
1417	GO:0006306	DNA methylation	20	137
1418	GO:0006344	maintenance of chromatin silencing	20	176
1419	GO:0006646	phosphatidylethanolamine biosynthetic process	20	212
1420	GO:0006783	heme biosynthetic process	20	118
1421	GO:0006821	chloride transport	20	95
1422	GO:0007052	mitotic spindle organization	20	132
1423	GO:0007143	female meiosis	20	99
1424	GO:0008637	apoptotic mitochondrial changes	20	62
1425	GO:0009086	methionine biosynthetic process	20	124
1426	GO:0009635	response to herbicide	20	68
1427	GO:0009743	response to carbohydrate stimulus	20	102
1428	GO:0009772	photosynthetic electron transport in photosystem II	20	104
1429	GO:0009945	radial axis specification	20	66
1430	GO:0010080	regulation of floral meristem growth	20	34
1431	GO:0010201	response to continuous far red light stimulus by the high-irradiance response system	20	106
1432	GO:0010321	regulation of vegetative phase change	20	58
1433	GO:0010358	leaf shaping	20	96
1434	GO:0010803	regulation of tumor necrosis factor-mediated signaling pathway	20	63
1435	GO:0010918	positive regulation of mitochondrial membrane potential	20	74
1436	GO:0015917	aminophospholipid transport	20	125
1437	GO:0016116	carotenoid metabolic process	20	74
1438	GO:0016337	cell-cell adhesion	20	101
1439	GO:0016559	peroxisome fission	20	142
1440	GO:0023051	regulation of signaling	20	74
1441	GO:0030245	cellulose catabolic process	20	89
1442	GO:0031525	menthol biosynthetic process	20	46
1443	GO:0032107	regulation of response to nutrient levels	20	72
1444	GO:0032212	positive regulation of telomere maintenance via telomerase	20	119
1445	GO:0032414	positive regulation of ion transmembrane transporter activity	20	107
1446	GO:0032870	cellular response to hormone stimulus	20	61
1447	GO:0042472	inner ear morphogenesis	20	137
1448	GO:0043581	mycelium development	20	92
1449	GO:0045116	protein neddylation	20	34
1450	GO:0045899	positive regulation of RNA polymerase II transcriptional preinitiation complex assembly	20	59
1451	GO:0045910	negative regulation of DNA recombination	20	141
1452	GO:0048026	positive regulation of nuclear mRNA splicing, via spliceosome	20	56

1453	GO:0048169	regulation of long-term neuronal synaptic plasticity	20	77
1454	GO:0048255	mRNA stabilization	20	72
1455	GO:0048497	maintenance of floral organ identity	20	69
1456	GO:0048508	embryonic meristem development	20	61
1457	GO:0051013	microtubule severing	20	170
1458	GO:0051973	positive regulation of telomerase activity	20	83
1459	GO:0060766	negative regulation of androgen receptor signaling pathway	20	107
1460	GO:0061157	mRNA destabilization	20	54
1461	GO:0071276	cellular response to cadmium ion	20	74
1462	GO:0071277	cellular response to calcium ion	20	80
1463	GO:0071585	detoxification of cadmium ion	20	53
1464	GO:0080164	regulation of nitric oxide metabolic process	20	33
1465	GO:2000573	positive regulation of DNA biosynthetic process	20	79
1466	GO:0000280	nuclear division	19	108
1467	GO:0000422	mitochondrion degradation	19	120
1468	GO:0000467	exonucleolytic trimming to generate mature 3'-end of 5.8S rRNA from tricistronic rRNA transcript (SSU-rRNA, 5.8S rRNA, LSU-rRNA)	19	104
1469	GO:0000472	endonucleolytic cleavage to generate mature 5'-end of SSU-rRNA from (SSU-rRNA, 5.8S rRNA, LSU-rRNA)	19	96
1470	GO:0000731	DNA synthesis involved in DNA repair	19	210
1471	GO:0001837	epithelial to mesenchymal transition	19	84
1472	GO:0001890	placenta development	19	130
1473	GO:0003091	renal water homeostasis	19	64
1474	GO:0006122	mitochondrial electron transport, ubiquinol to cytochrome c	19	46
1475	GO:0006275	regulation of DNA replication	19	92
1476	GO:0006407	rRNA export from nucleus	19	32
1477	GO:0006414	translational elongation	19	48
1478	GO:0006450	regulation of translational fidelity	19	91
1479	GO:0006621	protein retention in ER lumen	19	40
1480	GO:0006656	phosphatidylcholine biosynthetic process	19	100
1481	GO:0006695	cholesterol biosynthetic process	19	106
1482	GO:0006782	protoporphyrinogen IX biosynthetic process	19	110
1483	GO:0006829	zinc ion transport	19	87
1484	GO:0006913	nucleocytoplasmic transport	19	100
1485	GO:0007173	epidermal growth factor receptor signaling pathway	19	93
1486	GO:0007186	G-protein coupled receptor signaling pathway	19	125
1487	GO:0007224	smoothened signaling pathway	19	75
1488	GO:0007595	lactation	19	95
1489	GO:0007608	sensory perception of smell	19	67
1490	GO:0008217	regulation of blood pressure	19	119
1491	GO:0009642	response to light intensity	19	54
1492	GO:0009690	cytokinin metabolic process	19	158
1493	GO:0009819	drought recovery	19	71
1494	GO:0010050	vegetative phase change	19	78
1495	GO:0010055	atrichoblast differentiation	19	47
1496	GO:0010071	root meristem specification	19	131
1497	GO:0010497	plasmodesmata-mediated intercellular transport	19	40
1498	GO:0010555	response to mannitol stimulus	19	72
1499	GO:0010966	regulation of phosphate transport	19	38
1500	GO:0015808	L-alanine transport	19	70
1501	GO:0015810	aspartate transport	19	66
1502	GO:0015860	purine nucleoside transport	19	77
1503	GO:0015893	drug transport	19	89
1504	GO:0015969	guanosine tetraphosphate metabolic process	19	97
1505	GO:0015996	chlorophyll catabolic process	19	169
1506	GO:0016139	glycoside catabolic process	19	148
1507	GO:0016584	nucleosome positioning	19	182
1508	GO:0021987	cerebral cortex development	19	84

1509	GO:0030091	protein repair	19	66
1510	GO:0031054	pre-miRNA processing	19	83
1511	GO:0031333	negative regulation of protein complex assembly	19	81
1512	GO:0031537	regulation of anthocyanin metabolic process	19	72
1513	GO:0032981	mitochondrial respiratory chain complex I assembly	19	128
1514	GO:0033619	membrane protein proteolysis	19	97
1515	GO:0034047	regulation of protein phosphatase type 2A activity	19	71
1516	GO:0034097	response to cytokine stimulus	19	98
1517	GO:0035329	hippo signaling cascade	19	134
1518	GO:0035435	phosphate ion transmembrane transport	19	79
1519	GO:0040027	negative regulation of vulval development	19	157
1520	GO:0042048	olfactory behavior	19	130
1521	GO:0043044	ATP-dependent chromatin remodeling	19	103
1522	GO:0043651	linoleic acid metabolic process	19	151
1523	GO:0045165	cell fate commitment	19	43
1524	GO:0045332	phospholipid translocation	19	102
1525	GO:0046621	negative regulation of organ growth	19	139
1526	GO:0046827	positive regulation of protein export from nucleus	19	114
1527	GO:0050808	synapse organization	19	69
1528	GO:0051001	negative regulation of nitric-oxide synthase activity	19	84
1529	GO:0051384	response to glucocorticoid stimulus	19	97
1530	GO:0051534	negative regulation of NFAT protein import into nucleus	19	95
1531	GO:0060145	viral gene silencing in virus induced gene silencing	19	96
1532	GO:0060321	acceptance of pollen	19	149
1533	GO:0061014	positive regulation of mRNA catabolic process	19	169
1534	GO:0070555	response to interleukin-1	19	190
1535	GO:0070816	phosphorylation of RNA polymerase II C-terminal domain	19	112
1536	GO:0070935	3'-UTR-mediated mRNA stabilization	19	77
1537	GO:0071044	histone mRNA catabolic process	19	186
1538	GO:0071300	cellular response to retinoic acid	19	177
1539	GO:0071475	cellular hyperosmotic salinity response	19	30
1540	GO:0071486	cellular response to high light intensity	19	81
1541	GO:0071492	cellular response to UV-A	19	56
1542	GO:0071560	cellular response to transforming growth factor beta stimulus	19	78
1543	GO:0001947	heart looping	18	82
1544	GO:0002474	antigen processing and presentation of peptide antigen via MHC class I	18	60
1545	GO:0006091	generation of precursor metabolites and energy	18	95
1546	GO:0006265	DNA topological change	18	106
1547	GO:0006361	transcription initiation from RNA polymerase I promoter	18	99
1548	GO:0006362	transcription elongation from RNA polymerase I promoter	18	99
1549	GO:0006491	N-glycan processing	18	130
1550	GO:0006790	sulfur compound metabolic process	18	70
1551	GO:0006983	ER overload response	18	70
1552	GO:0007019	microtubule depolymerization	18	96
1553	GO:0007368	determination of left/right symmetry	18	95
1554	GO:0008089	anterograde axon cargo transport	18	99
1555	GO:0008344	adult locomotory behavior	18	83
1556	GO:0009225	nucleotide-sugar metabolic process	18	66
1557	GO:0009648	photoperiodism	18	67
1558	GO:0009662	etioplast organization	18	91
1559	GO:0010262	somatic embryogenesis	18	112
1560	GO:0010447	response to acidity	18	69
1561	GO:0010529	negative regulation of transposition	18	28
1562	GO:0010557	positive regulation of macromolecule biosynthetic process	18	70
1563	GO:0010796	regulation of multivesicular body size	18	41

1564	GO:0015809	arginine transport	18	69
1565	GO:0019752	carboxylic acid metabolic process	18	57
1566	GO:0030030	cell projection organization	18	139
1567	GO:0030177	positive regulation of Wnt receptor signaling pathway	18	144
1568	GO:0030866	cortical actin cytoskeleton organization	18	62
1569	GO:0031468	nuclear envelope reassembly	18	98
1570	GO:0031564	transcription antitermination	18	58
1571	GO:0032147	activation of protein kinase activity	18	59
1572	GO:0032206	positive regulation of telomere maintenance	18	140
1573	GO:0032570	response to progesterone stimulus	18	34
1574	GO:0032786	positive regulation of DNA-dependent transcription, elongation	18	81
1575	GO:0033120	positive regulation of RNA splicing	18	137
1576	GO:0034314	Arp2/3 complex-mediated actin nucleation	18	117
1577	GO:0034968	histone lysine methylation	18	70
1578	GO:0035265	organ growth	18	100
1579	GO:0036168	filamentous growth of a population of unicellular organisms in response to heat	18	126
1580	GO:0036170	filamentous growth of a population of unicellular organisms in response to starvation	18	88
1581	GO:0039020	pronephric nephron tubule development	18	65
1582	GO:0040009	regulation of growth rate	18	136
1583	GO:0040010	positive regulation of growth rate	18	101
1584	GO:0042176	regulation of protein catabolic process	18	69
1585	GO:0042220	response to cocaine	18	82
1586	GO:0042552	myelination	18	99
1587	GO:0043266	regulation of potassium ion transport	18	84
1588	GO:0043412	macromolecule modification	18	136
1589	GO:0043436	oxoacid metabolic process	18	36
1590	GO:0043588	skin development	18	118
1591	GO:0045739	positive regulation of DNA repair	18	81
1592	GO:0045860	positive regulation of protein kinase activity	18	108
1593	GO:0045931	positive regulation of mitotic cell cycle	18	91
1594	GO:0046329	negative regulation of JNK cascade	18	121
1595	GO:0046620	regulation of organ growth	18	73
1596	GO:0046938	phytochelatin biosynthetic process	18	116
1597	GO:0047496	vesicle transport along microtubule	18	30
1598	GO:0048576	positive regulation of short-day photoperiodism, flowering	18	52
1599	GO:0051567	histone H3-K9 methylation	18	80
1600	GO:0051593	response to folic acid	18	74
1601	GO:0055088	lipid homeostasis	18	89
1602	GO:0060341	regulation of cellular localization	18	118
1603	GO:0061158	3'-UTR-mediated mRNA destabilization	18	144
1604	GO:0070059	intrinsic apoptotic signaling pathway in response to endoplasmic reticulum stress	18	86
1605	GO:0070373	negative regulation of ERK1 and ERK2 cascade	18	78
1606	GO:0070389	chaperone cofactor-dependent protein refolding	18	48
1607	GO:0071230	cellular response to amino acid stimulus	18	107
1608	GO:0071396	cellular response to lipid	18	96
1609	GO:0071495	cellular response to endogenous stimulus	18	63
1610	GO:0071836	nectar secretion	18	53
1611	GO:0080051	cutin transport	18	50
1612	GO:0080149	sucrose induced translational repression	18	38
1613	GO:0080171	lytic vacuole organization	18	36
1614	GO:0085020	protein K6-linked ubiquitination	18	99
1615	GO:0090436	leaf pavement cell development	18	184
1616	GO:1900424	regulation of defense response to bacterium	18	124
1617	GO:1900740	positive regulation of protein insertion into mitochondrial membrane involved in apoptotic signaling pathway	18	92

1618	GO:2000008	regulation of protein localization at cell surface	18	49
1619	GO:2000030	regulation of response to red or far red light	18	33
1620	GO:2001244	positive regulation of intrinsic apoptotic signaling pathway	18	52
1621	GO:0000054	ribosomal subunit export from nucleus	17	58
1622	GO:0000470	maturation of LSU-rRNA	17	48
1623	GO:0000480	endonucleolytic cleavage in 5'-ETS of tricistronic rRNA transcript (SSU-rRNA, 5.8S rRNA, LSU-rRNA)	17	94
1624	GO:0001302	replicative cell aging	17	147
1625	GO:0001541	ovarian follicle development	17	89
1626	GO:0006002	fructose 6-phosphate metabolic process	17	106
1627	GO:0006040	amino sugar metabolic process	17	62
1628	GO:0006293	nucleotide-excision repair, preincision complex stabilization	17	187
1629	GO:0006295	nucleotide-excision repair, DNA incision, 3'-to lesion	17	187
1630	GO:0006336	DNA replication-independent nucleosome assembly	17	40
1631	GO:0006379	mRNA cleavage	17	142
1632	GO:0006535	cysteine biosynthetic process from serine	17	63
1633	GO:0006828	manganese ion transport	17	60
1634	GO:0006978	DNA damage response, signal transduction by p53 class mediator resulting in transcription of p21 class mediator	17	77
1635	GO:0007088	regulation of mitosis	17	102
1636	GO:0007179	transforming growth factor beta receptor signaling pathway	17	137
1637	GO:0007339	binding of sperm to zona pellucida	17	75
1638	GO:0007422	peripheral nervous system development	17	81
1639	GO:0007588	excretion	17	85
1640	GO:0008584	male gonad development	17	99
1641	GO:0009107	lipoate biosynthetic process	17	68
1642	GO:0009717	isoflavonoid biosynthetic process	17	23
1643	GO:0009902	chloroplast relocation	17	45
1644	GO:0009920	cell plate formation involved in plant-type cell wall biogenesis	17	57
1645	GO:0010099	regulation of photomorphogenesis	17	69
1646	GO:0010162	seed dormancy process	17	67
1647	GO:0010231	maintenance of seed dormancy	17	90
1648	GO:0010500	transmitting tissue development	17	50
1649	GO:0010564	regulation of cell cycle process	17	95
1650	GO:0010569	regulation of double-strand break repair via homologous recombination	17	115
1651	GO:0010830	regulation of myotube differentiation	17	80
1652	GO:0015827	tryptophan transport	17	63
1653	GO:0019219	regulation of nucleobase-containing compound metabolic process	17	84
1654	GO:0019745	pentacyclic triterpenoid biosynthetic process	17	111
1655	GO:0030111	regulation of Wnt receptor signaling pathway	17	63
1656	GO:0030516	regulation of axon extension	17	67
1657	GO:0030718	germ-line stem cell maintenance	17	114
1658	GO:0031000	response to caffeine	17	157
1659	GO:0031087	deadenylation-independent decapping of nuclear-transcribed mRNA	17	82
1660	GO:0031571	mitotic cell cycle G1/S transition DNA damage checkpoint	17	70
1661	GO:0033466	trans-zeatin biosynthetic process	17	60
1662	GO:0034727	piecemeal microautophagy of nucleus	17	89
1663	GO:0042275	error-free postreplication DNA repair	17	48
1664	GO:0042766	nucleosome mobilization	17	164
1665	GO:0043091	L-arginine import	17	38
1666	GO:0043153	entrainment of circadian clock by photoperiod	17	49
1667	GO:0043410	positive regulation of MAPK cascade	17	95
1668	GO:0043507	positive regulation of JUN kinase activity	17	61
1669	GO:0043562	cellular response to nitrogen levels	17	46

1670	GO:0044085	cellular component biogenesis	17	48
1671	GO:0044093	positive regulation of molecular function	17	94
1672	GO:0044314	protein K27-linked ubiquitination	17	96
1673	GO:0045487	gibberellin catabolic process	17	46
1674	GO:0045927	positive regulation of growth	17	97
1675	GO:0046256	2,4,6-trinitrotoluene catabolic process	17	26
1676	GO:0046415	urate metabolic process	17	56
1677	GO:0046482	para-aminobenzoic acid metabolic process	17	23
1678	GO:0046618	drug export	17	55
1679	GO:0048015	phosphatidylinositol-mediated signaling	17	167
1680	GO:0050434	positive regulation of viral transcription	17	72
1681	GO:0050688	regulation of defense response to virus	17	82
1682	GO:0050885	neuromuscular process controlling balance	17	62
1683	GO:0051052	regulation of DNA metabolic process	17	156
1684	GO:0051228	mitotic spindle disassembly	17	97
1685	GO:0051604	protein maturation	17	85
1686	GO:0060416	response to growth hormone stimulus	17	49
1687	GO:0061088	regulation of sequestering of zinc ion	17	102
1688	GO:0070919	production of siRNA involved in chromatin silencing by small RNA	17	95
1689	GO:0071407	cellular response to organic cyclic compound	17	142
1690	GO:0071480	cellular response to gamma radiation	17	162
1691	GO:0071482	cellular response to light stimulus	17	130
1692	GO:0072089	stem cell proliferation	17	91
1693	GO:0080117	secondary growth	17	116
1694	GO:0080169	cellular response to boron-containing substance deprivation	17	36
1695	GO:0090004	positive regulation of establishment of protein localization in plasma membrane	17	66
1696	GO:0090383	phagosome acidification	17	83
1697	GO:0000001	mitochondrion inheritance	16	73
1698	GO:0000132	establishment of mitotic spindle orientation	16	86
1699	GO:0000389	nuclear mRNA 3'-splice site recognition	16	71
1700	GO:0000578	embryonic axis specification	16	77
1701	GO:0000732	strand displacement	16	173
1702	GO:0001300	chronological cell aging	16	57
1703	GO:0001709	cell fate determination	16	112
1704	GO:0001824	blastocyst development	16	107
1705	GO:0002244	hemopoietic progenitor cell differentiation	16	73
1706	GO:0002755	MyD88-dependent toll-like receptor signaling pathway	16	73
1707	GO:0003008	system process	16	121
1708	GO:0003228	atrial cardiac muscle tissue development	16	72
1709	GO:0006073	cellular glucan metabolic process	16	47
1710	GO:0006102	isocitrate metabolic process	16	48
1711	GO:0006333	chromatin assembly or disassembly	16	104
1712	GO:0006405	RNA export from nucleus	16	95
1713	GO:0006469	negative regulation of protein kinase activity	16	79
1714	GO:0006499	N-terminal protein myristoylation	16	83
1715	GO:0006559	L-phenylalanine catabolic process	16	71
1716	GO:0006612	protein targeting to membrane	16	80
1717	GO:0006893	Golgi to plasma membrane transport	16	82
1718	GO:0007020	microtubule nucleation	16	105
1719	GO:0007205	protein kinase C-activating G-protein coupled receptor signaling pathway	16	101
1720	GO:0007613	memory	16	139
1721	GO:0008210	estrogen metabolic process	16	37
1722	GO:0009643	photosynthetic acclimation	16	46
1723	GO:0009688	abscisic acid biosynthetic process	16	79
1724	GO:0010117	photoprotection	16	38
1725	GO:0010158	abaxial cell fate specification	16	74

1726	GO:0010171	body morphogenesis	16	64
1727	GO:0010188	response to microbial phytotoxin	16	53
1728	GO:0010189	vitamin E biosynthetic process	16	60
1729	GO:0010288	response to lead ion	16	75
1730	GO:0010482	regulation of epidermal cell division	16	62
1731	GO:0010589	leaf proximal/distal pattern formation	16	84
1732	GO:0010659	cardiac muscle cell apoptotic process	16	39
1733	GO:0010994	free ubiquitin chain polymerization	16	55
1734	GO:0015746	citrate transport	16	71
1735	GO:0016051	carbohydrate biosynthetic process	16	79
1736	GO:0016241	regulation of macroautophagy	16	107
1737	GO:0016322	neuron remodeling	16	86
1738	GO:0019048	virus-host interaction	16	69
1739	GO:0019243	methylglyoxal catabolic process to D-lactate	16	72
1740	GO:0019530	taurine metabolic process	16	82
1741	GO:0030317	sperm motility	16	96
1742	GO:0030324	lung development	16	66
1743	GO:0030707	ovarian follicle cell development	16	85
1744	GO:0031122	cytoplasmic microtubule organization	16	102
1745	GO:0031398	positive regulation of protein ubiquitination	16	55
1746	GO:0032504	multicellular organism reproduction	16	31
1747	GO:0032511	late endosome to vacuole transport via multivesicular body sorting pathway	16	89
1748	GO:0032516	positive regulation of phosphoprotein phosphatase activity	16	76
1749	GO:0032787	monocarboxylic acid metabolic process	16	46
1750	GO:0033147	negative regulation of intracellular estrogen receptor signaling pathway	16	138
1751	GO:0034440	lipid oxidation	16	41
1752	GO:0042138	meiotic DNA double-strand break formation	16	80
1753	GO:0042207	styrene catabolic process	16	127
1754	GO:0043923	positive regulation by host of viral transcription	16	158
1755	GO:0043981	histone H4-K5 acetylation	16	114
1756	GO:0044283	small molecule biosynthetic process	16	101
1757	GO:0045132	meiotic chromosome segregation	16	65
1758	GO:0045653	negative regulation of megakaryocyte differentiation	16	37
1759	GO:0045665	negative regulation of neuron differentiation	16	61
1760	GO:0045719	negative regulation of glycogen biosynthetic process	16	78
1761	GO:0045730	respiratory burst	16	69
1762	GO:0045786	negative regulation of cell cycle	16	70
1763	GO:0045851	pH reduction	16	21
1764	GO:0045901	positive regulation of translational elongation	16	51
1765	GO:0048024	regulation of nuclear mRNA splicing, via spliceosome	16	62
1766	GO:0048149	behavioral response to ethanol	16	89
1767	GO:0048530	fruit morphogenesis	16	85
1768	GO:0051050	positive regulation of transport	16	98
1769	GO:0051170	nuclear import	16	48
1770	GO:0051208	sequestering of calcium ion	16	43
1771	GO:0051239	regulation of multicellular organismal process	16	52
1772	GO:0051246	regulation of protein metabolic process	16	68
1773	GO:0051781	positive regulation of cell division	16	67
1774	GO:0051881	regulation of mitochondrial membrane potential	16	42
1775	GO:0052325	cell wall pectin biosynthetic process	16	30
1776	GO:0055081	anion homeostasis	16	93
1777	GO:0055093	response to hyperoxia	16	77
1778	GO:0060429	epithelium development	16	70
1779	GO:0061024	membrane organization	16	71
1780	GO:0071042	nuclear polyadenylation-dependent mRNA catabolic process	16	101
1781	GO:0071280	cellular response to copper ion	16	99
1782	GO:0071366	cellular response to indolebutyric acid stimulus	16	128

1783	GO:0080155	regulation of double fertilization forming a zygote and endosperm	16	72
1784	GO:0097327	response to antineoplastic agent	16	62
1785	GO:1900103	positive regulation of endoplasmic reticulum unfolded protein response	16	51
1786	GO:1901137	carbohydrate derivative biosynthetic process	16	70
1787	GO:2000134	negative regulation of G1/S transition of mitotic cell cycle	16	115
1788	GO:0000188	inactivation of MAPK activity	15	56
1789	GO:0000244	assembly of spliceosomal tri-snRNP	15	66
1790	GO:0003281	ventricular septum development	15	73
1791	GO:0006075	(1->3)-beta-D-glucan biosynthetic process	15	132
1792	GO:0006110	regulation of glycolysis	15	89
1793	GO:0006273	lagging strand elongation	15	140
1794	GO:0006282	regulation of DNA repair	15	121
1795	GO:0006312	mitotic recombination	15	135
1796	GO:0006506	GPI anchor biosynthetic process	15	162
1797	GO:0006657	CDP-choline pathway	15	89
1798	GO:0006769	nicotinamide metabolic process	15	81
1799	GO:0007051	spindle organization	15	158
1800	GO:0007498	mesoderm development	15	54
1801	GO:0008203	cholesterol metabolic process	15	131
1802	GO:0008298	intracellular mRNA localization	15	67
1803	GO:0008544	epidermis development	15	82
1804	GO:0009056	catabolic process	15	25
1805	GO:0009116	nucleoside metabolic process	15	101
1806	GO:0009231	riboflavin biosynthetic process	15	58
1807	GO:0009240	isopentenyl diphosphate biosynthetic process	15	61
1808	GO:0009608	response to symbiont	15	43
1809	GO:0009652	thigmotropism	15	66
1810	GO:0009685	gibberellin metabolic process	15	37
1811	GO:0009691	cytokinin biosynthetic process	15	79
1812	GO:0010069	zygote asymmetric cytokinesis in embryo sac	15	38
1813	GO:0010225	response to UV-C	15	132
1814	GO:0010271	regulation of chlorophyll catabolic process	15	81
1815	GO:0010390	histone monoubiquitination	15	67
1816	GO:0010508	positive regulation of autophagy	15	64
1817	GO:0010801	negative regulation of peptidyl-threonine phosphorylation	15	59
1818	GO:0010928	regulation of auxin mediated signaling pathway	15	51
1819	GO:0015747	urate transport	15	47
1820	GO:0016558	protein import into peroxisome matrix	15	116
1821	GO:0016574	histone ubiquitination	15	66
1822	GO:0016578	histone deubiquitination	15	101
1823	GO:0018205	peptidyl-lysine modification	15	55
1824	GO:0019389	glucuronoside metabolic process	15	47
1825	GO:0019985	translesion synthesis	15	76
1826	GO:0030048	actin filament-based movement	15	129
1827	GO:0030335	positive regulation of cell migration	15	48
1828	GO:0030431	sleep	15	84
1829	GO:0030437	ascospore formation	15	48
1830	GO:0030509	BMP signaling pathway	15	92
1831	GO:0031116	positive regulation of microtubule polymerization	15	76
1832	GO:0031505	fungal-type cell wall organization	15	74
1833	GO:0031640	killing of cells of other organism	15	39
1834	GO:0032456	endocytic recycling	15	51
1835	GO:0032491	detection of molecule of fungal origin	15	58
1836	GO:0034051	negative regulation of plant-type hypersensitive response	15	76
1837	GO:0035278	negative regulation of translation involved in gene silencing by miRNA	15	34

1838	GO:0035518	histone H2A monoubiquitination	15	102
1839	GO:0035999	tetrahydrofolate interconversion	15	51
1840	GO:0042274	ribosomal small subunit biogenesis	15	99
1841	GO:0042313	protein kinase C deactivation	15	59
1842	GO:0042733	embryonic digit morphogenesis	15	120
1843	GO:0042814	monopolar cell growth	15	67
1844	GO:0044248	cellular catabolic process	15	81
1845	GO:0045003	double-strand break repair via synthesis-dependent strand annealing	15	163
1846	GO:0045218	zonula adherens maintenance	15	85
1847	GO:0045292	nuclear mRNA cis splicing, via spliceosome	15	55
1848	GO:0045740	positive regulation of DNA replication	15	53
1849	GO:0045842	positive regulation of mitotic metaphase/anaphase transition	15	44
1850	GO:0046483	heterocycle metabolic process	15	77
1851	GO:0046497	nicotinate nucleotide metabolic process	15	70
1852	GO:0046579	positive regulation of Ras protein signal transduction	15	48
1853	GO:0048002	antigen processing and presentation of peptide antigen	15	56
1854	GO:0048457	floral whorl morphogenesis	15	82
1855	GO:0048878	chemical homeostasis	15	48
1856	GO:0050714	positive regulation of protein secretion	15	76
1857	GO:0050891	multicellular organismal water homeostasis	15	102
1858	GO:0051173	positive regulation of nitrogen compound metabolic process	15	52
1859	GO:0051241	negative regulation of multicellular organismal process	15	46
1860	GO:0051645	Golgi localization	15	171
1861	GO:0060041	retina development in camera-type eye	15	64
1862	GO:0060136	embryonic process involved in female pregnancy	15	47
1863	GO:0060151	peroxisome localization	15	171
1864	GO:0060996	dendritic spine development	15	91
1865	GO:0070193	synaptonemal complex organization	15	81
1866	GO:0070676	intralumenal vesicle formation	15	37
1867	GO:0070940	dephosphorylation of RNA polymerase II C-terminal domain	15	99
1868	GO:0071157	negative regulation of cell cycle arrest	15	40
1869	GO:0071364	cellular response to epidermal growth factor stimulus	15	68
1870	GO:0071479	cellular response to ionizing radiation	15	117
1871	GO:0080006	internode patterning	15	82
1872	GO:0080134	regulation of response to stress	15	81
1873	GO:0080135	regulation of cellular response to stress	15	91
1874	GO:0080142	regulation of salicylic acid biosynthetic process	15	102
1875	GO:0090136	epithelial cell-cell adhesion	15	85
1876	GO:0090344	negative regulation of cell aging	15	63
1877	GO:0090398	cellular senescence	15	71
1878	GO:1900436	positive regulation of filamentous growth of a population of unicellular organisms in response to starvation	15	106
1879	GO:2000463	positive regulation of excitatory postsynaptic membrane potential	15	71
1880	GO:2000693	positive regulation of seed maturation	15	33
1881	GO:2001237	negative regulation of extrinsic apoptotic signaling pathway	15	94
1882	GO:0000056	ribosomal small subunit export from nucleus	14	57
1883	GO:0000436	carbon catabolite activation of transcription from RNA polymerase II promoter	14	38
1884	GO:0001172	transcription, RNA-dependent	14	57
1885	GO:0001568	blood vessel development	14	56
1886	GO:0001570	vasculogenesis	14	89
1887	GO:0001954	positive regulation of cell-matrix adhesion	14	64
1888	GO:0002009	morphogenesis of an epithelium	14	48
1889	GO:0005977	glycogen metabolic process	14	54
1890	GO:0006307	DNA dealkylation involved in DNA repair	14	52

1891	GO:0006564	L-serine biosynthetic process	14	72
1892	GO:0006672	ceramide metabolic process	14	42
1893	GO:0006696	ergosterol biosynthetic process	14	59
1894	GO:0006750	glutathione biosynthetic process	14	43
1895	GO:0006895	Golgi to endosome transport	14	84
1896	GO:0007094	mitotic cell cycle spindle assembly checkpoint	14	106
1897	GO:0007223	Wnt receptor signaling pathway, calcium modulating pathway	14	31
1898	GO:0007417	central nervous system development	14	73
1899	GO:0008207	C21-steroid hormone metabolic process	14	25
1900	GO:0009103	lipopolysaccharide biosynthetic process	14	75
1901	GO:0009252	peptidoglycan biosynthetic process	14	87
1902	GO:0009314	response to radiation	14	70
1903	GO:0009616	virus induced gene silencing	14	71
1904	GO:0009854	oxidative photosynthetic carbon pathway	14	60
1905	GO:0010086	embryonic root morphogenesis	14	107
1906	GO:0010264	myo-inositol hexakisphosphate biosynthetic process	14	105
1907	GO:0010421	hydrogen peroxide-mediated programmed cell death	14	56
1908	GO:0010440	stomatal lineage progression	14	40
1909	GO:0010842	retina layer formation	14	88
1910	GO:0010977	negative regulation of neuron projection development	14	83
1911	GO:0014066	regulation of phosphatidylinositol 3-kinase cascade	14	46
1912	GO:0015677	copper ion import	14	54
1913	GO:0015840	urea transport	14	35
1914	GO:0016050	vesicle organization	14	63
1915	GO:0016239	positive regulation of macroautophagy	14	95
1916	GO:0018106	peptidyl-histidine phosphorylation	14	111
1917	GO:0018991	oviposition	14	41
1918	GO:0019605	butyrate metabolic process	14	18
1919	GO:0022904	respiratory electron transport chain	14	152
1920	GO:0030050	vesicle transport along actin filament	14	84
1921	GO:0030336	negative regulation of cell migration	14	127
1922	GO:0030513	positive regulation of BMP signaling pathway	14	55
1923	GO:0031100	organ regeneration	14	58
1924	GO:0031399	regulation of protein modification process	14	114
1925	GO:0031507	heterochromatin assembly	14	95
1926	GO:0031954	positive regulation of protein autophosphorylation	14	48
1927	GO:0032480	negative regulation of type I interferon production	14	34
1928	GO:0032502	developmental process	14	66
1929	GO:0032940	secretion by cell	14	38
1930	GO:0033206	cytokinesis after meiosis	14	36
1931	GO:0033211	adiponectin-mediated signaling pathway	14	88
1932	GO:0033344	cholesterol efflux	14	33
1933	GO:0033683	nucleotide-excision repair, DNA incision	14	85
1934	GO:0035066	positive regulation of histone acetylation	14	66
1935	GO:0035295	tube development	14	66
1936	GO:0035335	peptidyl-tyrosine dephosphorylation	14	78
1937	GO:0040022	feminization of hermaphroditic germ-line	14	60
1938	GO:0042214	terpene metabolic process	14	31
1939	GO:0042255	ribosome assembly	14	70
1940	GO:0042572	retinol metabolic process	14	62
1941	GO:0043171	peptide catabolic process	14	50
1942	GO:0043388	positive regulation of DNA binding	14	77
1943	GO:0043968	histone H2A acetylation	14	62
1944	GO:0045055	regulated secretory pathway	14	99
1945	GO:0045736	negative regulation of cyclin-dependent protein kinase activity	14	77
1946	GO:0045770	positive regulation of asymmetric cell division	14	20
1947	GO:0045935	positive regulation of nucleobase-containing compound metabolic process	14	85

1948	GO:0046654	tetrahydrofolate biosynthetic process	14	86
1949	GO:0048066	developmental pigmentation	14	38
1950	GO:0048103	somatic stem cell division	14	20
1951	GO:0048442	sepal development	14	40
1952	GO:0048565	digestive tract development	14	61
1953	GO:0050679	positive regulation of epithelial cell proliferation	14	54
1954	GO:0050830	defense response to Gram-positive bacterium	14	55
1955	GO:0051085	chaperone mediated protein folding requiring cofactor	14	27
1956	GO:0051103	DNA ligation involved in DNA repair	14	148
1957	GO:0051216	cartilage development	14	63
1958	GO:0051223	regulation of protein transport	14	40
1959	GO:0051291	protein heterooligomerization	14	71
1960	GO:0051297	centrosome organization	14	103
1961	GO:0051343	positive regulation of cyclic-nucleotide phosphodiesterase activity	14	24
1962	GO:0051764	actin crosslink formation	14	128
1963	GO:0051788	response to misfolded protein	14	47
1964	GO:0051898	negative regulation of protein kinase B signaling cascade	14	69
1965	GO:0051983	regulation of chromosome segregation	14	102
1966	GO:0060359	response to ammonium ion	14	43
1967	GO:0060765	regulation of androgen receptor signaling pathway	14	97
1968	GO:0060776	simple leaf morphogenesis	14	27
1969	GO:0060976	coronary vasculature development	14	69
1970	GO:0065003	macromolecular complex assembly	14	124
1971	GO:0070076	histone lysine demethylation	14	63
1972	GO:0070262	peptidyl-serine dephosphorylation	14	64
1973	GO:0070863	positive regulation of protein exit from endoplasmic reticulum	14	59
1974	GO:0070932	histone H3 deacetylation	14	75
1975	GO:0071323	cellular response to chitin	14	63
1976	GO:0071421	manganese ion transmembrane transport	14	55
1977	GO:0072359	circulatory system development	14	37
1978	GO:0080170	hydrogen peroxide transmembrane transport	14	28
1979	GO:0090355	positive regulation of auxin metabolic process	14	30
1980	GO:0090358	positive regulation of tryptophan metabolic process	14	30
1981	GO:1900026	positive regulation of substrate adhesion-dependent cell spreading	14	40
1982	GO:1900087	positive regulation of G1/S transition of mitotic cell cycle	14	103
1983	GO:0000011	vacuole inheritance	13	64
1984	GO:0000350	generation of catalytic spliceosome for second transesterification step	13	55
1985	GO:0000466	maturation of 5.8S rRNA from tricistronic rRNA transcript (SSU-rRNA, 5.8S rRNA, LSU-rRNA)	13	46
1986	GO:0000712	resolution of meiotic recombination intermediates	13	119
1987	GO:0002239	response to oomycetes	13	23
1988	GO:0002576	platelet degranulation	13	20
1989	GO:0002752	cell surface pattern recognition receptor signaling pathway	13	56
1990	GO:0005513	detection of calcium ion	13	42
1991	GO:0006272	leading strand elongation	13	77
1992	GO:0006526	arginine biosynthetic process	13	72
1993	GO:0006537	glutamate biosynthetic process	13	74
1994	GO:0006809	nitric oxide biosynthetic process	13	45
1995	GO:0006959	humoral immune response	13	80
1996	GO:0007026	negative regulation of microtubule depolymerization	13	135
1997	GO:0007031	peroxisome organization	13	117
1998	GO:0007076	mitotic chromosome condensation	13	61
1999	GO:0007099	centriole replication	13	111
2000	GO:0007118	budding cell apical bud growth	13	93
2001	GO:0007163	establishment or maintenance of cell polarity	13	58

2002	GO:0008016	regulation of heart contraction	13	72
2003	GO:0008216	spermidine metabolic process	13	31
2004	GO:0008652	cellular amino acid biosynthetic process	13	56
2005	GO:0009423	chorismate biosynthetic process	13	27
2006	GO:0009765	photosynthesis, light harvesting	13	37
2007	GO:0009801	cinnamic acid ester metabolic process	13	25
2008	GO:0009820	alkaloid metabolic process	13	55
2009	GO:0010014	meristem initiation	13	64
2010	GO:0010163	high-affinity potassium ion import	13	72
2011	GO:0010165	response to X-ray	13	147
2012	GO:0010206	photosystem II repair	13	86
2013	GO:0010215	cellulose microfibril organization	13	63
2014	GO:0010304	PSII associated light-harvesting complex II catabolic process	13	82
2015	GO:0010388	cullin deneddylation	13	41
2016	GO:0010405	arabinogalactan protein metabolic process	13	53
2017	GO:0010507	negative regulation of autophagy	13	51
2018	GO:0010597	green leaf volatile biosynthetic process	13	24
2019	GO:0010686	tetracyclic triterpenoid biosynthetic process	13	69
2020	GO:0010881	regulation of cardiac muscle contraction by regulation of the release of sequestered calcium ion	13	38
2021	GO:0015012	heparan sulfate proteoglycan biosynthetic process	13	58
2022	GO:0015790	UDP-xylose transport	13	106
2023	GO:0015802	basic amino acid transport	13	50
2024	GO:0016123	xanthophyll biosynthetic process	13	99
2025	GO:0018258	protein O-linked glycosylation via hydroxyproline	13	53
2026	GO:0018401	peptidyl-proline hydroxylation to 4-hydroxy-L-proline	13	63
2027	GO:0019260	1,2-dichloroethane catabolic process	13	52
2028	GO:0021549	cerebellum development	13	23
2029	GO:0030007	cellular potassium ion homeostasis	13	46
2030	GO:0030042	actin filament depolymerization	13	33
2031	GO:0030397	membrane disassembly	13	37
2032	GO:0030448	hyphal growth	13	79
2033	GO:0030466	chromatin silencing at silent mating-type cassette	13	65
2034	GO:0030488	tRNA methylation	13	67
2035	GO:0030639	polyketide biosynthetic process	13	52
2036	GO:0030912	response to deep water	13	29
2037	GO:0031536	positive regulation of exit from mitosis	13	30
2038	GO:0031627	telomeric loop formation	13	80
2039	GO:0032101	regulation of response to external stimulus	13	46
2040	GO:0032438	melanosome organization	13	73
2041	GO:0032499	detection of peptidoglycan	13	56
2042	GO:0032760	positive regulation of tumor necrosis factor production	13	83
2043	GO:0033043	regulation of organelle organization	13	49
2044	GO:0034260	negative regulation of GTPase activity	13	73
2045	GO:0034720	histone H3-K4 demethylation	13	102
2046	GO:0035458	cellular response to interferon-beta	13	62
2047	GO:0035904	aorta development	13	67
2048	GO:0042177	negative regulation of protein catabolic process	13	61
2049	GO:0042256	mature ribosome assembly	13	38
2050	GO:0043010	camera-type eye development	13	79
2051	GO:0043647	inositol phosphate metabolic process	13	35
2052	GO:0043982	histone H4-K8 acetylation	13	91
2053	GO:0043984	histone H4-K16 acetylation	13	91
2054	GO:0044027	hypermethylation of CpG island	13	64
2055	GO:0044182	filamentous growth of a population of unicellular organisms	13	45
2056	GO:0044458	motile cilium assembly	13	68
2057	GO:0045014	negative regulation of transcription by glucose	13	62
2058	GO:0045022	early endosome to late endosome transport	13	89

2059	GO:0045921	positive regulation of exocytosis	13	61
2060	GO:0046656	folic acid biosynthetic process	13	94
2061	GO:0046785	microtubule polymerization	13	59
2062	GO:0046916	cellular transition metal ion homeostasis	13	25
2063	GO:0046950	cellular ketone body metabolic process	13	45
2064	GO:0048010	vascular endothelial growth factor receptor signaling pathway	13	46
2065	GO:0048168	regulation of neuronal synaptic plasticity	13	56
2066	GO:0048254	snoRNA localization	13	52
2067	GO:0048268	clathrin coat assembly	13	99
2068	GO:0048283	indeterminate inflorescence morphogenesis	13	45
2069	GO:0048358	mucilage pectin biosynthetic process	13	111
2070	GO:0048557	embryonic digestive tract morphogenesis	13	133
2071	GO:0048568	embryonic organ development	13	70
2072	GO:0048666	neuron development	13	57
2073	GO:0050908	detection of light stimulus involved in visual perception	13	76
2074	GO:0051049	regulation of transport	13	89
2075	GO:0051174	regulation of phosphorus metabolic process	13	56
2076	GO:0051258	protein polymerization	13	47
2077	GO:0051402	neuron apoptotic process	13	35
2078	GO:0051457	maintenance of protein location in nucleus	13	74
2079	GO:0051938	L-glutamate import	13	27
2080	GO:0052576	carbohydrate storage	13	31
2081	GO:0055065	metal ion homeostasis	13	45
2082	GO:0060612	adipose tissue development	13	53
2083	GO:0060828	regulation of canonical Wnt receptor signaling pathway	13	59
2084	GO:0060862	negative regulation of floral organ abscission	13	26
2085	GO:0061062	regulation of nematode larval development	13	31
2086	GO:0061136	regulation of proteasomal protein catabolic process	13	36
2087	GO:0061416	regulation of transcription from RNA polymerase II promoter in response to salt stress	13	69
2088	GO:0070814	hydrogen sulfide biosynthetic process	13	61
2089	GO:0070914	UV-damage excision repair	13	124
2090	GO:0070933	histone H4 deacetylation	13	72
2091	GO:0071109	superior temporal gyrus development	13	61
2092	GO:0071446	cellular response to salicylic acid stimulus	13	37
2093	GO:0071468	cellular response to acidity	13	39
2094	GO:0071596	ubiquitin-dependent protein catabolic process via the N-end rule pathway	13	56
2095	GO:0071669	plant-type cell wall organization or biogenesis	13	30
2096	GO:0071894	histone H2B conserved C-terminal lysine ubiquitination	13	110
2097	GO:0080182	histone H3-K4 trimethylation	13	66
2098	GO:0080186	developmental vegetative growth	13	44
2099	GO:0097352	autophagic vacuole maturation	13	36
2100	GO:2000036	regulation of stem cell maintenance	13	168
2101	GO:2000241	regulation of reproductive process	13	27
2102	GO:2000737	negative regulation of stem cell differentiation	13	79
2103	GO:0000059	protein import into nucleus, docking	12	77
2104	GO:0000278	mitotic cell cycle	12	67
2105	GO:0000479	endonucleolytic cleavage of tricistronic rRNA transcript (SSU-rRNA, 5.8S rRNA, LSU-rRNA)	12	30
2106	GO:0000973	posttranscriptional tethering of RNA polymerase II gene DNA at nuclear periphery	12	63
2107	GO:0001662	behavioral fear response	12	52
2108	GO:0001702	gastrulation with mouth forming second	12	101
2109	GO:0001843	neural tube closure	12	78
2110	GO:0002768	immune response-regulating cell surface receptor signaling pathway	12	23
2111	GO:0003011	involuntary skeletal muscle contraction	12	75
2112	GO:0003351	epithelial cilium movement	12	55

2113	GO:0006014	D-ribose metabolic process	12	39
2114	GO:0006123	mitochondrial electron transport, cytochrome c to oxygen	12	54
2115	GO:0006403	RNA localization	12	98
2116	GO:0006691	leukotriene metabolic process	12	41
2117	GO:0006777	Mo-molybdopterin cofactor biosynthetic process	12	68
2118	GO:0006820	anion transport	12	49
2119	GO:0006835	dicarboxylic acid transport	12	30
2120	GO:0006873	cellular ion homeostasis	12	36
2121	GO:0006875	cellular metal ion homeostasis	12	78
2122	GO:0006928	cellular component movement	12	61
2123	GO:0007021	tubulin complex assembly	12	70
2124	GO:0007084	mitotic nuclear envelope reassembly	12	74
2125	GO:0007268	synaptic transmission	12	54
2126	GO:0007294	germarium-derived oocyte fate determination	12	46
2127	GO:0007298	border follicle cell migration	12	78
2128	GO:0007405	neuroblast proliferation	12	76
2129	GO:0007520	myoblast fusion	12	62
2130	GO:0007565	female pregnancy	12	56
2131	GO:0008209	androgen metabolic process	12	33
2132	GO:0008295	spermidine biosynthetic process	12	44
2133	GO:0008585	female gonad development	12	68
2134	GO:0009085	lysine biosynthetic process	12	93
2135	GO:0009399	nitrogen fixation	12	29
2136	GO:0009410	response to xenobiotic stimulus	12	67
2137	GO:0009629	response to gravity	12	76
2138	GO:0009957	epidermal cell fate specification	12	88
2139	GO:0010021	amylopectin biosynthetic process	12	108
2140	GO:0010041	response to iron(III) ion	12	39
2141	GO:0010155	regulation of proton transport	12	52
2142	GO:0010259	multicellular organismal aging	12	70
2143	GO:0010586	miRNA metabolic process	12	51
2144	GO:0010606	positive regulation of cytoplasmic mRNA processing body assembly	12	132
2145	GO:0010667	negative regulation of cardiac muscle cell apoptotic process	12	20
2146	GO:0010824	regulation of centrosome duplication	12	52
2147	GO:0010939	regulation of necrotic cell death	12	32
2148	GO:0010996	response to auditory stimulus	12	75
2149	GO:0014911	positive regulation of smooth muscle cell migration	12	52
2150	GO:0015695	organic cation transport	12	26
2151	GO:0015760	glucose-6-phosphate transport	12	37
2152	GO:0015839	cadaverine transport	12	48
2153	GO:0015851	nucleobase transport	12	49
2154	GO:0016079	synaptic vesicle exocytosis	12	62
2155	GO:0017156	calcium ion-dependent exocytosis	12	37
2156	GO:0018885	carbon tetrachloride metabolic process	12	27
2157	GO:0019305	dTDP-rhamnose biosynthetic process	12	49
2158	GO:0019344	cysteine biosynthetic process	12	56
2159	GO:0019509	L-methionine salvage from methylthioadenosine	12	77
2160	GO:0022414	reproductive process	12	48
2161	GO:0030168	platelet activation	12	82
2162	GO:0030174	regulation of DNA-dependent DNA replication initiation	12	66
2163	GO:0030216	keratinocyte differentiation	12	88
2164	GO:0030217	T cell differentiation	12	40
2165	GO:0030388	fructose 1,6-bisphosphate metabolic process	12	34
2166	GO:0030435	sporulation resulting in formation of a cellular spore	12	68
2167	GO:0030717	karyosome formation	12	99
2168	GO:0031167	rRNA methylation	12	40
2169	GO:0032103	positive regulation of response to external stimulus	12	49

2170	GO:0032196	transposition	12	71
2171	GO:0032269	negative regulation of cellular protein metabolic process	12	49
2172	GO:0032435	negative regulation of proteasomal ubiquitin-dependent protein catabolic process	12	79
2173	GO:0032526	response to retinoic acid	12	84
2174	GO:0033160	positive regulation of protein import into nucleus, translocation	12	60
2175	GO:0033227	dsRNA transport	12	45
2176	GO:0034392	negative regulation of smooth muscle cell apoptotic process	12	55
2177	GO:0034475	U4 snRNA 3'-end processing	12	78
2178	GO:0035092	sperm chromatin condensation	12	77
2179	GO:0035519	protein K29-linked ubiquitination	12	64
2180	GO:0036010	protein localization in endosome	12	53
2181	GO:0036018	cellular response to erythropoietin	12	55
2182	GO:0036244	cellular response to neutral pH	12	77
2183	GO:0040025	vulval development	12	25
2184	GO:0040039	inductive cell migration	12	36
2185	GO:0042148	strand invasion	12	73
2186	GO:0042353	fucose biosynthetic process	12	68
2187	GO:0042616	paclitaxel metabolic process	12	21
2188	GO:0042732	D-xylose metabolic process	12	72
2189	GO:0042755	eating behavior	12	75
2190	GO:0043001	Golgi to plasma membrane protein transport	12	26
2191	GO:0043280	positive regulation of cysteine-type endopeptidase activity involved in apoptotic process	12	69
2192	GO:0043922	negative regulation by host of viral transcription	12	70
2193	GO:0044070	regulation of anion transport	12	44
2194	GO:0044087	regulation of cellular component biogenesis	12	64
2195	GO:0045038	protein import into chloroplast thylakoid membrane	12	63
2196	GO:0045676	regulation of R7 cell differentiation	12	64
2197	GO:0045903	positive regulation of translational fidelity	12	28
2198	GO:0046246	terpene biosynthetic process	12	17
2199	GO:0046274	lignin catabolic process	12	16
2200	GO:0046488	phosphatidylinositol metabolic process	12	68
2201	GO:0046500	S-adenosylmethionine metabolic process	12	49
2202	GO:0046520	sphingoid biosynthetic process	12	27
2203	GO:0046655	folic acid metabolic process	12	63
2204	GO:0048025	negative regulation of nuclear mRNA splicing, via spliceosome	12	92
2205	GO:0048132	female germ-line stem cell division	12	64
2206	GO:0048232	male gamete generation	12	48
2207	GO:0048387	negative regulation of retinoic acid receptor signaling pathway	12	100
2208	GO:0048439	flower morphogenesis	12	62
2209	GO:0048638	regulation of developmental growth	12	49
2210	GO:0048747	muscle fiber development	12	69
2211	GO:0048826	cotyledon morphogenesis	12	59
2212	GO:0050774	negative regulation of dendrite morphogenesis	12	55
2213	GO:0050877	neurological system process	12	56
2214	GO:0050884	neuromuscular process controlling posture	12	75
2215	GO:0050890	cognition	12	39
2216	GO:0051247	positive regulation of protein metabolic process	12	32
2217	GO:0051254	positive regulation of RNA metabolic process	12	39
2218	GO:0051502	diterpene phytoalexin biosynthetic process	12	22
2219	GO:0051504	diterpene phytoalexin precursor biosynthetic process pathway	12	29
2220	GO:0051693	actin filament capping	12	110
2221	GO:0051897	positive regulation of protein kinase B signaling cascade	12	95
2222	GO:0052542	defense response by callose deposition	12	85
2223	GO:0060052	neurofilament cytoskeleton organization	12	75

2224	GO:0060236	regulation of mitotic spindle organization	12	53
2225	GO:0060324	face development	12	56
2226	GO:0060384	innervation	12	43
2227	GO:0070207	protein homotrimerization	12	56
2228	GO:0070584	mitochondrion morphogenesis	12	82
2229	GO:0070734	histone H3-K27 methylation	12	67
2230	GO:0071035	nuclear polyadenylation-dependent rRNA catabolic process	12	89
2231	GO:0071051	polyadenylation-dependent snoRNA 3'-end processing	12	70
2232	GO:0071214	cellular response to abiotic stimulus	12	28
2233	GO:0071282	cellular response to iron(II) ion	12	55
2234	GO:0071287	cellular response to manganese ion	12	45
2235	GO:0071397	cellular response to cholesterol	12	97
2236	GO:0071871	response to epinephrine stimulus	12	55
2237	GO:0071901	negative regulation of protein serine/threonine kinase activity	12	29
2238	GO:0080026	response to indolebutyric acid stimulus	12	47
2239	GO:0080037	negative regulation of cytokinin mediated signaling pathway	12	19
2240	GO:0080129	proteasome core complex assembly	12	36
2241	GO:0080160	selenate transport	12	42
2242	GO:0080172	petal epidermis patterning	12	39
2243	GO:0080173	male-female gamete recognition during double fertilization	12	52
2244	GO:1900442	positive regulation of filamentous growth of a population of unicellular organisms in response to neutral pH	12	77
2245	GO:1900445	positive regulation of filamentous growth of a population of unicellular organisms in response to biotic stimulus	12	57
2246	GO:1901016	regulation of potassium ion transmembrane transporter activity	12	49
2247	GO:1901135	carbohydrate derivative metabolic process	12	47
2248	GO:1901183	positive regulation of camalexin biosynthetic process	12	53
2249	GO:2000033	regulation of seed dormancy process	12	25
2250	GO:2000306	positive regulation of photomorphogenesis	12	25
2251	GO:2000762	regulation of phenylpropanoid metabolic process	12	73
2252	GO:2001223	negative regulation of neuron migration	12	55
2253	GO:0000055	ribosomal large subunit export from nucleus	11	58
2254	GO:0000186	activation of MAPKK activity	11	59
2255	GO:0000390	spliceosomal complex disassembly	11	46
2256	GO:0000492	box C/D snoRNP assembly	11	33
2257	GO:0000741	karyogamy	11	67
2258	GO:0001410	chlamydospore formation	11	90
2259	GO:0001575	globoside metabolic process	11	53
2260	GO:0001736	establishment of planar polarity	11	99
2261	GO:0002082	regulation of oxidative phosphorylation	11	33
2262	GO:0002682	regulation of immune system process	11	40
2263	GO:0003009	skeletal muscle contraction	11	16
2264	GO:0005979	regulation of glycogen biosynthetic process	11	40
2265	GO:0006080	substituted mannan metabolic process	11	21
2266	GO:0006105	succinate metabolic process	11	53
2267	GO:0006189	'de novo' IMP biosynthetic process	11	44
2268	GO:0006221	pyrimidine nucleotide biosynthetic process	11	95
2269	GO:0006301	postreplication repair	11	93
2270	GO:0006376	mRNA splice site selection	11	97
2271	GO:0006518	peptide metabolic process	11	83
2272	GO:0006591	ornithine metabolic process	11	27
2273	GO:0006616	SRP-dependent cotranslational protein targeting to membrane, translocation	11	34
2274	GO:0006793	phosphorus metabolic process	11	49
2275	GO:0006808	regulation of nitrogen utilization	11	24

2276	GO:0006863	purine nucleobase transport	11	47
2277	GO:0006971	hypotonic response	11	18
2278	GO:0006998	nuclear envelope organization	11	33
2279	GO:0007079	mitotic chromosome movement towards spindle pole	11	62
2280	GO:0007124	pseudohyphal growth	11	60
2281	GO:0007212	dopamine receptor signaling pathway	11	55
2282	GO:0007265	Ras protein signal transduction	11	64
2283	GO:0007269	neurotransmitter secretion	11	45
2284	GO:0007317	regulation of pole plasm oskar mRNA localization	11	22
2285	GO:0008299	isoprenoid biosynthetic process	11	54
2286	GO:0008407	chaeta morphogenesis	11	59
2287	GO:0009113	purine nucleobase biosynthetic process	11	30
2288	GO:0009303	rRNA transcription	11	77
2289	GO:0009448	gamma-aminobutyric acid metabolic process	11	46
2290	GO:0010063	positive regulation of trichoblast fate specification	11	95
2291	GO:0010186	positive regulation of cellular defense response	11	14
2292	GO:0010226	response to lithium ion	11	26
2293	GO:0010310	regulation of hydrogen peroxide metabolic process	11	69
2294	GO:0010344	seed oilbody biogenesis	11	37
2295	GO:0010362	negative regulation of anion channel activity by blue light	11	46
2296	GO:0010499	proteasomal ubiquitin-independent protein catabolic process	11	17
2297	GO:0010600	regulation of auxin biosynthetic process	11	51
2298	GO:0010608	posttranscriptional regulation of gene expression	11	70
2299	GO:0010718	positive regulation of epithelial to mesenchymal transition	11	61
2300	GO:0010832	negative regulation of myotube differentiation	11	57
2301	GO:0010870	positive regulation of receptor biosynthetic process	11	60
2302	GO:0015884	folic acid transport	11	63
2303	GO:0016075	rRNA catabolic process	11	44
2304	GO:0016553	base conversion or substitution editing	11	27
2305	GO:0016570	histone modification	11	60
2306	GO:0017144	drug metabolic process	11	28
2307	GO:0019076	release of virus from host	11	43
2308	GO:0019220	regulation of phosphate metabolic process	11	64
2309	GO:0019464	glycine decarboxylation via glycine cleavage system	11	27
2310	GO:0019762	glucosinolate catabolic process	11	62
2311	GO:0019932	second-messenger-mediated signaling	11	40
2312	GO:0022604	regulation of cell morphogenesis	11	76
2313	GO:0030033	microvillus assembly	11	76
2314	GO:0030252	growth hormone secretion	11	21
2315	GO:0030644	cellular chloride ion homeostasis	11	67
2316	GO:0030705	cytoskeleton-dependent intracellular transport	11	42
2317	GO:0030801	positive regulation of cyclic nucleotide metabolic process	11	18
2318	GO:0031056	regulation of histone modification	11	64
2319	GO:0031060	regulation of histone methylation	11	58
2320	GO:0031204	posttranslational protein targeting to membrane, translocation	11	28
2321	GO:0031497	chromatin assembly	11	47
2322	GO:0031667	response to nutrient levels	11	41
2323	GO:0031938	regulation of chromatin silencing at telomere	11	29
2324	GO:0032790	ribosome disassembly	11	38
2325	GO:0032874	positive regulation of stress-activated MAPK cascade	11	78
2326	GO:0033129	positive regulation of histone phosphorylation	11	63
2327	GO:0033137	negative regulation of peptidyl-serine phosphorylation	11	51
2328	GO:0033692	cellular polysaccharide biosynthetic process	11	25
2329	GO:0034498	early endosome to Golgi transport	11	39
2330	GO:0035072	ecdysone-mediated induction of salivary gland cell autophagic cell death	11	56

2331	GO:0035167	larval lymph gland hemopoiesis	11	38
2332	GO:0035194	posttranscriptional gene silencing by RNA	11	72
2333	GO:0035428	hexose transmembrane transport	11	66
2334	GO:0035542	regulation of SNARE complex assembly	11	57
2335	GO:0035729	cellular response to hepatocyte growth factor stimulus	11	77
2336	GO:0035984	cellular response to trichostatin A	11	57
2337	GO:0036099	female germ-line stem cell maintenance	11	40
2338	GO:0043200	response to amino acid stimulus	11	65
2339	GO:0043447	alkane biosynthetic process	11	28
2340	GO:0043489	RNA stabilization	11	27
2341	GO:0044011	single-species biofilm formation on inanimate substrate	11	94
2342	GO:0044205	'de novo' UMP biosynthetic process	11	55
2343	GO:0044209	AMP salvage	11	57
2344	GO:0045141	meiotic telomere clustering	11	85
2345	GO:0045475	locomotor rhythm	11	89
2346	GO:0045596	negative regulation of cell differentiation	11	48
2347	GO:0045697	regulation of synergid differentiation	11	38
2348	GO:0045742	positive regulation of epidermal growth factor receptor signaling pathway	11	31
2349	GO:0045766	positive regulation of angiogenesis	11	50
2350	GO:0046580	negative regulation of Ras protein signal transduction	11	72
2351	GO:0046676	negative regulation of insulin secretion	11	79
2352	GO:0046939	nucleotide phosphorylation	11	45
2353	GO:0048194	Golgi vesicle budding	11	51
2354	GO:0048280	vesicle fusion with Golgi apparatus	11	30
2355	GO:0048489	synaptic vesicle transport	11	32
2356	GO:0048588	developmental cell growth	11	69
2357	GO:0048609	multicellular organismal reproductive process	11	37
2358	GO:0048714	positive regulation of oligodendrocyte differentiation	11	63
2359	GO:0050900	leukocyte migration	11	66
2360	GO:0050905	neuromuscular process	11	49
2361	GO:0051090	regulation of sequence-specific DNA binding transcription factor activity	11	54
2362	GO:0051098	regulation of binding	11	99
2363	GO:0051443	positive regulation of ubiquitin-protein ligase activity	11	35
2364	GO:0051491	positive regulation of filopodium assembly	11	42
2365	GO:0051656	establishment of organelle localization	11	72
2366	GO:0055013	cardiac muscle cell development	11	81
2367	GO:0060749	mammary gland alveolus development	11	50
2368	GO:0061061	muscle structure development	11	46
2369	GO:0070072	vacuolar proton-transporting V-type ATPase complex assembly	11	65
2370	GO:0070536	protein K63-linked deubiquitination	11	52
2371	GO:0071244	cellular response to carbon dioxide	11	26
2372	GO:0071329	cellular response to sucrose stimulus	11	84
2373	GO:0071354	cellular response to interleukin-6	11	41
2374	GO:0071483	cellular response to blue light	11	54
2375	GO:0071668	plant-type cell wall assembly	11	16
2376	GO:0072606	interleukin-8 secretion	11	21
2377	GO:0080029	cellular response to boron-containing substance levels	11	37
2378	GO:0080121	AMP transport	11	40
2379	GO:0080163	regulation of protein serine/threonine phosphatase activity	11	34
2380	GO:0086015	regulation of SA node cardiac muscle cell action potential	11	26
2381	GO:0090110	cargo loading into COPII-coated vesicle	11	21
2382	GO:1901012	(S)-reticuline biosynthetic process	11	20
2383	GO:2000002	negative regulation of DNA damage checkpoint	11	64
2384	GO:2000010	positive regulation of protein localization at cell surface	11	75
2385	GO:2000077	negative regulation of type B pancreatic cell development	11	52

2386	GO:2000466	negative regulation of glycogen (starch) synthase activity	11	52
2387	GO:2000676	positive regulation of type B pancreatic cell apoptotic process	11	80
2388	GO:2000905	negative regulation of starch metabolic process	11	28
2389	GO:2001135	regulation of endocytic recycling	11	30
2390	GO:2001240	negative regulation of extrinsic apoptotic signaling pathway in absence of ligand	11	44
2391	GO:2001241	positive regulation of extrinsic apoptotic signaling pathway in absence of ligand	11	30
2392	GO:0000022	mitotic spindle elongation	10	20
2393	GO:0000117	regulation of transcription involved in G2/M-phase of mitotic cell cycle	10	44
2394	GO:0000291	nuclear-transcribed mRNA catabolic process, exonucleolytic	10	61
2395	GO:0000747	conjugation with cellular fusion	10	72
2396	GO:0001403	invasive growth in response to glucose limitation	10	59
2397	GO:0001975	response to amphetamine	10	48
2398	GO:0002121	inter-male aggressive behavior	10	65
2399	GO:0002833	positive regulation of response to biotic stimulus	10	45
2400	GO:0003007	heart morphogenesis	10	85
2401	GO:0003231	cardiac ventricle development	10	36
2402	GO:0005981	regulation of glycogen catabolic process	10	30
2403	GO:0006069	ethanol oxidation	10	34
2404	GO:0006121	mitochondrial electron transport, succinate to ubiquinone	10	41
2405	GO:0006270	DNA-dependent DNA replication initiation	10	33
2406	GO:0006693	prostaglandin metabolic process	10	29
2407	GO:0006751	glutathione catabolic process	10	35
2408	GO:0006824	cobalt ion transport	10	64
2409	GO:0007004	telomere maintenance via telomerase	10	66
2410	GO:0007064	mitotic sister chromatid cohesion	10	30
2411	GO:0007093	mitotic cell cycle checkpoint	10	58
2412	GO:0007130	synaptonemal complex assembly	10	67
2413	GO:0007164	establishment of tissue polarity	10	23
2414	GO:0007307	eggshell chorion gene amplification	10	101
2415	GO:0007367	segment polarity determination	10	38
2416	GO:0008045	motor axon guidance	10	22
2417	GO:0008206	bile acid metabolic process	10	110
2418	GO:0008645	hexose transport	10	31
2419	GO:0009088	threonine biosynthetic process	10	34
2420	GO:0009228	thiamine biosynthetic process	10	48
2421	GO:0009249	protein lipoylation	10	47
2422	GO:0009298	GDP-mannose biosynthetic process	10	45
2423	GO:0009607	response to biotic stimulus	10	26
2424	GO:0009649	entrainment of circadian clock	10	36
2425	GO:0009700	indole phytoalexin biosynthetic process	10	23
2426	GO:0009773	photosynthetic electron transport in photosystem I	10	24
2427	GO:0009807	lignan biosynthetic process	10	14
2428	GO:0009886	post-embryonic morphogenesis	10	27
2429	GO:0009963	positive regulation of flavonoid biosynthetic process	10	27
2430	GO:0009971	anastral spindle assembly involved in male meiosis	10	64
2431	GO:0010048	vernalization response	10	62
2432	GO:0010059	positive regulation of atrichoblast fate specification	10	93
2433	GO:0010083	regulation of vegetative meristem growth	10	40
2434	GO:0010094	specification of carpel identity	10	64
2435	GO:0010184	cytokinin transport	10	46
2436	GO:0010196	nonphotochemical quenching	10	35
2437	GO:0010265	SCF complex assembly	10	48
2438	GO:0010366	negative regulation of ethylene biosynthetic process	10	86
2439	GO:0010498	proteasomal protein catabolic process	10	31

2440	GO:0010587	miRNA catabolic process	10	58
2441	GO:0010613	positive regulation of cardiac muscle hypertrophy	10	60
2442	GO:0010614	negative regulation of cardiac muscle hypertrophy	10	49
2443	GO:0010821	regulation of mitochondrion organization	10	68
2444	GO:0010923	negative regulation of phosphatase activity	10	65
2445	GO:0015758	glucose transport	10	26
2446	GO:0015798	myo-inositol transport	10	51
2447	GO:0015843	methylammonium transport	10	23
2448	GO:0015879	carnitine transport	10	28
2449	GO:0015918	sterol transport	10	44
2450	GO:0016046	detection of fungus	10	22
2451	GO:0016572	histone phosphorylation	10	55
2452	GO:0017157	regulation of exocytosis	10	34
2453	GO:0018171	peptidyl-cysteine oxidation	10	63
2454	GO:0018230	peptidyl-L-cysteine S-palmitoylation	10	79
2455	GO:0019563	glycerol catabolic process	10	47
2456	GO:0019740	nitrogen utilization	10	20
2457	GO:0021772	olfactory bulb development	10	36
2458	GO:0030218	erythrocyte differentiation	10	38
2459	GO:0030473	nuclear migration along microtubule	10	62
2460	GO:0030643	cellular phosphate ion homeostasis	10	23
2461	GO:0030951	establishment or maintenance of microtubule cytoskeleton polarity	10	52
2462	GO:0031532	actin cytoskeleton reorganization	10	52
2463	GO:0031584	activation of phospholipase D activity	10	43
2464	GO:0032008	positive regulation of TOR signaling cascade	10	86
2465	GO:0032780	negative regulation of ATPase activity	10	52
2466	GO:0032967	positive regulation of collagen biosynthetic process	10	53
2467	GO:0033189	response to vitamin A	10	46
2468	GO:0033523	histone H2B ubiquitination	10	30
2469	GO:0033674	positive regulation of kinase activity	10	64
2470	GO:0034067	protein localization in Golgi apparatus	10	52
2471	GO:0034470	ncRNA processing	10	38
2472	GO:0035063	nuclear speck organization	10	68
2473	GO:0035087	siRNA loading onto RISC involved in RNA interference	10	31
2474	GO:0035093	spermatogenesis, exchange of chromosomal proteins	10	143
2475	GO:0035094	response to nicotine	10	52
2476	GO:0035116	embryonic hindlimb morphogenesis	10	66
2477	GO:0035220	wing disc development	10	78
2478	GO:0035317	imaginal disc-derived wing hair organization	10	77
2479	GO:0035372	protein localization to microtubule	10	45
2480	GO:0035773	insulin secretion involved in cellular response to glucose stimulus	10	28
2481	GO:0035884	arabinan biosynthetic process	10	38
2482	GO:0035987	endodermal cell differentiation	10	14
2483	GO:0036016	cellular response to interleukin-3	10	49
2484	GO:0042149	cellular response to glucose starvation	10	134
2485	GO:0042307	positive regulation of protein import into nucleus	10	28
2486	GO:0042793	transcription from plastid promoter	10	44
2487	GO:0042921	glucocorticoid receptor signaling pathway	10	24
2488	GO:0043090	amino acid import	10	23
2489	GO:0043279	response to alkaloid	10	39
2490	GO:0043518	negative regulation of DNA damage response, signal transduction by p53 class mediator	10	60
2491	GO:0043951	negative regulation of cAMP-mediated signaling	10	41
2492	GO:0044262	cellular carbohydrate metabolic process	10	53
2493	GO:0045054	constitutive secretory pathway	10	28
2494	GO:0045324	late endosome to vacuole transport	10	30
2495	GO:0045347	negative regulation of MHC class II biosynthetic process	10	49
2496	GO:0045493	xylan catabolic process	10	24

2497	GO:0045494	photoreceptor cell maintenance	10	109
2498	GO:0045934	negative regulation of nucleobase-containing compound metabolic process	10	31
2499	GO:0046355	mannan catabolic process	10	20
2500	GO:0046622	positive regulation of organ growth	10	21
2501	GO:0046784	intronless viral mRNA export from host nucleus	10	53
2502	GO:0046839	phospholipid dephosphorylation	10	35
2503	GO:0047497	mitochondrion transport along microtubule	10	37
2504	GO:0048832	specification of organ number	10	40
2505	GO:0048834	specification of petal number	10	27
2506	GO:0050667	homocysteine metabolic process	10	56
2507	GO:0050691	regulation of defense response to virus by host	10	30
2508	GO:0050775	positive regulation of dendrite morphogenesis	10	22
2509	GO:0050847	progesterone receptor signaling pathway	10	54
2510	GO:0051094	positive regulation of developmental process	10	54
2511	GO:0051403	stress-activated MAPK cascade	10	38
2512	GO:0051489	regulation of filopodium assembly	10	55
2513	GO:0051683	establishment of Golgi localization	10	87
2514	GO:0052096	formation by symbiont of syncytium involving giant cell for nutrient acquisition from host	10	56
2515	GO:0055074	calcium ion homeostasis	10	124
2516	GO:0060013	righting reflex	10	43
2517	GO:0060044	negative regulation of cardiac muscle cell proliferation	10	60
2518	GO:0060069	Wnt receptor signaling pathway, regulating spindle positioning	10	64
2519	GO:0060212	negative regulation of nuclear-transcribed mRNA poly(A) tail shortening	10	39
2520	GO:0060348	bone development	10	49
2521	GO:0060420	regulation of heart growth	10	49
2522	GO:0060452	positive regulation of cardiac muscle contraction	10	14
2523	GO:0061087	positive regulation of histone H3-K27 methylation	10	74
2524	GO:0070125	mitochondrial translational elongation	10	85
2525	GO:0070371	ERK1 and ERK2 cascade	10	15
2526	GO:0071038	nuclear polyadenylation-dependent tRNA catabolic process	10	76
2527	GO:0071353	cellular response to interleukin-4	10	60
2528	GO:0071786	endoplasmic reticulum tubular network organization	10	30
2529	GO:0072331	signal transduction by p53 class mediator	10	92
2530	GO:0072383	plus-end-directed vesicle transport along microtubule	10	59
2531	GO:0072488	ammonium transmembrane transport	10	20
2532	GO:0072711	cellular response to hydroxyurea	10	87
2533	GO:0080094	response to trehalose-6-phosphate stimulus	10	49
2534	GO:0080127	fruit septum development	10	17
2535	GO:0080136	priming of cellular response to stress	10	47
2536	GO:0086002	regulation of cardiac muscle cell action potential involved in contraction	10	25
2537	GO:0086066	atrial cardiomyocyte to AV node cell communication	10	25
2538	GO:0086091	regulation of heart rate by cardiac conduction	10	43
2539	GO:0090005	negative regulation of establishment of protein localization in plasma membrane	10	30
2540	GO:0090089	regulation of dipeptide transport	10	36
2541	GO:0090148	membrane fission	10	77
2542	GO:0090219	negative regulation of lipid kinase activity	10	30
2543	GO:0090315	negative regulation of protein targeting to membrane	10	77
2544	GO:0097164	ammonium ion metabolic process	10	32
2545	GO:0097192	extrinsic apoptotic signaling pathway in absence of ligand	10	49
2546	GO:1900364	negative regulation of mRNA polyadenylation	10	35
2547	GO:1901030	positive regulation of mitochondrial outer membrane permeabilization	10	49

2548	GO:2000286	receptor internalization involved in canonical Wnt receptor signaling pathway	10	56
2549	GO:2000369	regulation of clathrin-mediated endocytosis	10	92
2550	GO:2001234	negative regulation of apoptotic signaling pathway	10	63
2551	GO:0000042	protein targeting to Golgi	9	17
2552	GO:0000105	histidine biosynthetic process	9	41
2553	GO:0000294	nuclear-transcribed mRNA catabolic process, endonucleolytic cleavage-dependent decay	9	98
2554	GO:0000320	re-entry into mitotic cell cycle	9	43
2555	GO:0000461	endonucleolytic cleavage to generate mature 3'-end of SSU-rRNA from (SSU-rRNA, 5.8S rRNA, LSU-rRNA)	9	15
2556	GO:0000719	photoreactive repair	9	72
2557	GO:0000956	nuclear-transcribed mRNA catabolic process	9	49
2558	GO:0001678	cellular glucose homeostasis	9	44
2559	GO:0001732	formation of translation initiation complex	9	18
2560	GO:0001745	compound eye morphogenesis	9	36
2561	GO:0001887	selenium compound metabolic process	9	38
2562	GO:0001916	positive regulation of T cell mediated cytotoxicity	9	11
2563	GO:0002088	lens development in camera-type eye	9	44
2564	GO:0002224	toll-like receptor signaling pathway	9	41
2565	GO:0002429	immune response-activating cell surface receptor signaling pathway	9	17
2566	GO:0002831	regulation of response to biotic stimulus	9	26
2567	GO:0006024	glycosaminoglycan biosynthetic process	9	34
2568	GO:0006048	UDP-N-acetylglucosamine biosynthetic process	9	50
2569	GO:0006452	translational frameshifting	9	48
2570	GO:0006475	internal protein amino acid acetylation	9	27
2571	GO:0006497	protein lipidation	9	72
2572	GO:0006517	protein deglycosylation	9	29
2573	GO:0006523	alanine biosynthetic process	9	25
2574	GO:0006545	glycine biosynthetic process	9	49
2575	GO:0006546	glycine catabolic process	9	28
2576	GO:0006622	protein targeting to lysosome	9	30
2577	GO:0006627	protein processing involved in protein targeting to mitochondrion	9	52
2578	GO:0006739	NADP metabolic process	9	53
2579	GO:0006740	NADPH regeneration	9	38
2580	GO:0007077	mitotic nuclear envelope disassembly	9	49
2581	GO:0007096	regulation of exit from mitosis	9	21
2582	GO:0007100	mitotic centrosome separation	9	44
2583	GO:0007291	sperm individualization	9	38
2584	GO:0007312	oocyte nucleus migration involved in oocyte dorsal/ventral axis specification	9	21
2585	GO:0007416	synapse assembly	9	50
2586	GO:0007423	sensory organ development	9	50
2587	GO:0007480	imaginal disc-derived leg morphogenesis	9	38
2588	GO:0008586	imaginal disc-derived wing vein morphogenesis	9	84
2589	GO:0008593	regulation of Notch signaling pathway	9	37
2590	GO:0008615	pyridoxine biosynthetic process	9	51
2591	GO:0009052	pentose-phosphate shunt, non-oxidative branch	9	19
2592	GO:0009057	macromolecule catabolic process	9	14
2593	GO:0009229	thiamine diphosphate biosynthetic process	9	32
2594	GO:0009698	phenylpropanoid metabolic process	9	24
2595	GO:0009727	detection of ethylene stimulus	9	59
2596	GO:0009746	response to hexose stimulus	9	26
2597	GO:0009823	cytokinin catabolic process	9	29
2598	GO:0009830	cell wall modification involved in abscission	9	41
2599	GO:0009871	jasmonic acid and ethylene-dependent systemic resistance, ethylene mediated signaling pathway	9	53
2600	GO:0009991	response to extracellular stimulus	9	39

2601	GO:0010028	xanthophyll cycle	9	44
2602	GO:0010042	response to manganese ion	9	45
2603	GO:0010045	response to nickel cation	9	11
2604	GO:0010054	trichoblast differentiation	9	11
2605	GO:0010190	cytochrome b6f complex assembly	9	52
2606	GO:0010306	rhamnogalacturonan II biosynthetic process	9	48
2607	GO:0010469	regulation of receptor activity	9	36
2608	GO:0010581	regulation of starch biosynthetic process	9	26
2609	GO:0010882	regulation of cardiac muscle contraction by calcium ion signaling	9	57
2610	GO:0010949	negative regulation of intestinal phytosterol absorption	9	18
2611	GO:0014003	oligodendrocyte development	9	53
2612	GO:0014043	negative regulation of neuron maturation	9	43
2613	GO:0015672	monovalent inorganic cation transport	9	45
2614	GO:0015713	phosphoglycerate transport	9	55
2615	GO:0015721	bile acid and bile salt transport	9	80
2616	GO:0015768	maltose transport	9	50
2617	GO:0015800	acidic amino acid transport	9	28
2618	GO:0015804	neutral amino acid transport	9	19
2619	GO:0015850	organic alcohol transport	9	21
2620	GO:0015882	L-ascorbic acid transport	9	42
2621	GO:0019221	cytokine-mediated signaling pathway	9	56
2622	GO:0019481	L-alanine catabolic process, by transamination	9	27
2623	GO:0021915	neural tube development	9	37
2624	GO:0022412	cellular process involved in reproduction in multicellular organism	9	59
2625	GO:0022417	protein maturation by protein folding	9	27
2626	GO:0030031	cell projection assembly	9	60
2627	GO:0030242	peroxisome degradation	9	93
2628	GO:0030261	chromosome condensation	9	23
2629	GO:0030505	inorganic diphosphate transport	9	32
2630	GO:0030511	positive regulation of transforming growth factor beta receptor signaling pathway	9	64
2631	GO:0030517	negative regulation of axon extension	9	45
2632	GO:0031117	positive regulation of microtubule depolymerization	9	90
2633	GO:0031400	negative regulation of protein modification process	9	71
2634	GO:0031401	positive regulation of protein modification process	9	63
2635	GO:0031539	positive regulation of anthocyanin metabolic process	9	27
2636	GO:0031623	receptor internalization	9	36
2637	GO:0031930	mitochondria-nucleus signaling pathway	9	37
2638	GO:0032201	telomere maintenance via semi-conservative replication	9	98
2639	GO:0032875	regulation of DNA endoreduplication	9	78
2640	GO:0032876	negative regulation of DNA endoreduplication	9	69
2641	GO:0032877	positive regulation of DNA endoreduplication	9	79
2642	GO:0032886	regulation of microtubule-based process	9	37
2643	GO:0033119	negative regulation of RNA splicing	9	37
2644	GO:0033473	indoleacetic acid conjugate metabolic process	9	44
2645	GO:0033506	glucosinolate biosynthetic process from homomethionine	9	29
2646	GO:0033555	multicellular organismal response to stress	9	61
2647	GO:0033601	positive regulation of mammary gland epithelial cell proliferation	9	88
2648	GO:0034198	cellular response to amino acid starvation	9	42
2649	GO:0034201	response to oleic acid	9	60
2650	GO:0034497	protein localization to pre-autophagosomal structure	9	89
2651	GO:0034501	protein localization to kinetochore	9	51
2652	GO:0034638	phosphatidylcholine catabolic process	9	68
2653	GO:0034660	ncRNA metabolic process	9	56
2654	GO:0034757	negative regulation of iron ion transport	9	69
2655	GO:0035071	salivary gland cell autophagic cell death	9	32
2656	GO:0035279	mRNA cleavage involved in gene silencing by miRNA	9	32

2657	GO:0035280	miRNA loading onto RISC involved in gene silencing by miRNA	9	65
2658	GO:0035434	copper ion transmembrane transport	9	41
2659	GO:0035436	triose phosphate transmembrane transport	9	55
2660	GO:0035725	sodium ion transmembrane transport	9	31
2661	GO:0036098	male germ-line stem cell maintenance	9	69
2662	GO:0042060	wound healing	9	76
2663	GO:0042547	cell wall modification involved in multidimensional cell growth	9	28
2664	GO:0042776	mitochondrial ATP synthesis coupled proton transport	9	22
2665	GO:0042790	transcription of nuclear large rRNA transcript from RNA polymerase I promoter	9	71
2666	GO:0043157	response to cation stress	9	48
2667	GO:0043652	engulfment of apoptotic cell	9	49
2668	GO:0043693	monoterpene biosynthetic process	9	25
2669	GO:0044337	canonical Wnt receptor signaling pathway involved in positive regulation of apoptotic process	9	43
2670	GO:0044351	macropinocytosis	9	32
2671	GO:0045041	protein import into mitochondrial intermembrane space	9	54
2672	GO:0045071	negative regulation of viral genome replication	9	31
2673	GO:0045184	establishment of protein localization	9	42
2674	GO:0045197	establishment or maintenance of epithelial cell apical/basal polarity	9	39
2675	GO:0045333	cellular respiration	9	41
2676	GO:0045737	positive regulation of cyclin-dependent protein kinase activity	9	56
2677	GO:0045746	negative regulation of Notch signaling pathway	9	50
2678	GO:0045777	positive regulation of blood pressure	9	33
2679	GO:0045796	negative regulation of intestinal cholesterol absorption	9	18
2680	GO:0045836	positive regulation of meiosis	9	75
2681	GO:0045962	positive regulation of development, heterochronic	9	17
2682	GO:0046330	positive regulation of JNK cascade	9	53
2683	GO:0046425	regulation of JAK-STAT cascade	9	57
2684	GO:0046443	FAD metabolic process	9	54
2685	GO:0046786	viral replication complex formation and maintenance	9	72
2686	GO:0046855	inositol phosphate dephosphorylation	9	60
2687	GO:0048363	mucilage pectin metabolic process	9	13
2688	GO:0048488	synaptic vesicle endocytosis	9	26
2689	GO:0048504	regulation of timing of organ formation	9	20
2690	GO:0048562	embryonic organ morphogenesis	9	56
2691	GO:0048742	regulation of skeletal muscle fiber development	9	29
2692	GO:0048800	antennal morphogenesis	9	76
2693	GO:0048814	regulation of dendrite morphogenesis	9	74
2694	GO:0048863	stem cell differentiation	9	31
2695	GO:0051036	regulation of endosome size	9	40
2696	GO:0051046	regulation of secretion	9	84
2697	GO:0051056	regulation of small GTPase mediated signal transduction	9	52
2698	GO:0051123	RNA polymerase II transcriptional preinitiation complex assembly	9	73
2699	GO:0051129	negative regulation of cellular component organization	9	73
2700	GO:0051211	anisotropic cell growth	9	26
2701	GO:0051235	maintenance of location	9	24
2702	GO:0051503	adenine nucleotide transport	9	27
2703	GO:0051601	exocyst localization	9	41
2704	GO:0051965	positive regulation of synapse assembly	9	34
2705	GO:0055002	striated muscle cell development	9	36
2706	GO:0055007	cardiac muscle cell differentiation	9	23
2707	GO:0055059	asymmetric neuroblast division	9	33
2708	GO:0055078	sodium ion homeostasis	9	22
2709	GO:0060135	maternal process involved in female pregnancy	9	40
2710	GO:0060218	hemopoietic stem cell differentiation	9	85

2711	GO:0060237	regulation of fungal-type cell wall organization	9	66
2712	GO:0060292	long term synaptic depression	9	47
2713	GO:0060315	negative regulation of ryanodine-sensitive calcium-release channel activity	9	16
2714	GO:0060316	positive regulation of ryanodine-sensitive calcium-release channel activity	9	16
2715	GO:0060471	cortical granule exocytosis	9	29
2716	GO:0060628	regulation of ER to Golgi vesicle-mediated transport	9	52
2717	GO:0060816	random inactivation of X chromosome	9	50
2718	GO:0061000	negative regulation of dendritic spine development	9	46
2719	GO:0061029	eyelid development in camera-type eye	9	48
2720	GO:0070206	protein trimerization	9	50
2721	GO:0070208	protein heterotrimerization	9	50
2722	GO:0070478	nuclear-transcribed mRNA catabolic process, 3'-5' exonucleolytic nonsense-mediated decay	9	73
2723	GO:0070829	heterochromatin maintenance	9	34
2724	GO:0071169	establishment of protein localization to chromatin	9	49
2725	GO:0071383	cellular response to steroid hormone stimulus	9	97
2726	GO:0071417	cellular response to organic nitrogen	9	94
2727	GO:0071474	cellular hyperosmotic response	9	86
2728	GO:0071481	cellular response to X-ray	9	38
2729	GO:0071705	nitrogen compound transport	9	21
2730	GO:0072318	clathrin coat disassembly	9	11
2731	GO:0072332	signal transduction by p53 class mediator resulting in induction of apoptosis	9	43
2732	GO:0072423	response to DNA damage checkpoint signal	9	37
2733	GO:0086036	regulation of cardiac muscle cell membrane potential	9	57
2734	GO:0090051	negative regulation of cell migration involved in sprouting angiogenesis	9	22
2735	GO:0090279	regulation of calcium ion import	9	86
2736	GO:0090303	positive regulation of wound healing	9	38
2737	GO:0097191	extrinsic apoptotic signaling pathway	9	71
2738	GO:0097193	intrinsic apoptotic signaling pathway	9	30
2739	GO:0097214	positive regulation of lysosomal membrane permeability	9	11
2740	GO:0097308	cellular response to farnesol	9	83
2741	GO:1900246	positive regulation of RIG-I signaling pathway	9	60
2742	GO:2000300	regulation of synaptic vesicle exocytosis	9	40
2743	GO:2000738	positive regulation of stem cell differentiation	9	43
2744	GO:2000786	positive regulation of autophagic vacuole assembly	9	69
2745	GO:2001020	regulation of response to DNA damage stimulus	9	51
2746	GO:2001168	positive regulation of histone H2B ubiquitination	9	27
2747	GO:2001236	regulation of extrinsic apoptotic signaling pathway	9	64
2748	GO:0000002	mitochondrial genome maintenance	8	38
2749	GO:0000075	cell cycle checkpoint	8	29
2750	GO:0000237	leptotene	8	30
2751	GO:0000730	DNA recombinase assembly	8	51
2752	GO:0000749	response to pheromone involved in conjugation with cellular fusion	8	22
2753	GO:0001818	negative regulation of cytokine production	8	30
2754	GO:0001819	positive regulation of cytokine production	8	18
2755	GO:0001974	blood vessel remodeling	8	35
2756	GO:0002031	G-protein coupled receptor internalization	8	34
2757	GO:0002092	positive regulation of receptor internalization	8	79
2758	GO:0002502	peptide antigen assembly with MHC class I protein complex	8	17
2759	GO:0006007	glucose catabolic process	8	23
2760	GO:0006164	purine nucleotide biosynthetic process	8	18
2761	GO:0006169	adenosine salvage	8	35
2762	GO:0006285	base-excision repair, AP site formation	8	27
2763	GO:0006402	mRNA catabolic process	8	18
2764	GO:0006415	translational termination	8	48

2765	GO:0006488	dolichol-linked oligosaccharide biosynthetic process	8	51
2766	GO:0006516	glycoprotein catabolic process	8	53
2767	GO:0006588	activation of tryptophan 5-monooxygenase activity	8	23
2768	GO:0006597	spermine biosynthetic process	8	34
2769	GO:0006921	cellular component disassembly involved in apoptotic process	8	70
2770	GO:0007057	spindle assembly involved in female meiosis I	8	43
2771	GO:0007127	meiosis I	8	31
2772	GO:0007257	activation of JUN kinase activity	8	65
2773	GO:0007279	pole cell formation	8	27
2774	GO:0007288	sperm axoneme assembly	8	30
2775	GO:0007403	glial cell fate determination	8	77
2776	GO:0007492	endoderm development	8	45
2777	GO:0007552	metamorphosis	8	101
2778	GO:0007611	learning or memory	8	48
2779	GO:0007638	mechanosensory behavior	8	30
2780	GO:0008015	blood circulation	8	42
2781	GO:0008542	visual learning	8	28
2782	GO:0009152	purine ribonucleotide biosynthetic process	8	28
2783	GO:0009270	response to humidity	8	35
2784	GO:0009432	SOS response	8	76
2785	GO:0009435	NAD biosynthetic process	8	49
2786	GO:0009446	putrescine biosynthetic process	8	69
2787	GO:0009558	embryo sac cellularization	8	34
2788	GO:0009593	detection of chemical stimulus	8	17
2789	GO:0009701	isoflavonoid phytoalexin biosynthetic process	8	14
2790	GO:0009722	detection of cytokinin stimulus	8	25
2791	GO:0009856	pollination	8	38
2792	GO:0010172	embryonic body morphogenesis	8	27
2793	GO:0010235	guard mother cell cytokinesis	8	55
2794	GO:0010236	plastoquinone biosynthetic process	8	58
2795	GO:0010248	establishment or maintenance of transmembrane electrochemical gradient	8	22
2796	GO:0010255	glucose mediated signaling pathway	8	38
2797	GO:0010274	hydrotropism	8	15
2798	GO:0010392	galactoglucomannan metabolic process	8	13
2799	GO:0010413	glucuronoxylan metabolic process	8	34
2800	GO:0010455	positive regulation of cell fate commitment	8	14
2801	GO:0010466	negative regulation of peptidase activity	8	12
2802	GO:0010476	gibberellin mediated signaling pathway	8	26
2803	GO:0010731	protein glutathionylation	8	23
2804	GO:0010817	regulation of hormone levels	8	22
2805	GO:0010845	positive regulation of reciprocal meiotic recombination	8	30
2806	GO:0014706	striated muscle tissue development	8	45
2807	GO:0015014	heparan sulfate proteoglycan biosynthetic process, polysaccharide chain biosynthetic process	8	19
2808	GO:0015698	inorganic anion transport	8	26
2809	GO:0015722	canalicular bile acid transport	8	22
2810	GO:0016319	mushroom body development	8	20
2811	GO:0016540	protein autoprocessing	8	37
2812	GO:0016571	histone methylation	8	25
2813	GO:0017183	peptidyl-diphthamide biosynthetic process from peptidyl-histidine	8	51
2814	GO:0019058	viral infectious cycle	8	45
2815	GO:0019222	regulation of metabolic process	8	25
2816	GO:0019676	ammonia assimilation cycle	8	38
2817	GO:0021680	cerebellar Purkinje cell layer development	8	56
2818	GO:0021702	cerebellar Purkinje cell differentiation	8	32
2819	GO:0021895	cerebral cortex neuron differentiation	8	140
2820	GO:0023052	signaling	8	40

2821	GO:0030003	cellular cation homeostasis	8	31
2822	GO:0030421	defecation	8	24
2823	GO:0030540	female genitalia development	8	30
2824	GO:0030837	negative regulation of actin filament polymerization	8	37
2825	GO:0030865	cortical cytoskeleton organization	8	19
2826	GO:0030902	hindbrain development	8	48
2827	GO:0031022	nuclear migration along microfilament	8	35
2828	GO:0031115	negative regulation of microtubule polymerization	8	40
2829	GO:0031295	T cell costimulation	8	63
2830	GO:0031327	negative regulation of cellular biosynthetic process	8	26
2831	GO:0031365	N-terminal protein amino acid modification	8	63
2832	GO:0031440	regulation of mRNA 3'-end processing	8	57
2833	GO:0031581	hemidesmosome assembly	8	66
2834	GO:0031929	TOR signaling cascade	8	49
2835	GO:0031952	regulation of protein autophosphorylation	8	47
2836	GO:0032079	positive regulation of endodeoxyribonuclease activity	8	47
2837	GO:0032211	negative regulation of telomere maintenance via telomerase	8	67
2838	GO:0032260	response to jasmonic acid stimulus involved in jasmonic acid and ethylene-dependent systemic resistance	8	13
2839	GO:0032460	negative regulation of protein oligomerization	8	13
2840	GO:0032544	plastid translation	8	27
2841	GO:0032732	positive regulation of interleukin-1 production	8	45
2842	GO:0032801	receptor catabolic process	8	32
2843	GO:0032889	regulation of vacuole fusion, non-autophagic	8	25
2844	GO:0032957	inositol trisphosphate metabolic process	8	38
2845	GO:0033128	negative regulation of histone phosphorylation	8	30
2846	GO:0033144	negative regulation of intracellular steroid hormone receptor signaling pathway	8	17
2847	GO:0033320	UDP-D-xylose biosynthetic process	8	56
2848	GO:0033383	geranyl diphosphate metabolic process	8	18
2849	GO:0033522	histone H2A ubiquitination	8	30
2850	GO:0033587	shikimate biosynthetic process	8	15
2851	GO:0033600	negative regulation of mammary gland epithelial cell proliferation	8	18
2852	GO:0034612	response to tumor necrosis factor	8	87
2853	GO:0035020	regulation of Rac protein signal transduction	8	57
2854	GO:0035042	fertilization, exchange of chromosomal proteins	8	124
2855	GO:0035188	hatching	8	66
2856	GO:0035445	borate transmembrane transport	8	32
2857	GO:0035646	endosome to melanosome transport	8	49
2858	GO:0035774	positive regulation of insulin secretion involved in cellular response to glucose stimulus	8	47
2859	GO:0035970	peptidyl-threonine dephosphorylation	8	40
2860	GO:0036065	fucosylation	8	31
2861	GO:0036091	positive regulation of transcription from RNA polymerase II promoter in response to oxidative stress	8	143
2862	GO:0038083	peptidyl-tyrosine autophosphorylation	8	72
2863	GO:0040034	regulation of development, heterochronic	8	56
2864	GO:0042144	vacuole fusion, non-autophagic	8	52
2865	GO:0042218	1-aminocyclopropane-1-carboxylate biosynthetic process	8	21
2866	GO:0042407	cristae formation	8	56
2867	GO:0042450	arginine biosynthetic process via ornithine	8	23
2868	GO:0042475	odontogenesis of dentin-containing tooth	8	45
2869	GO:0042517	positive regulation of tyrosine phosphorylation of Stat3 protein	8	45
2870	GO:0042518	negative regulation of tyrosine phosphorylation of Stat3 protein	8	27
2871	GO:0042692	muscle cell differentiation	8	49

2872	GO:0042998	positive regulation of Golgi to plasma membrane protein transport	8	22
2873	GO:0043122	regulation of I-kappaB kinase/NF-kappaB cascade	8	54
2874	GO:0043124	negative regulation of I-kappaB kinase/NF-kappaB cascade	8	45
2875	GO:0043406	positive regulation of MAP kinase activity	8	45
2876	GO:0043496	regulation of protein homodimerization activity	8	48
2877	GO:0043686	co-translational protein modification	8	55
2878	GO:0043970	histone H3-K9 acetylation	8	59
2879	GO:0045019	negative regulation of nitric oxide biosynthetic process	8	37
2880	GO:0045039	protein import into mitochondrial inner membrane	8	52
2881	GO:0045046	protein import into peroxisome membrane	8	40
2882	GO:0045807	positive regulation of endocytosis	8	61
2883	GO:0045841	negative regulation of mitotic metaphase/anaphase transition	8	9
2884	GO:0045879	negative regulation of smoothened signaling pathway	8	41
2885	GO:0046331	lateral inhibition	8	17
2886	GO:0046627	negative regulation of insulin receptor signaling pathway	8	35
2887	GO:0046716	muscle cell homeostasis	8	17
2888	GO:0048142	germarium-derived cystoblast division	8	21
2889	GO:0048209	regulation of vesicle targeting, to, from or within Golgi	8	50
2890	GO:0048312	intracellular distribution of mitochondria	8	55
2891	GO:0048446	petal morphogenesis	8	29
2892	GO:0048675	axon extension	8	60
2893	GO:0048920	posterior lateral line neuromast primordium migration	8	34
2894	GO:0050428	3'-phosphoadenosine 5'-phosphosulfate biosynthetic process	8	27
2895	GO:0050684	regulation of mRNA processing	8	71
2896	GO:0050765	negative regulation of phagocytosis	8	43
2897	GO:0050767	regulation of neurogenesis	8	38
2898	GO:0050801	ion homeostasis	8	33
2899	GO:0051014	actin filament severing	8	114
2900	GO:0051070	galactomannan biosynthetic process	8	13
2901	GO:0051147	regulation of muscle cell differentiation	8	20
2902	GO:0051234	establishment of localization	8	23
2903	GO:0051245	negative regulation of cellular defense response	8	32
2904	GO:0051315	attachment of spindle microtubules to kinetochore involved in mitotic sister chromatid segregation	8	49
2905	GO:0051606	detection of stimulus	8	9
2906	GO:0051896	regulation of protein kinase B signaling cascade	8	45
2907	GO:0055073	cadmium ion homeostasis	8	8
2908	GO:0060021	palate development	8	35
2909	GO:0060119	inner ear receptor cell development	8	36
2910	GO:0060306	regulation of membrane repolarization	8	41
2911	GO:0060318	definitive erythrocyte differentiation	8	81
2912	GO:0060744	mammary gland branching involved in thelarche	8	18
2913	GO:0060762	regulation of branching involved in mammary gland duct morphogenesis	8	18
2914	GO:0060789	hair follicle placode formation	8	45
2915	GO:0060850	regulation of transcription involved in cell fate commitment	8	140
2916	GO:0061198	fungiform papilla formation	8	45
2917	GO:0070286	axonemal dynein complex assembly	8	35
2918	GO:0070574	cadmium ion transmembrane transport	8	54
2919	GO:0070723	response to cholesterol	8	60
2920	GO:0070828	heterochromatin organization	8	14
2921	GO:0070988	demethylation	8	52
2922	GO:0070995	NADPH oxidation	8	61
2923	GO:0071049	nuclear retention of pre-mRNA with aberrant 3'-ends at the site of transcription	8	59
2924	GO:0071108	protein K48-linked deubiquitination	8	55

2925	GO:0071218	cellular response to misfolded protein	8	31
2926	GO:0071377	cellular response to glucagon stimulus	8	34
2927	GO:0071454	cellular response to anoxia	8	23
2928	GO:0071539	protein localization to centrosome	8	40
2929	GO:0071733	transcriptional activation by promoter-enhancer looping	8	35
2930	GO:0071850	mitotic cell cycle arrest	8	34
2931	GO:0071899	negative regulation of estrogen receptor binding	8	35
2932	GO:0072330	monocarboxylic acid biosynthetic process	8	26
2933	GO:0072521	purine-containing compound metabolic process	8	25
2934	GO:0072660	maintenance of protein location in plasma membrane	8	30
2935	GO:0080159	zygote elongation	8	75
2936	GO:0080183	response to photooxidative stress	8	115
2937	GO:0086069	bundle of His cardiac muscle cell to Purkinje myocyte communication	8	55
2938	GO:0090168	Golgi reassembly	8	75
2939	GO:0090311	regulation of protein deacetylation	8	45
2940	GO:0097190	apoptotic signaling pathway	8	38
2941	GO:1900095	regulation of dosage compensation by inactivation of X chromosome	8	49
2942	GO:1900260	negative regulation of RNA-directed RNA polymerase activity	8	13
2943	GO:1900745	positive regulation of p38MAPK cascade	8	31
2944	GO:1901017	negative regulation of potassium ion transmembrane transporter activity	8	80
2945	GO:2000014	regulation of endosperm development	8	74
2946	GO:2000072	regulation of defense response to fungus, incompatible interaction	8	49
2947	GO:2000104	negative regulation of DNA-dependent DNA replication	8	99
2948	GO:2000113	negative regulation of cellular macromolecule biosynthetic process	8	27
2949	GO:2000331	regulation of terminal button organization	8	43
2950	GO:2000343	positive regulation of chemokine (C-X-C motif) ligand 2 production	8	45
2951	GO:2000510	positive regulation of dendritic cell chemotaxis	8	17
2952	GO:2000757	negative regulation of peptidyl-lysine acetylation	8	45
2953	GO:2001021	negative regulation of response to DNA damage stimulus	8	37
2954	GO:0000050	urea cycle	7	38
2955	GO:0000212	meiotic spindle organization	7	58
2956	GO:0000271	polysaccharide biosynthetic process	7	53
2957	GO:0000288	nuclear-transcribed mRNA catabolic process, deadenylation-dependent decay	7	41
2958	GO:0000354	cis assembly of pre-catalytic spliceosome	7	22
2959	GO:0000710	meiotic mismatch repair	7	100
2960	GO:0000733	DNA strand renaturation	7	84
2961	GO:0000917	barrier septum assembly	7	26
2962	GO:0001523	retinoid metabolic process	7	44
2963	GO:0002481	antigen processing and presentation of exogenous protein antigen via MHC class Ib, TAP-dependent	7	38
2964	GO:0002485	antigen processing and presentation of endogenous peptide antigen via MHC class I via ER pathway, TAP-dependent	7	38
2965	GO:0002489	antigen processing and presentation of endogenous peptide antigen via MHC class Ib via ER pathway, TAP-dependent	7	38
2966	GO:0002539	prostaglandin production involved in inflammatory response	7	12
2967	GO:0002591	positive regulation of antigen processing and presentation of peptide antigen via MHC class I	7	38
2968	GO:0006066	alcohol metabolic process	7	18
2969	GO:0006165	nucleoside diphosphate phosphorylation	7	61
2970	GO:0006212	uracil catabolic process	7	15
2971	GO:0006269	DNA replication, synthesis of RNA primer	7	58

2972	GO:0006356	regulation of transcription from RNA polymerase I promoter	7	43
2973	GO:0006388	tRNA splicing, via endonucleolytic cleavage and ligation	7	44
2974	GO:0006409	tRNA export from nucleus	7	33
2975	GO:0006431	methionyl-tRNA aminoacylation	7	26
2976	GO:0006474	N-terminal protein amino acid acetylation	7	23
2977	GO:0006493	protein O-linked glycosylation	7	52
2978	GO:0006509	membrane protein ectodomain proteolysis	7	31
2979	GO:0006532	aspartate biosynthetic process	7	31
2980	GO:0006598	polyamine catabolic process	7	18
2981	GO:0006768	biotin metabolic process	7	56
2982	GO:0006836	neurotransmitter transport	7	28
2983	GO:0006880	intracellular sequestering of iron ion	7	23
2984	GO:0006911	phagocytosis, engulfment	7	35
2985	GO:0007107	membrane addition at site of cytokinesis	7	45
2986	GO:0007188	adenylate cyclase-modulating G-protein coupled receptor signaling pathway	7	41
2987	GO:0007229	integrin-mediated signaling pathway	7	57
2988	GO:0007292	female gamete generation	7	34
2989	GO:0007526	larval somatic muscle development	7	51
2990	GO:0007569	cell aging	7	41
2991	GO:0007628	adult walking behavior	7	43
2992	GO:0008103	oocyte microtubule cytoskeleton polarization	7	24
2993	GO:0008156	negative regulation of DNA replication	7	17
2994	GO:0008343	adult feeding behavior	7	55
2995	GO:0008631	induction of apoptosis by oxidative stress	7	17
2996	GO:0009058	biosynthetic process	7	51
2997	GO:0009082	branched chain family amino acid biosynthetic process	7	26
2998	GO:0009102	biotin biosynthetic process	7	91
2999	GO:0009236	cobalamin biosynthetic process	7	21
3000	GO:0009623	response to parasitic fungus	7	43
3001	GO:0009660	amyloplast organization	7	90
3002	GO:0009697	salicylic acid biosynthetic process	7	33
3003	GO:0009715	chalcone biosynthetic process	7	10
3004	GO:0009747	hexokinase-dependent signaling	7	32
3005	GO:0009787	regulation of abscisic acid mediated signaling pathway	7	25
3006	GO:0009838	abscission	7	26
3007	GO:0009847	spore germination	7	39
3008	GO:0009891	positive regulation of biosynthetic process	7	33
3009	GO:0009915	phloem sucrose loading	7	21
3010	GO:0009952	anterior/posterior pattern specification	7	22
3011	GO:0010032	meiotic chromosome condensation	7	37
3012	GO:0010046	response to mycotoxin	7	37
3013	GO:0010109	regulation of photosynthesis	7	42
3014	GO:0010246	rhamnogalacturonan I biosynthetic process	7	35
3015	GO:0010324	membrane invagination	7	30
3016	GO:0010374	stomatal complex development	7	20
3017	GO:0010389	regulation of G2/M transition of mitotic cell cycle	7	31
3018	GO:0010495	long-distance posttranscriptional gene silencing	7	58
3019	GO:0010618	aerenchyma formation	7	45
3020	GO:0010620	negative regulation of transcription by transcription factor catabolism	7	32
3021	GO:0010631	epithelial cell migration	7	25
3022	GO:0010638	positive regulation of organelle organization	7	25
3023	GO:0014045	establishment of endothelial blood-brain barrier	7	38
3024	GO:0014068	positive regulation of phosphatidylinositol 3-kinase cascade	7	43
3025	GO:0015684	ferrous iron transport	7	9
3026	GO:0015773	raffinose transport	7	9
3027	GO:0015780	nucleotide-sugar transport	7	55

3028	GO:0015812	gamma-aminobutyric acid transport	7	34
3029	GO:0015822	ornithine transport	7	22
3030	GO:0015858	nucleoside transport	7	41
3031	GO:0015885	5-formyltetrahydrofolate transport	7	22
3032	GO:0016052	carbohydrate catabolic process	7	29
3033	GO:0016331	morphogenesis of embryonic epithelium	7	21
3034	GO:0016444	somatic cell DNA recombination	7	27
3035	GO:0017196	N-terminal peptidyl-methionine acetylation	7	72
3036	GO:0018027	peptidyl-lysine dimethylation	7	51
3037	GO:0019439	aromatic compound catabolic process	7	21
3038	GO:0019483	beta-alanine biosynthetic process	7	19
3039	GO:0019566	arabinose metabolic process	7	47
3040	GO:0019567	arabinose biosynthetic process	7	45
3041	GO:0019877	diaminopimelate biosynthetic process	7	40
3042	GO:0021510	spinal cord development	7	44
3043	GO:0021954	central nervous system neuron development	7	98
3044	GO:0030011	maintenance of cell polarity	7	36
3045	GO:0030029	actin filament-based process	7	29
3046	GO:0030318	melanocyte differentiation	7	33
3047	GO:0030330	DNA damage response, signal transduction by p53 class mediator	7	42
3048	GO:0030491	heteroduplex formation	7	39
3049	GO:0030514	negative regulation of BMP signaling pathway	7	49
3050	GO:0030901	midbrain development	7	85
3051	GO:0031048	chromatin silencing by small RNA	7	33
3052	GO:0031119	tRNA pseudouridine synthesis	7	46
3053	GO:0031120	snRNA pseudouridine synthesis	7	23
3054	GO:0031572	G2/M transition DNA damage checkpoint	7	28
3055	GO:0031642	negative regulation of myelination	7	28
3056	GO:0031987	locomotion involved in locomotory behavior	7	38
3057	GO:0032006	regulation of TOR signaling cascade	7	17
3058	GO:0032012	regulation of ARF protein signal transduction	7	41
3059	GO:0032252	secretory granule localization	7	22
3060	GO:0032258	CVT pathway	7	22
3061	GO:0032534	regulation of microvillus assembly	7	66
3062	GO:0032776	DNA methylation on cytosine	7	46
3063	GO:0033077	T cell differentiation in thymus	7	13
3064	GO:0033306	phytol metabolic process	7	55
3065	GO:0033481	galacturonate biosynthetic process	7	10
3066	GO:0033595	response to genistein	7	37
3067	GO:0034063	stress granule assembly	7	41
3068	GO:0034101	erythrocyte homeostasis	7	10
3069	GO:0034394	protein localization at cell surface	7	67
3070	GO:0034427	nuclear-transcribed mRNA catabolic process, exonucleolytic, 3'-5'	7	40
3071	GO:0034476	U5 snRNA 3'-end processing	7	52
3072	GO:0034614	cellular response to reactive oxygen species	7	48
3073	GO:0034696	response to prostaglandin F stimulus	7	45
3074	GO:0034721	histone H3-K4 demethylation, trimethyl-H3-K4-specific	7	51
3075	GO:0035520	monoubiquitinated protein deubiquitination	7	81
3076	GO:0035719	tRNA import into nucleus	7	23
3077	GO:0035948	positive regulation of gluconeogenesis by positive regulation of transcription from RNA polymerase II promoter	7	21
3078	GO:0036092	phosphatidylinositol-3-phosphate biosynthetic process	7	88
3079	GO:0040012	regulation of locomotion	7	44
3080	GO:0042073	intraflagellar transport	7	66
3081	GO:0042435	indole-containing compound biosynthetic process	7	15
3082	GO:0042491	auditory receptor cell differentiation	7	33
3083	GO:0042891	antibiotic transport	7	13
3084	GO:0042989	sequestering of actin monomers	7	34

3085	GO:0043097	pyrimidine nucleoside salvage	7	21
3086	GO:0043149	stress fiber assembly	7	57
3087	GO:0043181	vacuolar sequestering	7	41
3088	GO:0043269	regulation of ion transport	7	13
3089	GO:0043328	protein targeting to vacuole involved in ubiquitin-dependent protein catabolic process via the multivesicular body sorting pathway	7	24
3090	GO:0043604	amide biosynthetic process	7	23
3091	GO:0043687	post-translational protein modification	7	48
3092	GO:0043953	protein transport by the Tat complex	7	40
3093	GO:0044154	histone H3-K14 acetylation	7	62
3094	GO:0044210	'de novo' CTP biosynthetic process	7	75
3095	GO:0044257	cellular protein catabolic process	7	34
3096	GO:0044344	cellular response to fibroblast growth factor stimulus	7	27
3097	GO:0044416	induction by symbiont of host defense response	7	38
3098	GO:0045033	peroxisome inheritance	7	45
3099	GO:0045047	protein targeting to ER	7	16
3100	GO:0045089	positive regulation of innate immune response	7	51
3101	GO:0045747	positive regulation of Notch signaling pathway	7	42
3102	GO:0045920	negative regulation of exocytosis	7	29
3103	GO:0045943	positive regulation of transcription from RNA polymerase I promoter	7	29
3104	GO:0045947	negative regulation of translational initiation	7	14
3105	GO:0046168	glycerol-3-phosphate catabolic process	7	36
3106	GO:0046394	carboxylic acid biosynthetic process	7	13
3107	GO:0046512	sphingosine biosynthetic process	7	30
3108	GO:0046598	positive regulation of viral entry into host cell	7	21
3109	GO:0046653	tetrahydrofolate metabolic process	7	48
3110	GO:0046822	regulation of nucleocytoplasmic transport	7	36
3111	GO:0046951	ketone body biosynthetic process	7	47
3112	GO:0048102	autophagic cell death	7	36
3113	GO:0048263	determination of dorsal identity	7	27
3114	GO:0048313	Golgi inheritance	7	45
3115	GO:0048315	conidium formation	7	29
3116	GO:0048515	spermatid differentiation	7	30
3117	GO:0048578	positive regulation of long-day photoperiodism, flowering	7	34
3118	GO:0048699	generation of neurons	7	21
3119	GO:0048701	embryonic cranial skeleton morphogenesis	7	70
3120	GO:0048705	skeletal system morphogenesis	7	32
3121	GO:0048749	compound eye development	7	28
3122	GO:0048754	branching morphogenesis of a tube	7	23
3123	GO:0050804	regulation of synaptic transmission	7	76
3124	GO:0051209	release of sequestered calcium ion into cytosol	7	31
3125	GO:0051336	regulation of hydrolase activity	7	7
3126	GO:0051501	diterpene phytoalexin metabolic process	7	22
3127	GO:0051569	regulation of histone H3-K4 methylation	7	32
3128	GO:0051639	actin filament network formation	7	42
3129	GO:0051642	centrosome localization	7	29
3130	GO:0051958	methotrexate transport	7	22
3131	GO:0051967	negative regulation of synaptic transmission, glutamatergic	7	63
3132	GO:0052545	callose localization	7	20
3133	GO:0055063	sulfate ion homeostasis	7	37
3134	GO:0060122	inner ear receptor stereocilium organization	7	33
3135	GO:0060216	definitive hemopoiesis	7	20
3136	GO:0060303	regulation of nucleosome density	7	80
3137	GO:0060411	cardiac septum morphogenesis	7	51
3138	GO:0060567	negative regulation of DNA-dependent transcription, termination	7	19
3139	GO:0060964	regulation of gene silencing by miRNA	7	38

3140	GO:0061002	negative regulation of dendritic spine morphogenesis	7	39
3141	GO:0061051	positive regulation of cell growth involved in cardiac muscle cell development	7	26
3142	GO:0070317	negative regulation of G0 to G1 transition	7	43
3143	GO:0070651	nonfunctional rRNA decay	7	34
3144	GO:0070682	proteasome regulatory particle assembly	7	17
3145	GO:0070791	cleistothecium development	7	14
3146	GO:0070922	small RNA loading onto RISC	7	21
3147	GO:0070925	organelle assembly	7	91
3148	GO:0071028	nuclear mRNA surveillance	7	41
3149	GO:0071236	cellular response to antibiotic	7	33
3150	GO:0071346	cellular response to interferon-gamma	7	22
3151	GO:0071361	cellular response to ethanol	7	27
3152	GO:0071372	cellular response to follicle-stimulating hormone stimulus	7	44
3153	GO:0071455	cellular response to hyperoxia	7	32
3154	GO:0071461	cellular response to redox state	7	62
3155	GO:0071465	cellular response to desiccation	7	9
3156	GO:0071476	cellular hypotonic response	7	94
3157	GO:0071900	regulation of protein serine/threonine kinase activity	7	49
3158	GO:0071940	fungal-type cell wall assembly	7	72
3159	GO:0072384	organelle transport along microtubule	7	26
3160	GO:0072520	seminiferous tubule development	7	57
3161	GO:0080009	mRNA methylation	7	40
3162	GO:0080040	positive regulation of cellular response to phosphate starvation	7	30
3163	GO:0080093	regulation of photorespiration	7	23
3164	GO:0086013	membrane repolarization involved in regulation of cardiac muscle cell action potential	7	36
3165	GO:0090068	positive regulation of cell cycle process	7	67
3166	GO:0090162	establishment of epithelial cell polarity	7	36
3167	GO:0090188	negative regulation of pancreatic juice secretion	7	77
3168	GO:0090204	protein localization to nuclear pore	7	18
3169	GO:0090309	positive regulation of methylation-dependent chromatin silencing	7	24
3170	GO:0090385	phagosome-lysosome fusion	7	11
3171	GO:0097009	energy homeostasis	7	38
3172	GO:0097185	cellular response to azide	7	11
3173	GO:0097295	morphine biosynthetic process	7	18
3174	GO:1900036	positive regulation of cellular response to heat	7	68
3175	GO:1900182	positive regulation of protein localization to nucleus	7	43
3176	GO:2000023	regulation of lateral root development	7	81
3177	GO:2000039	regulation of trichome morphogenesis	7	43
3178	GO:2000045	regulation of G1/S transition of mitotic cell cycle	7	68
3179	GO:2000279	negative regulation of DNA biosynthetic process	7	32
3180	GO:2000370	positive regulation of clathrin-mediated endocytosis	7	59
3181	GO:2000623	negative regulation of nuclear-transcribed mRNA catabolic process, nonsense-mediated decay	7	22
3182	GO:2000637	positive regulation of gene silencing by miRNA	7	42
3183	GO:2000641	regulation of early endosome to late endosome transport	7	69
3184	GO:0000076	DNA replication checkpoint	6	18
3185	GO:0000372	Group I intron splicing	6	53
3186	GO:0000414	regulation of histone H3-K36 methylation	6	82
3187	GO:0000738	DNA catabolic process, exonucleolytic	6	49
3188	GO:0000751	cell cycle arrest in response to pheromone	6	16
3189	GO:0000771	agglutination involved in conjugation	6	24
3190	GO:0000819	sister chromatid segregation	6	32
3191	GO:0001174	transcriptional start site selection at RNA polymerase II promoter	6	33
3192	GO:0001178	regulation of transcriptional start site selection at RNA polymerase II promoter	6	78

3193	GO:0001556	oocyte maturation	6	24
3194	GO:0001560	regulation of cell growth by extracellular stimulus	6	19
3195	GO:0001789	G-protein coupled receptor signaling pathway, coupled to S1P second messenger	6	32
3196	GO:0001832	blastocyst growth	6	45
3197	GO:0001835	blastocyst hatching	6	45
3198	GO:0003064	regulation of heart rate by hormone	6	12
3199	GO:0003151	outflow tract morphogenesis	6	45
3200	GO:0003300	cardiac muscle hypertrophy	6	41
3201	GO:0006082	organic acid metabolic process	6	6
3202	GO:0006116	NADH oxidation	6	23
3203	GO:0006145	purine nucleobase catabolic process	6	27
3204	GO:0006183	GTP biosynthetic process	6	21
3205	GO:0006222	UMP biosynthetic process	6	24
3206	GO:0006228	UTP biosynthetic process	6	21
3207	GO:0006241	CTP biosynthetic process	6	21
3208	GO:0006287	base-excision repair, gap-filling	6	63
3209	GO:0006323	DNA packaging	6	33
3210	GO:0006360	transcription from RNA polymerase I promoter	6	33
3211	GO:0006424	glutamyl-tRNA aminoacylation	6	37
3212	GO:0006433	prolyl-tRNA aminoacylation	6	18
3213	GO:0006490	oligosaccharide-lipid intermediate biosynthetic process	6	39
3214	GO:0006565	L-serine catabolic process	6	14
3215	GO:0006567	threonine catabolic process	6	39
3216	GO:0006572	tyrosine catabolic process	6	53
3217	GO:0006624	vacuolar protein processing	6	48
3218	GO:0006625	protein targeting to peroxisome	6	39
3219	GO:0006654	phosphatidic acid biosynthetic process	6	43
3220	GO:0006721	terpenoid metabolic process	6	15
3221	GO:0006723	cuticle hydrocarbon biosynthetic process	6	29
3222	GO:0006788	heme oxidation	6	18
3223	GO:0006851	mitochondrial calcium ion transport	6	21
3224	GO:0006936	muscle contraction	6	34
3225	GO:0007009	plasma membrane organization	6	29
3226	GO:0007041	lysosomal transport	6	25
3227	GO:0007042	lysosomal lumen acidification	6	9
3228	GO:0007060	male meiosis chromosome segregation	6	33
3229	GO:0007290	spermatid nucleus elongation	6	31
3230	GO:0007293	germarium-derived egg chamber formation	6	16
3231	GO:0007301	female germline ring canal formation	6	17
3232	GO:0007379	segment specification	6	45
3233	GO:0008039	synaptic target recognition	6	22
3234	GO:0008616	queuosine biosynthetic process	6	32
3235	GO:0009100	glycoprotein metabolic process	6	32
3236	GO:0009143	nucleoside triphosphate catabolic process	6	9
3237	GO:0009165	nucleotide biosynthetic process	6	27
3238	GO:0009436	glyoxylate catabolic process	6	25
3239	GO:0009584	detection of visible light	6	29
3240	GO:0009836	fruit ripening, climacteric	6	16
3241	GO:0009889	regulation of biosynthetic process	6	21
3242	GO:0009892	negative regulation of metabolic process	6	12
3243	GO:0009943	adaxial/abaxial axis specification	6	32
3244	GO:0009972	cytidine deamination	6	9
3245	GO:0010019	chloroplast-nucleus signaling pathway	6	49
3246	GO:0010166	wax metabolic process	6	12
3247	GO:0010198	synergid death	6	9
3248	GO:0010273	detoxification of copper ion	6	27
3249	GO:0010364	regulation of ethylene biosynthetic process	6	36
3250	GO:0010377	guard cell fate commitment	6	27
3251	GO:0010387	signalosome assembly	6	24

3252	GO:0010442	guard cell morphogenesis	6	29
3253	GO:0010636	positive regulation of mitochondrial fusion	6	37
3254	GO:0010664	negative regulation of striated muscle cell apoptotic process	6	17
3255	GO:0010793	regulation of mRNA export from nucleus	6	81
3256	GO:0010826	negative regulation of centrosome duplication	6	23
3257	GO:0010827	regulation of glucose transport	6	31
3258	GO:0010880	regulation of release of sequestered calcium ion into cytosol by sarcoplasmic reticulum	6	23
3259	GO:0010930	negative regulation of auxin mediated signaling pathway	6	24
3260	GO:0010951	negative regulation of endopeptidase activity	6	16
3261	GO:0015678	high-affinity copper ion transport	6	6
3262	GO:0015701	bicarbonate transport	6	24
3263	GO:0015708	silicate transport	6	17
3264	GO:0015819	lysine transport	6	14
3265	GO:0015837	amine transport	6	20
3266	GO:0015846	polyamine transport	6	20
3267	GO:0015868	purine ribonucleotide transport	6	23
3268	GO:0015878	biotin transport	6	36
3269	GO:0016104	triterpenoid biosynthetic process	6	13
3270	GO:0016119	carotene metabolic process	6	35
3271	GO:0016259	selenocysteine metabolic process	6	13
3272	GO:0018206	peptidyl-methionine modification	6	46
3273	GO:0018344	protein geranylgeranylation	6	26
3274	GO:0018992	germ-line sex determination	6	61
3275	GO:0019061	uncoating of virus	6	29
3276	GO:0019064	viral envelope fusion with host membrane	6	29
3277	GO:0019079	viral genome replication	6	12
3278	GO:0019371	cyclooxygenase pathway	6	20
3279	GO:0019655	glucose catabolic process to ethanol	6	23
3280	GO:0019896	axon transport of mitochondrion	6	38
3281	GO:0019919	peptidyl-arginine methylation, to asymmetrical-dimethyl arginine	6	26
3282	GO:0021522	spinal cord motor neuron differentiation	6	35
3283	GO:0021860	pyramidal neuron development	6	35
3284	GO:0022416	chaeta development	6	39
3285	GO:0022617	extracellular matrix disassembly	6	41
3286	GO:0030073	insulin secretion	6	23
3287	GO:0030166	proteoglycan biosynthetic process	6	31
3288	GO:0030183	B cell differentiation	6	27
3289	GO:0030239	myofibril assembly	6	17
3290	GO:0030518	intracellular steroid hormone receptor signaling pathway	6	38
3291	GO:0030833	regulation of actin filament polymerization	6	31
3292	GO:0030846	termination of RNA polymerase II transcription, poly(A)-coupled	6	28
3293	GO:0030847	termination of RNA polymerase II transcription, exosome-dependent	6	28
3294	GO:0030853	negative regulation of granulocyte differentiation	6	36
3295	GO:0030950	establishment or maintenance of actin cytoskeleton polarity	6	48
3296	GO:0031058	positive regulation of histone modification	6	18
3297	GO:0031065	positive regulation of histone deacetylation	6	64
3298	GO:0031129	inductive cell-cell signaling	6	41
3299	GO:0031338	regulation of vesicle fusion	6	53
3300	GO:0031340	positive regulation of vesicle fusion	6	30
3301	GO:0031346	positive regulation of cell projection organization	6	37
3302	GO:0031496	positive regulation of mating type switching	6	29
3303	GO:0031508	centromeric heterochromatin assembly	6	18
3304	GO:0031573	intra-S DNA damage checkpoint	6	69
3305	GO:0031579	membrane raft organization	6	47
3306	GO:0031937	positive regulation of chromatin silencing	6	30

3307	GO:0031939	negative regulation of chromatin silencing at telomere	6	39
3308	GO:0031991	regulation of actomyosin contractile ring contraction	6	17
3309	GO:0032020	ISG15-protein conjugation	6	26
3310	GO:0032204	regulation of telomere maintenance	6	34
3311	GO:0032228	regulation of synaptic transmission, GABAergic	6	34
3312	GO:0032376	positive regulation of cholesterol transport	6	36
3313	GO:0032412	regulation of ion transmembrane transporter activity	6	6
3314	GO:0032439	endosome localization	6	33
3315	GO:0032482	Rab protein signal transduction	6	29
3316	GO:0032509	endosome transport via multivesicular body sorting pathway	6	52
3317	GO:0032688	negative regulation of interferon-beta production	6	28
3318	GO:0032782	bile acid secretion	6	36
3319	GO:0032973	amino acid export	6	11
3320	GO:0033108	mitochondrial respiratory chain complex assembly	6	40
3321	GO:0033135	regulation of peptidyl-serine phosphorylation	6	82
3322	GO:0033310	chlorophyll a catabolic process	6	35
3323	GO:0033617	mitochondrial respiratory chain complex IV assembly	6	40
3324	GO:0034058	endosomal vesicle fusion	6	17
3325	GO:0034115	negative regulation of heterotypic cell-cell adhesion	6	10
3326	GO:0034332	adherens junction organization	6	41
3327	GO:0034502	protein localization to chromosome	6	33
3328	GO:0034628	de novo NAD biosynthetic process from aspartate	6	35
3329	GO:0034728	nucleosome organization	6	62
3330	GO:0035041	sperm chromatin decondensation	6	48
3331	GO:0035136	forelimb morphogenesis	6	14
3332	GO:0035269	protein O-linked mannosylation	6	14
3333	GO:0035315	hair cell differentiation	6	46
3334	GO:0035587	purinergic receptor signaling pathway	6	39
3335	GO:0035621	ER to Golgi ceramide transport	6	35
3336	GO:0035865	cellular response to potassium ion	6	7
3337	GO:0035887	aortic smooth muscle cell differentiation	6	45
3338	GO:0035973	aggrephagy	6	40
3339	GO:0036289	peptidyl-serine autophosphorylation	6	91
3340	GO:0038018	Wnt receptor catabolic process	6	17
3341	GO:0040001	establishment of mitotic spindle localization	6	12
3342	GO:0040016	embryonic cleavage	6	15
3343	GO:0042098	T cell proliferation	6	40
3344	GO:0042184	xylene catabolic process	6	14
3345	GO:0042203	toluene catabolic process	6	14
3346	GO:0042351	'de novo' GDP-L-fucose biosynthetic process	6	7
3347	GO:0042372	phylloquinone biosynthetic process	6	21
3348	GO:0042373	vitamin K metabolic process	6	16
3349	GO:0042428	serotonin metabolic process	6	28
3350	GO:0042574	retinal metabolic process	6	21
3351	GO:0042762	regulation of sulfur metabolic process	6	23
3352	GO:0043100	pyrimidine nucleobase salvage	6	42
3353	GO:0043129	surfactant homeostasis	6	25
3354	GO:0043249	erythrocyte maturation	6	23
3355	GO:0043255	regulation of carbohydrate biosynthetic process	6	10
3356	GO:0043268	positive regulation of potassium ion transport	6	49
3357	GO:0043403	skeletal muscle tissue regeneration	6	17
3358	GO:0043467	regulation of generation of precursor metabolites and energy	6	17
3359	GO:0043517	positive regulation of DNA damage response, signal transduction by p53 class mediator	6	69
3360	GO:0043519	regulation of myosin II filament organization	6	21
3361	GO:0043936	asexual sporulation resulting in formation of a cellular spore	6	28
3362	GO:0043971	histone H3-K18 acetylation	6	54
3363	GO:0043972	histone H3-K23 acetylation	6	54

3364	GO:0044109	cellular alcohol catabolic process	6	29
3365	GO:0044375	regulation of peroxisome size	6	31
3366	GO:0045013	carbon catabolite repression of transcription	6	27
3367	GO:0045128	negative regulation of reciprocal meiotic recombination	6	48
3368	GO:0045138	tail tip morphogenesis	6	41
3369	GO:0045167	asymmetric protein localization involved in cell fate determination	6	42
3370	GO:0045185	maintenance of protein location	6	20
3371	GO:0045329	carnitine biosynthetic process	6	15
3372	GO:0045339	farnesyl diphosphate catabolic process	6	33
3373	GO:0045415	negative regulation of interleukin-8 biosynthetic process	6	10
3374	GO:0045451	pole plasm oskar mRNA localization	6	22
3375	GO:0045638	negative regulation of myeloid cell differentiation	6	21
3376	GO:0045648	positive regulation of erythrocyte differentiation	6	26
3377	GO:0045666	positive regulation of neuron differentiation	6	27
3378	GO:0045725	positive regulation of glycogen biosynthetic process	6	34
3379	GO:0045738	negative regulation of DNA repair	6	23
3380	GO:0045820	negative regulation of glycolysis	6	51
3381	GO:0045950	negative regulation of mitotic recombination	6	90
3382	GO:0046033	AMP metabolic process	6	49
3383	GO:0046109	uridine biosynthetic process	6	9
3384	GO:0046185	aldehyde catabolic process	6	14
3385	GO:0046272	stilbene catabolic process	6	10
3386	GO:0046287	isoflavonoid metabolic process	6	6
3387	GO:0046325	negative regulation of glucose import	6	37
3388	GO:0046718	viral entry into host cell	6	8
3389	GO:0046719	regulation of viral protein levels in host cell	6	38
3390	GO:0046740	spread of virus in host, cell to cell	6	9
3391	GO:0048016	inositol phosphate-mediated signaling	6	26
3392	GO:0048140	male germ-line cyst encapsulation	6	10
3393	GO:0048219	inter-Golgi cisterna vesicle-mediated transport	6	24
3394	GO:0048286	lung alveolus development	6	27
3395	GO:0048451	petal formation	6	80
3396	GO:0048479	style development	6	12
3397	GO:0048563	post-embryonic organ morphogenesis	6	7
3398	GO:0048582	positive regulation of post-embryonic development	6	9
3399	GO:0048633	positive regulation of skeletal muscle tissue growth	6	17
3400	GO:0048678	response to axon injury	6	16
3401	GO:0048680	positive regulation of axon regeneration	6	64
3402	GO:0048730	epidermis morphogenesis	6	45
3403	GO:0048821	erythrocyte development	6	16
3404	GO:0050687	negative regulation of defense response to virus	6	46
3405	GO:0050728	negative regulation of inflammatory response	6	12
3406	GO:0050769	positive regulation of neurogenesis	6	38
3407	GO:0051093	negative regulation of developmental process	6	25
3408	GO:0051099	positive regulation of binding	6	34
3409	GO:0051101	regulation of DNA binding	6	38
3410	GO:0051124	synaptic growth at neuromuscular junction	6	47
3411	GO:0051127	positive regulation of actin nucleation	6	57
3412	GO:0051172	negative regulation of nitrogen compound metabolic process	6	20
3413	GO:0051176	positive regulation of sulfur metabolic process	6	35
3414	GO:0051256	spindle midzone assembly involved in mitosis	6	33
3415	GO:0051307	meiotic chromosome separation	6	63
3416	GO:0051338	regulation of transferase activity	6	32
3417	GO:0051345	positive regulation of hydrolase activity	6	61
3418	GO:0051347	positive regulation of transferase activity	6	6
3419	GO:0051353	positive regulation of oxidoreductase activity	6	18
3420	GO:0051451	myoblast migration	6	17
3421	GO:0051560	mitochondrial calcium ion homeostasis	6	27

3422	GO:0051561	elevation of mitochondrial calcium ion concentration	6	25
3423	GO:0051640	organelle localization	6	19
3424	GO:0051650	establishment of vesicle localization	6	16
3425	GO:0051895	negative regulation of focal adhesion assembly	6	48
3426	GO:0051974	negative regulation of telomerase activity	6	69
3427	GO:0052541	plant-type cell wall cellulose metabolic process	6	29
3428	GO:0052746	inositol phosphorylation	6	23
3429	GO:0055129	L-proline biosynthetic process	6	26
3430	GO:0060003	copper ion export	6	28
3431	GO:0060047	heart contraction	6	21
3432	GO:0060118	vestibular receptor cell development	6	7
3433	GO:0060195	negative regulation of antisense RNA transcription	6	46
3434	GO:0060267	positive regulation of respiratory burst	6	22
3435	GO:0060320	rejection of self pollen	6	11
3436	GO:0060334	regulation of interferon-gamma-mediated signaling pathway	6	61
3437	GO:0060347	heart trabecula formation	6	45
3438	GO:0060444	branching involved in mammary gland duct morphogenesis	6	56
3439	GO:0060560	developmental growth involved in morphogenesis	6	9
3440	GO:0060562	epithelial tube morphogenesis	6	66
3441	GO:0060586	multicellular organismal iron ion homeostasis	6	19
3442	GO:0060696	regulation of phospholipid catabolic process	6	20
3443	GO:0060761	negative regulation of response to cytokine stimulus	6	10
3444	GO:0060763	mammary duct terminal end bud growth	6	56
3445	GO:0061038	uterus morphogenesis	6	56
3446	GO:0061053	somite development	6	15
3447	GO:0061384	heart trabecula morphogenesis	6	17
3448	GO:0061404	positive regulation of transcription from RNA polymerase II promoter in response to increased salt	6	60
3449	GO:0070212	protein poly-ADP-ribosylation	6	60
3450	GO:0070306	lens fiber cell differentiation	6	27
3451	GO:0070307	lens fiber cell development	6	45
3452	GO:0070375	ERK5 cascade	6	10
3453	GO:0070498	interleukin-1-mediated signaling pathway	6	24
3454	GO:0070613	regulation of protein processing	6	36
3455	GO:0070842	aggresome assembly	6	19
3456	GO:0070904	transepithelial L-ascorbic acid transport	6	29
3457	GO:0070918	production of small RNA involved in gene silencing by RNA	6	44
3458	GO:0071158	positive regulation of cell cycle arrest	6	21
3459	GO:0071243	cellular response to arsenic-containing substance	6	22
3460	GO:0071245	cellular response to carbon monoxide	6	23
3461	GO:0071286	cellular response to magnesium ion	6	22
3462	GO:0071289	cellular response to nickel ion	6	8
3463	GO:0071294	cellular response to zinc ion	6	18
3464	GO:0071322	cellular response to carbohydrate stimulus	6	19
3465	GO:0071360	cellular response to exogenous dsRNA	6	24
3466	GO:0071370	cellular response to gibberellin stimulus	6	13
3467	GO:0071385	cellular response to glucocorticoid stimulus	6	33
3468	GO:0071423	malate transmembrane transport	6	28
3469	GO:0071426	ribonucleoprotein complex export from nucleus	6	43
3470	GO:0071435	potassium ion export	6	14
3471	GO:0071441	negative regulation of histone H3-K14 acetylation	6	78
3472	GO:0071478	cellular response to radiation	6	55
3473	GO:0071586	CAAX-box protein processing	6	26
3474	GO:0071822	protein complex subunit organization	6	44
3475	GO:0072389	flavin adenine dinucleotide catabolic process	6	12
3476	GO:0072722	response to amitrole	6	11
3477	GO:0080151	positive regulation of salicylic acid mediated signaling pathway	6	46

3478	GO:0090002	establishment of protein localization in plasma membrane	6	25
3479	GO:0090035	positive regulation of chaperone-mediated protein complex assembly	6	32
3480	GO:0090160	Golgi to lysosome transport	6	48
3481	GO:0090181	regulation of cholesterol metabolic process	6	10
3482	GO:0090284	positive regulation of protein glycosylation in Golgi	6	25
3483	GO:0090316	positive regulation of intracellular protein transport	6	26
3484	GO:0090359	negative regulation of abscisic acid biosynthetic process	6	12
3485	GO:0097034	mitochondrial respiratory chain complex IV biogenesis	6	51
3486	GO:0097054	L-glutamate biosynthetic process	6	26
3487	GO:1900050	negative regulation of histone exchange	6	78
3488	GO:1900078	positive regulation of cellular response to insulin stimulus	6	21
3489	GO:1900102	negative regulation of endoplasmic reticulum unfolded protein response	6	9
3490	GO:1900126	negative regulation of hyaluronan biosynthetic process	6	60
3491	GO:1900189	positive regulation of cell adhesion involved in single-species biofilm formation	6	28
3492	GO:1900233	positive regulation of single-species biofilm formation on inanimate substrate	6	47
3493	GO:1900363	regulation of mRNA polyadenylation	6	27
3494	GO:1900433	positive regulation of filamentous growth of a population of unicellular organisms in response to heat	6	47
3495	GO:2000001	regulation of DNA damage checkpoint	6	36
3496	GO:2000051	negative regulation of non-canonical Wnt receptor signaling pathway	6	17
3497	GO:2000060	positive regulation of protein ubiquitination involved in ubiquitin-dependent protein catabolic process	6	19
3498	GO:2000138	positive regulation of cell proliferation involved in heart morphogenesis	6	17
3499	GO:2000185	regulation of phosphate transmembrane transport	6	32
3500	GO:2000197	regulation of ribonucleoprotein complex localization	6	42
3501	GO:2000342	negative regulation of chemokine (C-X-C motif) ligand 2 production	6	10
3502	GO:2000616	negative regulation of histone H3-K9 acetylation	6	78
3503	GO:2000653	regulation of genetic imprinting	6	32
3504	GO:2000687	negative regulation of rubidium ion transmembrane transporter activity	6	73
3505	GO:2000727	positive regulation of cardiac muscle cell differentiation	6	25
3506	GO:2000819	regulation of nucleotide-excision repair	6	36
3507	GO:2000864	regulation of estradiol secretion	6	56
3508	GO:2001009	regulation of plant-type cell wall cellulose biosynthetic process	6	35
3509	GO:2001171	positive regulation of ATP biosynthetic process	6	12
3510	GO:2001272	positive regulation of cysteine-type endopeptidase activity involved in execution phase of apoptosis	6	23
3511	GO:0000018	regulation of DNA recombination	5	16
3512	GO:0000019	regulation of mitotic recombination	5	42
3513	GO:0000032	cell wall mannoprotein biosynthetic process	5	27
3514	GO:0000073	spindle pole body separation	5	12
3515	GO:0000301	retrograde transport, vesicle recycling within Golgi	5	23
3516	GO:0000305	response to oxygen radical	5	22
3517	GO:0000335	negative regulation of transposition, DNA-mediated	5	7
3518	GO:0000349	generation of catalytic spliceosome for first transesterification step	5	8
3519	GO:0000706	meiotic DNA double-strand break processing	5	46
3520	GO:0000910	cytokinesis	5	25
3521	GO:0001505	regulation of neurotransmitter levels	5	34
3522	GO:0001510	RNA methylation	5	33
3523	GO:0001552	ovarian follicle atresia	5	14
3524	GO:0001654	eye development	5	24

3525	GO:0001700	embryonic development via the syncytial blastoderm	5	44
3526	GO:0001763	morphogenesis of a branching structure	5	67
3527	GO:0001816	cytokine production	5	23
3528	GO:0001825	blastocyst formation	5	11
3529	GO:0001938	positive regulation of endothelial cell proliferation	5	33
3530	GO:0001959	regulation of cytokine-mediated signaling pathway	5	14
3531	GO:0002021	response to dietary excess	5	25
3532	GO:0002090	regulation of receptor internalization	5	24
3533	GO:0002188	translation reinitiation	5	13
3534	GO:0002218	activation of innate immune response	5	19
3535	GO:0002240	response to molecule of oomycetes origin	5	13
3536	GO:0002309	T cell proliferation involved in immune response	5	13
3537	GO:0002369	T cell cytokine production	5	16
3538	GO:0002931	response to ischemia	5	16
3539	GO:0003374	dynamin polymerization involved in mitochondrial fission	5	26
3540	GO:0006065	UDP-glucuronate biosynthetic process	5	13
3541	GO:0006104	succinyl-CoA metabolic process	5	18
3542	GO:0006124	ferredoxin metabolic process	5	6
3543	GO:0006210	thymine catabolic process	5	15
3544	GO:0006226	dUMP biosynthetic process	5	7
3545	GO:0006383	transcription from RNA polymerase III promoter	5	31
3546	GO:0006418	tRNA aminoacylation for protein translation	5	39
3547	GO:0006422	aspartyl-tRNA aminoacylation	5	16
3548	GO:0006527	arginine catabolic process	5	12
3549	GO:0006542	glutamine biosynthetic process	5	43
3550	GO:0006556	S-adenosylmethionine biosynthetic process	5	12
3551	GO:0006596	polyamine biosynthetic process	5	17
3552	GO:0006608	snRNP protein import into nucleus	5	12
3553	GO:0006690	icosanoid metabolic process	5	6
3554	GO:0006732	coenzyme metabolic process	5	16
3555	GO:0006753	nucleoside phosphate metabolic process	5	20
3556	GO:0006779	porphyrin-containing compound biosynthetic process	5	41
3557	GO:0006935	chemotaxis	5	34
3558	GO:0006941	striated muscle contraction	5	11
3559	GO:0007000	nucleolus organization	5	51
3560	GO:0007114	cell budding	5	17
3561	GO:0007187	G-protein coupled receptor signaling pathway, coupled to cyclic nucleotide second messenger	5	27
3562	GO:0007250	activation of NF-kappaB-inducing kinase activity	5	15
3563	GO:0007391	dorsal closure	5	17
3564	GO:0007406	negative regulation of neuroblast proliferation	5	52
3565	GO:0007412	axon target recognition	5	36
3566	GO:0007427	epithelial cell migration, open tracheal system	5	13
3567	GO:0007428	primary branching, open tracheal system	5	13
3568	GO:0007509	mesoderm migration involved in gastrulation	5	13
3569	GO:0007525	somatic muscle development	5	22
3570	GO:0007533	mating type switching	5	13
3571	GO:0007585	respiratory gaseous exchange	5	28
3572	GO:0007602	phototransduction	5	26
3573	GO:0007616	long-term memory	5	18
3574	GO:0008153	para-aminobenzoic acid biosynthetic process	5	41
3575	GO:0008347	glial cell migration	5	38
3576	GO:0008625	induction of apoptosis via death domain receptors	5	30
3577	GO:0009063	cellular amino acid catabolic process	5	25
3578	GO:0009101	glycoprotein biosynthetic process	5	7
3579	GO:0009142	nucleoside triphosphate biosynthetic process	5	51
3580	GO:0009164	nucleoside catabolic process	5	15
3581	GO:0009186	deoxyribonucleoside diphosphate metabolic process	5	28
3582	GO:0009226	nucleotide-sugar biosynthetic process	5	36

3583	GO:0009251	glucan catabolic process	5	29
3584	GO:0009606	tropism	5	27
3585	GO:0009798	axis specification	5	6
3586	GO:0009893	positive regulation of metabolic process	5	48
3587	GO:0009953	dorsal/ventral pattern formation	5	22
3588	GO:0010040	response to iron(II) ion	5	21
3589	GO:0010088	phloem development	5	54
3590	GO:0010106	cellular response to iron ion starvation	5	34
3591	GO:0010191	mucilage metabolic process	5	11
3592	GO:0010202	response to low fluence red light stimulus	5	17
3593	GO:0010245	radial microtubular system formation	5	25
3594	GO:0010253	UDP-rhamnose biosynthetic process	5	11
3595	GO:0010266	response to vitamin B1	5	14
3596	GO:0010275	NAD(P)H dehydrogenase complex assembly	5	18
3597	GO:0010312	detoxification of zinc ion	5	38
3598	GO:0010351	lithium ion transport	5	32
3599	GO:0010493	Lewis a epitope biosynthetic process	5	24
3600	GO:0010599	production of lsiRNA involved in RNA interference	5	11
3601	GO:0010617	circadian regulation of calcium ion oscillation	5	17
3602	GO:0010697	negative regulation of spindle pole body separation	5	9
3603	GO:0010719	negative regulation of epithelial to mesenchymal transition	5	21
3604	GO:0010944	negative regulation of transcription by competitive promoter binding	5	14
3605	GO:0015679	plasma membrane copper ion transport	5	17
3606	GO:0015680	intracellular copper ion transport	5	12
3607	GO:0015694	mercury ion transport	5	19
3608	GO:0015714	phosphoenolpyruvate transport	5	17
3609	GO:0015732	prostaglandin transport	5	19
3610	GO:0015771	trehalose transport	5	14
3611	GO:0015813	L-glutamate transport	5	18
3612	GO:0015864	pyrimidine nucleoside transport	5	21
3613	GO:0016199	axon midline choice point recognition	5	13
3614	GO:0016320	endoplasmic reticulum membrane fusion	5	14
3615	GO:0016445	somatic diversification of immunoglobulins	5	15
3616	GO:0016479	negative regulation of transcription from RNA polymerase I promoter	5	38
3617	GO:0016560	protein import into peroxisome matrix, docking	5	29
3618	GO:0017012	protein-phytochromobilin linkage	5	17
3619	GO:0017145	stem cell division	5	32
3620	GO:0018343	protein farnesylation	5	34
3621	GO:0019255	glucose 1-phosphate metabolic process	5	12
3622	GO:0019346	transsulfuration	5	22
3623	GO:0019369	arachidonic acid metabolic process	5	49
3624	GO:0019373	epoxygenase P450 pathway	5	7
3625	GO:0019419	sulfate reduction	5	24
3626	GO:0019640	glucuronate catabolic process to xylulose 5-phosphate	5	50
3627	GO:0019674	NAD metabolic process	5	19
3628	GO:0019725	cellular homeostasis	5	29
3629	GO:0021695	cerebellar cortex development	5	13
3630	GO:0021854	hypothalamus development	5	35
3631	GO:0021952	central nervous system projection neuron axonogenesis	5	31
3632	GO:0022622	root system development	5	14
3633	GO:0030071	regulation of mitotic metaphase/anaphase transition	5	18
3634	GO:0030187	melatonin biosynthetic process	5	19
3635	GO:0030381	chorion-containing eggshell pattern formation	5	45
3636	GO:0030539	male genitalia development	5	22
3637	GO:0030541	plasmid partitioning	5	9
3638	GO:0030578	PML body organization	5	15
3639	GO:0030579	ubiquitin-dependent SMAD protein catabolic process	5	21
3640	GO:0030819	positive regulation of cAMP biosynthetic process	5	60

3641	GO:0030822	positive regulation of cAMP catabolic process	5	8
3642	GO:0031055	chromatin remodeling at centromere	5	29
3643	GO:0031064	negative regulation of histone deacetylation	5	21
3644	GO:0031081	nuclear pore distribution	5	28
3645	GO:0031101	fin regeneration	5	14
3646	GO:0031118	rRNA pseudouridine synthesis	5	15
3647	GO:0031222	arabinan catabolic process	5	14
3648	GO:0031291	Ran protein signal transduction	5	11
3649	GO:0031427	response to methotrexate	5	19
3650	GO:0031529	ruffle organization	5	30
3651	GO:0031538	negative regulation of anthocyanin metabolic process	5	38
3652	GO:0031629	synaptic vesicle fusion to presynaptic membrane	5	22
3653	GO:0031669	cellular response to nutrient levels	5	67
3654	GO:0031935	regulation of chromatin silencing	5	91
3655	GO:0032025	response to cobalt ion	5	25
3656	GO:0032057	negative regulation of translational initiation in response to stress	5	17
3657	GO:0032094	response to food	5	26
3658	GO:0032324	molybdopterin cofactor biosynthetic process	5	20
3659	GO:0032464	positive regulation of protein homooligomerization	5	8
3660	GO:0032495	response to muramyl dipeptide	5	9
3661	GO:0032680	regulation of tumor necrosis factor production	5	8
3662	GO:0032720	negative regulation of tumor necrosis factor production	5	17
3663	GO:0032793	positive regulation of CREB transcription factor activity	5	58
3664	GO:0032956	regulation of actin cytoskeleton organization	5	33
3665	GO:0032958	inositol phosphate biosynthetic process	5	23
3666	GO:0033044	regulation of chromosome organization	5	24
3667	GO:0033151	V(D)J recombination	5	91
3668	GO:0033182	regulation of histone ubiquitination	5	15
3669	GO:0033197	response to vitamin E	5	33
3670	GO:0033235	positive regulation of protein sumoylation	5	14
3671	GO:0033345	asparagine catabolic process via L-aspartate	5	20
3672	GO:0033353	S-adenosylmethionine cycle	5	16
3673	GO:0033356	UDP-L-arabinose metabolic process	5	14
3674	GO:0033762	response to glucagon stimulus	5	31
3675	GO:0034050	host programmed cell death induced by symbiont	5	16
3676	GO:0034243	regulation of transcription elongation from RNA polymerase II promoter	5	11
3677	GO:0034244	negative regulation of transcription elongation from RNA polymerase II promoter	5	34
3678	GO:0034315	regulation of Arp2/3 complex-mediated actin nucleation	5	24
3679	GO:0034446	substrate adhesion-dependent cell spreading	5	8
3680	GO:0034503	protein localization to nucleolar rDNA repeats	5	38
3681	GO:0034637	cellular carbohydrate biosynthetic process	5	40
3682	GO:0034755	iron ion transmembrane transport	5	36
3683	GO:0034760	negative regulation of iron ion transmembrane transport	5	17
3684	GO:0034982	mitochondrial protein processing	5	26
3685	GO:0034983	peptidyl-lysine deacetylation	5	29
3686	GO:0035076	ecdysone receptor-mediated signaling pathway	5	48
3687	GO:0035162	embryonic hemopoiesis	5	14
3688	GO:0035444	nickel cation transmembrane transport	5	43
3689	GO:0035493	SNARE complex assembly	5	5
3690	GO:0035721	intraflagellar retrograde transport	5	22
3691	GO:0035902	response to immobilization stress	5	14
3692	GO:0036120	cellular response to platelet-derived growth factor stimulus	5	17
3693	GO:0036166	phenotypic switching	5	22
3694	GO:0036290	protein trans-autophosphorylation	5	5
3695	GO:0042078	germ-line stem cell division	5	21
3696	GO:0042100	B cell proliferation	5	41

3697	GO:0042308	negative regulation of protein import into nucleus	5	21
3698	GO:0042350	GDP-L-fucose biosynthetic process	5	6
3699	GO:0042440	pigment metabolic process	5	29
3700	GO:0042474	middle ear morphogenesis	5	26
3701	GO:0042550	photosystem I stabilization	5	19
3702	GO:0042573	retinoic acid metabolic process	5	18
3703	GO:0042713	sperm ejaculation	5	24
3704	GO:0042777	plasma membrane ATP synthesis coupled proton transport	5	31
3705	GO:0042816	vitamin B6 metabolic process	5	32
3706	GO:0042823	pyridoxal phosphate biosynthetic process	5	16
3707	GO:0042843	D-xylose catabolic process	5	9
3708	GO:0042908	xenobiotic transport	5	20
3709	GO:0042993	positive regulation of transcription factor import into nucleus	5	39
3710	GO:0043093	cytokinesis by binary fission	5	24
3711	GO:0043248	proteasome assembly	5	10
3712	GO:0043270	positive regulation of ion transport	5	8
3713	GO:0043308	eosinophil degranulation	5	5
3714	GO:0043312	neutrophil degranulation	5	5
3715	GO:0043330	response to exogenous dsRNA	5	24
3716	GO:0043491	protein kinase B signaling cascade	5	46
3717	GO:0043508	negative regulation of JUN kinase activity	5	43
3718	GO:0043523	regulation of neuron apoptotic process	5	26
3719	GO:0043545	molybdopterin cofactor metabolic process	5	21
3720	GO:0043653	mitochondrial fragmentation involved in apoptotic process	5	37
3721	GO:0043903	regulation of symbiosis, encompassing mutualism through parasitism	5	16
3722	GO:0043935	sexual sporulation resulting in formation of a cellular spore	5	14
3723	GO:0043985	histone H4-R3 methylation	5	17
3724	GO:0044272	sulfur compound biosynthetic process	5	16
3725	GO:0045002	double-strand break repair via single-strand annealing	5	35
3726	GO:0045104	intermediate filament cytoskeleton organization	5	38
3727	GO:0045338	farnesyl diphosphate metabolic process	5	8
3728	GO:0045445	myoblast differentiation	5	16
3729	GO:0045461	sterigmatocystin biosynthetic process	5	5
3730	GO:0045600	positive regulation of fat cell differentiation	5	17
3731	GO:0045664	regulation of neuron differentiation	5	20
3732	GO:0045668	negative regulation of osteoblast differentiation	5	21
3733	GO:0045721	negative regulation of gluconeogenesis	5	52
3734	GO:0045745	positive regulation of G-protein coupled receptor protein signaling pathway	5	14
3735	GO:0045785	positive regulation of cell adhesion	5	22
3736	GO:0045821	positive regulation of glycolysis	5	40
3737	GO:0045880	positive regulation of smoothened signaling pathway	5	26
3738	GO:0045905	positive regulation of translational termination	5	16
3739	GO:0045917	positive regulation of complement activation	5	14
3740	GO:0045937	positive regulation of phosphate metabolic process	5	15
3741	GO:0046020	negative regulation of transcription from RNA polymerase II promoter by pheromones	5	45
3742	GO:0046081	dUTP catabolic process	5	7
3743	GO:0046085	adenosine metabolic process	5	12
3744	GO:0046134	pyrimidine nucleoside biosynthetic process	5	17
3745	GO:0046183	L-idonate catabolic process	5	6
3746	GO:0046283	anthocyanin metabolic process	5	27
3747	GO:0046296	glycolate catabolic process	5	24
3748	GO:0046597	negative regulation of viral entry into host cell	5	53
3749	GO:0046660	female sex differentiation	5	22
3750	GO:0046683	response to organophosphorus	5	34

3751	GO:0046713	borate transport	5	8
3752	GO:0046778	modification by virus of host mRNA processing	5	17
3753	GO:0046825	regulation of protein export from nucleus	5	51
3754	GO:0046833	positive regulation of RNA export from nucleus	5	55
3755	GO:0046836	glycolipid transport	5	16
3756	GO:0048146	positive regulation of fibroblast proliferation	5	37
3757	GO:0048455	stamen formation	5	21
3758	GO:0048462	carpel formation	5	14
3759	GO:0048498	establishment of petal orientation	5	18
3760	GO:0048639	positive regulation of developmental growth	5	18
3761	GO:0048700	acquisition of desiccation tolerance	5	7
3762	GO:0050680	negative regulation of epithelial cell proliferation	5	22
3763	GO:0050771	negative regulation of axonogenesis	5	32
3764	GO:0051038	negative regulation of transcription during meiosis	5	38
3765	GO:0051054	positive regulation of DNA metabolic process	5	8
3766	GO:0051100	negative regulation of binding	5	31
3767	GO:0051106	positive regulation of DNA ligation	5	24
3768	GO:0051238	sequestering of metal ion	5	24
3769	GO:0051292	nuclear pore complex assembly	5	34
3770	GO:0051296	establishment of meiotic spindle orientation	5	35
3771	GO:0051306	mitotic sister chromatid separation	5	16
3772	GO:0051354	negative regulation of oxidoreductase activity	5	23
3773	GO:0051382	kinetochore assembly	5	5
3774	GO:0051439	regulation of ubiquitin-protein ligase activity involved in mitotic cell cycle	5	20
3775	GO:0051447	negative regulation of meiotic cell cycle	5	25
3776	GO:0051461	positive regulation of corticotropin secretion	5	34
3777	GO:0051480	cytosolic calcium ion homeostasis	5	39
3778	GO:0051533	positive regulation of NFAT protein import into nucleus	5	17
3779	GO:0051602	response to electrical stimulus	5	24
3780	GO:0051668	localization within membrane	5	70
3781	GO:0051701	interaction with host	5	38
3782	GO:0051901	positive regulation of mitochondrial depolarization	5	8
3783	GO:0051937	catecholamine transport	5	12
3784	GO:0051960	regulation of nervous system development	5	37
3785	GO:0055008	cardiac muscle tissue morphogenesis	5	14
3786	GO:0055069	zinc ion homeostasis	5	30
3787	GO:0055070	copper ion homeostasis	5	33
3788	GO:0055091	phospholipid homeostasis	5	55
3789	GO:0055119	relaxation of cardiac muscle	5	34
3790	GO:0060074	synapse maturation	5	46
3791	GO:0060290	transdifferentiation	5	14
3792	GO:0060355	positive regulation of cell adhesion molecule production	5	45
3793	GO:0060391	positive regulation of SMAD protein import into nucleus	5	67
3794	GO:0060576	intestinal epithelial cell development	5	11
3795	GO:0060997	dendritic spine morphogenesis	5	25
3796	GO:0061086	negative regulation of histone H3-K27 methylation	5	9
3797	GO:0061186	negative regulation of chromatin silencing at silent mating-type cassette	5	38
3798	GO:0061188	negative regulation of chromatin silencing at rDNA	5	38
3799	GO:0070085	glycosylation	5	21
3800	GO:0070192	chromosome organization involved in meiosis	5	17
3801	GO:0070194	synaptonemal complex disassembly	5	17
3802	GO:0070327	thyroid hormone transport	5	19
3803	GO:0070345	negative regulation of fat cell proliferation	5	40
3804	GO:0070507	regulation of microtubule cytoskeleton organization	5	38
3805	GO:0070509	calcium ion import	5	31
3806	GO:0070527	platelet aggregation	5	43
3807	GO:0070593	dendrite self-avoidance	5	37
3808	GO:0070681	glutaminyl-tRNAGln biosynthesis via transamidation	5	19

3809	GO:0070783	growth of unicellular organism as a thread of attached cells	5	17
3810	GO:0071034	CUT catabolic process	5	29
3811	GO:0071168	protein localization to chromatin	5	15
3812	GO:0071174	mitotic cell cycle spindle checkpoint	5	25
3813	GO:0071258	cellular response to gravity	5	5
3814	GO:0071275	cellular response to aluminum ion	5	44
3815	GO:0071279	cellular response to cobalt ion	5	17
3816	GO:0071284	cellular response to lead ion	5	17
3817	GO:0071320	cellular response to cAMP	5	8
3818	GO:0071344	diphosphate metabolic process	5	32
3819	GO:0071380	cellular response to prostaglandin E stimulus	5	23
3820	GO:0071493	cellular response to UV-B	5	10
3821	GO:0071731	response to nitric oxide	5	14
3822	GO:0071801	regulation of podosome assembly	5	59
3823	GO:0071806	protein transmembrane transport	5	12
3824	GO:0072001	renal system development	5	13
3825	GO:0072321	chaperone-mediated protein transport	5	10
3826	GO:0072576	liver morphogenesis	5	18
3827	GO:0080038	positive regulation of cytokinin mediated signaling pathway	5	28
3828	GO:0080126	ovary septum development	5	14
3829	GO:0080165	callose deposition in phloem sieve plate	5	7
3830	GO:0080185	effector dependent induction by symbiont of host immune response	5	17
3831	GO:0086004	regulation of cardiac muscle cell contraction	5	39
3832	GO:0086005	regulation of ventricular cardiac muscle cell action potential	5	33
3833	GO:0090042	tubulin deacetylation	5	29
3834	GO:0090166	Golgi disassembly	5	16
3835	GO:0090200	positive regulation of release of cytochrome c from mitochondria	5	22
3836	GO:0090261	positive regulation of inclusion body assembly	5	13
3837	GO:0090266	regulation of mitotic cell cycle spindle assembly checkpoint	5	25
3838	GO:0090277	positive regulation of peptide hormone secretion	5	9
3839	GO:0090428	perianth development	5	18
3840	GO:0097061	dendritic spine organization	5	24
3841	GO:0097320	membrane tubulation	5	24
3842	GO:1900055	regulation of leaf senescence	5	16
3843	GO:1900153	positive regulation of nuclear-transcribed mRNA catabolic process, deadenylation-dependent decay	5	10
3844	GO:1900264	positive regulation of DNA-directed DNA polymerase activity	5	21
3845	GO:1900376	regulation of secondary metabolite biosynthetic process	5	8
3846	GO:1900409	positive regulation of cellular response to oxidative stress	5	34
3847	GO:1900458	negative regulation of brassinosteroid mediated signaling pathway	5	12
3848	GO:1900483	regulation of protein targeting to vacuolar membrane	5	5
3849	GO:1901029	negative regulation of mitochondrial outer membrane permeabilization	5	14
3850	GO:1901086	benzylpenicillin metabolic process	5	19
3851	GO:2000058	regulation of protein ubiquitination involved in ubiquitin-dependent protein catabolic process	5	9
3852	GO:2000121	regulation of removal of superoxide radicals	5	32
3853	GO:2000177	regulation of neural precursor cell proliferation	5	48
3854	GO:2000304	positive regulation of ceramide biosynthetic process	5	8
3855	GO:2000323	negative regulation of glucocorticoid receptor signaling pathway	5	14
3856	GO:2000327	positive regulation of ligand-dependent nuclear receptor transcription coactivator activity	5	38

3857	GO:2000379	positive regulation of reactive oxygen species metabolic process	5	22
3858	GO:2000505	regulation of energy homeostasis	5	48
3859	GO:2000543	positive regulation of gastrulation	5	8
3860	GO:2000651	positive regulation of sodium ion transmembrane transporter activity	5	12
3861	GO:2001257	regulation of cation channel activity	5	20
3862	GO:0000025	maltose catabolic process	4	21
3863	GO:0000375	RNA splicing, via transesterification reactions	4	11
3864	GO:0000455	enzyme-directed rRNA pseudouridine synthesis	4	29
3865	GO:0000460	maturation of 5.8S rRNA	4	17
3866	GO:0000727	double-strand break repair via break-induced replication	4	37
3867	GO:0000769	syncytium formation by mitosis without cytokinesis	4	28
3868	GO:0000913	preprophase band assembly	4	19
3869	GO:0000915	cytokinesis, actomyosin contractile ring assembly	4	30
3870	GO:0000921	septin ring assembly	4	4
3871	GO:0000972	transcription-dependent tethering of RNA polymerase II gene DNA at nuclear periphery	4	20
3872	GO:0001508	regulation of action potential	4	16
3873	GO:0001817	regulation of cytokine production	4	16
3874	GO:0001827	inner cell mass cell fate commitment	4	20
3875	GO:0001828	inner cell mass cellular morphogenesis	4	20
3876	GO:0001829	trophectodermal cell differentiation	4	21
3877	GO:0001831	trophectodermal cellular morphogenesis	4	13
3878	GO:0001927	exocyst assembly	4	15
3879	GO:0001951	intestinal D-glucose absorption	4	21
3880	GO:0002072	optic cup morphogenesis involved in camera-type eye development	4	7
3881	GO:0002084	protein depalmitoylation	4	7
3882	GO:0002086	diaphragm contraction	4	6
3883	GO:0002168	instar larval development	4	23
3884	GO:0002227	innate immune response in mucosa	4	4
3885	GO:0002758	innate immune response-activating signal transduction	4	9
3886	GO:0002862	negative regulation of inflammatory response to antigenic stimulus	4	5
3887	GO:0003014	renal system process	4	19
3888	GO:0003222	ventricular trabecula myocardium morphogenesis	4	21
3889	GO:0003226	right ventricular compact myocardium morphogenesis	4	21
3890	GO:0005993	trehalose catabolic process	4	48
3891	GO:0005996	monosaccharide metabolic process	4	18
3892	GO:0006015	5-phosphoribose 1-diphosphate biosynthetic process	4	33
3893	GO:0006021	inositol biosynthetic process	4	12
3894	GO:0006112	energy reserve metabolic process	4	53
3895	GO:0006148	inosine catabolic process	4	8
3896	GO:0006168	adenine salvage	4	21
3897	GO:0006172	ADP biosynthetic process	4	45
3898	GO:0006188	IMP biosynthetic process	4	24
3899	GO:0006231	dTMP biosynthetic process	4	21
3900	GO:0006271	DNA strand elongation involved in DNA replication	4	24
3901	GO:0006276	plasmid maintenance	4	41
3902	GO:0006421	asparaginyl-tRNA aminoacylation	4	14
3903	GO:0006427	histidyl-tRNA aminoacylation	4	55
3904	GO:0006428	isoleucyl-tRNA aminoacylation	4	40
3905	GO:0006437	tyrosyl-tRNA aminoacylation	4	20
3906	GO:0006476	protein deacetylation	4	18
3907	GO:0006620	posttranslational protein targeting to membrane	4	6
3908	GO:0006639	acylglycerol metabolic process	4	25
3909	GO:0006658	phosphatidylserine metabolic process	4	32
3910	GO:0006706	steroid catabolic process	4	5
3911	GO:0006741	NADP biosynthetic process	4	24
3912	GO:0006772	thiamine metabolic process	4	16

3913	GO:0006776	vitamin A metabolic process	4	7
3914	GO:0006799	polyphosphate biosynthetic process	4	24
3915	GO:0006801	superoxide metabolic process	4	18
3916	GO:0007028	cytoplasm organization	4	22
3917	GO:0007056	spindle assembly involved in female meiosis	4	4
3918	GO:0007070	negative regulation of transcription from RNA polymerase II promoter during mitosis	4	32
3919	GO:0007098	centrosome cycle	4	9
3920	GO:0007191	adenylate cyclase-activating dopamine receptor signaling pathway	4	14
3921	GO:0007274	neuromuscular synaptic transmission	4	22
3922	GO:0007494	midgut development	4	26
3923	GO:0007512	adult heart development	4	21
3924	GO:0007548	sex differentiation	4	21
3925	GO:0007600	sensory perception	4	24
3926	GO:0008334	histone mRNA metabolic process	4	6
3927	GO:0008594	photoreceptor cell morphogenesis	4	32
3928	GO:0008608	attachment of spindle microtubules to kinetochore	4	10
3929	GO:0009150	purine ribonucleotide metabolic process	4	11
3930	GO:0009168	purine ribonucleoside monophosphate biosynthetic process	4	9
3931	GO:0009173	pyrimidine ribonucleoside monophosphate metabolic process	4	50
3932	GO:0009234	menaquinone biosynthetic process	4	36
3933	GO:0009235	cobalamin metabolic process	4	43
3934	GO:0009242	colanic acid biosynthetic process	4	20
3935	GO:0009263	deoxyribonucleotide biosynthetic process	4	20
3936	GO:0009312	oligosaccharide biosynthetic process	4	26
3937	GO:0009404	toxin metabolic process	4	13
3938	GO:0009415	response to water	4	29
3939	GO:0009438	methylglyoxal metabolic process	4	15
3940	GO:0009859	pollen hydration	4	72
3941	GO:0009866	induced systemic resistance, ethylene mediated signaling pathway	4	30
3942	GO:0009949	polarity specification of anterior/posterior axis	4	16
3943	GO:0009951	polarity specification of dorsal/ventral axis	4	16
3944	GO:0010136	ureide catabolic process	4	25
3945	GO:0010157	response to chlorate	4	20
3946	GO:0010269	response to selenium ion	4	32
3947	GO:0010322	regulation of isopentenyl diphosphate biosynthetic process, mevalonate-independent pathway	4	15
3948	GO:0010410	hemicellulose metabolic process	4	20
3949	GO:0010460	positive regulation of heart rate	4	19
3950	GO:0010556	regulation of macromolecule biosynthetic process	4	22
3951	GO:0010591	regulation of lamellipodium assembly	4	26
3952	GO:0010647	positive regulation of cell communication	4	19
3953	GO:0010701	positive regulation of norepinephrine secretion	4	21
3954	GO:0010751	negative regulation of nitric oxide mediated signal transduction	4	21
3955	GO:0010765	positive regulation of sodium ion transport	4	6
3956	GO:0010828	positive regulation of glucose transport	4	45
3957	GO:0010829	negative regulation of glucose transport	4	25
3958	GO:0010833	telomere maintenance via telomere lengthening	4	16
3959	GO:0010862	positive regulation of pathway-restricted SMAD protein phosphorylation	4	41
3960	GO:0010875	positive regulation of cholesterol efflux	4	65
3961	GO:0010876	lipid localization	4	27
3962	GO:0010972	negative regulation of G2/M transition of mitotic cell cycle	4	10
3963	GO:0010992	ubiquitin homeostasis	4	9
3964	GO:0014067	negative regulation of phosphatidylinositol 3-kinase cascade	4	51

3965	GO:0015670	carbon dioxide transport	4	17
3966	GO:0015671	oxygen transport	4	9
3967	GO:0015689	molybdate ion transport	4	5
3968	GO:0015696	ammonium transport	4	20
3969	GO:0015723	bilirubin transport	4	18
3970	GO:0015744	succinate transport	4	8
3971	GO:0015748	organophosphate ester transport	4	8
3972	GO:0015759	beta-glucoside transport	4	21
3973	GO:0015801	aromatic amino acid transport	4	10
3974	GO:0015817	histidine transport	4	12
3975	GO:0015853	adenine transport	4	10
3976	GO:0015966	diadenosine tetraphosphate biosynthetic process	4	18
3977	GO:0016048	detection of temperature stimulus	4	8
3978	GO:0016120	carotene biosynthetic process	4	41
3979	GO:0016183	synaptic vesicle coating	4	45
3980	GO:0016203	muscle attachment	4	15
3981	GO:0016321	female meiosis chromosome segregation	4	32
3982	GO:0016358	dendrite development	4	8
3983	GO:0016446	somatic hypermutation of immunoglobulin genes	4	57
3984	GO:0016482	cytoplasmic transport	4	23
3985	GO:0016576	histone dephosphorylation	4	21
3986	GO:0017004	cytochrome complex assembly	4	40
3987	GO:0017038	protein import	4	23
3988	GO:0017158	regulation of calcium ion-dependent exocytosis	4	10
3989	GO:0018008	N-terminal peptidyl-glycine N-myristoylation	4	15
3990	GO:0018076	N-terminal peptidyl-lysine acetylation	4	43
3991	GO:0018125	peptidyl-cysteine methylation	4	10
3992	GO:0018197	peptidyl-aspartic acid modification	4	22
3993	GO:0018208	peptidyl-proline modification	4	24
3994	GO:0018345	protein palmitoylation	4	25
3995	GO:0019294	keto-3-deoxy-D-manno-octulosonic acid biosynthetic process	4	16
3996	GO:0019343	cysteine biosynthetic process via cystathionine	4	19
3997	GO:0019370	leukotriene biosynthetic process	4	15
3998	GO:0019428	allantoin biosynthetic process	4	15
3999	GO:0019447	D-cysteine catabolic process	4	17
4000	GO:0019499	cyanide metabolic process	4	22
4001	GO:0019510	S-adenosylhomocysteine catabolic process	4	5
4002	GO:0019544	arginine catabolic process to glutamate	4	17
4003	GO:0019632	shikimate metabolic process	4	24
4004	GO:0019731	antibacterial humoral response	4	4
4005	GO:0019885	antigen processing and presentation of endogenous peptide antigen via MHC class I	4	16
4006	GO:0021537	telencephalon development	4	27
4007	GO:0021553	olfactory nerve development	4	21
4008	GO:0021591	ventricular system development	4	20
4009	GO:0021707	cerebellar granule cell differentiation	4	18
4010	GO:0021955	central nervous system neuron axonogenesis	4	36
4011	GO:0022618	ribonucleoprotein complex assembly	4	13
4012	GO:0030041	actin filament polymerization	4	22
4013	GO:0030099	myeloid cell differentiation	4	24
4014	GO:0030104	water homeostasis	4	9
4015	GO:0030199	collagen fibril organization	4	14
4016	GO:0030282	bone mineralization	4	16
4017	GO:0030418	nicotianamine biosynthetic process	4	4
4018	GO:0030423	targeting of mRNA for destruction involved in RNA interference	4	13
4019	GO:0030490	maturation of SSU-rRNA	4	6
4020	GO:0030521	androgen receptor signaling pathway	4	23
4021	GO:0030581	symbiont intracellular protein transport in host	4	10

4022	GO:0030703	eggshell formation	4	7
4023	GO:0031069	hair follicle morphogenesis	4	14
4024	GO:0031111	negative regulation of microtubule polymerization or depolymerization	4	27
4025	GO:0031125	rRNA 3'-end processing	4	17
4026	GO:0031247	actin rod assembly	4	14
4027	GO:0031293	membrane protein intracellular domain proteolysis	4	25
4028	GO:0031344	regulation of cell projection organization	4	15
4029	GO:0031448	positive regulation of fast-twitch skeletal muscle fiber contraction	4	19
4030	GO:0031585	regulation of inositol 1,4,5-trisphosphate-sensitive calcium-release channel activity	4	19
4031	GO:0031638	zymogen activation	4	11
4032	GO:0031639	plasminogen activation	4	22
4033	GO:0031663	lipopolysaccharide-mediated signaling pathway	4	13
4034	GO:0031860	telomeric 3' overhang formation	4	33
4035	GO:0031915	positive regulation of synaptic plasticity	4	5
4036	GO:0031936	negative regulation of chromatin silencing	4	27
4037	GO:0032007	negative regulation of TOR signaling cascade	4	26
4038	GO:0032099	negative regulation of appetite	4	5
4039	GO:0032230	positive regulation of synaptic transmission, GABAergic	4	22
4040	GO:0032288	myelin assembly	4	11
4041	GO:0032298	positive regulation of DNA-dependent DNA replication initiation	4	16
4042	GO:0032367	intracellular cholesterol transport	4	24
4043	GO:0032392	DNA geometric change	4	34
4044	GO:0032418	lysosome localization	4	16
4045	GO:0032447	protein urmylation	4	37
4046	GO:0032463	negative regulation of protein homooligomerization	4	56
4047	GO:0032466	negative regulation of cytokinesis	4	10
4048	GO:0032469	endoplasmic reticulum calcium ion homeostasis	4	12
4049	GO:0032470	elevation of endoplasmic reticulum calcium ion concentration	4	19
4050	GO:0032471	reduction of endoplasmic reticulum calcium ion concentration	4	19
4051	GO:0032483	regulation of Rab protein signal transduction	4	14
4052	GO:0032529	follicle cell microvillus organization	4	39
4053	GO:0032532	regulation of microvillus length	4	21
4054	GO:0032623	interleukin-2 production	4	12
4055	GO:0032872	regulation of stress-activated MAPK cascade	4	25
4056	GO:0032888	regulation of mitotic spindle elongation	4	10
4057	GO:0032933	SREBP signaling pathway	4	32
4058	GO:0032953	regulation of (1->3)-beta-D-glucan biosynthetic process	4	20
4059	GO:0032962	positive regulation of inositol trisphosphate biosynthetic process	4	21
4060	GO:0033075	isoquinoline alkaloid biosynthetic process	4	6
4061	GO:0033132	negative regulation of glucokinase activity	4	8
4062	GO:0033146	regulation of intracellular estrogen receptor signaling pathway	4	35
4063	GO:0033148	positive regulation of intracellular estrogen receptor signaling pathway	4	24
4064	GO:0033314	mitotic cell cycle DNA replication checkpoint	4	24
4065	GO:0033384	geranyl diphosphate biosynthetic process	4	6
4066	GO:0033468	CMP-keto-3-deoxy-D-manno-octulosonic acid biosynthetic process	4	10
4067	GO:0033499	galactose catabolic process via UDP-galactose	4	20
4068	GO:0033611	oxalate catabolic process	4	13
4069	GO:0034049	positive regulation of protein phosphatase type 2A activity	4	19
4070	GO:0034126	positive regulation of MyD88-dependent toll-like receptor signaling pathway	4	33
4071	GO:0034134	toll-like receptor 2 signaling pathway	4	13

4072	GO:0034140	negative regulation of toll-like receptor 3 signaling pathway	4	24
4073	GO:0034329	cell junction assembly	4	20
4074	GO:0034333	adherens junction assembly	4	31
4075	GO:0034340	response to type I interferon	4	7
4076	GO:0034341	response to interferon-gamma	4	14
4077	GO:0034380	high-density lipoprotein particle assembly	4	65
4078	GO:0034454	microtubule anchoring at centrosome	4	16
4079	GO:0034463	90S preribosome assembly	4	5
4080	GO:0035024	negative regulation of Rho protein signal transduction	4	13
4081	GO:0035096	larval midgut cell programmed cell death	4	17
4082	GO:0035138	pectoral fin morphogenesis	4	20
4083	GO:0035176	social behavior	4	30
4084	GO:0035222	wing disc pattern formation	4	37
4085	GO:0035293	chitin-based larval cuticle pattern formation	4	39
4086	GO:0035352	NAD transmembrane transport	4	42
4087	GO:0035437	maintenance of protein localization in endoplasmic reticulum	4	30
4088	GO:0035441	cell migration involved in vasculogenesis	4	58
4089	GO:0035494	SNARE complex disassembly	4	19
4090	GO:0035616	histone H2B conserved C-terminal lysine deubiquitination	4	29
4091	GO:0035617	stress granule disassembly	4	22
4092	GO:0035672	oligopeptide transmembrane transport	4	35
4093	GO:0035864	response to potassium ion	4	37
4094	GO:0035909	aorta morphogenesis	4	21
4095	GO:0035967	cellular response to topologically incorrect protein	4	20
4096	GO:0035994	response to muscle stretch	4	23
4097	GO:0036079	purine nucleotide-sugar transport	4	21
4098	GO:0036123	histone H3-K9 dimethylation	4	33
4099	GO:0038027	apolipoprotein A-I-mediated signaling pathway	4	65
4100	GO:0038066	p38MAPK cascade	4	7
4101	GO:0039019	pronephric nephron development	4	13
4102	GO:0040002	collagen and cuticulin-based cuticle development	4	10
4103	GO:0040015	negative regulation of multicellular organism growth	4	23
4104	GO:0042093	T-helper cell differentiation	4	14
4105	GO:0042133	neurotransmitter metabolic process	4	16
4106	GO:0042246	tissue regeneration	4	26
4107	GO:0042318	penicillin biosynthetic process	4	22
4108	GO:0042325	regulation of phosphorylation	4	19
4109	GO:0042326	negative regulation of phosphorylation	4	16
4110	GO:0042340	keratan sulfate catabolic process	4	30
4111	GO:0042414	epinephrine metabolic process	4	14
4112	GO:0042416	dopamine biosynthetic process	4	15
4113	GO:0042417	dopamine metabolic process	4	14
4114	GO:0042426	choline catabolic process	4	7
4115	GO:0042438	melanin biosynthetic process	4	19
4116	GO:0042476	odontogenesis	4	19
4117	GO:0042817	pyridoxal metabolic process	4	31
4118	GO:0042838	D-glucarate catabolic process	4	8
4119	GO:0042992	negative regulation of transcription factor import into nucleus	4	19
4120	GO:0042997	negative regulation of Golgi to plasma membrane protein transport	4	28
4121	GO:0043051	regulation of pharyngeal pumping	4	5
4122	GO:0043320	natural killer cell degranulation	4	4
4123	GO:0043393	regulation of protein binding	4	18
4124	GO:0043542	endothelial cell migration	4	17
4125	GO:0043570	maintenance of DNA repeat elements	4	79
4126	GO:0043584	nose development	4	21
4127	GO:0043666	regulation of phosphoprotein phosphatase activity	4	11

4128	GO:0043983	histone H4-K12 acetylation	4	36
4129	GO:0043987	histone H3-S10 phosphorylation	4	17
4130	GO:0043988	histone H3-S28 phosphorylation	4	17
4131	GO:0044005	induction by symbiont in host of tumor, nodule, or growth	4	32
4132	GO:0044092	negative regulation of molecular function	4	19
4133	GO:0044148	positive regulation of growth of symbiont involved in interaction with host	4	17
4134	GO:0044206	UMP salvage	4	48
4135	GO:0044264	cellular polysaccharide metabolic process	4	30
4136	GO:0044346	fibroblast apoptotic process	4	13
4137	GO:0044376	RNA polymerase II complex localization to nucleus	4	18
4138	GO:0044571	[2Fe-2S] cluster assembly	4	7
4139	GO:0045023	G0 to G1 transition	4	29
4140	GO:0045085	negative regulation of interleukin-2 biosynthetic process	4	7
4141	GO:0045190	isotype switching	4	46
4142	GO:0045337	farnesyl diphosphate biosynthetic process	4	6
4143	GO:0045345	positive regulation of MHC class I biosynthetic process	4	10
4144	GO:0045448	mitotic cell cycle, embryonic	4	18
4145	GO:0045467	R7 cell development	4	9
4146	GO:0045598	regulation of fat cell differentiation	4	28
4147	GO:0045652	regulation of megakaryocyte differentiation	4	12
4148	GO:0045759	negative regulation of action potential	4	14
4149	GO:0045763	negative regulation of cellular amino acid metabolic process	4	15
4150	GO:0045869	negative regulation of retroviral genome replication	4	25
4151	GO:0045876	positive regulation of sister chromatid cohesion	4	50
4152	GO:0045930	negative regulation of mitotic cell cycle	4	5
4153	GO:0045945	positive regulation of transcription from RNA polymerase III promoter	4	46
4154	GO:0045988	negative regulation of striated muscle contraction	4	19
4155	GO:0046128	purine ribonucleoside metabolic process	4	16
4156	GO:0046129	purine ribonucleoside biosynthetic process	4	6
4157	GO:0046135	pyrimidine nucleoside catabolic process	4	10
4158	GO:0046189	phenol-containing compound biosynthetic process	4	9
4159	GO:0046196	4-nitrophenol catabolic process	4	20
4160	GO:0046208	spermine catabolic process	4	19
4161	GO:0046326	positive regulation of glucose import	4	17
4162	GO:0046328	regulation of JNK cascade	4	6
4163	GO:0046340	diacylglycerol catabolic process	4	8
4164	GO:0046345	abscisic acid catabolic process	4	7
4165	GO:0046392	galactarate catabolic process	4	8
4166	GO:0046427	positive regulation of JAK-STAT cascade	4	42
4167	GO:0046602	regulation of mitotic centrosome separation	4	18
4168	GO:0046632	alpha-beta T cell differentiation	4	67
4169	GO:0046641	positive regulation of alpha-beta T cell proliferation	4	67
4170	GO:0046710	GDP metabolic process	4	40
4171	GO:0046741	spread of virus in host, tissue to tissue	4	8
4172	GO:0046826	negative regulation of protein export from nucleus	4	15
4173	GO:0046843	dorsal appendage formation	4	19
4174	GO:0046942	carboxylic acid transport	4	10
4175	GO:0048008	platelet-derived growth factor receptor signaling pathway	4	36
4176	GO:0048096	chromatin-mediated maintenance of transcription	4	14
4177	GO:0048167	regulation of synaptic plasticity	4	28
4178	GO:0048172	regulation of short-term neuronal synaptic plasticity	4	34
4179	GO:0048213	Golgi vesicle prefusion complex stabilization	4	10
4180	GO:0048251	elastic fiber assembly	4	14
4181	GO:0048264	determination of ventral identity	4	30
4182	GO:0048265	response to pain	4	16
4183	GO:0048308	organelle inheritance	4	36

4184	GO:0048332	mesoderm morphogenesis	4	58
4185	GO:0048478	replication fork protection	4	39
4186	GO:0048490	anterograde synaptic vesicle transport	4	30
4187	GO:0048514	blood vessel morphogenesis	4	24
4188	GO:0048534	hemopoietic or lymphoid organ development	4	25
4189	GO:0048569	post-embryonic organ development	4	22
4190	GO:0048571	long-day photoperiodism	4	50
4191	GO:0048640	negative regulation of developmental growth	4	8
4192	GO:0048655	tapetal layer morphogenesis	4	27
4193	GO:0048667	cell morphogenesis involved in neuron differentiation	4	26
4194	GO:0048732	gland development	4	14
4195	GO:0048752	semicircular canal morphogenesis	4	21
4196	GO:0048839	inner ear development	4	27
4197	GO:0048867	stem cell fate determination	4	17
4198	GO:0048873	homeostasis of number of cells within a tissue	4	9
4199	GO:0050482	arachidonic acid secretion	4	31
4200	GO:0050731	positive regulation of peptidyl-tyrosine phosphorylation	4	38
4201	GO:0050732	negative regulation of peptidyl-tyrosine phosphorylation	4	8
4202	GO:0050773	regulation of dendrite development	4	25
4203	GO:0050777	negative regulation of immune response	4	7
4204	GO:0050778	positive regulation of immune response	4	19
4205	GO:0050873	brown fat cell differentiation	4	29
4206	GO:0050920	regulation of chemotaxis	4	18
4207	GO:0051012	microtubule sliding	4	18
4208	GO:0051086	chaperone mediated protein folding independent of cofactor	4	13
4209	GO:0051135	positive regulation of NK T cell activation	4	10
4210	GO:0051205	protein insertion into membrane	4	9
4211	GO:0051220	cytoplasmic sequestering of protein	4	29
4212	GO:0051446	positive regulation of meiotic cell cycle	4	28
4213	GO:0051452	intracellular pH reduction	4	7
4214	GO:0051455	attachment of spindle microtubules to kinetochore involved in homologous chromosome segregation	4	30
4215	GO:0051482	elevation of cytosolic calcium ion concentration involved in phospholipase C-activating G-protein coupled signaling pathway	4	21
4216	GO:0051490	negative regulation of filopodium assembly	4	26
4217	GO:0051497	negative regulation of stress fiber assembly	4	25
4218	GO:0051511	negative regulation of unidimensional cell growth	4	6
4219	GO:0051513	regulation of monopolar cell growth	4	11
4220	GO:0051518	positive regulation of bipolar cell growth	4	37
4221	GO:0051542	elastin biosynthetic process	4	14
4222	GO:0051599	response to hydrostatic pressure	4	21
4223	GO:0051643	endoplasmic reticulum localization	4	19
4224	GO:0051648	vesicle localization	4	15
4225	GO:0051659	maintenance of mitochondrion location	4	19
4226	GO:0051762	sesquiterpene biosynthetic process	4	9
4227	GO:0051926	negative regulation of calcium ion transport	4	25
4228	GO:0051930	regulation of sensory perception of pain	4	23
4229	GO:0052548	regulation of endopeptidase activity	4	5
4230	GO:0052651	monoacylglycerol catabolic process	4	8
4231	GO:0060039	pericardium development	4	58
4232	GO:0060048	cardiac muscle contraction	4	6
4233	GO:0060078	regulation of postsynaptic membrane potential	4	9
4234	GO:0060123	regulation of growth hormone secretion	4	20
4235	GO:0060148	positive regulation of posttranscriptional gene silencing	4	32
4236	GO:0060291	long-term synaptic potentiation	4	39
4237	GO:0060389	pathway-restricted SMAD protein phosphorylation	4	29
4238	GO:0060439	trachea morphogenesis	4	36
4239	GO:0060542	regulation of strand invasion	4	4

4240	GO:0060613	fat pad development	4	20
4241	GO:0060669	embryonic placenta morphogenesis	4	58
4242	GO:0060710	chorio-allantoic fusion	4	12
4243	GO:0060729	intestinal epithelial structure maintenance	4	21
4244	GO:0060977	coronary vasculature morphogenesis	4	58
4245	GO:0061084	negative regulation of protein refolding	4	16
4246	GO:0061085	regulation of histone H3-K27 methylation	4	13
4247	GO:0061138	morphogenesis of a branching epithelium	4	14
4248	GO:0065002	intracellular protein transmembrane transport	4	28
4249	GO:0070050	neuron homeostasis	4	20
4250	GO:0070058	tRNA gene clustering	4	25
4251	GO:0070084	protein initiator methionine removal	4	8
4252	GO:0070244	negative regulation of thymocyte apoptotic process	4	67
4253	GO:0070266	necroptosis	4	26
4254	GO:0070481	nuclear-transcribed mRNA catabolic process, non-stop decay	4	5
4255	GO:0070550	rDNA condensation	4	25
4256	GO:0070585	protein localization in mitochondrion	4	10
4257	GO:0070649	formin-nucleated actin cable assembly	4	21
4258	GO:0070837	dehydroascorbic acid transport	4	22
4259	GO:0070843	misfolded protein transport	4	19
4260	GO:0070885	negative regulation of calcineurin-NFAT signaling cascade	4	21
4261	GO:0070972	protein localization in endoplasmic reticulum	4	24
4262	GO:0071027	nuclear RNA surveillance	4	26
4263	GO:0071048	nuclear retention of unspliced pre-mRNA at the site of transcription	4	11
4264	GO:0071050	snoRNA polyadenylation	4	33
4265	GO:0071103	DNA conformation change	4	30
4266	GO:0071248	cellular response to metal ion	4	35
4267	GO:0071318	cellular response to ATP	4	14
4268	GO:0071340	skeletal muscle acetylcholine-gated channel clustering	4	10
4269	GO:0071367	cellular response to brassinosteroid stimulus	4	27
4270	GO:0071466	cellular response to xenobiotic stimulus	4	21
4271	GO:0071469	cellular response to alkalinity	4	18
4272	GO:0071498	cellular response to fluid shear stress	4	34
4273	GO:0071548	response to dexamethasone stimulus	4	23
4274	GO:0071630	nucleus-associated proteasomal ubiquitin-dependent protein catabolic process	4	17
4275	GO:0071824	protein-DNA complex subunit organization	4	9
4276	GO:0071870	cellular response to catecholamine stimulus	4	14
4277	GO:0071872	cellular response to epinephrine stimulus	4	21
4278	GO:0071985	multivesicular body sorting pathway	4	13
4279	GO:0072592	oxygen metabolic process	4	13
4280	GO:0080034	host response to induction by symbiont of tumor, nodule or growth in host	4	10
4281	GO:0080058	protein deglutathionylation	4	12
4282	GO:0080111	DNA demethylation	4	40
4283	GO:0080119	ER body organization	4	25
4284	GO:0090076	relaxation of skeletal muscle	4	19
4285	GO:0090129	positive regulation of synapse maturation	4	5
4286	GO:0090141	positive regulation of mitochondrial fission	4	32
4287	GO:0090156	cellular sphingolipid homeostasis	4	26
4288	GO:0090394	negative regulation of excitatory postsynaptic membrane potential	4	21
4289	GO:0097031	mitochondrial respiratory chain complex I biogenesis	4	40
4290	GO:0097178	ruffle assembly	4	25
4291	GO:0097194	execution phase of apoptosis	4	26
4292	GO:0097198	histone H3-K36 trimethylation	4	58
4293	GO:0097248	maintenance of protein location in cell cortex of cell tip	4	37
4294	GO:1900082	negative regulation of arginine catabolic process	4	21

4295	GO:1900186	negative regulation of clathrin-mediated endocytosis	4	24
4296	GO:1900239	regulation of phenotypic switching	4	18
4297	GO:1900274	regulation of phospholipase C activity	4	21
4298	GO:1900384	regulation of flavonol biosynthetic process	4	5
4299	GO:1900743	positive regulation of filamentous growth of a population of unicellular organisms in response to pH	4	18
4300	GO:1901020	negative regulation of calcium ion transmembrane transporter activity	4	19
4301	GO:1901097	negative regulation of autophagic vacuole maturation	4	24
4302	GO:1901194	negative regulation of formation of translation preinitiation complex	4	8
4303	GO:2000052	positive regulation of non-canonical Wnt receptor signaling pathway	4	17
4304	GO:2000059	negative regulation of protein ubiquitination involved in ubiquitin-dependent protein catabolic process	4	5
4305	GO:2000100	regulation of establishment or maintenance of bipolar cell polarity regulating cell shape	4	37
4306	GO:2000124	regulation of endocannabinoid signaling pathway	4	23
4307	GO:2000145	regulation of cell motility	4	18
4308	GO:2000171	negative regulation of dendrite development	4	16
4309	GO:2000278	regulation of DNA biosynthetic process	4	27
4310	GO:2000311	regulation of alpha-amino-3-hydroxy-5-methyl-4-isoxazole propionate selective glutamate receptor activity	4	9
4311	GO:2000394	positive regulation of lamellipodium morphogenesis	4	48
4312	GO:2000481	positive regulation of cAMP-dependent protein kinase activity	4	21
4313	GO:2000643	positive regulation of early endosome to late endosome transport	4	15
4314	GO:2000648	positive regulation of stem cell proliferation	4	50
4315	GO:2000650	negative regulation of sodium ion transmembrane transporter activity	4	15
4316	GO:2000672	negative regulation of motor neuron apoptotic process	4	27
4317	GO:2000679	positive regulation of transcription regulatory region DNA binding	4	47
4318	GO:2000774	positive regulation of cellular senescence	4	17
4319	GO:2000811	negative regulation of anoikis	4	47
4320	GO:2000825	positive regulation of androgen receptor activity	4	13
4321	GO:2001006	regulation of cellulose biosynthetic process	4	48
4322	GO:2001137	positive regulation of endocytic recycling	4	23
4323	GO:2001159	regulation of CVT pathway	4	30
4324	GO:0000023	maltose metabolic process	3	3
4325	GO:0000097	sulfur amino acid biosynthetic process	3	22
4326	GO:0000256	allantoin catabolic process	3	22
4327	GO:0000282	cellular bud site selection	3	11
4328	GO:0000494	box C/D snoRNA 3'-end processing	3	6
4329	GO:0000495	box H/ACA snoRNA 3'-end processing	3	13
4330	GO:0000709	meiotic joint molecule formation	3	11
4331	GO:0000711	meiotic DNA repair synthesis	3	16
4332	GO:0000737	DNA catabolic process, endonucleolytic	3	13
4333	GO:0000746	conjugation	3	22
4334	GO:0000903	regulation of cell shape during vegetative growth phase	3	11
4335	GO:0000914	phragmoplast assembly	3	11
4336	GO:0000958	mitochondrial mRNA catabolic process	3	27
4337	GO:0000962	positive regulation of mitochondrial RNA catabolic process	3	27
4338	GO:0001193	maintenance of transcriptional fidelity during DNA-dependent transcription elongation from RNA polymerase II promoter	3	4
4339	GO:0001208	histone H2A-H2B dimer displacement	3	11
4340	GO:0001308	negative regulation of chromatin silencing involved in replicative cell aging	3	10
4341	GO:0001315	age-dependent response to reactive oxygen species	3	6

4342	GO:0001504	neurotransmitter uptake	3	15
4343	GO:0001682	tRNA 5'-leader removal	3	28
4344	GO:0001751	compound eye photoreceptor cell differentiation	3	12
4345	GO:0001836	release of cytochrome c from mitochondria	3	10
4346	GO:0001894	tissue homeostasis	3	23
4347	GO:0001921	positive regulation of receptor recycling	3	12
4348	GO:0001937	negative regulation of endothelial cell proliferation	3	9
4349	GO:0001942	hair follicle development	3	10
4350	GO:0002003	angiotensin maturation	3	22
4351	GO:0002026	regulation of the force of heart contraction	3	14
4352	GO:0002076	osteoblast development	3	12
4353	GO:0002138	retinoic acid biosynthetic process	3	3
4354	GO:0002143	tRNA wobble position uridine thiolation	3	38
4355	GO:0002176	male germ cell proliferation	3	8
4356	GO:0002184	cytoplasmic translational termination	3	16
4357	GO:0002252	immune effector process	3	3
4358	GO:0002286	T cell activation involved in immune response	3	11
4359	GO:0002456	T cell mediated immunity	3	7
4360	GO:0002495	antigen processing and presentation of peptide antigen via MHC class II	3	9
4361	GO:0002683	negative regulation of immune system process	3	11
4362	GO:0003032	detection of oxygen	3	13
4363	GO:0003205	cardiac chamber development	3	23
4364	GO:0003283	atrial septum development	3	7
4365	GO:0003380	establishment or maintenance of cytoskeleton polarity involved in gastrulation	3	9
4366	GO:0003400	regulation of COPII vesicle coating	3	5
4367	GO:0005976	polysaccharide metabolic process	3	13
4368	GO:0005997	xylulose metabolic process	3	3
4369	GO:0006001	fructose catabolic process	3	15
4370	GO:0006005	L-fucose biosynthetic process	3	3
4371	GO:0006030	chitin metabolic process	3	8
4372	GO:0006044	N-acetylglucosamine metabolic process	3	5
4373	GO:0006045	N-acetylglucosamine biosynthetic process	3	21
4374	GO:0006062	sorbitol catabolic process	3	4
4375	GO:0006081	cellular aldehyde metabolic process	3	12
4376	GO:0006114	glycerol biosynthetic process	3	5
4377	GO:0006127	glycerophosphate shuttle	3	4
4378	GO:0006163	purine nucleotide metabolic process	3	18
4379	GO:0006196	AMP catabolic process	3	22
4380	GO:0006235	dTTP biosynthetic process	3	28
4381	GO:0006238	CMP salvage	3	37
4382	GO:0006264	mitochondrial DNA replication	3	14
4383	GO:0006390	transcription from mitochondrial promoter	3	15
4384	GO:0006391	transcription initiation from mitochondrial promoter	3	47
4385	GO:0006399	tRNA metabolic process	3	47
4386	GO:0006419	alanyl-tRNA aminoacylation	3	25
4387	GO:0006425	glutaminyl-tRNA aminoacylation	3	13
4388	GO:0006426	glycyl-tRNA aminoacylation	3	38
4389	GO:0006435	threonyl-tRNA aminoacylation	3	18
4390	GO:0006436	tryptophanyl-tRNA aminoacylation	3	19
4391	GO:0006438	valyl-tRNA aminoacylation	3	31
4392	GO:0006533	aspartate catabolic process	3	16
4393	GO:0006557	S-adenosylmethioninamine biosynthetic process	3	3
4394	GO:0006582	melanin metabolic process	3	13
4395	GO:0006595	polyamine metabolic process	3	14
4396	GO:0006617	SRP-dependent cotranslational protein targeting to membrane, signal sequence recognition	3	12
4397	GO:0006649	phospholipid transfer to membrane	3	52
4398	GO:0006659	phosphatidylserine biosynthetic process	3	51

4399	GO:0006667	sphinganine metabolic process	3	10
4400	GO:0006680	glucosylceramide catabolic process	3	13
4401	GO:0006702	androgen biosynthetic process	3	3
4402	GO:0006792	regulation of sulfur utilization	3	15
4403	GO:0006827	high-affinity iron ion transport	3	5
4404	GO:0006844	acyl carnitine transport	3	13
4405	GO:0006876	cellular cadmium ion homeostasis	3	14
4406	GO:0006883	cellular sodium ion homeostasis	3	18
4407	GO:0006903	vesicle targeting	3	13
4408	GO:0006910	phagocytosis, recognition	3	11
4409	GO:0006924	activation-induced cell death of T cells	3	8
4410	GO:0006963	positive regulation of antibacterial peptide biosynthetic process	3	45
4411	GO:0006987	activation of signaling protein activity involved in unfolded protein response	3	6
4412	GO:0006990	positive regulation of transcription from RNA polymerase II promoter involved in unfolded protein response	3	35
4413	GO:0007023	post-chaperonin tubulin folding pathway	3	16
4414	GO:0007089	traversing start control point of mitotic cell cycle	3	40
4415	GO:0007097	nuclear migration	3	10
4416	GO:0007133	meiotic anaphase I	3	25
4417	GO:0007144	female meiosis I	3	11
4418	GO:0007175	negative regulation of epidermal growth factor-activated receptor activity	3	24
4419	GO:0007213	G-protein coupled acetylcholine receptor signaling pathway	3	13
4420	GO:0007221	positive regulation of transcription of Notch receptor target	3	15
4421	GO:0007256	activation of JNKK activity	3	7
4422	GO:0007284	spermatogonial cell division	3	19
4423	GO:0007310	oocyte dorsal/ventral axis specification	3	4
4424	GO:0007314	oocyte anterior/posterior axis specification	3	4
4425	GO:0007430	terminal branching, open tracheal system	3	21
4426	GO:0007455	eye-antennal disc morphogenesis	3	13
4427	GO:0007465	R7 cell fate commitment	3	17
4428	GO:0007474	imaginal disc-derived wing vein specification	3	9
4429	GO:0007482	haltere development	3	13
4430	GO:0007485	imaginal disc-derived male genitalia development	3	36
4431	GO:0007528	neuromuscular junction development	3	19
4432	GO:0007566	embryo implantation	3	18
4433	GO:0007610	behavior	3	35
4434	GO:0007625	grooming behavior	3	31
4435	GO:0007629	flight behavior	3	31
4436	GO:0007630	jump response	3	31
4437	GO:0008049	male courtship behavior	3	36
4438	GO:0008053	mitochondrial fusion	3	19
4439	GO:0008069	dorsal/ventral axis specification, ovarian follicular epithelium	3	45
4440	GO:0008101	decapentaplegic signaling pathway	3	6
4441	GO:0008355	olfactory learning	3	14
4442	GO:0008535	respiratory chain complex IV assembly	3	27
4443	GO:0008589	regulation of smoothened signaling pathway	3	35
4444	GO:0008630	DNA damage response, signal transduction resulting in induction of apoptosis	3	55
4445	GO:0009083	branched chain family amino acid catabolic process	3	10
4446	GO:0009092	homoserine metabolic process	3	8
4447	GO:0009156	ribonucleoside monophosphate biosynthetic process	3	18
4448	GO:0009202	deoxyribonucleoside triphosphate biosynthetic process	3	20
4449	GO:0009250	glucan biosynthetic process	3	18
4450	GO:0009308	amine metabolic process	3	7

4451	GO:0009396	folic acid-containing compound biosynthetic process	3	21
4452	GO:0009403	toxin biosynthetic process	3	28
4453	GO:0009443	pyridoxal 5'-phosphate salvage	3	20
4454	GO:0009663	plasmodesma organization	3	21
4455	GO:0009756	carbohydrate mediated signaling	3	11
4456	GO:0009769	photosynthesis, light harvesting in photosystem II	3	8
4457	GO:0009855	determination of bilateral symmetry	3	9
4458	GO:0009947	centrolateral axis specification	3	6
4459	GO:0009948	anterior/posterior axis specification	3	12
4460	GO:0010070	zygote asymmetric cell division	3	16
4461	GO:0010133	proline catabolic process to glutamate	3	17
4462	GO:0010219	regulation of vernalization response	3	10
4463	GO:0010254	nectary development	3	13
4464	GO:0010375	stomatal complex patterning	3	6
4465	GO:0010381	attachment of peroxisome to chloroplast	3	28
4466	GO:0010422	regulation of brassinosteroid biosynthetic process	3	12
4467	GO:0010434	bract formation	3	13
4468	GO:0010448	vegetative meristem growth	3	25
4469	GO:0010450	inflorescence meristem growth	3	4
4470	GO:0010470	regulation of gastrulation	3	9
4471	GO:0010565	regulation of cellular ketone metabolic process	3	12
4472	GO:0010595	positive regulation of endothelial cell migration	3	19
4473	GO:0010602	regulation of 1-aminocyclopropane-1-carboxylate metabolic process	3	11
4474	GO:0010621	negative regulation of transcription by transcription factor localization	3	10
4475	GO:0010654	apical cell fate commitment	3	6
4476	GO:0010688	negative regulation of ribosomal protein gene transcription from RNA polymerase II promoter	3	4
4477	GO:0010727	negative regulation of hydrogen peroxide metabolic process	3	15
4478	GO:0010734	negative regulation of protein glutathionylation	3	18
4479	GO:0010737	protein kinase A signaling cascade	3	10
4480	GO:0010745	negative regulation of macrophage derived foam cell differentiation	3	43
4481	GO:0010763	positive regulation of fibroblast migration	3	12
4482	GO:0010768	negative regulation of transcription from RNA polymerase II promoter in response to UV-induced DNA damage	3	10
4483	GO:0010811	positive regulation of cell-substrate adhesion	3	9
4484	GO:0010813	neuropeptide catabolic process	3	15
4485	GO:0010815	bradykinin catabolic process	3	11
4486	GO:0010849	regulation of proton-transporting ATPase activity, rotational mechanism	3	12
4487	GO:0010865	stipule development	3	6
4488	GO:0010886	positive regulation of cholesterol storage	3	13
4489	GO:0010929	positive regulation of auxin mediated signaling pathway	3	40
4490	GO:0010955	negative regulation of protein processing	3	21
4491	GO:0010960	magnesium ion homeostasis	3	7
4492	GO:0010975	regulation of neuron projection development	3	6
4493	GO:0010978	gene silencing involved in chronological cell aging	3	10
4494	GO:0010995	free ubiquitin chain depolymerization	3	52
4495	GO:0014075	response to amine stimulus	3	9
4496	GO:0014894	response to denervation involved in regulation of muscle adaptation	3	12
4497	GO:0014898	cardiac muscle hypertrophy in response to stress	3	12
4498	GO:0015675	nickel cation transport	3	13
4499	GO:0015676	vanadium ion transport	3	13
4500	GO:0015707	nitrite transport	3	6
4501	GO:0015739	sialic acid transport	3	34
4502	GO:0015755	fructose transport	3	17
4503	GO:0015767	lactose transport	3	12

4504	GO:0015781	pyrimidine nucleotide-sugar transport	3	6
4505	GO:0015782	CMP-N-acetylneuraminate transport	3	29
4506	GO:0015794	glycerol-3-phosphate transport	3	6
4507	GO:0015816	glycine transport	3	4
4508	GO:0015842	synaptic vesicle amine transport	3	12
4509	GO:0015844	monoamine transport	3	12
4510	GO:0015919	peroxisomal membrane transport	3	52
4511	GO:0016093	polyprenol metabolic process	3	8
4512	GO:0016094	polyprenol biosynthetic process	3	6
4513	GO:0016101	diterpenoid metabolic process	3	4
4514	GO:0016122	xanthophyll metabolic process	3	16
4515	GO:0016242	negative regulation of macroautophagy	3	13
4516	GO:0016255	attachment of GPI anchor to protein	3	38
4517	GO:0016339	calcium-dependent cell-cell adhesion	3	16
4518	GO:0016344	meiotic chromosome movement towards spindle pole	3	12
4519	GO:0016348	imaginal disc-derived leg joint morphogenesis	3	36
4520	GO:0016601	Rac protein signal transduction	3	13
4521	GO:0016926	protein desumoylation	3	46
4522	GO:0018283	iron incorporation into metallo-sulfur cluster	3	10
4523	GO:0018315	molybdenum incorporation into molybdenum-molybdopterin complex	3	21
4524	GO:0018879	biphenyl metabolic process	3	3
4525	GO:0018894	dibenzo-p-dioxin metabolic process	3	3
4526	GO:0018963	phthalate metabolic process	3	3
4527	GO:0019062	viral attachment to host cell	3	48
4528	GO:0019089	transmission of virus	3	10
4529	GO:0019249	lactate biosynthetic process	3	11
4530	GO:0019265	glycine biosynthetic process, by transamination of glyoxylate	3	8
4531	GO:0019279	L-methionine biosynthetic process from L-homoserine via cystathionine	3	16
4532	GO:0019310	inositol catabolic process	3	11
4533	GO:0019322	pentose biosynthetic process	3	18
4534	GO:0019336	phenol-containing compound catabolic process	3	7
4535	GO:0019348	dolichol metabolic process	3	9
4536	GO:0019358	nicotinate nucleotide salvage	3	27
4537	GO:0019388	galactose catabolic process	3	13
4538	GO:0019424	sulfide oxidation, using siroheme sulfite reductase	3	22
4539	GO:0019470	4-hydroxyproline catabolic process	3	16
4540	GO:0019500	cyanide catabolic process	3	5
4541	GO:0019550	glutamate catabolic process to aspartate	3	16
4542	GO:0019551	glutamate catabolic process to 2-oxoglutarate	3	16
4543	GO:0019679	propionate metabolic process, methylcitrate cycle	3	15
4544	GO:0019685	photosynthesis, dark reaction	3	13
4545	GO:0019697	L-xylitol catabolic process to xylulose 5-phosphate	3	3
4546	GO:0021542	dentate gyrus development	3	18
4547	GO:0021675	nerve development	3	19
4548	GO:0021764	amygdala development	3	14
4549	GO:0021768	nucleus accumbens development	3	5
4550	GO:0021799	cerebral cortex radially oriented cell migration	3	6
4551	GO:0021942	radial glia guided migration of Purkinje cell	3	6
4552	GO:0021953	central nervous system neuron differentiation	3	6
4553	GO:0021983	pituitary gland development	3	4
4554	GO:0022605	oogenesis stage	3	8
4555	GO:0022613	ribonucleoprotein complex biogenesis	3	9
4556	GO:0023019	signal transduction involved in regulation of gene expression	3	45
4557	GO:0023056	positive regulation of signaling	3	16
4558	GO:0023061	signal release	3	4
4559	GO:0030207	chondroitin sulfate catabolic process	3	14
4560	GO:0030210	heparin biosynthetic process	3	6

4561	GO:0030214	hyaluronan catabolic process	3	14
4562	GO:0030224	monocyte differentiation	3	7
4563	GO:0030225	macrophage differentiation	3	45
4564	GO:0030302	deoxynucleotide transport	3	13
4565	GO:0030449	regulation of complement activation	3	4
4566	GO:0030472	mitotic spindle organization in nucleus	3	12
4567	GO:0030476	ascospore wall assembly	3	11
4568	GO:0030520	intracellular estrogen receptor signaling pathway	3	37
4569	GO:0030576	Cajal body organization	3	45
4570	GO:0030593	neutrophil chemotaxis	3	8
4571	GO:0030595	leukocyte chemotaxis	3	12
4572	GO:0030708	germarium-derived female germ-line cyst encapsulation	3	10
4573	GO:0030727	germarium-derived female germ-line cyst formation	3	13
4574	GO:0030850	prostate gland development	3	8
4575	GO:0030903	notochord development	3	8
4576	GO:0031099	regeneration	3	9
4577	GO:0031456	glycine betaine biosynthetic process	3	18
4578	GO:0031503	protein complex localization	3	12
4579	GO:0031534	minus-end directed microtubule sliding	3	3
4580	GO:0031665	negative regulation of lipopolysaccharide-mediated signaling pathway	3	13
4581	GO:0031943	regulation of glucocorticoid metabolic process	3	17
4582	GO:0031990	mRNA export from nucleus in response to heat stress	3	3
4583	GO:0032024	positive regulation of insulin secretion	3	7
4584	GO:0032066	nucleolus to nucleoplasm transport	3	37
4585	GO:0032077	positive regulation of deoxyribonuclease activity	3	16
4586	GO:0032119	sequestering of zinc ion	3	35
4587	GO:0032148	activation of protein kinase B activity	3	19
4588	GO:0032200	telomere organization	3	4
4589	GO:0032210	regulation of telomere maintenance via telomerase	3	10
4590	GO:0032286	central nervous system myelin maintenance	3	19
4591	GO:0032354	response to follicle-stimulating hormone stimulus	3	3
4592	GO:0032364	oxygen homeostasis	3	34
4593	GO:0032365	intracellular lipid transport	3	19
4594	GO:0032386	regulation of intracellular transport	3	19
4595	GO:0032400	melanosome localization	3	13
4596	GO:0032446	protein modification by small protein conjugation	3	27
4597	GO:0032489	regulation of Cdc42 protein signal transduction	3	52
4598	GO:0032507	maintenance of protein location in cell	3	16
4599	GO:0032717	negative regulation of interleukin-8 production	3	13
4600	GO:0032728	positive regulation of interferon-beta production	3	16
4601	GO:0032729	positive regulation of interferon-gamma production	3	8
4602	GO:0032757	positive regulation of interleukin-8 production	3	4
4603	GO:0032784	regulation of DNA-dependent transcription, elongation	3	22
4604	GO:0032837	distributive segregation	3	3
4605	GO:0032927	positive regulation of activin receptor signaling pathway	3	23
4606	GO:0033047	regulation of mitotic sister chromatid segregation	3	4
4607	GO:0033059	cellular pigmentation	3	4
4608	GO:0033076	isoquinoline alkaloid metabolic process	3	12
4609	GO:0033157	regulation of intracellular protein transport	3	10
4610	GO:0033158	regulation of protein import into nucleus, translocation	3	12
4611	GO:0033184	positive regulation of histone ubiquitination	3	17
4612	GO:0033198	response to ATP	3	10
4613	GO:0033234	negative regulation of protein sumoylation	3	13
4614	GO:0033385	geranylgeranyl diphosphate metabolic process	3	3
4615	GO:0033386	geranylgeranyl diphosphate biosynthetic process	3	5
4616	GO:0033388	putrescine biosynthetic process from arginine	3	8
4617	GO:0033485	cyanidin 3-O-glucoside biosynthetic process	3	6
4618	GO:0033490	cholesterol biosynthetic process via lathosterol	3	10
4619	GO:0033521	phytyl diphosphate biosynthetic process	3	3

4620	GO:0033528	S-methylmethionine cycle	3	26
4621	GO:0033566	gamma-tubulin complex localization	3	44
4622	GO:0033577	protein glycosylation in endoplasmic reticulum	3	19
4623	GO:0033687	osteoblast proliferation	3	16
4624	GO:0033700	phospholipid efflux	3	33
4625	GO:0034120	positive regulation of erythrocyte aggregation	3	3
4626	GO:0034122	negative regulation of toll-like receptor signaling pathway	3	13
4627	GO:0034138	toll-like receptor 3 signaling pathway	3	11
4628	GO:0034227	tRNA thio-modification	3	10
4629	GO:0034284	response to monosaccharide stimulus	3	24
4630	GO:0034312	diol biosynthetic process	3	7
4631	GO:0034383	low-density lipoprotein particle clearance	3	13
4632	GO:0034414	tRNA 3'-trailer cleavage, endonucleolytic	3	29
4633	GO:0034472	snRNA 3'-end processing	3	16
4634	GO:0034473	U1 snRNA 3'-end processing	3	17
4635	GO:0034587	piRNA metabolic process	3	34
4636	GO:0034616	response to laminar fluid shear stress	3	52
4637	GO:0034622	cellular macromolecular complex assembly	3	7
4638	GO:0034724	DNA replication-independent nucleosome organization	3	15
4639	GO:0034754	cellular hormone metabolic process	3	13
4640	GO:0034775	glutathione transmembrane transport	3	11
4641	GO:0034970	histone H3-R2 methylation	3	13
4642	GO:0035046	pronuclear migration	3	3
4643	GO:0035065	regulation of histone acetylation	3	43
4644	GO:0035080	heat shock-mediated polytene chromosome puffing	3	45
4645	GO:0035082	axoneme assembly	3	4
4646	GO:0035088	establishment or maintenance of apical/basal cell polarity	3	13
4647	GO:0035137	hindlimb morphogenesis	3	10
4648	GO:0035214	eye-antennal disc development	3	6
4649	GO:0035239	tube morphogenesis	3	14
4650	GO:0035349	coenzyme A transmembrane transport	3	13
4651	GO:0035551	protein initiator methionine removal involved in protein maturation	3	29
4652	GO:0035552	oxidative single-stranded DNA demethylation	3	4
4653	GO:0035553	oxidative single-stranded RNA demethylation	3	4
4654	GO:0035562	negative regulation of chromatin binding	3	43
4655	GO:0035563	positive regulation of chromatin binding	3	13
4656	GO:0035640	exploration behavior	3	7
4657	GO:0035694	mitochondrial protein catabolic process	3	17
4658	GO:0035897	proteolysis in other organism	3	24
4659	GO:0035914	skeletal muscle cell differentiation	3	7
4660	GO:0036089	cleavage furrow formation	3	7
4661	GO:0036151	phosphatidylcholine acyl-chain remodeling	3	39
4662	GO:0036228	protein targeting to nuclear inner membrane	3	19
4663	GO:0036292	DNA rewinding	3	10
4664	GO:0040024	dauer larval development	3	9
4665	GO:0042102	positive regulation of T cell proliferation	3	17
4666	GO:0042110	T cell activation	3	5
4667	GO:0042167	heme catabolic process	3	7
4668	GO:0042179	nicotine biosynthetic process	3	23
4669	GO:0042180	cellular ketone metabolic process	3	14
4670	GO:0042206	halogenated hydrocarbon catabolic process	3	9
4671	GO:0042303	molting cycle	3	6
4672	GO:0042311	vasodilation	3	6
4673	GO:0042343	indole glucosinolate metabolic process	3	3
4674	GO:0042347	negative regulation of NF-kappaB import into nucleus	3	15
4675	GO:0042421	norepinephrine biosynthetic process	3	10
4676	GO:0042427	serotonin biosynthetic process	3	12
4677	GO:0042430	indole-containing compound metabolic process	3	7

4678	GO:0042445	hormone metabolic process	3	20
4679	GO:0042548	regulation of photosynthesis, light reaction	3	21
4680	GO:0042554	superoxide anion generation	3	19
4681	GO:0042770	signal transduction in response to DNA damage	3	13
4682	GO:0042794	rRNA transcription from plastid promoter	3	25
4683	GO:0042886	amide transport	3	8
4684	GO:0042948	salicin transport	3	17
4685	GO:0042987	amyloid precursor protein catabolic process	3	22
4686	GO:0043132	NAD transport	3	10
4687	GO:0043137	DNA replication, removal of RNA primer	3	16
4688	GO:0043144	snoRNA processing	3	28
4689	GO:0043182	vacuolar sequestering of sodium ion	3	17
4690	GO:0043207	response to external biotic stimulus	3	9
4691	GO:0043242	negative regulation of protein complex disassembly	3	15
4692	GO:0043276	anoikis	3	25
4693	GO:0043281	regulation of cysteine-type endopeptidase activity involved in apoptotic process	3	23
4694	GO:0043327	chemotaxis to cAMP	3	9
4695	GO:0043414	macromolecule methylation	3	13
4696	GO:0043427	carbon fixation by 3-hydroxypropionate cycle	3	17
4697	GO:0043462	regulation of ATPase activity	3	15
4698	GO:0043504	mitochondrial DNA repair	3	45
4699	GO:0043572	plastid fission	3	7
4700	GO:0043617	cellular response to sucrose starvation	3	22
4701	GO:0043618	regulation of transcription from RNA polymerase II promoter in response to stress	3	29
4702	GO:0043629	ncRNA polyadenylation	3	30
4703	GO:0043648	dicarboxylic acid metabolic process	3	3
4704	GO:0043691	reverse cholesterol transport	3	52
4705	GO:0043902	positive regulation of multi-organism process	3	23
4706	GO:0043937	regulation of sporulation	3	11
4707	GO:0043969	histone H2B acetylation	3	35
4708	GO:0044003	modification by symbiont of host morphology or physiology	3	3
4709	GO:0044010	single-species biofilm formation	3	4
4710	GO:0044089	positive regulation of cellular component biogenesis	3	8
4711	GO:0044111	development involved in symbiotic interaction	3	10
4712	GO:0044128	positive regulation of growth of symbiont in host	3	19
4713	GO:0044211	CTP salvage	3	39
4714	GO:0045004	DNA replication proofreading	3	34
4715	GO:0045053	protein retention in Golgi apparatus	3	7
4716	GO:0045056	transcytosis	3	25
4717	GO:0045069	regulation of viral genome replication	3	14
4718	GO:0045124	regulation of bone resorption	3	33
4719	GO:0045212	neurotransmitter receptor biosynthetic process	3	7
4720	GO:0045214	sarcomere organization	3	12
4721	GO:0045216	cell-cell junction organization	3	7
4722	GO:0045226	extracellular polysaccharide biosynthetic process	3	8
4723	GO:0045446	endothelial cell differentiation	3	4
4724	GO:0045464	R8 cell fate specification	3	34
4725	GO:0045610	regulation of hemocyte differentiation	3	13
4726	GO:0045637	regulation of myeloid cell differentiation	3	5
4727	GO:0045667	regulation of osteoblast differentiation	3	14
4728	GO:0045722	positive regulation of gluconeogenesis	3	35
4729	GO:0045760	positive regulation of action potential	3	5
4730	GO:0045792	negative regulation of cell size	3	25
4731	GO:0045806	negative regulation of endocytosis	3	3
4732	GO:0045838	positive regulation of membrane potential	3	5
4733	GO:0045839	negative regulation of mitosis	3	15

4734	GO:0045843	negative regulation of striated muscle tissue development	3	12
4735	GO:0045861	negative regulation of proteolysis	3	15
4736	GO:0045977	positive regulation of mitotic cell cycle, embryonic	3	31
4737	GO:0045991	carbon catabolite activation of transcription	3	14
4738	GO:0046037	GMP metabolic process	3	18
4739	GO:0046040	IMP metabolic process	3	10
4740	GO:0046103	inosine biosynthetic process	3	44
4741	GO:0046187	acetaldehyde catabolic process	3	14
4742	GO:0046202	cyanide biosynthetic process	3	8
4743	GO:0046203	spermidine catabolic process	3	18
4744	GO:0046247	terpene catabolic process	3	20
4745	GO:0046273	lignan catabolic process	3	7
4746	GO:0046292	formaldehyde metabolic process	3	5
4747	GO:0046370	fructose biosynthetic process	3	4
4748	GO:0046390	ribose phosphate biosynthetic process	3	18
4749	GO:0046395	carboxylic acid catabolic process	3	9
4750	GO:0046506	sulfolipid biosynthetic process	3	30
4751	GO:0046514	ceramide catabolic process	3	21
4752	GO:0046595	establishment of pole plasm mRNA localization	3	4
4753	GO:0046604	positive regulation of mitotic centrosome separation	3	3
4754	GO:0046626	regulation of insulin receptor signaling pathway	3	40
4755	GO:0046673	negative regulation of compound eye retinal cell programmed cell death	3	13
4756	GO:0046680	response to DDT	3	26
4757	GO:0046684	response to pyrethroid	3	12
4758	GO:0046690	response to tellurium ion	3	25
4759	GO:0046700	heterocycle catabolic process	3	7
4760	GO:0046726	positive regulation of viral protein levels in host cell	3	41
4761	GO:0046755	non-lytic virus budding	3	4
4762	GO:0046824	positive regulation of nucleocytoplasmic transport	3	10
4763	GO:0046831	regulation of RNA export from nucleus	3	12
4764	GO:0046847	filopodium assembly	3	11
4765	GO:0048210	Golgi vesicle fusion to target membrane	3	5
4766	GO:0048240	sperm capacitation	3	4
4767	GO:0048250	mitochondrial iron ion transport	3	5
4768	GO:0048284	organelle fusion	3	18
4769	GO:0048314	embryo sac morphogenesis	3	13
4770	GO:0048318	axial mesoderm development	3	3
4771	GO:0048383	mesectoderm development	3	9
4772	GO:0048385	regulation of retinoic acid receptor signaling pathway	3	6
4773	GO:0048482	ovule morphogenesis	3	50
4774	GO:0048524	positive regulation of viral reproduction	3	29
4775	GO:0048587	regulation of short-day photoperiodism, flowering	3	5
4776	GO:0048621	post-embryonic digestive tract morphogenesis	3	18
4777	GO:0048679	regulation of axon regeneration	3	33
4778	GO:0048681	negative regulation of axon regeneration	3	20
4779	GO:0048708	astrocyte differentiation	3	12
4780	GO:0048709	oligodendrocyte differentiation	3	17
4781	GO:0048728	proboscis development	3	36
4782	GO:0048738	cardiac muscle tissue development	3	5
4783	GO:0048793	pronephros development	3	8
4784	GO:0048806	genitalia development	3	6
4785	GO:0048865	stem cell fate commitment	3	13
4786	GO:0050702	interleukin-1 beta secretion	3	52
4787	GO:0050708	regulation of protein secretion	3	47
4788	GO:0050709	negative regulation of protein secretion	3	10
4789	GO:0050796	regulation of insulin secretion	3	14
4790	GO:0050848	regulation of calcium-mediated signaling	3	6
4791	GO:0050892	intestinal absorption	3	14

4792	GO:0050898	nitrile metabolic process	3	11
4793	GO:0050906	detection of stimulus involved in sensory perception	3	7
4794	GO:0050955	thermoception	3	11
4795	GO:0050966	detection of mechanical stimulus involved in sensory perception of pain	3	11
4796	GO:0050968	detection of chemical stimulus involved in sensory perception of pain	3	11
4797	GO:0050982	detection of mechanical stimulus	3	6
4798	GO:0050994	regulation of lipid catabolic process	3	24
4799	GO:0051053	negative regulation of DNA metabolic process	3	34
4800	GO:0051084	'de novo' posttranslational protein folding	3	32
4801	GO:0051096	positive regulation of helicase activity	3	46
4802	GO:0051125	regulation of actin nucleation	3	23
4803	GO:0051146	striated muscle cell differentiation	3	6
4804	GO:0051187	cofactor catabolic process	3	12
4805	GO:0051188	cofactor biosynthetic process	3	23
4806	GO:0051253	negative regulation of RNA metabolic process	3	38
4807	GO:0051282	regulation of sequestering of calcium ion	3	14
4808	GO:0051304	chromosome separation	3	33
4809	GO:0051410	detoxification of nitrogen compound	3	5
4810	GO:0051415	interphase microtubule nucleation by interphase microtubule organizing center	3	19
4811	GO:0051454	intracellular pH elevation	3	12
4812	GO:0051469	vesicle fusion with vacuole	3	10
4813	GO:0051484	isopentenyl diphosphate biosynthetic process, mevalonate-independent pathway involved in terpenoid biosynthetic process	3	10
4814	GO:0051573	negative regulation of histone H3-K9 methylation	3	17
4815	GO:0051608	histamine transport	3	8
4816	GO:0051653	spindle localization	3	12
4817	GO:0051770	positive regulation of nitric-oxide synthase biosynthetic process	3	14
4818	GO:0051775	response to redox state	3	12
4819	GO:0051902	negative regulation of mitochondrial depolarization	3	44
4820	GO:0051928	positive regulation of calcium ion transport	3	15
4821	GO:0051932	synaptic transmission, GABAergic	3	9
4822	GO:0051972	regulation of telomerase activity	3	53
4823	GO:0052314	phytoalexin metabolic process	3	12
4824	GO:0052572	response to host immune response	3	15
4825	GO:0052865	1-deoxy-D-xylulose 5-phosphate biosynthetic process	3	28
4826	GO:0055076	transition metal ion homeostasis	3	6
4827	GO:0055080	cation homeostasis	3	6
4828	GO:0055082	cellular chemical homeostasis	3	5
4829	GO:0055086	nucleobase-containing small molecule metabolic process	3	11
4830	GO:0055092	sterol homeostasis	3	16
4831	GO:0055094	response to lipoprotein stimulus	3	25
4832	GO:0055098	response to low-density lipoprotein particle stimulus	3	52
4833	GO:0060065	uterus development	3	6
4834	GO:0060155	platelet dense granule organization	3	52
4835	GO:0060166	olfactory pit development	3	5
4836	GO:0060173	limb development	3	15
4837	GO:0060253	negative regulation of glial cell proliferation	3	34
4838	GO:0060284	regulation of cell development	3	22
4839	GO:0060307	regulation of ventricular cardiomyocyte membrane repolarization	3	7
4840	GO:0060314	regulation of ryanodine-sensitive calcium-release channel activity	3	3
4841	GO:0060322	head development	3	11
4842	GO:0060338	regulation of type I interferon-mediated signaling pathway	3	48
4843	GO:0060339	negative regulation of type I interferon-mediated signaling pathway	3	13

4844	GO:0060393	regulation of pathway-restricted SMAD protein phosphorylation	3	3
4845	GO:0060395	SMAD protein signal transduction	3	20
4846	GO:0060415	muscle tissue morphogenesis	3	10
4847	GO:0060465	pharynx development	3	18
4848	GO:0060539	diaphragm development	3	9
4849	GO:0060541	respiratory system development	3	13
4850	GO:0060561	apoptotic process involved in morphogenesis	3	5
4851	GO:0060632	regulation of microtubule-based movement	3	15
4852	GO:0060702	negative regulation of endoribonuclease activity	3	14
4853	GO:0060733	regulation of eIF2 alpha phosphorylation by amino acid starvation	3	4
4854	GO:0060840	artery development	3	23
4855	GO:0060963	positive regulation of ribosomal protein gene transcription from RNA polymerase II promoter	3	4
4856	GO:0060969	negative regulation of gene silencing	3	22
4857	GO:0061003	positive regulation of dendritic spine morphogenesis	3	12
4858	GO:0061041	regulation of wound healing	3	23
4859	GO:0061057	peptidoglycan recognition protein signaling pathway	3	31
4860	GO:0061067	negative regulation of dauer larval development	3	18
4861	GO:0061166	establishment of endoplasmic reticulum localization involved in endoplasmic reticulum polarization at cell division site	3	4
4862	GO:0061337	cardiac conduction	3	11
4863	GO:0061343	cell adhesion involved in heart morphogenesis	3	9
4864	GO:0061357	positive regulation of Wnt protein secretion	3	25
4865	GO:0061412	positive regulation of transcription from RNA polymerase II promoter in response to amino acid starvation	3	19
4866	GO:0070126	mitochondrial translational termination	3	19
4867	GO:0070131	positive regulation of mitochondrial translation	3	13
4868	GO:0070179	D-serine biosynthetic process	3	16
4869	GO:0070201	regulation of establishment of protein localization	3	8
4870	GO:0070231	T cell apoptotic process	3	11
4871	GO:0070433	negative regulation of nucleotide-binding oligomerization domain containing 2 signaling pathway	3	5
4872	GO:0070455	positive regulation of heme biosynthetic process	3	8
4873	GO:0070476	rRNA (guanine-N7)-methylation	3	18
4874	GO:0070482	response to oxygen levels	3	6
4875	GO:0070543	response to linoleic acid	3	24
4876	GO:0070564	positive regulation of vitamin D receptor signaling pathway	3	6
4877	GO:0070601	centromeric sister chromatid cohesion	3	17
4878	GO:0070627	ferrous iron import	3	13
4879	GO:0070633	transepithelial transport	3	21
4880	GO:0070781	response to biotin	3	9
4881	GO:0070827	chromatin maintenance	3	23
4882	GO:0070830	tight junction assembly	3	21
4883	GO:0070845	polyubiquitinated misfolded protein transport	3	15
4884	GO:0070846	Hsp90 deacetylation	3	15
4885	GO:0070848	response to growth factor stimulus	3	15
4886	GO:0070880	fungal-type cell wall beta-glucan biosynthetic process	3	13
4887	GO:0070886	positive regulation of calcineurin-NFAT signaling cascade	3	11
4888	GO:0070900	mitochondrial tRNA modification	3	4
4889	GO:0070921	regulation of production of siRNA involved in chromatin silencing by small RNA	3	42
4890	GO:0070934	CRD-mediated mRNA stabilization	3	27
4891	GO:0070966	nuclear-transcribed mRNA catabolic process, no-go decay	3	19
4892	GO:0070981	L-asparagine biosynthetic process	3	6

4893	GO:0071033	nuclear retention of pre-mRNA at the site of transcription	3	18
4894	GO:0071039	nuclear polyadenylation-dependent CUT catabolic process	3	16
4895	GO:0071271	1-butanol biosynthetic process	3	13
4896	GO:0071312	cellular response to alkaloid	3	12
4897	GO:0071313	cellular response to caffeine	3	19
4898	GO:0071395	cellular response to jasmonic acid stimulus	3	8
4899	GO:0071484	cellular response to light intensity	3	3
4900	GO:0071496	cellular response to external stimulus	3	5
4901	GO:0071503	response to heparin	3	12
4902	GO:0071709	membrane assembly	3	5
4903	GO:0071803	positive regulation of podosome assembly	3	10
4904	GO:0071816	tail-anchored membrane protein insertion into ER membrane	3	35
4905	GO:0072002	Malpighian tubule development	3	5
4906	GO:0072347	response to anesthetic	3	31
4907	GO:0072385	minus-end-directed organelle transport along microtubule	3	7
4908	GO:0072643	interferon-gamma secretion	3	7
4909	GO:0072657	protein localization in membrane	3	18
4910	GO:0075133	modulation by symbiont of host calcium or calmodulin-mediated signal transduction	3	3
4911	GO:0075177	regulation of calcium or calmodulin-mediated signal transduction in response to host	3	3
4912	GO:0080021	response to benzoic acid stimulus	3	11
4913	GO:0080153	negative regulation of reductive pentose-phosphate cycle	3	3
4914	GO:0080175	phragmoplast microtubule organization	3	7
4915	GO:0080181	lateral root branching	3	7
4916	GO:0086012	membrane depolarization involved in regulation of cardiac muscle cell action potential	3	18
4917	GO:0086019	cell-cell signaling involved in cardiac conduction	3	31
4918	GO:0090043	regulation of tubulin deacetylation	3	35
4919	GO:0090069	regulation of ribosome biogenesis	3	12
4920	GO:0090071	negative regulation of ribosome biogenesis	3	17
4921	GO:0090073	positive regulation of protein homodimerization activity	3	15
4922	GO:0090170	regulation of Golgi inheritance	3	24
4923	GO:0090201	negative regulation of release of cytochrome c from mitochondria	3	12
4924	GO:0090213	regulation of radial pattern formation	3	45
4925	GO:0090297	positive regulation of mitochondrial DNA replication	3	9
4926	GO:0090306	spindle assembly involved in meiosis	3	10
4927	GO:0090314	positive regulation of protein targeting to membrane	3	12
4928	GO:0090324	negative regulation of oxidative phosphorylation	3	12
4929	GO:0090377	seed trichome initiation	3	3
4930	GO:0090378	seed trichome elongation	3	3
4931	GO:0090407	organophosphate biosynthetic process	3	16
4932	GO:0097050	type B pancreatic cell apoptotic process	3	42
4933	GO:0097051	establishment of protein localization in endoplasmic reticulum membrane	3	39
4934	GO:0097167	circadian regulation of translation	3	6
4935	GO:0097274	urea homeostasis	3	14
4936	GO:0097284	hepatocyte apoptotic process	3	33
4937	GO:0097298	regulation of nucleus size	3	23
4938	GO:1900016	negative regulation of cytokine production involved in inflammatory response	3	8
4939	GO:1900027	regulation of ruffle assembly	3	24
4940	GO:1900030	regulation of pectin biosynthetic process	3	4
4941	GO:1900060	negative regulation of ceramide biosynthetic process	3	4
4942	GO:1900101	regulation of endoplasmic reticulum unfolded protein response	3	5

4943	GO:1900160	plastid DNA packaging	3	22
4944	GO:1900180	regulation of protein localization to nucleus	3	3
4945	GO:1900181	negative regulation of protein localization to nucleus	3	17
4946	GO:1900370	positive regulation of RNA interference	3	13
4947	GO:1900439	positive regulation of filamentous growth of a population of unicellular organisms in response to chemical stimulus	3	22
4948	GO:1900827	positive regulation of membrane depolarization involved in regulation of cardiac muscle cell action potential	3	5
4949	GO:2000011	regulation of adaxial/abaxial pattern formation	3	68
4950	GO:2000123	positive regulation of stomatal complex development	3	4
4951	GO:2000144	positive regulation of DNA-dependent transcription, initiation	3	43
4952	GO:2000146	negative regulation of cell motility	3	3
4953	GO:2000158	positive regulation of ubiquitin-specific protease activity	3	4
4954	GO:2000189	positive regulation of cholesterol homeostasis	3	9
4955	GO:2000214	regulation of proline metabolic process	3	6
4956	GO:2000219	positive regulation of invasive growth in response to glucose limitation	3	19
4957	GO:2000251	positive regulation of actin cytoskeleton reorganization	3	12
4958	GO:2000276	negative regulation of oxidative phosphorylation uncoupler activity	3	12
4959	GO:2000488	positive regulation of brassinosteroid biosynthetic process	3	9
4960	GO:2000582	positive regulation of plus-end-directed microtubule motor activity	3	4
4961	GO:2000615	regulation of histone H3-K9 acetylation	3	12
4962	GO:2000678	negative regulation of transcription regulatory region DNA binding	3	17
4963	GO:2000728	regulation of mRNA export from nucleus in response to heat stress	3	25
4964	GO:2000749	positive regulation of chromatin silencing at rDNA	3	3
4965	GO:2000772	regulation of cellular senescence	3	9
4966	GO:2000806	positive regulation of termination of RNA polymerase II transcription, poly(A)-coupled	3	43
4967	GO:2001014	regulation of skeletal muscle cell differentiation	3	15
4968	GO:2001141	regulation of RNA biosynthetic process	3	20
4969	GO:2001162	positive regulation of histone H3-K79 methylation	3	14
4970	GO:2001259	positive regulation of cation channel activity	3	5
4971	GO:2001286	regulation of caveolin-mediated endocytosis	3	44
4972	GO:0000012	single strand break repair	2	35
4973	GO:0000303	response to superoxide	2	15
4974	GO:0000379	tRNA-type intron splice site recognition and cleavage	2	9
4975	GO:0000394	RNA splicing, via endonucleolytic cleavage and ligation	2	3
4976	GO:0000395	nuclear mRNA 5'-splice site recognition	2	7
4977	GO:0000464	endonucleolytic cleavage in ITS1 upstream of 5.8S rRNA from tricistronic rRNA transcript (SSU-rRNA, 5.8S rRNA, LSU-rRNA)	2	4
4978	GO:0000725	recombinational repair	2	22
4979	GO:0000742	karyogamy involved in conjugation with cellular fusion	2	9
4980	GO:0000743	nuclear migration involved in conjugation with cellular fusion	2	9
4981	GO:0000753	cell morphogenesis involved in conjugation with cellular fusion	2	9
4982	GO:0000909	sporocarp development involved in sexual reproduction	2	10
4983	GO:0000918	barrier septum site selection	2	3
4984	GO:0000947	amino acid catabolic process to alcohol via Ehrlich pathway	2	5
4985	GO:0000957	mitochondrial RNA catabolic process	2	4
4986	GO:0000965	mitochondrial RNA 3'-end processing	2	20
4987	GO:0001123	transcription initiation from bacterial-type RNA polymerase promoter	2	22
4988	GO:0001522	pseudouridine synthesis	2	11
4989	GO:0001569	patterning of blood vessels	2	11

4990	GO:0001656	metanephros development	2	3
4991	GO:0001667	ameboidal cell migration	2	3
4992	GO:0001706	endoderm formation	2	9
4993	GO:0001737	establishment of imaginal disc-derived wing hair orientation	2	4
4994	GO:0001754	eye photoreceptor cell differentiation	2	6
4995	GO:0001755	neural crest cell migration	2	14
4996	GO:0001833	inner cell mass cell proliferation	2	6
4997	GO:0001892	embryonic placenta development	2	20
4998	GO:0001896	autolysis	2	18
4999	GO:0001913	T cell mediated cytotoxicity	2	3
5000	GO:0001958	endochondral ossification	2	14
5001	GO:0001967	suckling behavior	2	15
5002	GO:0002035	brain renin-angiotensin system	2	11
5003	GO:0002040	sprouting angiogenesis	2	5
5004	GO:0002052	positive regulation of neuroblast proliferation	2	10
5005	GO:0002087	regulation of respiratory gaseous exchange by neurological system process	2	15
5006	GO:0002155	regulation of thyroid hormone mediated signaling pathway	2	36
5007	GO:0002164	larval development	2	4
5008	GO:0002165	instar larval or pupal development	2	5
5009	GO:0002183	cytoplasmic translational initiation	2	8
5010	GO:0002191	cap-dependent translational initiation	2	5
5011	GO:0002192	IRES-dependent translational initiation	2	6
5012	GO:0002263	cell activation involved in immune response	2	7
5013	GO:0002361	CD4-positive, CD25-positive, alpha-beta regulatory T cell differentiation	2	36
5014	GO:0002428	antigen processing and presentation of peptide antigen via MHC class Ib	2	15
5015	GO:0002483	antigen processing and presentation of endogenous peptide antigen	2	15
5016	GO:0002521	leukocyte differentiation	2	4
5017	GO:0002548	monocyte chemotaxis	2	11
5018	GO:0002573	myeloid leukocyte differentiation	2	4
5019	GO:0002574	thrombocyte differentiation	2	9
5020	GO:0002606	positive regulation of dendritic cell antigen processing and presentation	2	5
5021	GO:0002684	positive regulation of immune system process	2	4
5022	GO:0002699	positive regulation of immune effector process	2	12
5023	GO:0002790	peptide secretion	2	30
5024	GO:0002827	positive regulation of T-helper 1 type immune response	2	5
5025	GO:0002902	regulation of B cell apoptotic process	2	15
5026	GO:0003012	muscle system process	2	17
5027	GO:0003015	heart process	2	12
5028	GO:0003016	respiratory system process	2	6
5029	GO:0003073	regulation of systemic arterial blood pressure	2	8
5030	GO:0003214	cardiac left ventricle morphogenesis	2	8
5031	GO:0003247	post-embryonic cardiac muscle cell growth involved in heart morphogenesis	2	27
5032	GO:0003342	proepicardium development	2	3
5033	GO:0003406	retinal pigment epithelium development	2	10
5034	GO:0005998	xylulose catabolic process	2	46
5035	GO:0006038	cell wall chitin biosynthetic process	2	14
5036	GO:0006041	glucosamine metabolic process	2	18
5037	GO:0006042	glucosamine biosynthetic process	2	14
5038	GO:0006047	UDP-N-acetylglucosamine metabolic process	2	20
5039	GO:0006152	purine nucleoside catabolic process	2	2
5040	GO:0006166	purine ribonucleoside salvage	2	14
5041	GO:0006175	dATP biosynthetic process	2	4
5042	GO:0006213	pyrimidine nucleoside metabolic process	2	5

5043	GO:0006214	thymidine catabolic process	2	3
5044	GO:0006220	pyrimidine nucleotide metabolic process	2	8
5045	GO:0006227	dUDP biosynthetic process	2	24
5046	GO:0006233	dTDP biosynthetic process	2	24
5047	GO:0006267	pre-replicative complex assembly	2	2
5048	GO:0006286	base-excision repair, base-free sugar-phosphate removal	2	13
5049	GO:0006288	base-excision repair, DNA ligation	2	27
5050	GO:0006290	pyrimidine dimer repair	2	16
5051	GO:0006309	apoptotic DNA fragmentation	2	5
5052	GO:0006354	DNA-dependent transcription, elongation	2	13
5053	GO:0006429	leucyl-tRNA aminoacylation	2	6
5054	GO:0006430	lysyl-tRNA aminoacylation	2	16
5055	GO:0006432	phenylalanyl-tRNA aminoacylation	2	11
5056	GO:0006434	seryl-tRNA aminoacylation	2	11
5057	GO:0006501	C-terminal protein lipidation	2	13
5058	GO:0006528	asparagine metabolic process	2	4
5059	GO:0006529	asparagine biosynthetic process	2	17
5060	GO:0006543	glutamine catabolic process	2	15
5061	GO:0006666	3-keto-sphinganine metabolic process	2	3
5062	GO:0006671	phytosphingosine metabolic process	2	11
5063	GO:0006673	inositolphosphoceramide metabolic process	2	8
5064	GO:0006700	C21-steroid hormone biosynthetic process	2	44
5065	GO:0006707	cholesterol catabolic process	2	43
5066	GO:0006714	sesquiterpenoid metabolic process	2	3
5067	GO:0006729	tetrahydrobiopterin biosynthetic process	2	5
5068	GO:0006735	NADH regeneration	2	2
5069	GO:0006760	folic acid-containing compound metabolic process	2	3
5070	GO:0006761	dihydrofolate biosynthetic process	2	16
5071	GO:0006778	porphyrin-containing compound metabolic process	2	6
5072	GO:0006781	succinyl-CoA pathway	2	2
5073	GO:0006849	plasma membrane pyruvate transport	2	10
5074	GO:0006856	eye pigment precursor transport	2	30
5075	GO:0006858	extracellular transport	2	3
5076	GO:0006884	cell volume homeostasis	2	15
5077	GO:0006901	vesicle coating	2	5
5078	GO:0006975	DNA damage induced protein phosphorylation	2	50
5079	GO:0007091	mitotic metaphase/anaphase transition	2	47
5080	GO:0007110	cytokinesis after meiosis I	2	3
5081	GO:0007111	cytokinesis after meiosis II	2	3
5082	GO:0007156	homophilic cell adhesion	2	16
5083	GO:0007172	signal complex assembly	2	30
5084	GO:0007174	epidermal growth factor catabolic process	2	3
5085	GO:0007176	regulation of epidermal growth factor-activated receptor activity	2	4
5086	GO:0007190	activation of adenylate cyclase activity	2	8
5087	GO:0007234	osmosensory signaling pathway via two-component system	2	19
5088	GO:0007249	I-kappaB kinase/NF-kappaB cascade	2	13
5089	GO:0007266	Rho protein signal transduction	2	18
5090	GO:0007280	pole cell migration	2	12
5091	GO:0007303	cytoplasmic transport, nurse cell to oocyte	2	13
5092	GO:0007309	oocyte axis specification	2	11
5093	GO:0007344	pronuclear fusion	2	2
5094	GO:0007362	terminal region determination	2	12
5095	GO:0007370	ventral furrow formation	2	2
5096	GO:0007390	germ-band shortening	2	12
5097	GO:0007400	neuroblast fate determination	2	32
5098	GO:0007431	salivary gland development	2	12
5099	GO:0007436	larval salivary gland morphogenesis	2	12
5100	GO:0007444	imaginal disc development	2	4

5101	GO:0007464	R3/R4 cell fate commitment	2	4
5102	GO:0007477	notum development	2	12
5103	GO:0007479	leg disc proximal/distal pattern formation	2	12
5104	GO:0007530	sex determination	2	3
5105	GO:0007594	puparial adhesion	2	13
5106	GO:0007603	phototransduction, visible light	2	30
5107	GO:0008057	eye pigment granule organization	2	10
5108	GO:0008071	maternal determination of dorsal/ventral axis, ovarian follicular epithelium, soma encoded	2	12
5109	GO:0008105	asymmetric protein localization	2	4
5110	GO:0008154	actin polymerization or depolymerization	2	8
5111	GO:0008218	bioluminescence	2	4
5112	GO:0008292	acetylcholine biosynthetic process	2	12
5113	GO:0008293	torso signaling pathway	2	13
5114	GO:0008306	associative learning	2	6
5115	GO:0008362	chitin-based embryonic cuticle biosynthetic process	2	12
5116	GO:0008611	ether lipid biosynthetic process	2	14
5117	GO:0008612	peptidyl-lysine modification to hypusine	2	17
5118	GO:0009067	aspartate family amino acid biosynthetic process	2	3
5119	GO:0009070	serine family amino acid biosynthetic process	2	4
5120	GO:0009090	homoserine biosynthetic process	2	17
5121	GO:0009095	aromatic amino acid family biosynthetic process, prephenate pathway	2	18
5122	GO:0009115	xanthine catabolic process	2	6
5123	GO:0009190	cyclic nucleotide biosynthetic process	2	19
5124	GO:0009200	deoxyribonucleoside triphosphate metabolic process	2	8
5125	GO:0009220	pyrimidine ribonucleotide biosynthetic process	2	6
5126	GO:0009257	10-formyltetrahydrofolate biosynthetic process	2	5
5127	GO:0009307	DNA restriction-modification system	2	6
5128	GO:0009313	oligosaccharide catabolic process	2	3
5129	GO:0009442	allantoin assimilation pathway	2	12
5130	GO:0009445	putrescine metabolic process	2	3
5131	GO:0009447	putrescine catabolic process	2	5
5132	GO:0009560	embryo sac egg cell differentiation	2	11
5133	GO:0009566	fertilization	2	10
5134	GO:0009597	detection of virus	2	14
5135	GO:0009710	tropane alkaloid biosynthetic process	2	2
5136	GO:0009745	sucrose mediated signaling	2	21
5137	GO:0009780	photosynthetic NADP+ reduction	2	2
5138	GO:0009900	dehiscence	2	7
5139	GO:0010024	phytochromobilin biosynthetic process	2	2
5140	GO:0010036	response to boron-containing substance	2	3
5141	GO:0010124	phenylacetate catabolic process	2	2
5142	GO:0010239	chloroplast mRNA processing	2	4
5143	GO:0010258	NADH dehydrogenase complex (plastoquinone) assembly	2	2
5144	GO:0010260	organ senescence	2	23
5145	GO:0010270	photosystem II oxygen evolving complex assembly	2	22
5146	GO:0010342	endosperm cellularization	2	5
5147	GO:0010350	cellular response to magnesium starvation	2	3
5148	GO:0010378	temperature compensation of the circadian clock	2	11
5149	GO:0010401	pectic galactan metabolic process	2	13
5150	GO:0010479	stele development	2	15
5151	GO:0010518	positive regulation of phospholipase activity	2	10
5152	GO:0010525	regulation of transposition, RNA-mediated	2	36
5153	GO:0010571	positive regulation of DNA replication involved in S phase	2	28
5154	GO:0010575	positive regulation vascular endothelial growth factor production	2	10
5155	GO:0010585	glutamine secretion	2	2

5156	GO:0010603	regulation of cytoplasmic mRNA processing body assembly	2	15
5157	GO:0010609	posttranscriptional regulation of gene expression by mRNA localization	2	16
5158	GO:0010642	negative regulation of platelet-derived growth factor receptor signaling pathway	2	3
5159	GO:0010716	negative regulation of extracellular matrix disassembly	2	2
5160	GO:0010761	fibroblast migration	2	6
5161	GO:0010766	negative regulation of sodium ion transport	2	5
5162	GO:0010770	positive regulation of cell morphogenesis involved in differentiation	2	12
5163	GO:0010777	meiotic mismatch repair involved in reciprocal meiotic recombination	2	40
5164	GO:0010807	regulation of synaptic vesicle priming	2	2
5165	GO:0010837	regulation of keratinocyte proliferation	2	3
5166	GO:0010887	negative regulation of cholesterol storage	2	30
5167	GO:0010905	negative regulation of UDP-glucose catabolic process	2	8
5168	GO:0010950	positive regulation of endopeptidase activity	2	3
5169	GO:0010959	regulation of metal ion transport	2	2
5170	GO:0014002	astrocyte development	2	6
5171	GO:0014012	peripheral nervous system axon regeneration	2	3
5172	GO:0014049	positive regulation of glutamate secretion	2	3
5173	GO:0014065	phosphatidylinositol 3-kinase cascade	2	3
5174	GO:0014074	response to purine-containing compound	2	7
5175	GO:0014832	urinary bladder smooth muscle contraction	2	4
5176	GO:0014866	skeletal myofibril assembly	2	7
5177	GO:0014891	striated muscle atrophy	2	23
5178	GO:0014902	myotube differentiation	2	9
5179	GO:0015673	silver ion transport	2	12
5180	GO:0015716	organic phosphonate transport	2	4
5181	GO:0015718	monocarboxylic acid transport	2	13
5182	GO:0015720	allantoin transport	2	24
5183	GO:0015729	oxaloacetate transport	2	11
5184	GO:0015742	alpha-ketoglutarate transport	2	11
5185	GO:0015749	monosaccharide transport	2	16
5186	GO:0015750	pentose transport	2	5
5187	GO:0015766	disaccharide transport	2	10
5188	GO:0015772	oligosaccharide transport	2	4
5189	GO:0015774	polysaccharide transport	2	3
5190	GO:0015805	S-adenosylmethionine transport	2	2
5191	GO:0015818	isoleucine transport	2	15
5192	GO:0015828	tyrosine transport	2	15
5193	GO:0015857	uracil transport	2	24
5194	GO:0015871	choline transport	2	14
5195	GO:0015889	cobalamin transport	2	13
5196	GO:0015946	methanol oxidation	2	2
5197	GO:0015964	diadenosine triphosphate catabolic process	2	13
5198	GO:0015993	molecular hydrogen transport	2	10
5199	GO:0016074	snoRNA metabolic process	2	8
5200	GO:0016180	snRNA processing	2	13
5201	GO:0016198	axon choice point recognition	2	6
5202	GO:0016254	preassembly of GPI anchor in ER membrane	2	28
5203	GO:0016318	ommatidial rotation	2	12
5204	GO:0016330	second mitotic wave involved in compound eye morphogenesis	2	12
5205	GO:0016334	establishment or maintenance of polarity of follicular epithelium	2	4
5206	GO:0016336	establishment or maintenance of polarity of larval imaginal disc epithelium	2	4
5207	GO:0016447	somatic recombination of immunoglobulin gene segments	2	41

5208	GO:0016480	negative regulation of transcription from RNA polymerase III promoter	2	8
5209	GO:0016539	intein-mediated protein splicing	2	8
5210	GO:0016561	protein import into peroxisome matrix, translocation	2	13
5211	GO:0016999	antibiotic metabolic process	2	39
5212	GO:0017006	protein-tetrapyrrole linkage	2	14
5213	GO:0017085	response to insecticide	2	12
5214	GO:0017198	N-terminal peptidyl-serine acetylation	2	2
5215	GO:0018002	N-terminal peptidyl-glutamic acid acetylation	2	2
5216	GO:0018146	keratan sulfate biosynthetic process	2	16
5217	GO:0018149	peptide cross-linking	2	35
5218	GO:0018160	peptidyl-pyrromethane cofactor linkage	2	10
5219	GO:0018216	peptidyl-arginine methylation	2	26
5220	GO:0018316	peptide cross-linking via L-cystine	2	2
5221	GO:0018940	orcinol metabolic process	2	10
5222	GO:0019074	viral RNA genome packaging	2	8
5223	GO:0019218	regulation of steroid metabolic process	2	12
5224	GO:0019228	regulation of action potential in neuron	2	4
5225	GO:0019264	glycine biosynthetic process from serine	2	2
5226	GO:0019276	UDP-N-acetylgalactosamine metabolic process	2	10
5227	GO:0019285	glycine betaine biosynthetic process from choline	2	11
5228	GO:0019323	pentose catabolic process	2	7
5229	GO:0019363	pyridine nucleotide biosynthetic process	2	14
5230	GO:0019365	pyridine nucleotide salvage	2	3
5231	GO:0019408	dolichol biosynthetic process	2	15
5232	GO:0019450	L-cysteine catabolic process to pyruvate	2	5
5233	GO:0019471	4-hydroxyproline metabolic process	2	13
5234	GO:0019569	L-arabinose catabolic process to xylulose 5-phosphate	2	2
5235	GO:0019588	anaerobic glycerol catabolic process	2	29
5236	GO:0019628	urate catabolic process	2	8
5237	GO:0019637	organophosphate metabolic process	2	13
5238	GO:0019661	glucose catabolic process to lactate via pyruvate	2	4
5239	GO:0019673	GDP-mannose metabolic process	2	2
5240	GO:0019742	pentacyclic triterpenoid metabolic process	2	13
5241	GO:0019805	quinolinate biosynthetic process	2	10
5242	GO:0019856	pyrimidine nucleobase biosynthetic process	2	4
5243	GO:0019878	lysine biosynthetic process via aminoadipic acid	2	6
5244	GO:0019933	cAMP-mediated signaling	2	8
5245	GO:0019991	septate junction assembly	2	4
5246	GO:0020027	hemoglobin metabolic process	2	12
5247	GO:0021517	ventral spinal cord development	2	5
5248	GO:0021589	cerebellum structural organization	2	4
5249	GO:0021691	cerebellar Purkinje cell layer maturation	2	6
5250	GO:0021700	developmental maturation	2	4
5251	GO:0021794	thalamus development	2	36
5252	GO:0021888	hypothalamus gonadotrophin-releasing hormone neuron development	2	5
5253	GO:0022408	negative regulation of cell-cell adhesion	2	8
5254	GO:0022411	cellular component disassembly	2	12
5255	GO:0022602	ovulation cycle process	2	26
5256	GO:0022615	protein to membrane docking	2	3
5257	GO:0022619	generative cell differentiation	2	19
5258	GO:0030004	cellular monovalent inorganic cation homeostasis	2	6
5259	GO:0030032	lamellipodium assembly	2	9
5260	GO:0030043	actin filament fragmentation	2	5
5261	GO:0030070	insulin processing	2	13
5262	GO:0030072	peptide hormone secretion	2	5
5263	GO:0030157	pancreatic juice secretion	2	15
5264	GO:0030201	heparan sulfate proteoglycan metabolic process	2	7
5265	GO:0030204	chondroitin sulfate metabolic process	2	7

5266	GO:0030220	platelet formation	2	16
5267	GO:0030263	apoptotic chromosome condensation	2	3
5268	GO:0030301	cholesterol transport	2	5
5269	GO:0030326	embryonic limb morphogenesis	2	5
5270	GO:0030416	methylamine metabolic process	2	2
5271	GO:0030500	regulation of bone mineralization	2	5
5272	GO:0030534	adult behavior	2	15
5273	GO:0030574	collagen catabolic process	2	6
5274	GO:0030713	ovarian follicle cell stalk formation	2	7
5275	GO:0030728	ovulation	2	14
5276	GO:0030814	regulation of cAMP metabolic process	2	15
5277	GO:0030834	regulation of actin filament depolymerization	2	12
5278	GO:0030838	positive regulation of actin filament polymerization	2	10
5279	GO:0030851	granulocyte differentiation	2	2
5280	GO:0030854	positive regulation of granulocyte differentiation	2	34
5281	GO:0030878	thyroid gland development	2	23
5282	GO:0030889	negative regulation of B cell proliferation	2	50
5283	GO:0030948	negative regulation of vascular endothelial growth factor receptor signaling pathway	2	18
5284	GO:0031016	pancreas development	2	14
5285	GO:0031063	regulation of histone deacetylation	2	20
5286	GO:0031086	nuclear-transcribed mRNA catabolic process, deadenylation-independent decay	2	3
5287	GO:0031114	regulation of microtubule depolymerization	2	17
5288	GO:0031123	RNA 3'-end processing	2	15
5289	GO:0031139	positive regulation of conjugation with cellular fusion	2	2
5290	GO:0031144	proteasome localization	2	21
5291	GO:0031452	negative regulation of heterochromatin assembly	2	6
5292	GO:0031453	positive regulation of heterochromatin assembly	2	10
5293	GO:0031542	positive regulation of anthocyanin biosynthetic process	2	15
5294	GO:0031567	cell size control checkpoint	2	6
5295	GO:0031570	DNA integrity checkpoint	2	4
5296	GO:0031589	cell-substrate adhesion	2	12
5297	GO:0031644	regulation of neurological system process	2	11
5298	GO:0031658	negative regulation of cyclin-dependent protein kinase activity involved in G1/S	2	15
5299	GO:0031668	cellular response to extracellular stimulus	2	4
5300	GO:0031848	protection from non-homologous end joining at telomere	2	10
5301	GO:0031940	positive regulation of chromatin silencing at telomere	2	27
5302	GO:0032026	response to magnesium ion	2	10
5303	GO:0032095	regulation of response to food	2	15
5304	GO:0032199	reverse transcription involved in RNA-mediated transposition	2	6
5305	GO:0032203	telomere formation via telomerase	2	11
5306	GO:0032205	negative regulation of telomere maintenance	2	17
5307	GO:0032232	negative regulation of actin filament bundle assembly	2	4
5308	GO:0032264	IMP salvage	2	4
5309	GO:0032287	peripheral nervous system myelin maintenance	2	10
5310	GO:0032330	regulation of chondrocyte differentiation	2	25
5311	GO:0032350	regulation of hormone metabolic process	2	2
5312	GO:0032372	negative regulation of sterol transport	2	2
5313	GO:0032383	regulation of intracellular cholesterol transport	2	32
5314	GO:0032434	regulation of proteasomal ubiquitin-dependent protein catabolic process	2	10
5315	GO:0032459	regulation of protein oligomerization	2	10
5316	GO:0032468	Golgi calcium ion homeostasis	2	11
5317	GO:0032472	Golgi calcium ion transport	2	11
5318	GO:0032486	Rap protein signal transduction	2	12
5319	GO:0032510	endosome to lysosome transport via multivesicular body sorting pathway	2	4

5320	GO:0032515	negative regulation of phosphoprotein phosphatase activity	2	5
5321	GO:0032632	interleukin-3 production	2	5
5322	GO:0032637	interleukin-8 production	2	4
5323	GO:0032675	regulation of interleukin-6 production	2	4
5324	GO:0032692	negative regulation of interleukin-1 production	2	4
5325	GO:0032703	negative regulation of interleukin-2 production	2	14
5326	GO:0032774	RNA biosynthetic process	2	18
5327	GO:0032792	negative regulation of CREB transcription factor activity	2	8
5328	GO:0032844	regulation of homeostatic process	2	11
5329	GO:0032846	positive regulation of homeostatic process	2	2
5330	GO:0032928	regulation of superoxide anion generation	2	10
5331	GO:0032995	regulation of fungal-type cell wall biogenesis	2	2
5332	GO:0033032	regulation of myeloid cell apoptotic process	2	15
5333	GO:0033081	regulation of T cell differentiation in thymus	2	5
5334	GO:0033133	positive regulation of glucokinase activity	2	8
5335	GO:0033145	positive regulation of intracellular steroid hormone receptor signaling pathway	2	2
5336	GO:0033316	meiotic spindle assembly checkpoint	2	6
5337	GO:0033342	negative regulation of collagen binding	2	8
5338	GO:0033358	UDP-L-arabinose biosynthetic process	2	13
5339	GO:0033363	secretory granule organization	2	13
5340	GO:0033387	putrescine biosynthetic process from ornithine	2	7
5341	GO:0033486	delphinidin 3-O-glucoside biosynthetic process	2	5
5342	GO:0033488	cholesterol biosynthetic process via 24,25-dihydrolanosterol	2	17
5343	GO:0033512	L-lysine catabolic process to acetyl-CoA via saccharopine	2	9
5344	GO:0033625	positive regulation of integrin activation	2	30
5345	GO:0033632	regulation of cell-cell adhesion mediated by integrin	2	2
5346	GO:0033859	furaldehyde metabolic process	2	5
5347	GO:0034089	establishment of meiotic sister chromatid cohesion	2	8
5348	GO:0034196	acylglycerol transport	2	11
5349	GO:0034247	snoRNA splicing	2	6
5350	GO:0034379	very-low-density lipoprotein particle assembly	2	15
5351	GO:0034398	telomere tethering at nuclear periphery	2	16
5352	GO:0034508	centromere complex assembly	2	29
5353	GO:0034514	mitochondrial unfolded protein response	2	2
5354	GO:0034629	cellular protein complex localization	2	8
5355	GO:0034635	glutathione transport	2	14
5356	GO:0034756	regulation of iron ion transport	2	2
5357	GO:0034969	histone arginine methylation	2	6
5358	GO:0034971	histone H3-R17 methylation	2	11
5359	GO:0034972	histone H3-R26 methylation	2	11
5360	GO:0035002	liquid clearance, open tracheal system	2	13
5361	GO:0035079	polytene chromosome puffing	2	5
5362	GO:0035099	hemocyte migration	2	2
5363	GO:0035147	branch fusion, open tracheal system	2	12
5364	GO:0035149	lumen formation, open tracheal system	2	12
5365	GO:0035158	regulation of tube diameter, open tracheal system	2	12
5366	GO:0035159	regulation of tube length, open tracheal system	2	13
5367	GO:0035160	maintenance of epithelial integrity, open tracheal system	2	12
5368	GO:0035204	negative regulation of lamellocyte differentiation	2	2
5369	GO:0035207	negative regulation of hemocyte proliferation	2	2
5370	GO:0035225	determination of genital disc primordium	2	12
5371	GO:0035277	spiracle morphogenesis, open tracheal system	2	12
5372	GO:0035281	pre-miRNA export from nucleus	2	17
5373	GO:0035282	segmentation	2	4
5374	GO:0035310	notum cell fate specification	2	12
5375	GO:0035321	maintenance of imaginal disc-derived wing hair orientation	2	32

5376	GO:0035344	hypoxanthine transport	2	2
5377	GO:0035522	monoubiquitinated histone H2A deubiquitination	2	7
5378	GO:0035523	protein K29-linked deubiquitination	2	13
5379	GO:0035567	non-canonical Wnt receptor signaling pathway	2	10
5380	GO:0035600	tRNA methylthiolation	2	10
5381	GO:0035641	locomotory exploration behavior	2	4
5382	GO:0035674	tricarboxylic acid transmembrane transport	2	2
5383	GO:0035675	neuromast hair cell development	2	10
5384	GO:0035710	CD4-positive, alpha-beta T cell activation	2	7
5385	GO:0035720	intraflagellar anterograde transport	2	23
5386	GO:0035786	protein complex oligomerization	2	6
5387	GO:0035945	mitochondrial ncRNA surveillance	2	20
5388	GO:0035946	mitochondrial mRNA surveillance	2	20
5389	GO:0035998	7,8-dihydroneopterin 3'-triphosphate biosynthetic process	2	5
5390	GO:0036035	osteoclast development	2	30
5391	GO:0036124	histone H3-K9 trimethylation	2	18
5392	GO:0036148	phosphatidylglycerol acyl-chain remodeling	2	13
5393	GO:0036177	filamentous growth of a population of unicellular organisms in response to pH	2	19
5394	GO:0036187	cell growth mode switching, budding to filamentous	2	22
5395	GO:0036261	7-methylguanosine cap hypermethylation	2	47
5396	GO:0036265	RNA (guanine-N7)-methylation	2	12
5397	GO:0038127	ERBB signaling pathway	2	4
5398	GO:0039022	pronephric duct development	2	10
5399	GO:0039531	regulation of viral-induced cytoplasmic pattern recognition receptor signaling pathway	2	12
5400	GO:0040019	positive regulation of embryonic development	2	31
5401	GO:0040032	post-embryonic body morphogenesis	2	5
5402	GO:0042035	regulation of cytokine biosynthetic process	2	5
5403	GO:0042051	compound eye photoreceptor development	2	32
5404	GO:0042074	cell migration involved in gastrulation	2	9
5405	GO:0042116	macrophage activation	2	5
5406	GO:0042126	nitrate metabolic process	2	2
5407	GO:0042183	formate catabolic process	2	2
5408	GO:0042219	cellular modified amino acid catabolic process	2	15
5409	GO:0042262	DNA protection	2	4
5410	GO:0042352	GDP-L-fucose salvage	2	9
5411	GO:0042359	vitamin D metabolic process	2	11
5412	GO:0042425	choline biosynthetic process	2	8
5413	GO:0042455	ribonucleoside biosynthetic process	2	10
5414	GO:0042464	dosage compensation by hypoactivation of X chromosome	2	11
5415	GO:0042534	regulation of tumor necrosis factor biosynthetic process	2	4
5416	GO:0042537	benzene-containing compound metabolic process	2	3
5417	GO:0042549	photosystem II stabilization	2	3
5418	GO:0042660	positive regulation of cell fate specification	2	5
5419	GO:0042711	maternal behavior	2	15
5420	GO:0042775	mitochondrial ATP synthesis coupled electron transport	2	3
5421	GO:0042797	tRNA transcription from RNA polymerase III promoter	2	7
5422	GO:0042820	vitamin B6 catabolic process	2	10
5423	GO:0042821	pyridoxal biosynthetic process	2	12
5424	GO:0042832	defense response to protozoan	2	5
5425	GO:0042868	antisense RNA metabolic process	2	24
5426	GO:0042904	9-cis-retinoic acid biosynthetic process	2	5
5427	GO:0042906	xanthine transport	2	24
5428	GO:0042953	lipoprotein transport	2	14
5429	GO:0042985	negative regulation of amyloid precursor protein biosynthetic process	2	35
5430	GO:0043046	DNA methylation involved in gamete generation	2	3
5431	GO:0043054	dauer exit	2	2

5432	GO:0043060	meiotic metaphase I plate congression	2	23
5433	GO:0043101	purine-containing compound salvage	2	19
5434	GO:0043112	receptor metabolic process	2	8
5435	GO:0043114	regulation of vascular permeability	2	30
5436	GO:0043241	protein complex disassembly	2	11
5437	GO:0043282	pharyngeal muscle development	2	2
5438	GO:0043323	positive regulation of natural killer cell degranulation	2	26
5439	GO:0043408	regulation of MAPK cascade	2	16
5440	GO:0043448	alkane catabolic process	2	4
5441	GO:0043456	regulation of pentose-phosphate shunt	2	9
5442	GO:0043482	cellular pigment accumulation	2	19
5443	GO:0043537	negative regulation of blood vessel endothelial cell migration	2	19
5444	GO:0043550	regulation of lipid kinase activity	2	26
5445	GO:0043609	regulation of carbon utilization	2	24
5446	GO:0043697	cell dedifferentiation	2	10
5447	GO:0043900	regulation of multi-organism process	2	12
5448	GO:0043949	regulation of cAMP-mediated signaling	2	15
5449	GO:0043990	histone H2A-S1 phosphorylation	2	11
5450	GO:0044053	translocation of peptides or proteins into host cell cytoplasm	2	11
5451	GO:0044065	regulation of respiratory system process	2	6
5452	GO:0044130	negative regulation of growth of symbiont in host	2	7
5453	GO:0044208	'de novo' AMP biosynthetic process	2	3
5454	GO:0044265	cellular macromolecule catabolic process	2	9
5455	GO:0044282	small molecule catabolic process	2	3
5456	GO:0044313	protein K6-linked deubiquitination	2	13
5457	GO:0044319	wound healing, spreading of cells	2	9
5458	GO:0044342	type B pancreatic cell proliferation	2	4
5459	GO:0044396	actin cortical patch organization	2	8
5460	GO:0044409	entry into host	2	25
5461	GO:0045109	intermediate filament organization	2	9
5462	GO:0045122	aflatoxin biosynthetic process	2	2
5463	GO:0045143	homologous chromosome segregation	2	27
5464	GO:0045186	zonula adherens assembly	2	4
5465	GO:0045196	establishment or maintenance of neuroblast polarity	2	3
5466	GO:0045198	establishment of epithelial cell apical/basal polarity	2	4
5467	GO:0045227	capsule polysaccharide biosynthetic process	2	13
5468	GO:0045299	otolith mineralization	2	14
5469	GO:0045321	leukocyte activation	2	4
5470	GO:0045342	MHC class II biosynthetic process	2	5
5471	GO:0045433	male courtship behavior, veined wing generated song production	2	12
5472	GO:0045541	negative regulation of cholesterol biosynthetic process	2	3
5473	GO:0045570	regulation of imaginal disc growth	2	23
5474	GO:0045571	negative regulation of imaginal disc growth	2	4
5475	GO:0045577	regulation of B cell differentiation	2	3
5476	GO:0045616	regulation of keratinocyte differentiation	2	3
5477	GO:0045647	negative regulation of erythrocyte differentiation	2	3
5478	GO:0045657	positive regulation of monocyte differentiation	2	3
5479	GO:0045661	regulation of myoblast differentiation	2	3
5480	GO:0045694	regulation of embryo sac egg cell differentiation	2	4
5481	GO:0045733	acetate catabolic process	2	6
5482	GO:0045765	regulation of angiogenesis	2	5
5483	GO:0045823	positive regulation of heart contraction	2	8
5484	GO:0045835	negative regulation of meiosis	2	11
5485	GO:0045840	positive regulation of mitosis	2	2
5486	GO:0045857	negative regulation of molecular function, epigenetic	2	14
5487	GO:0045887	positive regulation of synaptic growth at neuromuscular junction	2	13

5488	GO:0045898	regulation of RNA polymerase II transcriptional preinitiation complex assembly	2	25
5489	GO:0045900	negative regulation of translational elongation	2	12
5490	GO:0045951	positive regulation of mitotic recombination	2	41
5491	GO:0046041	ITP metabolic process	2	3
5492	GO:0046051	UTP metabolic process	2	3
5493	GO:0046068	cGMP metabolic process	2	14
5494	GO:0046078	dUMP metabolic process	2	12
5495	GO:0046176	aldonic acid catabolic process	2	4
5496	GO:0046219	indolalkylamine biosynthetic process	2	11
5497	GO:0046293	formaldehyde biosynthetic process	2	17
5498	GO:0046294	formaldehyde catabolic process	2	5
5499	GO:0046338	phosphatidylethanolamine catabolic process	2	27
5500	GO:0046426	negative regulation of JAK-STAT cascade	2	13
5501	GO:0046436	D-alanine metabolic process	2	13
5502	GO:0046477	glycosylceramide catabolic process	2	26
5503	GO:0046496	nicotinamide nucleotide metabolic process	2	11
5504	GO:0046548	retinal rod cell development	2	23
5505	GO:0046578	regulation of Ras protein signal transduction	2	14
5506	GO:0046594	maintenance of pole plasm mRNA location	2	3
5507	GO:0046638	positive regulation of alpha-beta T cell differentiation	2	2
5508	GO:0046664	dorsal closure, amnioserosa morphology change	2	2
5509	GO:0046667	compound eye retinal cell programmed cell death	2	13
5510	GO:0046689	response to mercury ion	2	4
5511	GO:0046732	active induction of host immune response by virus	2	8
5512	GO:0046739	spread of virus in host	2	2
5513	GO:0046838	phosphorylated carbohydrate dephosphorylation	2	9
5514	GO:0046865	terpenoid transport	2	14
5515	GO:0046928	regulation of neurotransmitter secretion	2	2
5516	GO:0046931	pore complex assembly	2	16
5517	GO:0046957	negative phototaxis	2	12
5518	GO:0046963	3'-phosphoadenosine 5'-phosphosulfate transport	2	6
5519	GO:0046985	positive regulation of hemoglobin biosynthetic process	2	10
5520	GO:0048009	insulin-like growth factor receptor signaling pathway	2	17
5521	GO:0048106	establishment of thoracic bristle planar orientation	2	32
5522	GO:0048205	COPI coating of Golgi vesicle	2	7
5523	GO:0048260	positive regulation of receptor-mediated endocytosis	2	7
5524	GO:0048382	mesendoderm development	2	3
5525	GO:0048384	retinoic acid receptor signaling pathway	2	3
5526	GO:0048386	positive regulation of retinoic acid receptor signaling pathway	2	4
5527	GO:0048448	stamen morphogenesis	2	3
5528	GO:0048505	regulation of timing of cell differentiation	2	12
5529	GO:0048538	thymus development	2	23
5530	GO:0048539	bone marrow development	2	6
5531	GO:0048581	negative regulation of post-embryonic development	2	10
5532	GO:0048599	oocyte development	2	33
5533	GO:0048662	negative regulation of smooth muscle cell proliferation	2	3
5534	GO:0048665	neuron fate specification	2	9
5535	GO:0048729	tissue morphogenesis	2	28
5536	GO:0048745	smooth muscle tissue development	2	6
5537	GO:0048853	forebrain morphogenesis	2	15
5538	GO:0048870	cell motility	2	11
5539	GO:0048872	homeostasis of number of cells	2	20
5540	GO:0050435	beta-amyloid metabolic process	2	10
5541	GO:0050650	chondroitin sulfate proteoglycan biosynthetic process	2	7
5542	GO:0050770	regulation of axonogenesis	2	2
5543	GO:0050772	positive regulation of axonogenesis	2	23
5544	GO:0050803	regulation of synapse structure and activity	2	4
5545	GO:0050806	positive regulation of synaptic transmission	2	2

5546	GO:0050851	antigen receptor-mediated signaling pathway	2	8
5547	GO:0050870	positive regulation of T cell activation	2	9
5548	GO:0050872	white fat cell differentiation	2	15
5549	GO:0050910	detection of mechanical stimulus involved in sensory perception of sound	2	14
5550	GO:0050965	detection of temperature stimulus involved in sensory perception of pain	2	2
5551	GO:0050992	dimethylallyl diphosphate biosynthetic process	2	3
5552	GO:0050995	negative regulation of lipid catabolic process	2	29
5553	GO:0051047	positive regulation of secretion	2	22
5554	GO:0051068	dihydrolipoamide metabolic process	2	3
5555	GO:0051148	negative regulation of muscle cell differentiation	2	23
5556	GO:0051153	regulation of striated muscle cell differentiation	2	7
5557	GO:0051154	negative regulation of striated muscle cell differentiation	2	2
5558	GO:0051177	meiotic sister chromatid cohesion	2	10
5559	GO:0051204	protein insertion into mitochondrial membrane	2	11
5560	GO:0051222	positive regulation of protein transport	2	15
5561	GO:0051229	meiotic spindle disassembly	2	15
5562	GO:0051255	spindle midzone assembly	2	6
5563	GO:0051257	spindle midzone assembly involved in meiosis	2	23
5564	GO:0051281	positive regulation of release of sequestered calcium ion into cytosol	2	10
5565	GO:0051310	metaphase plate congression	2	2
5566	GO:0051383	kinetochore organization	2	16
5567	GO:0051409	response to nitrosative stress	2	14
5568	GO:0051417	microtubule nucleation by spindle pole body	2	17
5569	GO:0051495	positive regulation of cytoskeleton organization	2	3
5570	GO:0051597	response to methylmercury	2	12
5571	GO:0051666	actin cortical patch localization	2	7
5572	GO:0051702	interaction with symbiont	2	37
5573	GO:0051783	regulation of nuclear division	2	19
5574	GO:0051962	positive regulation of nervous system development	2	9
5575	GO:0051987	positive regulation of attachment of spindle microtubules to kinetochore	2	12
5576	GO:0052652	cyclic purine nucleotide metabolic process	2	19
5577	GO:0052837	thiazole biosynthetic process	2	2
5578	GO:0055047	generative cell mitosis	2	23
5579	GO:0060024	rhythmic synaptic transmission	2	15
5580	GO:0060042	retina morphogenesis in camera-type eye	2	18
5581	GO:0060088	auditory receptor cell stereocilium organization	2	14
5582	GO:0060134	prepulse inhibition	2	15
5583	GO:0060142	regulation of syncytium formation by plasma membrane fusion	2	8
5584	GO:0060161	positive regulation of dopamine receptor signaling pathway	2	22
5585	GO:0060179	male mating behavior	2	15
5586	GO:0060211	regulation of nuclear-transcribed mRNA poly(A) tail shortening	2	2
5587	GO:0060215	primitive hemopoiesis	2	11
5588	GO:0060244	negative regulation of cell proliferation involved in contact inhibition	2	2
5589	GO:0060249	anatomical structure homeostasis	2	3
5590	GO:0060325	face morphogenesis	2	32
5591	GO:0060337	type I interferon-mediated signaling pathway	2	3
5592	GO:0060340	positive regulation of type I interferon-mediated signaling pathway	2	9
5593	GO:0060368	regulation of Fc receptor mediated stimulatory signaling pathway	2	16
5594	GO:0060378	regulation of brood size	2	13
5595	GO:0060440	trachea formation	2	23
5596	GO:0060448	dichotomous subdivision of terminal units involved in lung branching	2	3

5597	GO:0060502	epithelial cell proliferation involved in lung morphogenesis	2	23
5598	GO:0060566	positive regulation of DNA-dependent transcription, termination	2	18
5599	GO:0060603	mammary gland duct morphogenesis	2	15
5600	GO:0060626	regulation of cullin deneddylation	2	12
5601	GO:0060712	spongiotrophoblast layer development	2	3
5602	GO:0060715	syncytiotrophoblast cell differentiation involved in labyrinthine layer development	2	9
5603	GO:0060717	chorion development	2	9
5604	GO:0060736	prostate gland growth	2	15
5605	GO:0060760	positive regulation of response to cytokine stimulus	2	2
5606	GO:0060772	leaf phyllotactic patterning	2	8
5607	GO:0060785	regulation of apoptosis involved in tissue homeostasis	2	8
5608	GO:0060867	fruit abscission	2	2
5609	GO:0060872	semicircular canal development	2	18
5610	GO:0060999	positive regulation of dendritic spine development	2	22
5611	GO:0061001	regulation of dendritic spine morphogenesis	2	9
5612	GO:0061031	endodermal digestive tract morphogenesis	2	21
5613	GO:0061052	negative regulation of cell growth involved in cardiac muscle cell development	2	8
5614	GO:0061063	positive regulation of nematode larval development	2	13
5615	GO:0061099	negative regulation of protein tyrosine kinase activity	2	2
5616	GO:0061113	pancreas morphogenesis	2	21
5617	GO:0061154	endothelial tube morphogenesis	2	19
5618	GO:0061162	establishment of monopolar cell polarity	2	7
5619	GO:0061180	mammary gland epithelium development	2	17
5620	GO:0061331	epithelial cell proliferation involved in Malpighian tubule morphogenesis	2	12
5621	GO:0061387	regulation of extent of cell growth	2	19
5622	GO:0061388	regulation of rate of cell growth	2	14
5623	GO:0070086	ubiquitin-dependent endocytosis	2	2
5624	GO:0070124	mitochondrial translational initiation	2	5
5625	GO:0070129	regulation of mitochondrial translation	2	6
5626	GO:0070143	mitochondrial alanyl-tRNA aminoacylation	2	10
5627	GO:0070221	sulfide oxidation, using sulfide:quinone oxidoreductase	2	9
5628	GO:0070257	positive regulation of mucus secretion	2	21
5629	GO:0070407	oxidation-dependent protein catabolic process	2	29
5630	GO:0070434	positive regulation of nucleotide-binding oligomerization domain containing 2 signaling pathway	2	2
5631	GO:0070475	rRNA base methylation	2	14
5632	GO:0070544	histone H3-K36 demethylation	2	9
5633	GO:0070672	response to interleukin-15	2	11
5634	GO:0070727	cellular macromolecule localization	2	9
5635	GO:0070813	hydrogen sulfide metabolic process	2	9
5636	GO:0070838	divalent metal ion transport	2	2
5637	GO:0070839	divalent metal ion export	2	5
5638	GO:0070903	mitochondrial tRNA thio-modification	2	4
5639	GO:0070973	protein localization to endoplasmic reticulum exit site	2	7
5640	GO:0070983	dendrite guidance	2	17
5641	GO:0070986	left/right axis specification	2	10
5642	GO:0071036	nuclear polyadenylation-dependent snoRNA catabolic process	2	8
5643	GO:0071037	nuclear polyadenylation-dependent snRNA catabolic process	2	8
5644	GO:0071040	nuclear polyadenylation-dependent antisense transcript catabolic process	2	8
5645	GO:0071046	nuclear polyadenylation-dependent ncRNA catabolic process	2	14
5646	GO:0071076	RNA 3' uridylation	2	18
5647	GO:0071107	response to parathyroid hormone stimulus	2	4

5648	GO:0071139	resolution of recombination intermediates	2	18
5649	GO:0071140	resolution of mitotic recombination intermediates	2	10
5650	GO:0071166	ribonucleoprotein complex localization	2	13
5651	GO:0071167	ribonucleoprotein complex import into nucleus	2	47
5652	GO:0071211	protein targeting to vacuole involved in autophagy	2	8
5653	GO:0071242	cellular response to ammonium ion	2	23
5654	GO:0071267	L-methionine salvage	2	3
5655	GO:0071291	cellular response to selenium ion	2	9
5656	GO:0071292	cellular response to silver ion	2	12
5657	GO:0071315	cellular response to morphine	2	21
5658	GO:0071325	cellular response to mannitol stimulus	2	3
5659	GO:0071327	cellular response to trehalose stimulus	2	29
5660	GO:0071393	cellular response to progesterone stimulus	2	30
5661	GO:0071425	hemopoietic stem cell proliferation	2	2
5662	GO:0071453	cellular response to oxygen levels	2	12
5663	GO:0071467	cellular response to pH	2	19
5664	GO:0071490	cellular response to far red light	2	11
5665	GO:0071491	cellular response to red light	2	11
5666	GO:0071494	cellular response to UV-C	2	38
5667	GO:0071500	cellular response to nitrosative stress	2	50
5668	GO:0071526	semaphorin-plexin signaling pathway	2	3
5669	GO:0071528	tRNA re-export from nucleus	2	39
5670	GO:0071542	dopaminergic neuron differentiation	2	3
5671	GO:0071602	phytosphingosine biosynthetic process	2	11
5672	GO:0071625	vocalization behavior	2	20
5673	GO:0071638	negative regulation of monocyte chemotactic protein-1 production	2	3
5674	GO:0071674	mononuclear cell migration	2	3
5675	GO:0071879	positive regulation of adrenergic receptor signaling pathway	2	8
5676	GO:0071962	mitotic sister chromatid cohesion, centromeric	2	3
5677	GO:0072015	glomerular visceral epithelial cell development	2	19
5678	GO:0072091	regulation of stem cell proliferation	2	3
5679	GO:0072319	vesicle uncoating	2	4
5680	GO:0072348	sulfur compound transport	2	6
5681	GO:0072362	regulation of glycolysis by negative regulation of transcription from RNA polymerase II promoter	2	36
5682	GO:0072365	regulation of cellular ketone metabolic process by negative regulation of transcription from RNA polymerase II promoter	2	36
5683	GO:0072368	regulation of lipid transport by negative regulation of transcription from RNA polymerase II promoter	2	36
5684	GO:0072422	signal transduction involved in DNA damage checkpoint	2	5
5685	GO:0072434	signal transduction involved in mitotic G2/M transition DNA damage checkpoint	2	50
5686	GO:0072503	cellular divalent inorganic cation homeostasis	2	17
5687	GO:0072507	divalent inorganic cation homeostasis	2	6
5688	GO:0072528	pyrimidine-containing compound biosynthetic process	2	7
5689	GO:0072553	terminal button organization	2	29
5690	GO:0072554	blood vessel lumenization	2	11
5691	GO:0072577	endothelial cell apoptotic process	2	22
5692	GO:0072709	cellular response to sorbitol	2	3
5693	GO:0072715	cellular response to selenite ion	2	6
5694	GO:0072719	cellular response to cisplatin	2	10
5695	GO:0080005	photosystem stoichiometry adjustment	2	56
5696	GO:0080143	regulation of amino acid export	2	2
5697	GO:0080145	cysteine homeostasis	2	14
5698	GO:0080158	chloroplast ribulose bisphosphate carboxylase complex biogenesis	2	2
5699	GO:0086001	regulation of cardiac muscle cell action potential	2	6
5700	GO:0086003	cardiac muscle cell contraction	2	6

5701	GO:0086014	regulation of atrial cardiac muscle cell action potential	2	5
5702	GO:0086070	SA node cardiomyocyte to atrial cardiomyocyte communication	2	6
5703	GO:0086098	angiotensin-mediated signaling pathway involved in heart process	2	30
5704	GO:0090036	regulation of protein kinase C signaling cascade	2	13
5705	GO:0090050	positive regulation of cell migration involved in sprouting angiogenesis	2	14
5706	GO:0090054	regulation of chromatin silencing at silent mating-type cassette	2	3
5707	GO:0090056	regulation of chlorophyll metabolic process	2	25
5708	GO:0090063	positive regulation of microtubule nucleation	2	29
5709	GO:0090070	positive regulation of ribosome biogenesis	2	4
5710	GO:0090086	negative regulation of protein deubiquitination	2	3
5711	GO:0090087	regulation of peptide transport	2	11
5712	GO:0090102	cochlea development	2	14
5713	GO:0090116	C-5 methylation of cytosine	2	3
5714	GO:0090143	nucleoid organization	2	3
5715	GO:0090149	membrane fission involved in mitochondrial fission	2	10
5716	GO:0090161	Golgi ribbon formation	2	6
5717	GO:0090228	positive regulation of red or far-red light signaling pathway	2	8
5718	GO:0090233	negative regulation of spindle checkpoint	2	6
5719	GO:0090244	Wnt receptor signaling pathway involved in somitogenesis	2	6
5720	GO:0090249	regulation of cell motility involved in somitogenic axis elongation	2	6
5721	GO:0090267	positive regulation of mitotic cell cycle spindle assembly checkpoint	2	10
5722	GO:0090296	regulation of mitochondrial DNA replication	2	29
5723	GO:0090298	negative regulation of mitochondrial DNA replication	2	21
5724	GO:0090312	positive regulation of protein deacetylation	2	21
5725	GO:0090325	regulation of locomotion involved in locomotory behavior	2	4
5726	GO:0090391	granum assembly	2	2
5727	GO:0090392	sepal giant cell differentiation	2	46
5728	GO:0090399	replicative senescence	2	50
5729	GO:0090435	protein localization in nuclear envelope	2	4
5730	GO:0097017	renal protein absorption	2	23
5731	GO:0097033	mitochondrial respiratory chain complex III biogenesis	2	11
5732	GO:0097052	L-kynurenine metabolic process	2	5
5733	GO:0097056	selenocysteinyl-tRNA(Sec) biosynthetic process	2	11
5734	GO:0097067	cellular response to thyroid hormone stimulus	2	3
5735	GO:0097105	presynaptic membrane assembly	2	15
5736	GO:0097107	postsynaptic density assembly	2	15
5737	GO:0097111	endoplasmic reticulum-Golgi intermediate compartment organization	2	7
5738	GO:0097186	amelogenesis	2	7
5739	GO:0097264	self proteolysis	2	46
5740	GO:0097305	response to alcohol	2	2
5741	GO:0097306	cellular response to alcohol	2	9
5742	GO:0097325	melanocyte proliferation	2	2
5743	GO:0097345	mitochondrial outer membrane permeabilization	2	13
5744	GO:1900006	positive regulation of dendrite development	2	3
5745	GO:1900018	phosphorylation of RNA polymerase II C-terminal domain serine 5 residues involved in recruitment of mRNA capping enzyme to RNA polymerase II holoenzyme complex	2	5
5746	GO:1900037	regulation of cellular response to hypoxia	2	19
5747	GO:1900063	regulation of peroxisome organization	2	10
5748	GO:1900119	positive regulation of execution phase of apoptosis	2	2
5749	GO:1900210	positive regulation of cardiolipin metabolic process	2	7
5750	GO:1900223	positive regulation of beta-amyloid clearance	2	35

5751	GO:1900245	positive regulation of MDA-5 signaling pathway	2	18
5752	GO:1900367	positive regulation of defense response to insect	2	29
5753	GO:1900429	negative regulation of filamentous growth of a population of unicellular organisms	2	6
5754	GO:1900490	positive regulation of hydroxymethylglutaryl-CoA reductase (NADPH) activity	2	4
5755	GO:1900570	diorcinol metabolic process	2	10
5756	GO:1900588	violaceol I metabolic process	2	10
5757	GO:1900591	violaceol II metabolic process	2	10
5758	GO:1900797	cordyol C metabolic process	2	10
5759	GO:1900871	chloroplast mRNA modification	2	2
5760	GO:1901076	positive regulation of engulfment of apoptotic cell	2	35
5761	GO:1901094	negative regulation of protein homotetramerization	2	13
5762	GO:1901096	regulation of autophagic vacuole maturation	2	7
5763	GO:1901185	negative regulation of ERBB signaling pathway	2	11
5764	GO:2000009	negative regulation of protein localization at cell surface	2	6
5765	GO:2000029	regulation of proanthocyanidin biosynthetic process	2	6
5766	GO:2000082	regulation of L-ascorbic acid biosynthetic process	2	2
5767	GO:2000098	negative regulation of smooth muscle cell-matrix adhesion	2	3
5768	GO:2000109	regulation of macrophage apoptotic process	2	11
5769	GO:2000117	negative regulation of cysteine-type endopeptidase activity	2	2
5770	GO:2000147	positive regulation of cell motility	2	23
5771	GO:2000234	positive regulation of rRNA processing	2	22
5772	GO:2000242	negative regulation of reproductive process	2	10
5773	GO:2000273	positive regulation of receptor activity	2	15
5774	GO:2000288	positive regulation of myoblast proliferation	2	7
5775	GO:2000397	positive regulation of ubiquitin-dependent endocytosis	2	7
5776	GO:2000405	negative regulation of T cell migration	2	3
5777	GO:2000435	negative regulation of protein neddylation	2	17
5778	GO:2000467	positive regulation of glycogen (starch) synthase activity	2	8
5779	GO:2000483	negative regulation of interleukin-8 secretion	2	7
5780	GO:2000619	negative regulation of histone H4-K16 acetylation	2	21
5781	GO:2000685	positive regulation of cellular response to X-ray	2	33
5782	GO:2000692	negative regulation of seed maturation	2	2
5783	GO:2000694	regulation of phragmoplast microtubule organization	2	18
5784	GO:2000726	negative regulation of cardiac muscle cell differentiation	2	4
5785	GO:2000736	regulation of stem cell differentiation	2	15
5786	GO:2000780	negative regulation of double-strand break repair	2	7
5787	GO:2000808	negative regulation of synaptic vesicle clustering	2	15
5788	GO:2001023	regulation of response to drug	2	12
5789	GO:2001057	reactive nitrogen species metabolic process	2	8
5790	GO:2001142	nicotinate transport	2	21
5791	GO:2001143	N-methylnicotinate transport	2	21
5792	GO:2001170	negative regulation of ATP biosynthetic process	2	14
5793	GO:2001233	regulation of apoptotic signaling pathway	2	7
5794	GO:2001235	positive regulation of apoptotic signaling pathway	2	3
5795	GO:2001251	negative regulation of chromosome organization	2	9
5796	GO:2001269	positive regulation of cysteine-type endopeptidase activity involved in apoptotic signaling pathway	2	23
5797	GO:0000096	sulfur amino acid metabolic process	1	4
5798	GO:0000098	sulfur amino acid catabolic process	1	6
5799	GO:0000147	actin cortical patch assembly	1	4
5800	GO:0000173	inactivation of MAPK activity involved in osmosensory signaling pathway	1	7
5801	GO:0000338	protein deneddylation	1	1
5802	GO:0000429	carbon catabolite regulation of transcription from RNA polymerase II promoter	1	13

5803	GO:0000448	cleavage in ITS2 between 5.8S rRNA and LSU-rRNA of tricistronic rRNA transcript (SSU-rRNA, 5.8S rRNA, LSU-rRNA)	1	7
5804	GO:0000453	enzyme-directed rRNA 2'-O-methylation	1	8
5805	GO:0000454	snoRNA guided rRNA pseudouridine synthesis	1	2
5806	GO:0000478	endonucleolytic cleavage involved in rRNA processing	1	7
5807	GO:0000481	maturation of 5S rRNA	1	4
5808	GO:0000707	meiotic DNA recombinase assembly	1	3
5809	GO:0000716	transcription-coupled nucleotide-excision repair, DNA damage recognition	1	1
5810	GO:0000720	pyrimidine dimer repair by nucleotide-excision repair	1	13
5811	GO:0000736	double-strand break repair via single-strand annealing, removal of nonhomologous ends	1	19
5812	GO:0000740	nuclear membrane fusion	1	2
5813	GO:0000750	pheromone-dependent signal transduction involved in conjugation with cellular fusion	1	20
5814	GO:0000754	adaptation of signaling pathway by response to pheromone involved in conjugation with cellular fusion	1	2
5815	GO:0000905	sporocarp development involved in asexual reproduction	1	17
5816	GO:0000916	actomyosin contractile ring contraction	1	1
5817	GO:0000964	mitochondrial RNA 5'-end processing	1	7
5818	GO:0001111	promoter clearance from RNA polymerase II promoter	1	11
5819	GO:0001113	transcriptional open complex formation at RNA polymerase II promoter	1	11
5820	GO:0001189	RNA polymerase I transcriptional preinitiation complex assembly at the promoter for the nuclear large rRNA transcript	1	7
5821	GO:0001516	prostaglandin biosynthetic process	1	26
5822	GO:0001574	ganglioside biosynthetic process	1	11
5823	GO:0001655	urogenital system development	1	6
5824	GO:0001672	regulation of chromatin assembly or disassembly	1	9
5825	GO:0001680	tRNA 3'-terminal CCA addition	1	16
5826	GO:0001694	histamine biosynthetic process	1	1
5827	GO:0001742	oenocyte differentiation	1	2
5828	GO:0001752	compound eye photoreceptor fate commitment	1	2
5829	GO:0001775	cell activation	1	6
5830	GO:0001780	neutrophil homeostasis	1	3
5831	GO:0001823	mesonephros development	1	9
5832	GO:0001826	inner cell mass cell differentiation	1	14
5833	GO:0001841	neural tube formation	1	10
5834	GO:0001895	retina homeostasis	1	1
5835	GO:0001919	regulation of receptor recycling	1	2
5836	GO:0001935	endothelial cell proliferation	1	2
5837	GO:0001936	regulation of endothelial cell proliferation	1	2
5838	GO:0001941	postsynaptic membrane organization	1	13
5839	GO:0001946	lymphangiogenesis	1	1
5840	GO:0001949	sebaceous gland cell differentiation	1	4
5841	GO:0001956	positive regulation of neurotransmitter secretion	1	11
5842	GO:0001966	thigmotaxis	1	1
5843	GO:0002005	angiotensin catabolic process in blood	1	1
5844	GO:0002011	morphogenesis of an epithelial sheet	1	3
5845	GO:0002018	renin-angiotensin regulation of aldosterone production	1	9
5846	GO:0002028	regulation of sodium ion transport	1	6
5847	GO:0002029	desensitization of G-protein coupled receptor protein signaling pathway	1	8
5848	GO:0002062	chondrocyte differentiation	1	2
5849	GO:0002063	chondrocyte development	1	2
5850	GO:0002070	epithelial cell maturation	1	3
5851	GO:0002100	tRNA wobble adenosine to inosine editing	1	3
5852	GO:0002101	tRNA wobble cytosine modification	1	6

5853	GO:0002103	endonucleolytic cleavage of tetracistronic rRNA transcript (SSU-rRNA, LSU-rRNA, 4.5S-rRNA, 5S-rRNA)	1	2
5854	GO:0002107	generation of mature 3'-end of 5S rRNA generated by RNA polymerase III	1	5
5855	GO:0002128	tRNA nucleoside ribose methylation	1	3
5856	GO:0002131	wobble position cytosine ribose methylation	1	9
5857	GO:0002132	wobble position uridine ribose methylation	1	9
5858	GO:0002204	somatic recombination of immunoglobulin genes involved in immune response	1	1
5859	GO:0002206	gene conversion of immunoglobulin genes	1	15
5860	GO:0002236	detection of misfolded protein	1	2
5861	GO:0002250	adaptive immune response	1	6
5862	GO:0002253	activation of immune response	1	8
5863	GO:0002260	lymphocyte homeostasis	1	2
5864	GO:0002281	macrophage activation involved in immune response	1	8
5865	GO:0002331	pre-B cell allelic exclusion	1	32
5866	GO:0002368	B cell cytokine production	1	3
5867	GO:0002446	neutrophil mediated immunity	1	2
5868	GO:0002537	nitric oxide production involved in inflammatory response	1	7
5869	GO:0002587	negative regulation of antigen processing and presentation of peptide antigen via MHC class II	1	1
5870	GO:0002686	negative regulation of leukocyte migration	1	2
5871	GO:0002756	MyD88-independent toll-like receptor signaling pathway	1	1
5872	GO:0002757	immune response-activating signal transduction	1	1
5873	GO:0002805	regulation of antimicrobial peptide biosynthetic process	1	1
5874	GO:0002821	positive regulation of adaptive immune response	1	2
5875	GO:0002842	positive regulation of T cell mediated immune response to tumor cell	1	3
5876	GO:0002903	negative regulation of B cell apoptotic process	1	6
5877	GO:0003013	circulatory system process	1	14
5878	GO:0003085	negative regulation of systemic arterial blood pressure	1	6
5879	GO:0003093	regulation of glomerular filtration	1	7
5880	GO:0003131	mesodermal-endodermal cell signaling	1	12
5881	GO:0003156	regulation of organ formation	1	2
5882	GO:0003157	endocardium development	1	10
5883	GO:0003254	regulation of membrane depolarization	1	5
5884	GO:0003279	cardiac septum development	1	1
5885	GO:0003323	type B pancreatic cell development	1	3
5886	GO:0003338	metanephros morphogenesis	1	2
5887	GO:0003341	cilium movement	1	7
5888	GO:0003382	epithelial cell morphogenesis	1	3
5889	GO:0003384	apical constriction involved in gastrulation	1	2
5890	GO:0005990	lactose catabolic process	1	3
5891	GO:0005991	trehalose metabolic process	1	1
5892	GO:0006003	fructose 2,6-bisphosphate metabolic process	1	19
5893	GO:0006064	glucuronate catabolic process	1	9
5894	GO:0006067	ethanol metabolic process	1	4
5895	GO:0006068	ethanol catabolic process	1	1
5896	GO:0006072	glycerol-3-phosphate metabolic process	1	1
5897	GO:0006089	lactate metabolic process	1	2
5898	GO:0006106	fumarate metabolic process	1	4
5899	GO:0006111	regulation of gluconeogenesis	1	12
5900	GO:0006176	dATP biosynthetic process from ADP	1	1
5901	GO:0006177	GMP biosynthetic process	1	1
5902	GO:0006195	purine nucleotide catabolic process	1	2
5903	GO:0006230	TMP biosynthetic process	1	1
5904	GO:0006266	DNA ligation	1	1
5905	GO:0006277	DNA amplification	1	19

5906	GO:0006279	premeiotic DNA replication	1	14
5907	GO:0006311	meiotic gene conversion	1	10
5908	GO:0006343	establishment of chromatin silencing	1	1
5909	GO:0006359	regulation of transcription from RNA polymerase III promoter	1	9
5910	GO:0006384	transcription initiation from RNA polymerase III promoter	1	9
5911	GO:0006386	termination of RNA polymerase III transcription	1	1
5912	GO:0006398	histone mRNA 3'-end processing	1	2
5913	GO:0006408	snRNA export from nucleus	1	2
5914	GO:0006420	arginyl-tRNA aminoacylation	1	7
5915	GO:0006423	cysteinyl-tRNA aminoacylation	1	1
5916	GO:0006481	C-terminal protein methylation	1	24
5917	GO:0006489	dolichyl diphosphate biosynthetic process	1	3
5918	GO:0006505	GPI anchor metabolic process	1	3
5919	GO:0006524	alanine catabolic process	1	1
5920	GO:0006548	histidine catabolic process	1	1
5921	GO:0006549	isoleucine metabolic process	1	1
5922	GO:0006562	proline catabolic process	1	3
5923	GO:0006580	ethanolamine metabolic process	1	4
5924	GO:0006584	catecholamine metabolic process	1	3
5925	GO:0006589	octopamine biosynthetic process	1	1
5926	GO:0006590	thyroid hormone generation	1	1
5927	GO:0006592	ornithine biosynthetic process	1	4
5928	GO:0006593	ornithine catabolic process	1	8
5929	GO:0006600	creatine metabolic process	1	1
5930	GO:0006613	cotranslational protein targeting to membrane	1	1
5931	GO:0006663	platelet activating factor biosynthetic process	1	6
5932	GO:0006678	glucosylceramide metabolic process	1	4
5933	GO:0006681	galactosylceramide metabolic process	1	4
5934	GO:0006686	sphingomyelin biosynthetic process	1	7
5935	GO:0006718	juvenile hormone biosynthetic process	1	2
5936	GO:0006747	FAD biosynthetic process	1	7
5937	GO:0006766	vitamin metabolic process	1	1
5938	GO:0006784	heme a biosynthetic process	1	5
5939	GO:0006791	sulfur utilization	1	1
5940	GO:0006850	mitochondrial pyruvate transport	1	2
5941	GO:0006853	carnitine shuttle	1	9
5942	GO:0006900	membrane budding	1	1
5943	GO:0006907	pinocytosis	1	8
5944	GO:0006933	negative regulation of cell adhesion involved in substrate-bound cell migration	1	1
5945	GO:0006942	regulation of striated muscle contraction	1	4
5946	GO:0006956	complement activation	1	8
5947	GO:0006999	nuclear pore organization	1	4
5948	GO:0007039	vacuolar protein catabolic process	1	2
5949	GO:0007043	cell-cell junction assembly	1	1
5950	GO:0007069	negative regulation of transcription from RNA polymerase I promoter during mitosis	1	2
5951	GO:0007113	endomitotic cell cycle	1	2
5952	GO:0007119	budding cell isotropic bud growth	1	1
5953	GO:0007135	meiosis II	1	10
5954	GO:0007142	male meiosis II	1	3
5955	GO:0007162	negative regulation of cell adhesion	1	7
5956	GO:0007193	adenylate cyclase-inhibiting G-protein coupled receptor signaling pathway	1	6
5957	GO:0007202	activation of phospholipase C activity	1	10
5958	GO:0007204	elevation of cytosolic calcium ion concentration	1	1
5959	GO:0007340	acrosome reaction	1	1
5960	GO:0007343	egg activation	1	1

5961	GO:0007349	cellularization	1	1
5962	GO:0007377	germ-band extension	1	2
5963	GO:0007396	suture of dorsal opening	1	1
5964	GO:0007421	stomatogastric nervous system development	1	2
5965	GO:0007424	open tracheal system development	1	1
5966	GO:0007426	tracheal outgrowth, open tracheal system	1	1
5967	GO:0007438	oenocyte development	1	4
5968	GO:0007446	imaginal disc growth	1	11
5969	GO:0007447	imaginal disc pattern formation	1	34
5970	GO:0007458	progression of morphogenetic furrow involved in compound eye morphogenesis	1	2
5971	GO:0007469	antennal development	1	2
5972	GO:0007473	wing disc proximal/distal pattern formation	1	2
5973	GO:0007475	apposition of dorsal and ventral imaginal disc-derived wing surfaces	1	4
5974	GO:0007488	histoblast morphogenesis	1	2
5975	GO:0007521	muscle cell fate determination	1	4
5976	GO:0007538	primary sex determination	1	3
5977	GO:0007539	primary sex determination, soma	1	2
5978	GO:0007549	dosage compensation	1	1
5979	GO:0007563	regulation of eclosion	1	7
5980	GO:0007586	digestion	1	1
5981	GO:0007589	body fluid secretion	1	7
5982	GO:0007591	molting cycle, chitin-based cuticle	1	42
5983	GO:0007597	blood coagulation, intrinsic pathway	1	6
5984	GO:0007599	hemostasis	1	6
5985	GO:0007614	short-term memory	1	4
5986	GO:0007635	chemosensory behavior	1	5
5987	GO:0008064	regulation of actin polymerization or depolymerization	1	6
5988	GO:0008065	establishment of blood-nerve barrier	1	9
5989	GO:0008078	mesodermal cell migration	1	4
5990	GO:0008088	axon cargo transport	1	3
5991	GO:0008204	ergosterol metabolic process	1	15
5992	GO:0008205	ecdysone metabolic process	1	2
5993	GO:0008215	spermine metabolic process	1	1
5994	GO:0008354	germ cell migration	1	1
5995	GO:0008366	axon ensheathment	1	2
5996	GO:0008582	regulation of synaptic growth at neuromuscular junction	1	1
5997	GO:0008595	anterior/posterior axis specification, embryo	1	4
5998	GO:0009048	dosage compensation by inactivation of X chromosome	1	11
5999	GO:0009064	glutamine family amino acid metabolic process	1	2
6000	GO:0009072	aromatic amino acid family metabolic process	1	1
6001	GO:0009084	glutamine family amino acid biosynthetic process	1	1
6002	GO:0009120	deoxyribonucleoside metabolic process	1	3
6003	GO:0009139	pyrimidine nucleoside diphosphate biosynthetic process	1	5
6004	GO:0009141	nucleoside triphosphate metabolic process	1	5
6005	GO:0009151	purine deoxyribonucleotide metabolic process	1	5
6006	GO:0009154	purine ribonucleotide catabolic process	1	1
6007	GO:0009157	deoxyribonucleoside monophosphate biosynthetic process	1	1
6008	GO:0009166	nucleotide catabolic process	1	1
6009	GO:0009201	ribonucleoside triphosphate biosynthetic process	1	5
6010	GO:0009239	enterobactin biosynthetic process	1	8
6011	GO:0009244	lipopolysaccharide core region biosynthetic process	1	4
6012	GO:0009256	10-formyltetrahydrofolate metabolic process	1	5
6013	GO:0009260	ribonucleotide biosynthetic process	1	5
6014	GO:0009264	deoxyribonucleotide catabolic process	1	1
6015	GO:0009301	snRNA transcription	1	7
6016	GO:0009302	snoRNA transcription	1	4
6017	GO:0009413	response to flooding	1	8

6018	GO:0009440	cyanate catabolic process	1	2
6019	GO:0009450	gamma-aminobutyric acid catabolic process	1	4
6020	GO:0009594	detection of nutrient	1	2
6021	GO:0009661	chromoplast organization	1	5
6022	GO:0009760	C4 photosynthesis	1	1
6023	GO:0009794	regulation of mitotic cell cycle, embryonic	1	2
6024	GO:0009822	alkaloid catabolic process	1	1
6025	GO:0009868	jasmonic acid and ethylene-dependent systemic resistance, jasmonic acid mediated signaling pathway	1	8
6026	GO:0010001	glial cell differentiation	1	2
6027	GO:0010081	regulation of inflorescence meristem growth	1	3
6028	GO:0010101	post-embryonic root morphogenesis	1	5
6029	GO:0010121	arginine catabolic process to proline via ornithine	1	8
6030	GO:0010142	farnesyl diphosphate biosynthetic process, mevalonate pathway	1	3
6031	GO:0010213	non-photoreactive DNA repair	1	14
6032	GO:0010220	positive regulation of vernalization response	1	1
6033	GO:0010241	ent-kaurene oxidation to kaurenoic acid	1	3
6034	GO:0010299	detoxification of cobalt ion	1	3
6035	GO:0010343	singlet oxygen-mediated programmed cell death	1	2
6036	GO:0010352	lithium ion export	1	21
6037	GO:0010360	negative regulation of anion channel activity	1	2
6038	GO:0010393	galacturonan metabolic process	1	1
6039	GO:0010412	mannan metabolic process	1	1
6040	GO:0010463	mesenchymal cell proliferation	1	2
6041	GO:0010477	response to sulfur dioxide	1	4
6042	GO:0010509	polyamine homeostasis	1	4
6043	GO:0010520	regulation of reciprocal meiotic recombination	1	11
6044	GO:0010558	negative regulation of macromolecule biosynthetic process	1	2
6045	GO:0010648	negative regulation of cell communication	1	10
6046	GO:0010649	regulation of cell communication by electrical coupling	1	15
6047	GO:0010668	ectodermal cell differentiation	1	15
6048	GO:0010675	regulation of cellular carbohydrate metabolic process	1	2
6049	GO:0010677	negative regulation of cellular carbohydrate metabolic process	1	1
6050	GO:0010705	meiotic DNA double-strand break processing involved in reciprocal meiotic recombination	1	14
6051	GO:0010710	regulation of collagen catabolic process	1	1
6052	GO:0010720	positive regulation of cell development	1	14
6053	GO:0010738	regulation of protein kinase A signaling cascade	1	1
6054	GO:0010742	macrophage derived foam cell differentiation	1	3
6055	GO:0010756	positive regulation of plasminogen activation	1	2
6056	GO:0010769	regulation of cell morphogenesis involved in differentiation	1	1
6057	GO:0010792	DNA double-strand break processing involved in repair via single-strand annealing	1	5
6058	GO:0010797	regulation of multivesicular body size involved in endosome transport	1	7
6059	GO:0010812	negative regulation of cell-substrate adhesion	1	14
6060	GO:0010835	regulation of protein ADP-ribosylation	1	8
6061	GO:0010869	regulation of receptor biosynthetic process	1	1
6062	GO:0010878	cholesterol storage	1	3
6063	GO:0010914	positive regulation of sterigmatocystin biosynthetic process	1	17
6064	GO:0010922	positive regulation of phosphatase activity	1	1
6065	GO:0010947	negative regulation of meiotic joint molecule formation	1	3
6066	GO:0010948	negative regulation of cell cycle process	1	2
6067	GO:0010954	positive regulation of protein processing	1	3
6068	GO:0010964	regulation of chromatin silencing by small RNA	1	14
6069	GO:0010970	microtubule-based transport	1	3

6070	GO:0010989	negative regulation of low-density lipoprotein particle clearance	1	14
6071	GO:0014013	regulation of gliogenesis	1	1
6072	GO:0014014	negative regulation of gliogenesis	1	5
6073	GO:0014032	neural crest cell development	1	2
6074	GO:0014038	regulation of Schwann cell differentiation	1	1
6075	GO:0014042	positive regulation of neuron maturation	1	10
6076	GO:0014047	glutamate secretion	1	13
6077	GO:0014736	negative regulation of muscle atrophy	1	10
6078	GO:0014808	release of sequestered calcium ion into cytosol by sarcoplasmic reticulum	1	25
6079	GO:0014819	regulation of skeletal muscle contraction	1	2
6080	GO:0014829	vascular smooth muscle contraction	1	15
6081	GO:0014834	satellite cell maintenance involved in skeletal muscle regeneration	1	1
6082	GO:0014889	muscle atrophy	1	1
6083	GO:0015690	aluminum cation transport	1	5
6084	GO:0015789	UDP-N-acetylgalactosamine transport	1	5
6085	GO:0015821	methionine transport	1	2
6086	GO:0015833	peptide transport	1	7
6087	GO:0015838	betaine transport	1	1
6088	GO:0015847	putrescine transport	1	6
6089	GO:0015865	purine nucleotide transport	1	4
6090	GO:0015870	acetylcholine transport	1	6
6091	GO:0015942	formate metabolic process	1	3
6092	GO:0015968	stringent response	1	1
6093	GO:0016031	tRNA import into mitochondrion	1	12
6094	GO:0016056	rhodopsin mediated signaling pathway	1	1
6095	GO:0016064	immunoglobulin mediated immune response	1	1
6096	GO:0016068	type I hypersensitivity	1	1
6097	GO:0016072	rRNA metabolic process	1	2
6098	GO:0016073	snRNA metabolic process	1	3
6099	GO:0016081	synaptic vesicle docking involved in exocytosis	1	1
6100	GO:0016082	synaptic vesicle priming	1	11
6101	GO:0016106	sesquiterpenoid biosynthetic process	1	1
6102	GO:0016121	carotene catabolic process	1	1
6103	GO:0016124	xanthophyll catabolic process	1	1
6104	GO:0016127	sterol catabolic process	1	1
6105	GO:0016137	glycoside metabolic process	1	2
6106	GO:0016202	regulation of striated muscle tissue development	1	6
6107	GO:0016237	microautophagy	1	1
6108	GO:0016260	selenocysteine biosynthetic process	1	2
6109	GO:0016557	peroxisome membrane biogenesis	1	1
6110	GO:0017000	antibiotic biosynthetic process	1	4
6111	GO:0017001	antibiotic catabolic process	1	8
6112	GO:0017015	regulation of transforming growth factor beta receptor signaling pathway	1	7
6113	GO:0017121	phospholipid scrambling	1	22
6114	GO:0017186	peptidyl-pyroglutamic acid biosynthetic process, using glutaminyl-peptide cyclotransferase	1	4
6115	GO:0018013	N-terminal peptidyl-glycine methylation	1	10
6116	GO:0018022	peptidyl-lysine methylation	1	1
6117	GO:0018094	protein polyglycylation	1	2
6118	GO:0018095	protein polyglutamylation	1	2
6119	GO:0018117	protein adenylylation	1	1
6120	GO:0018131	oxazole or thiazole biosynthetic process	1	2
6121	GO:0018192	enzyme active site formation via L-cysteine persulfide	1	18
6122	GO:0018272	protein-pyridoxal-5-phosphate linkage via peptidyl-N6-pyridoxal phosphate-L-lysine	1	6
6123	GO:0018323	enzyme active site formation via L-cysteine sulfinic acid	1	2

6124	GO:0018342	protein prenylation	1	1
6125	GO:0018364	peptidyl-glutamine methylation	1	9
6126	GO:0018394	peptidyl-lysine acetylation	1	1
6127	GO:0018395	peptidyl-lysine hydroxylation to 5-hydroxy-L-lysine	1	1
6128	GO:0018888	3-chloroacrylic acid metabolic process	1	10
6129	GO:0018920	glyphosate metabolic process	1	1
6130	GO:0018924	mandelate metabolic process	1	7
6131	GO:0018958	phenol-containing compound metabolic process	1	2
6132	GO:0019054	modulation by virus of host cellular process	1	2
6133	GO:0019081	viral protein biosynthetic process	1	2
6134	GO:0019088	immortalization of host cell by virus	1	4
6135	GO:0019099	female germ-line sex determination	1	2
6136	GO:0019254	carnitine metabolic process, CoA-linked	1	3
6137	GO:0019354	siroheme biosynthetic process	1	4
6138	GO:0019357	nicotinate nucleotide biosynthetic process	1	20
6139	GO:0019381	atrazine catabolic process	1	13
6140	GO:0019441	tryptophan catabolic process to kynurenine	1	5
6141	GO:0019458	methionine catabolic process via 2-oxobutanoate	1	3
6142	GO:0019477	L-lysine catabolic process	1	19
6143	GO:0019484	beta-alanine catabolic process	1	10
6144	GO:0019516	lactate oxidation	1	8
6145	GO:0019532	oxalate transport	1	1
6146	GO:0019568	arabinose catabolic process	1	5
6147	GO:0019607	phenylethylamine catabolic process	1	1
6148	GO:0019646	aerobic electron transport chain	1	23
6149	GO:0019720	Mo-molybdopterin cofactor metabolic process	1	7
6150	GO:0019836	hemolysis by symbiont of host erythrocytes	1	13
6151	GO:0019860	uracil metabolic process	1	4
6152	GO:0019918	peptidyl-arginine methylation, to symmetrical-dimethyl arginine	1	9
6153	GO:0019988	charged-tRNA amino acid modification	1	22
6154	GO:0021515	cell differentiation in spinal cord	1	1
6155	GO:0021533	cell differentiation in hindbrain	1	1
6156	GO:0021543	pallium development	1	1
6157	GO:0021661	rhombomere 4 morphogenesis	1	12
6158	GO:0021670	lateral ventricle development	1	3
6159	GO:0021685	cerebellar granular layer structural organization	1	1
6160	GO:0021693	cerebellar Purkinje cell layer structural organization	1	1
6161	GO:0021696	cerebellar cortex morphogenesis	1	2
6162	GO:0021697	cerebellar cortex formation	1	1
6163	GO:0021782	glial cell development	1	4
6164	GO:0021797	forebrain anterior/posterior pattern specification	1	12
6165	GO:0021846	cell proliferation in forebrain	1	1
6166	GO:0022616	DNA strand elongation	1	1
6167	GO:0023057	negative regulation of signaling	1	10
6168	GO:0030049	muscle filament sliding	1	2
6169	GO:0030200	heparan sulfate proteoglycan catabolic process	1	5
6170	GO:0030299	intestinal cholesterol absorption	1	1
6171	GO:0030321	transepithelial chloride transport	1	21
6172	GO:0030327	prenylated protein catabolic process	1	2
6173	GO:0030328	prenylcysteine catabolic process	1	2
6174	GO:0030501	positive regulation of bone mineralization	1	15
6175	GO:0030522	intracellular receptor mediated signaling pathway	1	16
6176	GO:0030536	larval feeding behavior	1	4
6177	GO:0030638	polyketide metabolic process	1	2
6178	GO:0030702	chromatin silencing at centromere	1	2
6179	GO:0030835	negative regulation of actin filament depolymerization	1	2
6180	GO:0030836	positive regulation of actin filament depolymerization	1	4
6181	GO:0030856	regulation of epithelial cell differentiation	1	1
6182	GO:0030858	positive regulation of epithelial cell differentiation	1	2

6183	GO:0030917	midbrain-hindbrain boundary development	1	12
6184	GO:0030980	alpha-glucan catabolic process	1	10
6185	GO:0031017	exocrine pancreas development	1	5
6186	GO:0031018	endocrine pancreas development	1	2
6187	GO:0031023	microtubule organizing center organization	1	3
6188	GO:0031057	negative regulation of histone modification	1	6
6189	GO:0031076	embryonic camera-type eye development	1	1
6190	GO:0031126	snoRNA 3'-end processing	1	2
6191	GO:0031214	biomineral tissue development	1	6
6192	GO:0031292	gene conversion at mating-type locus, DNA double-strand break processing	1	3
6193	GO:0031329	regulation of cellular catabolic process	1	1
6194	GO:0031407	oxylipin metabolic process	1	5
6195	GO:0031442	positive regulation of mRNA 3'-end processing	1	2
6196	GO:0031577	spindle checkpoint	1	2
6197	GO:0031578	mitotic cell cycle spindle orientation checkpoint	1	6
6198	GO:0031591	wybutosine biosynthetic process	1	5
6199	GO:0031641	regulation of myelination	1	1
6200	GO:0031662	positive regulation of cyclin-dependent protein kinase activity involved in G2/M	1	3
6201	GO:0031953	negative regulation of protein autophosphorylation	1	1
6202	GO:0031960	response to corticosteroid stimulus	1	20
6203	GO:0032042	mitochondrial DNA metabolic process	1	33
6204	GO:0032233	positive regulation of actin filament bundle assembly	1	4
6205	GO:0032263	GMP salvage	1	1
6206	GO:0032328	alanine transport	1	6
6207	GO:0032388	positive regulation of intracellular transport	1	6
6208	GO:0032413	negative regulation of ion transmembrane transporter activity	1	1
6209	GO:0032425	positive regulation of mismatch repair	1	5
6210	GO:0032443	regulation of ergosterol biosynthetic process	1	2
6211	GO:0032461	positive regulation of protein oligomerization	1	1
6212	GO:0032474	otolith morphogenesis	1	3
6213	GO:0032479	regulation of type I interferon production	1	1
6214	GO:0032485	regulation of Ral protein signal transduction	1	1
6215	GO:0032487	regulation of Rap protein signal transduction	1	1
6216	GO:0032535	regulation of cellular component size	1	3
6217	GO:0032652	regulation of interleukin-1 production	1	1
6218	GO:0032715	negative regulation of interleukin-6 production	1	14
6219	GO:0032727	positive regulation of interferon-alpha production	1	3
6220	GO:0032733	positive regulation of interleukin-10 production	1	3
6221	GO:0032735	positive regulation of interleukin-12 production	1	3
6222	GO:0032755	positive regulation of interleukin-6 production	1	1
6223	GO:0032785	negative regulation of DNA-dependent transcription, elongation	1	5
6224	GO:0032835	glomerulus development	1	1
6225	GO:0032917	polyamine acetylation	1	1
6226	GO:0032955	regulation of barrier septum assembly	1	2
6227	GO:0032960	regulation of inositol trisphosphate biosynthetic process	1	10
6228	GO:0033002	muscle cell proliferation	1	2
6229	GO:0033014	tetrapyrrole biosynthetic process	1	5
6230	GO:0033168	conversion of ds siRNA to ss siRNA involved in RNA interference	1	2
6231	GO:0033214	iron assimilation by chelation and transport	1	3
6232	GO:0033259	plastid DNA replication	1	6
6233	GO:0033260	DNA replication involved in S phase	1	1
6234	GO:0033280	response to vitamin D	1	15
6235	GO:0033303	quercetin O-glucoside biosynthetic process	1	1
6236	GO:0033317	pantothenate biosynthetic process from valine	1	4
6237	GO:0033330	kaempferol O-glucoside biosynthetic process	1	1
6238	GO:0033339	pectoral fin development	1	6

6239	GO:0033354	chlorophyll cycle	1	11
6240	GO:0033489	cholesterol biosynthetic process via desmosterol	1	7
6241	GO:0033567	DNA replication, Okazaki fragment processing	1	10
6242	GO:0033605	positive regulation of catecholamine secretion	1	1
6243	GO:0033624	negative regulation of integrin activation	1	1
6244	GO:0033627	cell adhesion mediated by integrin	1	2
6245	GO:0033673	negative regulation of kinase activity	1	14
6246	GO:0033864	positive regulation of NAD(P)H oxidase activity	1	8
6247	GO:0033955	mitochondrial DNA inheritance	1	19
6248	GO:0034048	negative regulation of protein phosphatase type 2A activity	1	6
6249	GO:0034087	establishment of mitotic sister chromatid cohesion	1	4
6250	GO:0034088	maintenance of mitotic sister chromatid cohesion	1	3
6251	GO:0034090	maintenance of meiotic sister chromatid cohesion	1	5
6252	GO:0034121	regulation of toll-like receptor signaling pathway	1	1
6253	GO:0034162	toll-like receptor 9 signaling pathway	1	1
6254	GO:0034205	beta-amyloid formation	1	1
6255	GO:0034213	quinolinate catabolic process	1	7
6256	GO:0034248	regulation of cellular amide metabolic process	1	6
6257	GO:0034263	autophagy in response to ER overload	1	2
6258	GO:0034265	isopentenyl adenine biosynthetic process	1	1
6259	GO:0034276	kynurenic acid biosynthetic process	1	2
6260	GO:0034308	primary alcohol metabolic process	1	1
6261	GO:0034316	negative regulation of Arp2/3 complex-mediated actin nucleation	1	1
6262	GO:0034351	negative regulation of glial cell apoptotic process	1	32
6263	GO:0034375	high-density lipoprotein particle remodeling	1	13
6264	GO:0034402	recruitment of 3'-end processing factors to RNA polymerase II holoenzyme complex	1	3
6265	GO:0034415	tRNA 3'-trailer cleavage, exonucleolytic	1	5
6266	GO:0034434	sterol esterification	1	1
6267	GO:0034435	cholesterol esterification	1	3
6268	GO:0034462	small-subunit processome assembly	1	11
6269	GO:0034477	U6 snRNA 3'-end processing	1	6
6270	GO:0034551	mitochondrial respiratory chain complex III assembly	1	8
6271	GO:0034553	mitochondrial respiratory chain complex II assembly	1	7
6272	GO:0034653	retinoic acid catabolic process	1	12
6273	GO:0034655	nucleobase-containing compound catabolic process	1	1
6274	GO:0034661	ncRNA catabolic process	1	1
6275	GO:0034729	histone H3-K79 methylation	1	3
6276	GO:0034766	negative regulation of ion transmembrane transport	1	12
6277	GO:0035011	melanotic encapsulation of foreign target	1	1
6278	GO:0035044	sperm aster formation	1	1
6279	GO:0035047	centrosomal and pronuclear rotation	1	1
6280	GO:0035050	embryonic heart tube development	1	15
6281	GO:0035073	pupariation	1	42
6282	GO:0035089	establishment of apical/basal cell polarity	1	1
6283	GO:0035107	appendage morphogenesis	1	15
6284	GO:0035108	limb morphogenesis	1	4
6285	GO:0035115	embryonic forelimb morphogenesis	1	2
6286	GO:0035118	embryonic pectoral fin morphogenesis	1	12
6287	GO:0035166	post-embryonic hemopoiesis	1	13
6288	GO:0035186	syncytial blastoderm mitotic cell cycle	1	2
6289	GO:0035187	hatching behavior	1	10
6290	GO:0035202	tracheal pit formation in open tracheal system	1	2
6291	GO:0035206	regulation of hemocyte proliferation	1	7
6292	GO:0035212	cell competition in a multicellular organism	1	1
6293	GO:0035247	peptidyl-arginine omega-N-methylation	1	3
6294	GO:0035249	synaptic transmission, glutamatergic	1	1
6295	GO:0035304	regulation of protein dephosphorylation	1	1

6296	GO:0035318	imaginal disc-derived wing hair outgrowth	1	1
6297	GO:0035357	peroxisome proliferator activated receptor signaling pathway	1	3
6298	GO:0035404	histone-serine phosphorylation	1	2
6299	GO:0035418	protein localization to synapse	1	1
6300	GO:0035606	peptidyl-cysteine S-trans-nitrosylation	1	2
6301	GO:0035609	C-terminal protein deglutamylation	1	7
6302	GO:0035634	response to stilbenoid	1	2
6303	GO:0035767	endothelial cell chemotaxis	1	1
6304	GO:0035799	ureter maturation	1	2
6305	GO:0035826	rubidium ion transport	1	13
6306	GO:0035855	megakaryocyte development	1	12
6307	GO:0035863	dITP catabolic process	1	1
6308	GO:0035924	cellular response to vascular endothelial growth factor stimulus	1	2
6309	GO:0035928	rRNA import into mitochondrion	1	7
6310	GO:0035964	COPI-coated vesicle budding	1	1
6311	GO:0036003	positive regulation of transcription from RNA polymerase II promoter in response to stress	1	3
6312	GO:0036060	slit diaphragm assembly	1	7
6313	GO:0036068	light-independent chlorophyll biosynthetic process	1	3
6314	GO:0036152	phosphatidylethanolamine acyl-chain remodeling	1	26
6315	GO:0036171	filamentous growth of a population of unicellular organisms in response to chemical stimulus	1	4
6316	GO:0036172	thiamine salvage	1	3
6317	GO:0036205	histone catabolic process	1	10
6318	GO:0036245	cellular response to menadione	1	4
6319	GO:0036269	swimming behavior	1	1
6320	GO:0036295	cellular response to increased oxygen levels	1	3
6321	GO:0039520	induction by virus of host autophagy	1	2
6322	GO:0039534	negative regulation of MDA-5 signaling pathway	1	13
6323	GO:0039535	regulation of RIG-I signaling pathway	1	1
6324	GO:0040017	positive regulation of locomotion	1	4
6325	GO:0040031	snRNA modification	1	18
6326	GO:0040036	regulation of fibroblast growth factor receptor signaling pathway	1	1
6327	GO:0042001	hermaphrodite somatic sex determination	1	9
6328	GO:0042058	regulation of epidermal growth factor receptor signaling pathway	1	1
6329	GO:0042063	gliogenesis	1	2
6330	GO:0042113	B cell activation	1	6
6331	GO:0042135	neurotransmitter catabolic process	1	4
6332	GO:0042157	lipoprotein metabolic process	1	22
6333	GO:0042159	lipoprotein catabolic process	1	32
6334	GO:0042245	RNA repair	1	2
6335	GO:0042267	natural killer cell mediated cytotoxicity	1	1
6336	GO:0042278	purine nucleoside metabolic process	1	3
6337	GO:0042327	positive regulation of phosphorylation	1	1
6338	GO:0042330	taxis	1	1
6339	GO:0042345	regulation of NF-kappaB import into nucleus	1	1
6340	GO:0042376	phylloquinone catabolic process	1	5
6341	GO:0042403	thyroid hormone metabolic process	1	7
6342	GO:0042415	norepinephrine metabolic process	1	4
6343	GO:0042461	photoreceptor cell development	1	4
6344	GO:0042462	eye photoreceptor cell development	1	26
6345	GO:0042634	regulation of hair cycle	1	4
6346	GO:0042676	compound eye cone cell fate commitment	1	2
6347	GO:0042694	muscle cell fate specification	1	2
6348	GO:0042727	flavin-containing compound biosynthetic process	1	7
6349	GO:0042743	hydrogen peroxide metabolic process	1	2
6350	GO:0042756	drinking behavior	1	9

6351	GO:0042780	tRNA 3'-end processing	1	15
6352	GO:0042789	mRNA transcription from RNA polymerase II promoter	1	3
6353	GO:0042819	vitamin B6 biosynthetic process	1	1
6354	GO:0042853	L-alanine catabolic process	1	5
6355	GO:0042986	positive regulation of amyloid precursor protein biosynthetic process	1	3
6356	GO:0042990	regulation of transcription factor import into nucleus	1	17
6357	GO:0043011	myeloid dendritic cell differentiation	1	1
6358	GO:0043030	regulation of macrophage activation	1	7
6359	GO:0043032	positive regulation of macrophage activation	1	3
6360	GO:0043039	tRNA aminoacylation	1	8
6361	GO:0043042	amino acid adenylylation by nonribosomal peptide synthase	1	26
6362	GO:0043050	pharyngeal pumping	1	4
6363	GO:0043094	cellular metabolic compound salvage	1	3
6364	GO:0043096	purine nucleobase salvage	1	1
6365	GO:0043150	DNA synthesis involved in double-strand break repair via homologous recombination	1	6
6366	GO:0043217	myelin maintenance	1	4
6367	GO:0043244	regulation of protein complex disassembly	1	1
6368	GO:0043277	apoptotic cell clearance	1	2
6369	GO:0043305	negative regulation of mast cell degranulation	1	2
6370	GO:0043374	CD8-positive, alpha-beta T cell differentiation	1	1
6371	GO:0043409	negative regulation of MAPK cascade	1	13
6372	GO:0043490	malate-aspartate shuttle	1	10
6373	GO:0043506	regulation of JUN kinase activity	1	6
6374	GO:0043535	regulation of blood vessel endothelial cell migration	1	6
6375	GO:0043555	regulation of translation in response to stress	1	4
6376	GO:0043603	cellular amide metabolic process	1	3
6377	GO:0043605	cellular amide catabolic process	1	2
6378	GO:0043610	regulation of carbohydrate utilization	1	10
6379	GO:0043612	isoprene biosynthetic process	1	1
6380	GO:0043624	cellular protein complex disassembly	1	3
6381	GO:0043628	ncRNA 3'-end processing	1	1
6382	GO:0043633	polyadenylation-dependent RNA catabolic process	1	1
6383	GO:0043952	protein transport by the Sec complex	1	7
6384	GO:0044036	cell wall macromolecule metabolic process	1	1
6385	GO:0044057	regulation of system process	1	1
6386	GO:0044091	membrane biogenesis	1	1
6387	GO:0044119	growth of symbiont in host cell	1	2
6388	GO:0044268	multicellular organismal protein metabolic process	1	9
6389	GO:0044273	sulfur compound catabolic process	1	1
6390	GO:0044275	cellular carbohydrate catabolic process	1	5
6391	GO:0044387	negative regulation of protein kinase activity by regulation of protein phosphorylation	1	3
6392	GO:0044524	protein sulfhydration	1	6
6393	GO:0044557	relaxation of smooth muscle	1	15
6394	GO:0044648	histone H3-K4 dimethylation	1	10
6395	GO:0045008	depyrimidination	1	2
6396	GO:0045026	plasma membrane fusion	1	19
6397	GO:0045048	protein insertion into ER membrane	1	2
6398	GO:0045075	regulation of interleukin-12 biosynthetic process	1	17
6399	GO:0045117	azole transport	1	1
6400	GO:0045200	establishment of neuroblast polarity	1	1
6401	GO:0045201	maintenance of neuroblast polarity	1	34
6402	GO:0045213	neurotransmitter receptor metabolic process	1	7
6403	GO:0045453	bone resorption	1	1
6404	GO:0045466	R7 cell differentiation	1	2
6405	GO:0045468	regulation of R8 cell spacing in compound eye	1	2
6406	GO:0045488	pectin metabolic process	1	1

6407	GO:0045560	regulation of TRAIL receptor biosynthetic process	1	2
6408	GO:0045576	mast cell activation	1	6
6409	GO:0045580	regulation of T cell differentiation	1	1
6410	GO:0045585	positive regulation of cytotoxic T cell differentiation	1	4
6411	GO:0045599	negative regulation of fat cell differentiation	1	1
6412	GO:0045602	negative regulation of endothelial cell differentiation	1	3
6413	GO:0045605	negative regulation of epidermal cell differentiation	1	1
6414	GO:0045621	positive regulation of lymphocyte differentiation	1	5
6415	GO:0045622	regulation of T-helper cell differentiation	1	6
6416	GO:0045646	regulation of erythrocyte differentiation	1	3
6417	GO:0045654	positive regulation of megakaryocyte differentiation	1	19
6418	GO:0045670	regulation of osteoclast differentiation	1	2
6419	GO:0045724	positive regulation of flagellum assembly	1	2
6420	GO:0045726	positive regulation of integrin biosynthetic process	1	8
6421	GO:0045743	positive regulation of fibroblast growth factor receptor signaling pathway	1	11
6422	GO:0045752	positive regulation of Toll signaling pathway	1	11
6423	GO:0045779	negative regulation of bone resorption	1	14
6424	GO:0045830	positive regulation of isotype switching	1	9
6425	GO:0045848	positive regulation of nitrogen utilization	1	2
6426	GO:0045870	positive regulation of retroviral genome replication	1	1
6427	GO:0045907	positive regulation of vasoconstriction	1	7
6428	GO:0045956	positive regulation of calcium ion-dependent exocytosis	1	12
6429	GO:0046015	regulation of transcription by glucose	1	11
6430	GO:0046031	ADP metabolic process	1	3
6431	GO:0046048	UDP metabolic process	1	22
6432	GO:0046084	adenine biosynthetic process	1	9
6433	GO:0046092	deoxycytidine metabolic process	1	3
6434	GO:0046100	hypoxanthine metabolic process	1	1
6435	GO:0046104	thymidine metabolic process	1	1
6436	GO:0046165	alcohol biosynthetic process	1	1
6437	GO:0046184	aldehyde biosynthetic process	1	1
6438	GO:0046364	monosaccharide biosynthetic process	1	3
6439	GO:0046373	L-arabinose metabolic process	1	20
6440	GO:0046396	D-galacturonate metabolic process	1	8
6441	GO:0046398	UDP-glucuronate metabolic process	1	4
6442	GO:0046417	chorismate metabolic process	1	23
6443	GO:0046451	diaminopimelate metabolic process	1	5
6444	GO:0046470	phosphatidylcholine metabolic process	1	2
6445	GO:0046471	phosphatidylglycerol metabolic process	1	1
6446	GO:0046498	S-adenosylhomocysteine metabolic process	1	24
6447	GO:0046499	S-adenosylmethioninamine metabolic process	1	24
6448	GO:0046511	sphinganine biosynthetic process	1	7
6449	GO:0046530	photoreceptor cell differentiation	1	1
6450	GO:0046534	positive regulation of photoreceptor cell differentiation	1	1
6451	GO:0046649	lymphocyte activation	1	5
6452	GO:0046830	positive regulation of RNA import into nucleus	1	4
6453	GO:0046845	branched duct epithelial cell fate determination, open tracheal system	1	2
6454	GO:0046900	tetrahydrofolylpolyglutamate metabolic process	1	4
6455	GO:0046901	tetrahydrofolylpolyglutamate biosynthetic process	1	13
6456	GO:0046902	regulation of mitochondrial membrane permeability	1	2
6457	GO:0046903	secretion	1	2
6458	GO:0046940	nucleoside monophosphate phosphorylation	1	1
6459	GO:0046952	ketone body catabolic process	1	3
6460	GO:0046967	cytosol to ER transport	1	15
6461	GO:0048034	heme O biosynthetic process	1	2
6462	GO:0048041	focal adhesion assembly	1	13
6463	GO:0048058	compound eye corneal lens development	1	1
6464	GO:0048136	male germ-line cyst formation	1	4

6465	GO:0048137	spermatocyte division	1	4
6466	GO:0048138	germ-line cyst encapsulation	1	10
6467	GO:0048139	female germ-line cyst encapsulation	1	2
6468	GO:0048148	behavioral response to cocaine	1	8
6469	GO:0048199	vesicle targeting, to, from or within Golgi	1	1
6470	GO:0048259	regulation of receptor-mediated endocytosis	1	7
6471	GO:0048261	negative regulation of receptor-mediated endocytosis	1	2
6472	GO:0048285	organelle fission	1	1
6473	GO:0048291	isotype switching to IgG isotypes	1	3
6474	GO:0048309	endoplasmic reticulum inheritance	1	1
6475	GO:0048453	sepal formation	1	6
6476	GO:0048469	cell maturation	1	9
6477	GO:0048526	imaginal disc-derived wing expansion	1	1
6478	GO:0048542	lymph gland development	1	1
6479	GO:0048546	digestive tract morphogenesis	1	5
6480	GO:0048566	embryonic digestive tract development	1	2
6481	GO:0048572	short-day photoperiodism	1	1
6482	GO:0048592	eye morphogenesis	1	1
6483	GO:0048596	embryonic camera-type eye morphogenesis	1	10
6484	GO:0048611	embryonic ectodermal digestive tract development	1	15
6485	GO:0048692	negative regulation of axon extension involved in regeneration	1	13
6486	GO:0048703	embryonic viscerocranium morphogenesis	1	3
6487	GO:0048704	embryonic skeletal system morphogenesis	1	2
6488	GO:0048707	instar larval or pupal morphogenesis	1	2
6489	GO:0048713	regulation of oligodendrocyte differentiation	1	1
6490	GO:0048789	cytoskeletal matrix organization at active zone	1	4
6491	GO:0048791	calcium ion-dependent exocytosis of neurotransmitter	1	1
6492	GO:0048854	brain morphogenesis	1	12
6493	GO:0048871	multicellular organismal homeostasis	1	13
6494	GO:0048925	lateral line system development	1	1
6495	GO:0048936	peripheral nervous system neuron axonogenesis	1	1
6496	GO:0050427	3'-phosphoadenosine 5'-phosphosulfate metabolic process	1	1
6497	GO:0050654	chondroitin sulfate proteoglycan metabolic process	1	2
6498	GO:0050658	RNA transport	1	2
6499	GO:0050673	epithelial cell proliferation	1	9
6500	GO:0050729	positive regulation of inflammatory response	1	2
6501	GO:0050730	regulation of peptidyl-tyrosine phosphorylation	1	2
6502	GO:0050764	regulation of phagocytosis	1	5
6503	GO:0050787	detoxification of mercury ion	1	2
6504	GO:0050807	regulation of synapse organization	1	2
6505	GO:0050817	coagulation	1	6
6506	GO:0050850	positive regulation of calcium-mediated signaling	1	1
6507	GO:0050853	B cell receptor signaling pathway	1	4
6508	GO:0050860	negative regulation of T cell receptor signaling pathway	1	7
6509	GO:0050863	regulation of T cell activation	1	3
6510	GO:0050864	regulation of B cell activation	1	2
6511	GO:0050868	negative regulation of T cell activation	1	7
6512	GO:0050882	voluntary musculoskeletal movement	1	1
6513	GO:0050916	sensory perception of sweet taste	1	6
6514	GO:0050918	positive chemotaxis	1	1
6515	GO:0050921	positive regulation of chemotaxis	1	15
6516	GO:0050957	equilibrioception	1	26
6517	GO:0050975	sensory perception of touch	1	1
6518	GO:0050983	deoxyhypusine biosynthetic process from spermidine	1	13
6519	GO:0050993	dimethylallyl diphosphate metabolic process	1	1
6520	GO:0051044	positive regulation of membrane protein ectodomain proteolysis	1	1
6521	GO:0051048	negative regulation of secretion	1	6
6522	GO:0051051	negative regulation of transport	1	3

6523	GO:0051083	'de novo' cotranslational protein folding	1	6
6524	GO:0051145	smooth muscle cell differentiation	1	2
6525	GO:0051160	L-xylitol catabolic process	1	1
6526	GO:0051180	vitamin transport	1	3
6527	GO:0051186	cofactor metabolic process	1	3
6528	GO:0051224	negative regulation of protein transport	1	1
6529	GO:0051230	spindle disassembly	1	16
6530	GO:0051232	meiotic spindle elongation	1	2
6531	GO:0051249	regulation of lymphocyte activation	1	7
6532	GO:0051272	positive regulation of cellular component movement	1	4
6533	GO:0051279	regulation of release of sequestered calcium ion into cytosol	1	1
6534	GO:0051303	establishment of chromosome localization	1	1
6535	GO:0051385	response to mineralocorticoid stimulus	1	1
6536	GO:0051391	tRNA acetylation	1	6
6537	GO:0051444	negative regulation of ubiquitin-protein ligase activity	1	2
6538	GO:0051445	regulation of meiotic cell cycle	1	4
6539	GO:0051481	reduction of cytosolic calcium ion concentration	1	15
6540	GO:0051512	positive regulation of unidimensional cell growth	1	3
6541	GO:0051554	flavonol metabolic process	1	1
6542	GO:0051570	regulation of histone H3-K9 methylation	1	15
6543	GO:0051583	dopamine uptake involved in synaptic transmission	1	2
6544	GO:0051654	establishment of mitochondrion localization	1	1
6545	GO:0051673	membrane disruption in other organism	1	1
6546	GO:0051682	galactomannan catabolic process	1	1
6547	GO:0051729	germline cell cycle switching, mitotic to meiotic cell cycle	1	2
6548	GO:0051754	meiotic sister chromatid cohesion, centromeric	1	2
6549	GO:0051771	negative regulation of nitric-oxide synthase biosynthetic process	1	7
6550	GO:0051776	detection of redox state	1	17
6551	GO:0051784	negative regulation of nuclear division	1	15
6552	GO:0051819	induction of tumor, nodule, or growth in other organism involved in symbiotic interaction	1	2
6553	GO:0051875	pigment granule localization	1	26
6554	GO:0051899	membrane depolarization	1	2
6555	GO:0051984	positive regulation of chromosome segregation	1	10
6556	GO:0051985	negative regulation of chromosome segregation	1	15
6557	GO:0052060	evasion or tolerance by symbiont of host-produced nitric oxide	1	1
6558	GO:0052573	UDP-D-galactose metabolic process	1	4
6559	GO:0052889	9,9'-di-cis-zeta-carotene desaturation to 7,9,7',9'-tetra-cis-lycopene	1	17
6560	GO:0055014	atrial cardiac muscle cell development	1	12
6561	GO:0055015	ventricular cardiac muscle cell development	1	1
6562	GO:0055067	monovalent inorganic cation homeostasis	1	1
6563	GO:0055068	cobalt ion homeostasis	1	8
6564	GO:0055071	manganese ion homeostasis	1	5
6565	GO:0055108	Golgi to transport vesicle transport	1	12
6566	GO:0060025	regulation of synaptic activity	1	1
6567	GO:0060045	positive regulation of cardiac muscle cell proliferation	1	1
6568	GO:0060055	angiogenesis involved in wound healing	1	6
6569	GO:0060059	embryonic retina morphogenesis in camera-type eye	1	10
6570	GO:0060079	regulation of excitatory postsynaptic membrane potential	1	1
6571	GO:0060081	membrane hyperpolarization	1	2
6572	GO:0060149	negative regulation of posttranscriptional gene silencing	1	11
6573	GO:0060152	microtubule-based peroxisome localization	1	6
6574	GO:0060184	cell cycle switching	1	7
6575	GO:0060235	lens induction in camera-type eye	1	10
6576	GO:0060250	germ-line stem-cell niche homeostasis	1	4

6577	GO:0060252	positive regulation of glial cell proliferation	1	10
6578	GO:0060259	regulation of feeding behavior	1	2
6579	GO:0060265	positive regulation of respiratory burst involved in inflammatory response	1	4
6580	GO:0060266	negative regulation of respiratory burst involved in inflammatory response	1	4
6581	GO:0060281	regulation of oocyte development	1	17
6582	GO:0060296	regulation of cilium beat frequency involved in ciliary motility	1	2
6583	GO:0060323	head morphogenesis	1	1
6584	GO:0060326	cell chemotaxis	1	3
6585	GO:0060327	cytoplasmic actin-based contraction involved in cell motility	1	4
6586	GO:0060373	regulation of ventricular cardiomyocyte membrane depolarization	1	1
6587	GO:0060402	calcium ion transport into cytosol	1	2
6588	GO:0060404	axonemal microtubule depolymerization	1	7
6589	GO:0060421	positive regulation of heart growth	1	9
6590	GO:0060427	lung connective tissue development	1	7
6591	GO:0060438	trachea development	1	1
6592	GO:0060441	epithelial tube branching involved in lung morphogenesis	1	5
6593	GO:0060446	branching involved in open tracheal system development	1	1
6594	GO:0060491	regulation of cell projection assembly	1	4
6595	GO:0060543	negative regulation of strand invasion	1	10
6596	GO:0060544	regulation of necroptosis	1	1
6597	GO:0060545	positive regulation of necroptosis	1	1
6598	GO:0060546	negative regulation of necroptosis	1	1
6599	GO:0060574	intestinal epithelial cell maturation	1	4
6600	GO:0060718	chorionic trophoblast cell differentiation	1	7
6601	GO:0060734	regulation of eIF2 alpha phosphorylation by endoplasmic reticulum stress	1	2
6602	GO:0060800	regulation of cell differentiation involved in embryonic placenta development	1	10
6603	GO:0060904	regulation of protein folding in endoplasmic reticulum	1	2
6604	GO:0061037	negative regulation of cartilage development	1	2
6605	GO:0061047	positive regulation of branching involved in lung morphogenesis	1	2
6606	GO:0061072	iris morphogenesis	1	10
6607	GO:0061090	positive regulation of sequestering of zinc ion	1	3
6608	GO:0061178	regulation of insulin secretion involved in cellular response to glucose stimulus	1	8
6609	GO:0061179	negative regulation of insulin secretion involved in cellular response to glucose stimulus	1	5
6610	GO:0061191	positive regulation of vacuole fusion, non-autophagic	1	1
6611	GO:0061246	establishment or maintenance of bipolar cell polarity regulating cell shape	1	1
6612	GO:0061300	cerebellum vasculature development	1	13
6613	GO:0061351	neural precursor cell proliferation	1	5
6614	GO:0061371	determination of heart left/right asymmetry	1	4
6615	GO:0061430	bone trabecula morphogenesis	1	8
6616	GO:0070055	HAC1-type intron splice site recognition and cleavage	1	2
6617	GO:0070078	histone H3-R2 demethylation	1	1
6618	GO:0070079	histone H4-R3 demethylation	1	1
6619	GO:0070127	tRNA aminoacylation for mitochondrial protein translation	1	6
6620	GO:0070142	synaptic vesicle budding	1	12
6621	GO:0070154	mitochondrial lysyl-tRNA aminoacylation	1	12
6622	GO:0070189	kynurenine metabolic process	1	3
6623	GO:0070200	establishment of protein localization to telomere	1	3
6624	GO:0070291	N-acylethanolamine metabolic process	1	10

6625	GO:0070294	renal sodium ion absorption	1	4
6626	GO:0070296	sarcoplasmic reticulum calcium ion transport	1	1
6627	GO:0070314	G1 to G0 transition	1	1
6628	GO:0070318	positive regulation of G0 to G1 transition	1	2
6629	GO:0070424	regulation of nucleotide-binding oligomerization domain containing signaling pathway	1	1
6630	GO:0070452	positive regulation of ergosterol biosynthetic process	1	2
6631	GO:0070462	plus-end specific microtubule depolymerization	1	7
6632	GO:0070493	thrombin receptor signaling pathway	1	3
6633	GO:0070528	protein kinase C signaling cascade	1	3
6634	GO:0070634	transepithelial ammonium transport	1	21
6635	GO:0070893	transposon integration	1	7
6636	GO:0070894	regulation of transposon integration	1	1
6637	GO:0070897	DNA-dependent transcriptional preinitiation complex assembly	1	16
6638	GO:0070898	RNA polymerase III transcriptional preinitiation complex assembly	1	7
6639	GO:0070901	mitochondrial tRNA methylation	1	15
6640	GO:0070902	mitochondrial tRNA pseudouridine synthesis	1	1
6641	GO:0070989	oxidative demethylation	1	9
6642	GO:0070994	detection of oxidative stress	1	3
6643	GO:0071025	RNA surveillance	1	1
6644	GO:0071031	nuclear mRNA surveillance of mRNA 3'-end processing	1	7
6645	GO:0071173	spindle assembly checkpoint	1	15
6646	GO:0071241	cellular response to inorganic substance	1	1
6647	GO:0071264	positive regulation of translational initiation in response to starvation	1	2
6648	GO:0071266	'de novo' L-methionine biosynthetic process	1	3
6649	GO:0071299	cellular response to vitamin A	1	12
6650	GO:0071314	cellular response to cocaine	1	8
6651	GO:0071348	cellular response to interleukin-11	1	8
6652	GO:0071359	cellular response to dsRNA	1	11
6653	GO:0071368	cellular response to cytokinin stimulus	1	7
6654	GO:0071373	cellular response to luteinizing hormone stimulus	1	1
6655	GO:0071374	cellular response to parathyroid hormone stimulus	1	3
6656	GO:0071375	cellular response to peptide hormone stimulus	1	14
6657	GO:0071384	cellular response to corticosteroid stimulus	1	14
6658	GO:0071394	cellular response to testosterone stimulus	1	3
6659	GO:0071436	sodium ion export	1	15
6660	GO:0071477	cellular hypotonic salinity response	1	13
6661	GO:0071485	cellular response to absence of light	1	1
6662	GO:0071514	genetic imprinting	1	1
6663	GO:0071569	protein ufmylation	1	1
6664	GO:0071570	cement gland development	1	4
6665	GO:0071600	otic vesicle morphogenesis	1	12
6666	GO:0071622	regulation of granulocyte chemotaxis	1	13
6667	GO:0071651	positive regulation of chemokine (C-C motif) ligand 5 production	1	6
6668	GO:0071768	mycolic acid biosynthetic process	1	1
6669	GO:0071774	response to fibroblast growth factor stimulus	1	4
6670	GO:0071827	plasma lipoprotein particle organization	1	1
6671	GO:0071866	negative regulation of apoptotic process in bone marrow	1	2
6672	GO:0071869	response to catecholamine stimulus	1	6
6673	GO:0071896	protein localization to adherens junction	1	1
6674	GO:0071918	urea transmembrane transport	1	7
6675	GO:0071973	bacterial-type flagellar cell motility	1	17
6676	GO:0072077	renal vesicle morphogenesis	1	2
6677	GO:0072078	nephron tubule morphogenesis	1	2
6678	GO:0072112	glomerular visceral epithelial cell differentiation	1	7
6679	GO:0072156	distal tubule morphogenesis	1	4
6680	GO:0072229	metanephric proximal convoluted tubule development	1	3

6681	GO:0072329	monocarboxylic acid catabolic process	1	9
6682	GO:0072337	modified amino acid transport	1	32
6683	GO:0072344	rescue of stalled ribosome	1	10
6684	GO:0072376	protein activation cascade	1	1
6685	GO:0072429	response to intra-S DNA damage checkpoint signal	1	2
6686	GO:0072524	pyridine-containing compound metabolic process	1	5
6687	GO:0072525	pyridine-containing compound biosynthetic process	1	1
6688	GO:0072527	pyrimidine-containing compound metabolic process	1	4
6689	GO:0072578	neurotransmitter-gated ion channel clustering	1	13
6690	GO:0072655	establishment of protein localization in mitochondrion	1	6
6691	GO:0072673	lamellipodium morphogenesis	1	1
6692	GO:0072675	osteoclast fusion	1	8
6693	GO:0072684	mitochondrial tRNA 3'-trailer cleavage, endonucleolytic	1	10
6694	GO:0072697	protein localization to cell cortex	1	1
6695	GO:0072718	response to cisplatin	1	1
6696	GO:0075307	positive regulation of conidium formation	1	17
6697	GO:0080003	thalianol metabolic process	1	1
6698	GO:0080028	nitrile biosynthetic process	1	2
6699	GO:0080056	petal vascular tissue pattern formation	1	7
6700	GO:0080057	sepal vascular tissue pattern formation	1	7
6701	GO:0080088	spermidine hydroxycinnamate conjugate biosynthetic process	1	1
6702	GO:0085030	mutualism	1	1
6703	GO:0086010	membrane depolarization involved in regulation of action potential	1	11
6704	GO:0086064	cell communication by electrical coupling involved in cardiac conduction	1	15
6705	GO:0090038	negative regulation of protein kinase C signaling cascade	1	1
6706	GO:0090044	positive regulation of tubulin deacetylation	1	7
6707	GO:0090045	positive regulation of deacetylase activity	1	7
6708	GO:0090065	regulation of production of siRNA involved in RNA interference	1	10
6709	GO:0090074	negative regulation of protein homodimerization activity	1	2
6710	GO:0090101	negative regulation of transmembrane receptor protein serine/threonine kinase signaling pathway	1	3
6711	GO:0090114	COPII-coated vesicle budding	1	1
6712	GO:0090140	regulation of mitochondrial fission	1	10
6713	GO:0090169	regulation of spindle assembly	1	3
6714	GO:0090218	positive regulation of lipid kinase activity	1	1
6715	GO:0090232	positive regulation of spindle checkpoint	1	6
6716	GO:0090234	regulation of kinetochore assembly	1	3
6717	GO:0090239	regulation of histone H4 acetylation	1	2
6718	GO:0090241	negative regulation of histone H4 acetylation	1	9
6719	GO:0090242	retinoic acid receptor signaling pathway involved in somitogenesis	1	12
6720	GO:0090276	regulation of peptide hormone secretion	1	1
6721	GO:0090308	regulation of methylation-dependent chromatin silencing	1	9
6722	GO:0090322	regulation of superoxide metabolic process	1	7
6723	GO:0090335	regulation of brown fat cell differentiation	1	10
6724	GO:0090382	phagosome maturation	1	6
6725	GO:0097006	regulation of plasma lipoprotein particle levels	1	1
6726	GO:0097046	replication fork progression beyond termination site	1	1
6727	GO:0097150	neuronal stem cell maintenance	1	13
6728	GO:0097187	dentinogenesis	1	7
6729	GO:0097202	activation of cysteine-type endopeptidase activity	1	2
6730	GO:0097206	nephrocyte filtration	1	1
6731	GO:0097212	lysosomal membrane organization	1	12
6732	GO:0097222	mitochondrial mRNA polyadenylation	1	7
6733	GO:0097237	cellular response to toxin	1	17
6734	GO:0097250	mitochondrial respiratory chain supercomplex assembly	1	3

6735	GO:1900004	negative regulation of serine-type endopeptidase activity	1	6
6736	GO:1900015	regulation of cytokine production involved in inflammatory response	1	3
6737	GO:1900029	positive regulation of ruffle assembly	1	2
6738	GO:1900038	negative regulation of cellular response to hypoxia	1	17
6739	GO:1900073	regulation of neuromuscular synaptic transmission	1	9
6740	GO:1900120	regulation of receptor binding	1	7
6741	GO:1900131	negative regulation of lipid binding	1	1
6742	GO:1900138	negative regulation of phospholipase A2 activity	1	6
6743	GO:1900165	negative regulation of interleukin-6 secretion	1	2
6744	GO:1900194	negative regulation of oocyte maturation	1	4
6745	GO:1900195	positive regulation of oocyte maturation	1	2
6746	GO:1900198	positive regulation of penicillin biosynthetic process	1	1
6747	GO:1900235	negative regulation of Kit signaling pathway	1	2
6748	GO:1900273	positive regulation of long-term synaptic potentiation	1	2
6749	GO:1900368	regulation of RNA interference	1	11
6750	GO:1900430	positive regulation of filamentous growth of a population of unicellular organisms	1	1
6751	GO:1900674	olefin biosynthetic process	1	1
6752	GO:1900747	negative regulation of vascular endothelial growth factor signaling pathway	1	3
6753	GO:1901006	ubiquinone-6 biosynthetic process	1	5
6754	GO:1901142	insulin metabolic process	1	2
6755	GO:2000025	regulation of leaf formation	1	4
6756	GO:2000042	negative regulation of double-strand break repair via homologous recombination	1	9
6757	GO:2000071	regulation of defense response by callose deposition	1	1
6758	GO:2000105	positive regulation of DNA-dependent DNA replication	1	18
6759	GO:2000136	regulation of cell proliferation involved in heart morphogenesis	1	2
6760	GO:2000155	positive regulation of flagellar cell motility	1	2
6761	GO:2000157	negative regulation of ubiquitin-specific protease activity	1	2
6762	GO:2000209	regulation of anoikis	1	1
6763	GO:2000210	positive regulation of anoikis	1	6
6764	GO:2000221	negative regulation of pseudohyphal growth	1	2
6765	GO:2000232	regulation of rRNA processing	1	1
6766	GO:2000253	positive regulation of feeding behavior	1	2
6767	GO:2000254	regulation of male germ cell proliferation	1	3
6768	GO:2000269	regulation of fibroblast apoptotic process	1	2
6769	GO:2000270	negative regulation of fibroblast apoptotic process	1	3
6770	GO:2000274	regulation of epithelial cell migration, open tracheal system	1	11
6771	GO:2000275	regulation of oxidative phosphorylation uncoupler activity	1	2
6772	GO:2000277	positive regulation of oxidative phosphorylation uncoupler activity	1	2
6773	GO:2000289	regulation of photoreceptor cell axon guidance	1	4
6774	GO:2000324	positive regulation of glucocorticoid receptor signaling pathway	1	10
6775	GO:2000373	positive regulation of DNA topoisomerase (ATP-hydrolyzing) activity	1	6
6776	GO:2000392	regulation of lamellipodium morphogenesis	1	6
6777	GO:2000427	positive regulation of apoptotic cell clearance	1	8
6778	GO:2000465	regulation of glycogen (starch) synthase activity	1	1
6779	GO:2000491	positive regulation of hepatic stellate cell activation	1	4
6780	GO:2000507	positive regulation of energy homeostasis	1	1
6781	GO:2000574	regulation of microtubule motor activity	1	3
6782	GO:2000601	positive regulation of Arp2/3 complex-mediated actin nucleation	1	2
6783	GO:2000627	positive regulation of miRNA catabolic process	1	7
6784	GO:2000630	positive regulation of miRNA metabolic process	1	3

6785	GO:2000636	positive regulation of primary miRNA processing	1	3
6786	GO:2000649	regulation of sodium ion transmembrane transporter activity	1	1
6787	GO:2000688	positive regulation of rubidium ion transmembrane transporter activity	1	1
6788	GO:2000696	regulation of epithelial cell differentiation involved in kidney development	1	2
6789	GO:2000765	regulation of cytoplasmic translation	1	2
6790	GO:2000766	negative regulation of cytoplasmic translation	1	3
6791	GO:2000773	negative regulation of cellular senescence	1	3
6792	GO:2000778	positive regulation of interleukin-6 secretion	1	2
6793	GO:2000873	regulation of histone H4 acetylation involved in response to DNA damage stimulus	1	1
6794	GO:2000899	xyloglucan catabolic process	1	1
6795	GO:2001034	positive regulation of double-strand break repair via nonhomologous end joining	1	6
6796	GO:2001213	negative regulation of vasculogenesis	1	3
6797	GO:2001246	negative regulation of phosphatidylcholine biosynthetic process	1	6
6798	GO:2001252	positive regulation of chromosome organization	1	16
6799	GO:2001268	negative regulation of cysteine-type endopeptidase activity involved in apoptotic signaling pathway	1	2
6800	GO:2001300	lipoxin metabolic process	1	1

Supplementary Table S2. List of GO terms associated with various biological processes of fatty acid (A), lipid (B) and others (C) in oil palm.

No.	Gene	Labelled as	Source	Reference /targeted gene/isoform	TA (ºC)	Amplicon length (bp)	Primer	Sequence (5'->3')	Primer length (bp)
1	*Polyubiquitin*	*UBIQUITIN*	Chan *et al.* (2014)	Reference gene	63	130	Forward	CCAGGCCAATCTCTCAGGATG	21
							Reverse	GGGGGATGCCCTCTTTATCC	20
2	*Alpha-tubulin1*	*TUBULIN*	Chan *et al.* (2014)	Reference gene	60	109	Forward	CATGGCTTGCTGCCTTATGTATC	23
							Reverse	AGGACACCAGTCAACAAACTGGA	23
3	*Gibberellin-responsive protein*	*GRAS*	Yeap *et al.* (2014)	Reference gene	54	103	Forward	CGGAAGAGTCACATCAATCG	20
							Reverse	GGAGCAAGAAGCCAACAC	18
4	*NADH dehydrogenase subunit5*	*NAD5*	Chan *et al.* (2014)	Reference gene	60	112	Forward	CATTTCTGGTTCACACGACTTCAG	24
							Reverse	AGAGAGTAAAACGACCCGAAATCC	24
5	*predicted protein IFH-1*	*pOP-EA01332*	Chan *et al.* (2014)	Reference gene	60	111	Forward	AAACGAAGGTACGGCAAGTACAAG	24
							Reverse	CTTAGCACATGCAGAGCAGATGTT	24
6	*Glyceraldehyde-3-phosphate dehydrogenase*	*GAPDH*	Chan *et al.* (2014)	Reference gene	60	124	Forward	GATCGAGAAATCAGCCACGTATG	23
							Reverse	GTCACCAATAAAGTCGGTGGACA	23
7	*Cyclophilin 2*	*Cyp2*	Yeap *et al.* (2014)	Reference gene	54	163	Forward	CTCGTCTGATGTCGTCTA	18
							Reverse	CTGCTGGTACTCTGGTAA	18
8	*Actin*	*ACTIN*	Chan *et al.* (2014)	Reference gene	60	147	Forward	TGCTGATCGTATGAGCAAGGAAA	23
							Reverse	GAAATCCACATCTGCTGGAAGGT	23
9	*Palmitoyl-ACP thioesterase*	*FATB_1*	Present study	Targeted gene	58	150	Forward	GCTGCAGCTGGAATGTG	17
							Reverse	CTGGCATAGTCGAGAATGGA	20
10	*Palmitoyl-ACP thioesterase*	*FATB_1cd*	Present study	Targeted isoforms	62	104	Forward	TGTTGGAGCAGCCAAGGTTA	20
							Reverse	GAGGCGGCAATTGAAGCAA	19
11	*Palmitoyl-ACP thioesterase*	*FATB_1bcd*	Present study	Targeted isoforms	63	132	Forward	CACCGAACAGGCCAAACCATCT	22
							Reverse	TGCAGCACGTTCCACAAAGTAAGG	24
12	*Long-chain acyl-CoA synthetases 4*	*LACS4_1ab cefg*	Present study	Targeted isoforms	62	135	Forward	CGTGGAGAAATTTGCATCAGGGG	23
							Reverse	TAAGCTTCCGTCTGCTTGCCA	21
13	*Long-chain acyl-CoA synthetases 4*	*LACS4_1b*	Present study	Targeted isoform	60	139	Forward	CGTTAACCAACCCATCTTTCGTTC	24
							Reverse	CTGCATCTTGAACACACCTCTC	22

14	*Long-chain acyl-CoA synthetases 4*	*LACS4_1bc*	Present study	Targeted isoforms	58	128	Forward	ATGTCTGTGGAGAAGAATCCC	21
							Reverse	GAAGCCCCAACTTTCATCAC	20
15	*Long-chain acyl-CoA synthetases 4*	*LACS4_1d*	Present study	Targeted isoform	63	80	Forward	TCATGGGGCCTCAATTGGAT	20
							Reverse	GGTTTTAGCACCTTGAATCCTTGA	24

Supplementary Table S3. Primer information for the reference genes used in this study.
